# Variation in secondary metabolite production potential in the *Fusarium incarnatum-equiseti* species complex revealed by comparative analysis of 13 genomes

**DOI:** 10.1186/s12864-019-5567-7

**Published:** 2019-04-24

**Authors:** Alessandra Villani, Robert H. Proctor, Hye-Seon Kim, Daren W. Brown, Antonio F. Logrieco, Maria Teresa Amatulli, Antonio Moretti, Antonia Susca

**Affiliations:** 1 Institute of Sciences of Food Production, National Research Council, Bari, Italy; 2 Department of Agriculture Peoria, National Center for Agricultural Utilization Research, U.S., Peoria, IL USA; 3 Thales Alenia Space Italia, Torino, Italy

**Keywords:** *Fusarium incarnatum*-*equiseti* species complex, Comparative genome analyses, Secondary metabolite genes, Phylogeny, Horizontal gene transfer

## Abstract

**Background:**

The *Fusarium incarnatum*-*equiseti* species complex (FIESC) comprises 33 phylogenetically distinct species that have been recovered from diverse biological sources, but have been most often isolated from agricultural plants and soils. Collectively, members of FIESC can produce diverse mycotoxins. However, because the species diversity of FIESC has been recognized only recently, the potential of species to cause mycotoxin contamination of crop plants is unclear. In this study, therefore, we used comparative genomics to investigate the distribution of and variation in genes and gene clusters responsible for the synthesis of mycotoxins and other secondary metabolites (SMs) in FIESC.

**Results:**

We examined genomes of 13 members of FIESC that were selected based primarily on their phylogenetic diversity and/or occurrence on crops. The presence and absence of SM biosynthetic gene clusters varied markedly among the genomes. For example, the trichothecene mycotoxin as well as the carotenoid and fusarubin pigment clusters were present in all genomes examined, whereas the enniatin, fusarin, and zearalenone mycotoxin clusters were present in only some genomes. Some clusters exhibited discontinuous patterns of distribution in that their presence and absence was not correlated with the phylogenetic relationships of species. We also found evidence that cluster loss and horizontal gene transfer have contributed to such distribution patterns. For example, a combination of multiple phylogenetic analyses suggest that five NRPS and seven PKS genes were introduced into FIESC from other *Fusarium* lineages.

**Conclusion:**

Our results suggest that although the portion of the genome devoted to SM biosynthesis has remained similar during the evolutionary diversification of FIESC, the ability to produce SMs could be affected by the different distribution of related functional and complete gene clusters.

**Electronic supplementary material:**

The online version of this article (10.1186/s12864-019-5567-7) contains supplementary material, which is available to authorized users.

## Background

The genus *Fusarium* includes some of the most destructive plant pathogens of food and feed crops and produces some of the mycotoxins of greatest concern to food and feed safety. Collectively, *Fusarium* species (fusaria) are pathogenic on most economically important crops and produce structurally diverse secondary metabolites (SMs), including mycotoxins that have adverse health effects, including immune suppression and cancer [1, 2]. Phylogenetic analysis has resolved the genus into 22 species complexes and seven monotypic lineages that together include over 300 phylogenetically distinct species [3–5].

Advances in Next Generation Sequencing technologies and bioinformatics software have provided powerful tools to expand understanding of variation in genomes within and among lineages of *Fusarium* [6–9]. Two recent studies focused on variation within single lineages. The first study compared 10 isolates of the *Fusarium graminearum* species complex and highlighted the extent of genetic diversity and similarity among the genomes [9]. For example, of the 15,297 genes present in the pan-genome of these fungi, 12% were absent in at least one species. Furthermore, the analyses identified 163 pan-genes that exhibited high variability among genomes that was consistent with allelism and could reflect a role in niche adaptation and disease. The second study compared five members of the *F. fujikuroi* species complex and revealed that although a large percentage of SM biosynthetic genes were conserved among the fungi examined, there were species and isolate-specific differences in gene content and expression [8]. The authors concluded that the differences had potential to affect host specificity as well as the pathogenic versus endophytic lifestyles of the fungi.

Members of the *Fusarium incarnatum*-*equiseti* species complex (FIESC) are cosmopolitan soil inhabitants, but can also occur on aerial plant parts, and are often recovered along with plant pathogens in field surveys of cereals, fruits, and vegetables [10]. FIESC members are regarded as moderately aggressive plant pathogens and are associated with human and insect infections as well [11–15]. Collectively, FIESC species can produce multiple mycotoxins, including apicidin [16], beauvericin [17], butenolide [18], enniatins [19], equisetin [20], trichothecenes [21, 22], fusarochromanone [23] and zearalenone [24].

FIESC has been resolved into 33 phylogenetically distinct species that group into two major clades, designated *Equiseti* and *Incarnatum*, using DNA-based Genealogical Concordance Phylogenetic Species Recognition (GCPSR) [14, 22, 25, 26]. Most species within the complex have not been formally described and are referred to with the designations FIESC 1 – FIESC 33 rather than with Latin binomials. However, three of the species have Latin binomials: *F. equiseti* (FIESC 14), *F. lacertarum* (FIESC 4) and *F. scirpi* (FIESC 9) [14]. Furthermore, analyses indicate that *F. camptoceras* likely represents a third lineage of FIESC [22, 27].

Although chemical analyses indicate that various members of FIESC produce the SMs noted above, production of all eight SMs has not been reported in all FIESC members. Overall, little is known about the variation in SM biosynthetic gene clusters within the complex. At present, only one FIESC genome (strain CS3069, a member of phylogenetic species FIESC 5) is publically available [28]. The presence of 11 polyketide synthase (PKS) genes and 13 non-ribosomal peptide synthetase (NRPS) genes in the CS3069 genome sequence suggest that the SM production potential of FIESC is much greater than what has been observed by chemical analyses [8, 29, 30].

A number of studies suggest that SM gene content varies among FIESC members and between FIESC and other lineages of *Fusarium*. One study revealed marked differences in the trichothecene biosynthetic gene (*TRI*) cluster and other *TRI* loci in FIESC versus other trichothecene-producing fusaria, such as *F. graminearum* and *F. sporotrichioides* [27]. The differences include: translocation of three genes (*TRI3*, *TRI7*, and *TRI8*) within the *TRI* cluster; translocation of two genes, *TRI1* and *TRI101*, into the FIESC *TRI* cluster from other genomic locations; absence of the trichothecene transporter gene *TRI12* in FIESC; and the presence of a novel Zn_2_Cys_6_ transcription factor gene in the FIESC *TRI* cluster that is absent in other trichothecene-producing fusaria [27]. Other studies provide evidence that production of trichothecenes and other mycotoxins differs among phylogenetic species and haplotypes within FIESC [21, 22, 31]. However, the genetic bases of such variation have not yet been investigated. In addition, the extent of variation among FIESC members irrespective of SM biosynthetic genes remains largely unknown. In the current study, therefore, we employed a comparative genomic approach to examine the distribution and variability of SM biosynthetic genes among 13 members of FIESC. The results indicate that the percent of the genome likely involved in SM synthesis in FIESC is similar to other fusaria, and that there is considerable variation in potential for production of SMs among FIESC members. Our results also revealed genetic variation within FIESC that is consistent with previously observed phenotypic variability.

Although multiple studies have examined the diversity of secondary metabolite biosynthetic genes and gene clusters within lineages of fungi within a genus [8, 9], these studies have not systematically explored possible evolutionary processes that have contributed to the diversity. Therefore, in the current study, we generated genome sequences for a subset of members of FIESC, examined diversity and distribution of secondary metabolite biosynthetic genes in FIESC genomes, and then investigated evolutionary processes that have likely contributed to the observed distribution using a combination of data from FIESC and other fusaria.

## Results

### Genome sequence

The twelve FIESC strains selected for genomic sequence analysis in this study represent a wide range of the phylogenetic diversity that exists within FIESC and were isolated from a broad range of host plants [22]. Analysis of the genome-sequence data indicated that the genomes were similar in size, ranging from 36.7 Mb in *F*. *camptoceras* to 39.9 Mb in *F*. *equiseti*. This range of sizes is comparable to the 38-Mb genome previously described for FIESC 5 strain CS3069 [28] as well as to other previously described genomes for species in other lineages of *Fusarium*: e.g., 41.5–43.1 Mb for *F. avenaceum*, 43.9 Mb for *F. fujikuroi* [7], 36 Mb for *F. graminearum* [32], and 41.8 Mb for *F. verticillioides* [6] (Table 1). The FIESC genome sizes are substantially less than those of *F. oxysporum*, *F. poae* and *F. solani* f. sp. *pisi* (46–60 Mb), which have large accessory genomes: that is genomic regions not shared by related species and in some cases by different strains of the same species, or duplicated genes, transposons and repetitive sequences [6, 33, 34].

**Table 1:** Assembly statistics for draft genome sequences of the 13 FIESC strains analyzed in this study

Species	Strain designation	Alternative Designation	GenBank Accession No.^a^	Genome Size (Mb)	No. Contigs	N50	GC (%)
*F. camptoceras*	NRRL 13381	FRC R-5200	QGED00000000	36.6	601	209,627	48.44
*F. equiseti*	ITEM 11363	NRRL 66338	QGEB00000000	40.0	669	233,726	48.46
*F. scirpi*	NRRL 66328	FRC R-06979	QHHJ00000000	39.5	1035	168,857	48.42
FIESC 5	ITEM 11348	NRRL 66337	QGEC00000000	38.6	880	157,961	48.58
FIESC 5	CS3069	NRRL 62617	CBMI000000000	38.0	5111	–	–
FIESC 12	ITEM 11294	NRRL 66336	QHHI00000000	39.6	1502	75,753	48.57
FIESC 15	NRRL 31160	–	QGEA00000000	37.4	634	153,141	48.64
FIESC 23	ITEM 7155	NRRL 66325	QGDZ00000000	37.5	421	291,968	48.66
FIESC 25	ITEM 6748	NRRL 66324	QGDY00000000	37.2	560	213,588	48.62
FIESC 28	ITEM 1616	NRRL 66322	QGDX00000000	37.0	562	199,056	48.62
FIESC 29	ITEM 10392	NRRL 66334	QHHH00000000	37.9	471	225,592	48.53
FIESC 33	ITEM 10395	NRRL 66335	QHHG00000000	39.0	593	183,362	48.56
FIESC 33	ITEM 11401	NRRL 66339	QHKN00000000	39.2	806	208,659	48.55

^a^With the exception of FIESC 5 strain CS3069, genome sequences were generated during the course of this study using a MiSeq Illumina platform. The sequence for CS3069 was generated previously [28] and was downloaded from the National Center for Biotechnology Information website

### Species phylogeny

**Fig. 1:**
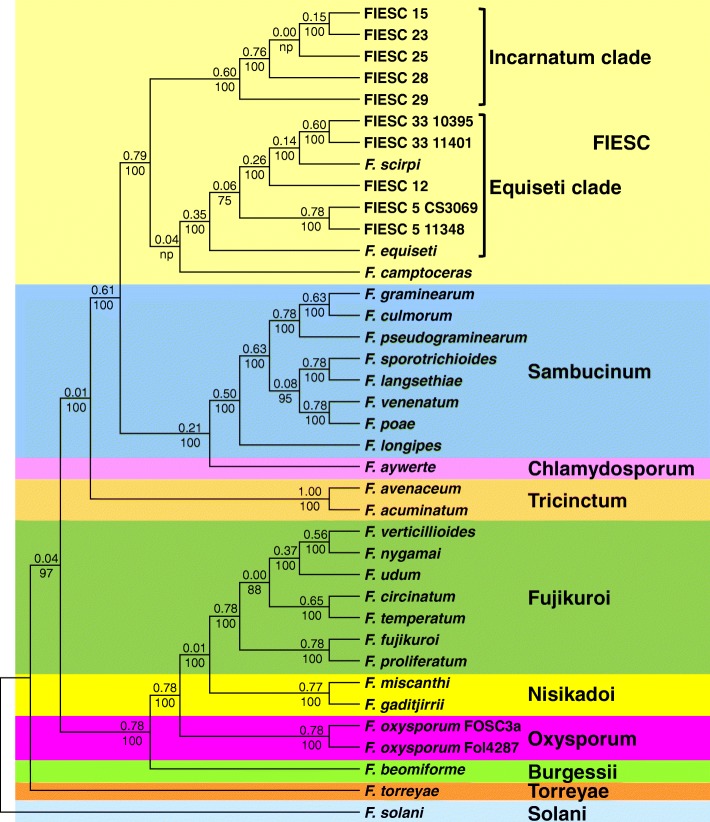
*Fusarium* species tree inferred using the extended majority rule consensus method from individual maximum likelihood trees of 30 housekeeping genes (Additional file 1). Values above branches are internode certainty values, and values below branches are bootstrap values based on 1000 replicates generated during maximum likelihood analysis of concatenated sequences of the 30 housekeeping genes. Colored boxes demarcate species complexes and the names of the complexes are indicated to the right using unitalicized specific epithets of the species after which each complex is named [3]

To assess phylogenetic relationships of FIESC members to one another and to other fusaria, we inferred a species tree that included the 13 FIESC members and 24 species from nine other *Fusarium* species complexes (Fig. 1). For brevity here, we will refer to species complexes, except FIESC, using the specific epithets upon which the complex names are based (e.g., Sambucinum complex instead of the *F. sambucinum* species complexes). The species tree was generated by the extended majority rule (MRE) consensus approach using Maximum Likelihood (ML) trees inferred separately for each of 30 housekeeping (HK) genes (Additional file 1). For evaluation of branch support, we used internode certainty and bootstrap analyses. The latter were obtained by ML analysis of concatenated sequences of the 30 HK genes. Internode certainty values varied from 0.00 to 0.79, while bootstrap values for almost all branches were 100. Even though the internode certainty and bootstrap analyses indicated marked differences in levels of support for some branches, the topologies of the MRE consensus tree and the ML tree inferred from concatenated sequences were largely the same (Fig. 1; Additional file 2).

In the species tree, FIESC members were resolved as an exclusive clade, as were members of the previously described *Incarnatum* and *Equiseti* clades within FIESC (Fig. 1). *F. camptoceras* was sister to the *Equiseti* clade in the species tree, but support for this relationship was low (internode certainty value = 0.04). In the concatenated-sequence tree, *F. camptoceras* was basal to all other members of FIESC, but with low support (bootstrap value = 72; Additional file 2). The two most basal branches of the *Incarnatum* clade, FIESC 29 and FIESC 28, were more highly supported than other branches in this clade, according to the internode certainty values. Within the *Equiseti* clade, the internode certainty values for all branches were relatively low, except for the branches leading to the two FIESC 5 and two FIESC 33 strains. The Sambucinum, Tricinctum, Fujikuroi, Nisikadoi and Oxysporum complexes were each represented by two or more members, and were also resolved as exclusive clades in the species tree. The Solani and Torreyae complexes were each represented by one member, and formed the most basal branches in the tree (Fig. 1). The tree topology indicated that FIESC was most closely related to the Chlamydosporum (represented by *F. aywerte*) and Sambucinum complexes. Support for the FIESC-Chlamydosporum-Sambucinum branch was relatively high (internode certainty = 0.61). This close relationship of FIESC to the Chlamydosporum and Sambucinum complexes relative to other lineages of *Fusarium* is consistent with previously reported phylogenetic analyses [3, 15, 29, 35].

### Secondary metabolite biosynthetic genes

**Fig. 2:**
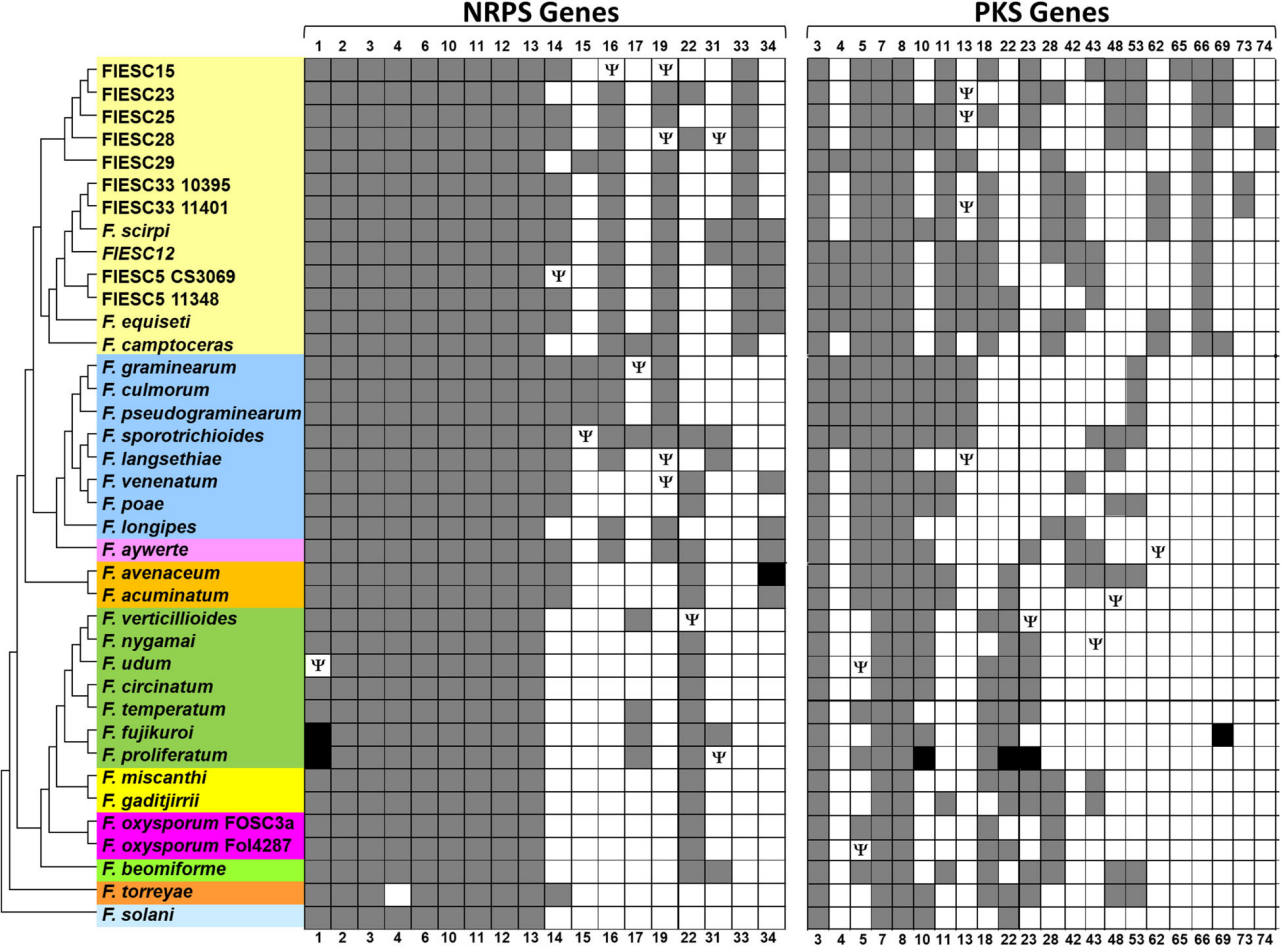
Distribution of NRPS and PKS genes among *Fusarium* species based on BLASTn analysis of genome sequences. A gray box indicates that an apparently functional copy of the gene was present in the genome of species/strain indicated in the species tree to the left. A white box indicates that the gene was absent. A white box with the Greek letter Ψ indicates that only a pseudogene was detected in the genome. A black box indicates that an apparently functional gene was present in one or more strains of a species but was absent (or present as a pseudogene) in one or more other strains of the same species. Note that we analyzed multiple strains of only a small number of species; in addition to the two strains of *F. oxysporum*, FIESC 5 and FIESC 33 included in the species tree, we analyzed publicly available genomes of two or more strains of *F. avenaceum*, *F. fujikuroi*, *F. graminearum*, *F. oxysporum*, *F. pseudograminearum* and *F. proliferatum*. The species tree shown to the left is derived from (i.e., includes the same species and has the same topology) as the species tree in Fig. 1

BLAST analysis indicated that there were 9–13 PKS genes and 11–15 NRPS genes per FIESC genome (Fig. 2, Additional file 3). According to antiSMASH analysis, there were 33–42 SM biosynthetic gene clusters per FIESC genome (Table 2). This range is comparable to the numbers of clusters in other *Fusarium* genomes [6–8, 30]. Overall, 3–4% of the genes in each FIESC genome were predicted by antiSMASH to be SM biosynthetic genes (Table 2). The exception to this was FIESC 5 CS3069, in which SM biosynthetic genes were estimated to constitute only 1.6% of the genes. Homologs from approximately 50% of the PKS and NRPS ortholog groups were present in all FIESC genomes examined (Fig. 2). The fusarubin (*PKS3*), malonichrome (*NRPS1*), ferricrocin (*NRPS2*), and fusarinine (*NRPS6*) biosynthetic gene clusters were among the clusters that were present in all the FIESC genomes examined, as were four terpene biosynthetic genes clusters that included the TS gene required for synthesis of α-acorenol (*STC6*), koraiol (*STC4*), carotenoids (*TeTC1*), or trichothecenes (*TRI5*) (Fig. 2, Additional file 3). PKS and NRPS genes that were not present in all FIESC genomes often exhibited discontinuous patterns of distribution in that their presence and absence was not correlated with the phylogenetic relationships of the species in which they occurred. Some of these latter PKS and NRPS genes were present in both the *Equiseti* and *Incarnatum* clades (Fig. 2). For example, *PKS28* was present in five members of the *Equiseti* clade and two members of the *Incarnatum* clade, as well as *F. camptoceras*. Other PKS and NRPS genes were present in only one FIESC strain examined: i.e., *PKS65* was only in FIESC 15; *NRPS15* was only in FIESC 29; and *NRPS17* was only in *F. camptoceras*. Although *PKS65* was found in only FIESC 15, homologs of this gene have been reported previously in *F. redolens* and *F. babinda* [29]. Similarly, *NRPS15* and *NRPS17* were found in other *Fusarium* species (Fig. 2). In contrast, *PKS62*, *NRPS33*, and two novel PKS, hereafter named *PKS73* and *PKS74*, have not been reported in other fusaria so far, and therefore could be unique to FIESC.

**Table 2:** antiSMASH-based estimates of secondary metabolite (SM) biosynthetic gene clusters and proportion of genome involved in SM biosynthesis

Species	Strain No.	No. SM Clusters	Genome Size (Mb)	Mb of SM Clusters	SM Clusters as % of Genome	No. Predicted Genes	No. Predicted SM Genes	SM Genes as % of Genome
Species from current study								
*F. camptoceras*	NRRL 13381	39	36.8	1.6	4.3	12,018	512	4.3
*F. equiseti*	11,363	40	40.0	1.6	4.1	12,905	521	4.0
*F. scirpi*	NRRL 66328	43	39.5	1.7	4.4	13,071	560	4.3
FIESC5	11,348	36	38.6	1.4	3.6	12,723	459	3.6
FIESC12	11,294	42	39.6	1.4	3.5	13,219	433	3.3
FIESC15	NRRL 31160	36	37.4	1.4	3.7	12,421	442	3.6
FIESC23	7155	34	37.5	1.4	3.7	12,325	446	3.6
FIESC25	6748	35	37.2	1.4	3.8	12,175	449	3.7
FIESC28	1616	37	37.0	1.3	3.7	12,168	456	3.7
FIESC29	10,392	33	37.9	1.3	3.3	12,469	417	3.3
FIESC33	10,395	39	39.0	1.6	4.1	12,810	522	4.1
FIESC33	11,401	38	39.2	1.6	4.1	12,819	506	3.9
Species from Hansen et al. [30]								
FIESC 5	CS3069	37	38.1	0.7	1.9	13,047	215	1.6
*F. acuminatum*	CS5907	55	44.0	1.6	3.6	14,516	461	3.2
*F. avenaceum*	Fa05001	63	41.5	2.8	6.8	13,214	831	6.3
*F. culmorum*	CS7071	42	37.7	1.3	3.5	11,922	393	3.3
*F. fujikuroi*	IMI58289	50	43.8	2.2	4.9	13,692	607	4.4
*F. graminearum*	PH-1	42	36.4	1.9	5.2	11,683	604	5.2
*F. oxysporum*	4287	50	60.2	2.0	3.4	18,382	664	3.6
*F. pseudograminearum*	CS3096	38	36.3	1.7	4.5	11,721	511	4.4
*F. solani*	77–13-4	38	51.2	1.6	3.1	16,410	523	3.2
*F. verticillioides*	7600	49	41.1	2.1	5.1	13,701	931	6.8

The fusaridione (*PKS69*) cluster and clusters with *PKS23*, *PKS48* or *PKS58* gene occurred only in the *Incarnatum* clade, while a cluster that included *PKS42* and *NRPS34* occurred only in the *Equiseti* clade (Fig. 2). The apicidin cluster [36] was present in one member of the *Incarnatum* clade and two members of the *Equiseti* clade (Additional file 3). A butenolide cluster has not been functionally characterized and most likely does not include a PKS, NRPS or TS gene, however, a monooxygenase gene required for butenolide synthesis and a putative biosynthetic cluster have been identified in *F. graminearum* [37]. Homologs of the monooxygenase gene required for butenolide synthesis were present in two members of the *Incarnatum* clade (FIESC 15 and 25) and one member of the *Equiseti* clade (FIESC 33). Thus, some members of FIESC may be able to produce butenolide. Furthermore, the entire fusarin biosynthetic gene cluster was present in three FIESC members, indicating that these fungi have the potential to produce fusarins (Additional file 3).

**Fig. 3:**
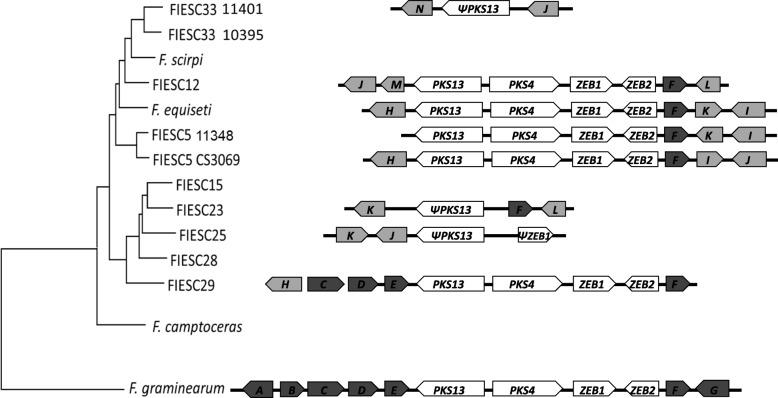
Organization of the ZEA gene cluster and flanking genes. The arrows represent the indicated genes while the direction of the arrow shows direction of transcription: Genes A, B, C, D, E, F, G, H, I, J, K, L, M, and N correspond to FGSG_02392, FGSG_02393, FGSG_02394, FGSG_12124, FGSG_12125, FGSG_02399, FGSG_02400, FGSG 11341, FGSG_07712, FGSG_04615, FGSG_11645, FPSE_12200, FVEG_13785, and FGSG 02196, respectively. FIESC homologs A, B, C, D, E, F, G, H, J, M, N, and I share > 70% identity while FIESC homologs of J and K share < 50% identity

An intact zearalenone biosynthetic gene (ZEA) cluster [38] was present in three members of the *Equiseti* clade and one member of the *Incarnatum* clade, while degenerated ZEA cluster homologs (i.e., partial cluster consisting of one or two pseudogenized ZEA genes) were present in one member of the *Equiseti* clade and two members of the *Incarnatum* clade (Fig. 3). Likewise, part of the equisetin biosynthetic gene cluster appears to have been deleted in some members of FIESC. In *F. graminearum*, the equisetin cluster consists of *PKS18* and ten other genes [39]. Intact homologs of the cluster occur in most of the FIESC strains examined here. However, in FIESC 23, 28, and 29, six of the cluster genes, including *PKS18*, are absent.

Genes and gene clusters responsible for synthesis of multiple mycotoxins and other SMs produced by other fusaria were not present in any of the FIESC genomes examined. These included genes or clusters responsible for synthesis of the polyketide-derived metabolites aurofusarin, bikaverin, depudecin, fujikurin, fumonisins, and W493 B, and the terpenoid metabolites eremophilene, guaia-6,10(14)-diene, and gibberellins. Synthesis of the terpenoid mycotoxin culmorin requires a TS and a cytochrome P450 monooxygenase encoded by *CLM1* and *CLM2*, respectively [40]. None of the FIESC strains examined had both of these genes, however, FIESC 23 had a *CLM1* homolog (Additional file 3).

### Homolog of endocrocin cluster in FIESC

**Fig. 4:**
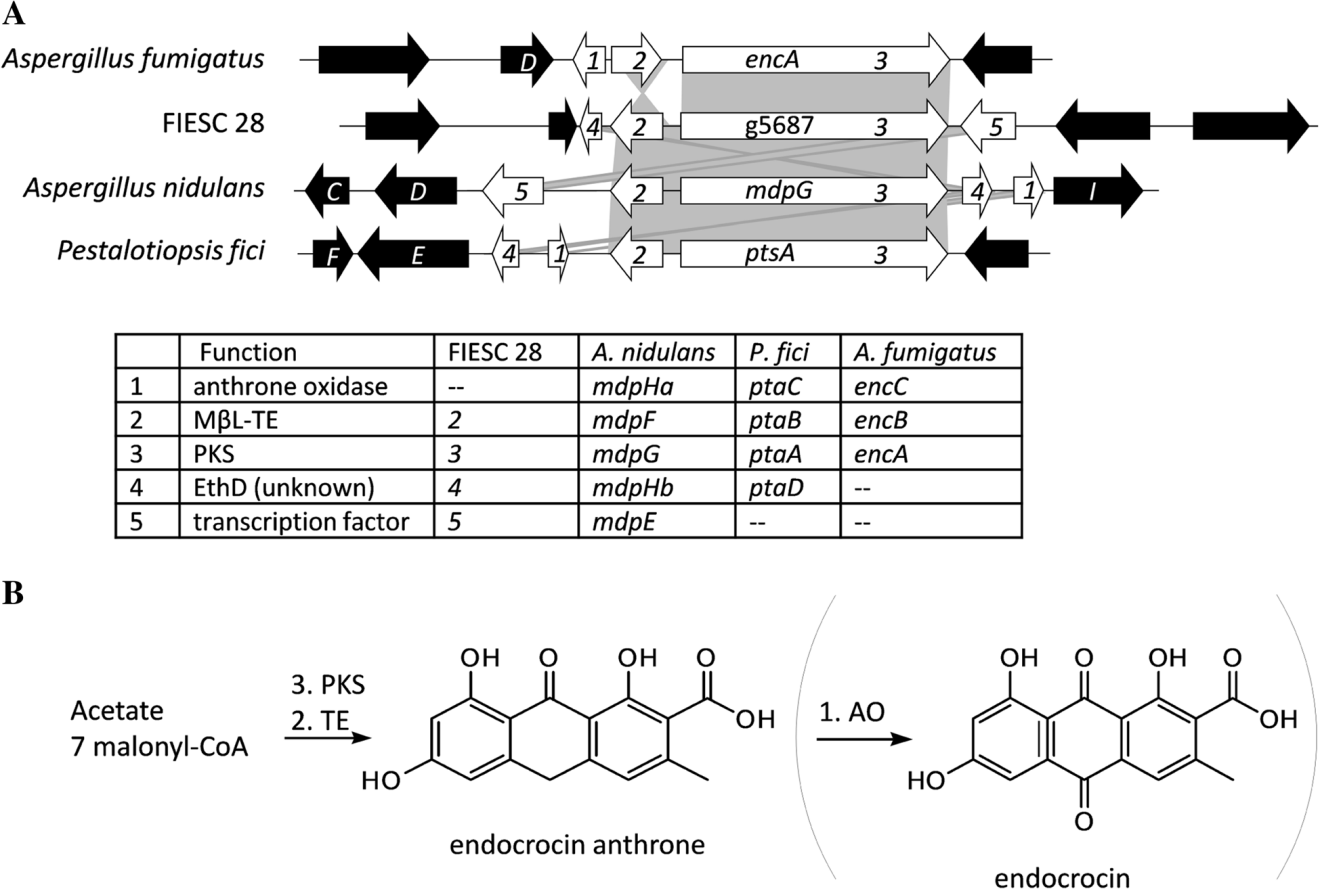
**a**. Organization of homologs of the endocrocin biosynthetic gene cluster in *Aspergillus* species, FIESC 28 and *Pestalotiopsis fici*. Blue arrows represent homologous genes present in at least two fungi. Direction of arrows indicate direction of transcription. Abbreviations for gene/protein functions are as follows: AO, anthrone oxidase; TE, metallo-β-lactamase type thioesterase (MβL-TE); PKS, polyketide synthase; TF, = transcription factor, UN, gene of unknown function. The numbers in blue arrows indicate homologs. Black arrows indicate genes that are considered part of the biosynthetic cluster in the respective fungi but that were not present in FIESC 28. **b**. Proposed biosynthetic pathway for endocrocin anthrone in FIESC 28 and for endocrocin in the other fungi

As noted above, *PKS73* in FIESC 33 and *PKS74* in FIESC 28 represent novel PKS clades in *Fusarium*. To determine whether closely related homologs of these *PKS* genes occur in other fungi, we did BLASTx analyses using *PKS73* and *PKS74* sequences as queries against the NCBI fungal protein database. No homologs closely related to *PKS73* were identified by this approach, but homologs closely related to *PKS74* were identified in multiple fungi. The *PKS74* homologs included *encA* (AFUA_4G00210) from *Aspergillus fumigatus*, *mdpG* (AN0150) from *A. nidulans*, and *ptaA* (AG059040) from *Pestalotiopsis fici*, three PKS genes that are required for the synthesis of endocrocin anthrone [41–43]. In these fungi, endocrocin anthrone serves as a biosynthetic precursor to more structurally complex SMs. The other enzymes in the endocrocin biosynthetic pathway are encoded by two to four genes located immediately up or downstream of the PKS genes (Fig. 4). In FIESC 28, homologs of three of these genes are adjacent to *PKS74* (Fig. 4).

The endocrocin anthrone PKS belongs to PKS group V, a clade of NR-PKSs that lack a thioesterase domain and, thus, require a second protein, a metallo-β-lactamase type thioesterase (MβL-TE), for release of the nascent polyketide from the PKS [44]. In FIESC 28, the gene adjacent to *PKS74* is predicted to encode a MβL-TE (Fig. 4a) which could catalyze release of an endocrocin-like polyketide from *PKS74*. In a previous study, group V PKSs were observed in 39 genera of Ascomycetes, but not in *Fusarium* [44]. The presence of *PKS74* in FIESC 28, therefore, constitutes the first report of a group V PKS in *Fusarium*.

### Phylogenetic relationships of FIESC PKSs

**Fig. 5:**
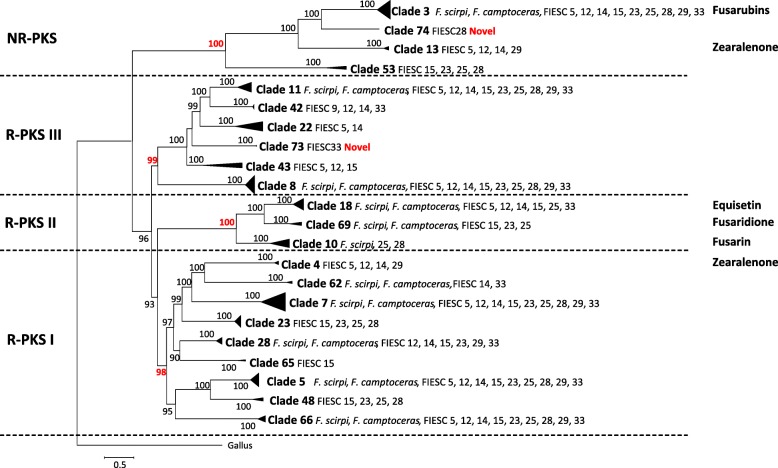
Phylogeny of PKS genes in FIESC. The condensed phylogenetic tree was generated by maximum likelihood analysis of the entire amino acid sequences of the coding regions predicted from 146 PKS enzymes identified in 13 FIESC genomes. All the homologous PKSs from Brown and Proctor 2016 were included in the analysis. The *Gallus gallus* fatty acid synthase (FAS) gene was used as outgroup. Clades corresponding to the three previously described major clades of reducing PKSs (R-PKS I, R-PKS II and R-PKS III) and the one major clade of non-reducing PKSs were resolved in this analysis with high levels of bootstrap support (in red type) and are delineated with horizontal lines

Our analyses identified 146 PKS genes in the 13 FIESC genomes examined in this study. Phylogenetic analysis of these PKSs plus 216 PKSs from other fusaria resolved the 146 FIESC PKSs into four large clades that corresponded to the non-reducing PKS (NR-PKS) and three reducing PKS (R-PKS I, II and III) groups (Fig. 5; Additional files 4 and 5) [29, 45]. The four clades further resolved into 22 well-supported smaller clades. Twenty of these smaller clades corresponded to clades recently described in an analysis of *Fusarium* PKSs [29] while clade 74 and clade 73, appear to be novel. Clade 74 consists of a single ortholog from FIESC 28 and is embedded within the NR-PKS group, while clade 73 consists of two orthologs from the FIESC 33 strains and is embedded within the R-PKS III group. PKS clade 62 was previously described based on a single PKS homolog from *F. scirpi* [29]. Here, we identified four additional clade 62 homologs: one in *F. equiseti* (FIESC 14), one in each of the two FIESC 33 strains, and one in *F. camptoceras*. PKS homologs present in six clades (clades 3, 5, 7, 8, 11 and 66) were present in all 13 FIESC genomes examined.

### Phylogenetic analysis of FIESC NRPSs

**Fig. 6:**
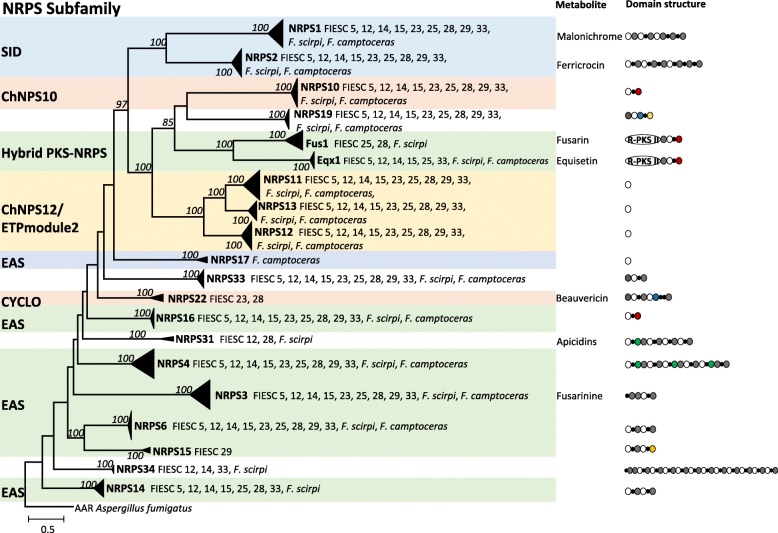
ML phylogenetic tree of adenylation domains from NRPSs and hybrid PKS-NRPS. Numbers at nodes indicate bootstrap value greater than 70%, performed with 1000 replications. Name of subfamilies are shown to the left [46]. Known products and domain structures [30] are shown on the right

The NRPS analysis included A domains from 269 NRPS genes recovered from the 12 FIESC genome sequences generated in this study and 95 NRPSs from other fusaria. In the resulting tree, the FIESC A domains were resolved into 20 well-supported clades, 15 of which corresponded to NRPS ortholog groups described by Hansen et a1. [30] (Fig. 6). The 20 A-domain clades could also be grouped into larger assemblages corresponding to the nine NRPS subfamilies described by Bushley and Turgeon [46]. Seven of the FIESC NRPS clades also corresponded to an even larger grouping consisting of mono/bimodular NRPSs described by Bushley and Turgeon [46]. This grouping consisted of the ChNPS10, ChNPS12, PKS:NRPS, and Cyclosporin synthetases (CYCLO) subfamilies. Eight other NRPS clades corresponded to the two subfamilies of multimodular enzymes: the siderophore synthetase (SID) and Euascomycete synthetase (EAS) subfamilies. Within the mono/bimodular enzyme group, clade *NRPS10* corresponded to the ChNPS10 subfamily; two clades consisting of the fusarin and equisetin PKS-NRPSs subfamily; clades *NRPS11*, *NRPS12* and *NRPS13* corresponded to the ChNPS12 subfamily; and clade *NRPS22* corresponded to the CYCLO subfamily. For the multimodular NRPSs, clades *NRPS1* and *NRPS2* corresponded to the SID subfamily, and clades *NRPS3*, *NRPS4*, *NRPS6*, *NRPS14*, *NRPS15*, and *NRPS17* corresponded to the EAS subfamily.

Most of the basal branches in the FIESC A-domain tree did not have significant bootstrap support, and because of this it was not possible to assign the *NPRS16*, *NRPS31* or *NRPS33* clades to subfamilies. There were some inconsistencies in the phylogenetic relationships inferred in our FIESC A-domain tree compared to those inferred by Bushley and Turgeon [46]. In the FIESC A-domain tree (Fig. 6), the multimodular subfamily SID formed a monophyletic lineage along with the mono/bimodular subfamilies ChNPS10, hybrid PKS-NRPS, and ChNPS12/ETPmodule2 (bootstrap value = 100). This contrasts with the results of Bushley and Turgeon [46], where the multimoldular subfamilies SID and EAS formed a monophyletic lineage (bootstrap value = 97). Also in the Bushley and Turgeon study, EAS was a monophyletic lineage, whereas in the current study EAS was not monophyletic. The results of the current study are, however, consistent with those of Gallo et al. [47] in that neither the mono/bimodular nor the multimodular NRPSs formed well supported monophyletic lineages. Thus, results of the Bushley and Turgeon study suggest that all multimodular NRPSs have a common evolutionary history distinct from mono/bimodular NRPSs, whereas results from the current study and Gallo et al. [47] suggest that this is not the case.

### Evolutionary forces acting upon distribution of SM gene clusters within FIESC

A combination of processes, including gene loss, gene duplication and horizontal gene transfer (HGT), likely contributed to the discontinuous distribution patterns and tree topologies observed for other NRPS/PKS genes. Thus, subsequent analyses were aimed at investigating which of these processes have contributed to the distribution of which NRPS/PKS genes in FIESC.

### Gene loss

To assess the contribution of gene loss to the distribution of NRPS/PKS genes in FIESC, we re-examined distribution patterns to determine which NRPS/PKS genes were present in one or more FIESC members but absent in closely related member. We found that six NRPS genes and 10 PKS genes exhibited such distribution patterns (Fig. 2). For example, *PKS18* was present in all members of the *Equiseti* clade, and only two members (FIESC 15 and FIESC 25) of the *Incarnatum* clade. This suggests the gene was present in the common ancestor of the two clades and was subsequently lost during divergence of the *Incarnatum* clade such that it is currently present only in FIESC 15 and FIESC 25.

We also used NOTUNG analysis to assess loss of NRPS/PKS genes in FIESC. In this analysis, ML trees inferred for each NRPS/PKS gene were reconciled with the species tree shown in Fig. 1. NOTUNG inferred 127–129 loss events of NRPS/PKS genes within FIESC to account for the differences between individual gene trees and the species tree (Additional file 6).

The presence of pseudogenized NRPS/PKS genes (i.e., genes with mutations that result in frameshifts, truncations and/or internal stop codons) in some FIESC members is also consistent with gene loss events. Within FIESC, we detected pseudogenized versions of *NRPS14*, *NRPS16*, *NRPS19*, *NRPS31* and *PKS13* in FIESC (Fig. 2). *PKS13* along with *PKS4*, *ZEB1* and *ZEB2* comprise the zearalenone biosynthetic gene cluster (Fig. 3). Intact homologs of this cluster were detected in four members of the Sambucinum complex and five members of FIESC, including one species in the *Incarnatum* clade and three species in the *Equiseti* clade. Among the other eight members of FIESC examined, three had a degenerate zearalenone cluster, which always included a pseudogenized *PKS13*, and five FIESC members did not have detectable full-length or pseudogenized zearalenone biosynthetic genes (Fig. 3).

### HGT- manual tree comparisons and reconciliation analysis

**Fig. 7:**
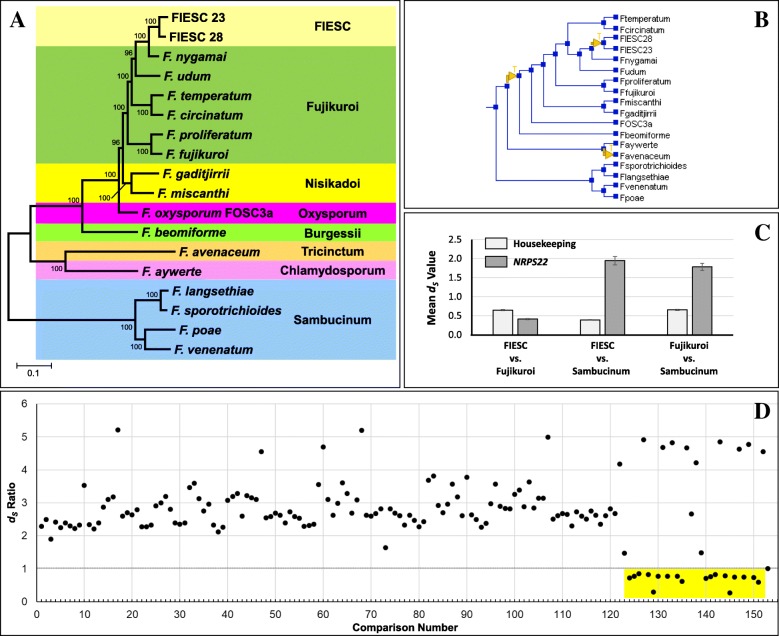
Results of phylogenetic analyses of the beauvericin/enniatin NRPS gene *NRPS22* (*ESYN1*) as an example of analyses used to assess HGT of NRPS and PKS genes. **a**. ML tree of *NRPS22* sequences from all species included in this study that have the gene. The colored boxes demarcate species complexes. Numerical values near branches are bootstrap values based on 1000 replications. **b**. Phylogenetic tree from NOTUNG reconciliation analysis using the species tree shown in Fig. 1 and default cost settings specified by NOTUNG. Yellow arrows indicate NOTUNG-inferred HGT events. **c**. Mean d_S_ values for *NRPS22* and HK genes from pairwise comparisons of members of FIESC and the Sambucinum and Fujikuroi species complexes shown in A. Plot of ratio of d_S_ values for *NRPS22*:HK genes (i.e., d_S_ ratio) for the 153 pairwise comparisons of taxa shown in A. The ratios at the bottom right of the plot that are highlighted with yellow are the only ratios that were less than 1 and correspond to comparison involving FIESC 23 or FIESC 25 with members of the Fujikuroi, Nisikadoi and Oxysporum complexes. The specific pairwise comparisons corresponding to the numbers along the X-axis are shown in the *NRPS22* tab of Additional file 7

We also examined the results of the phylogenetic analyses for evidence that HGT contributed to the introduction of NRPS/PKS genes in FIESC. Manual comparison of the NRPS/PKS gene trees to the species tree revealed 10 branch conflicts that were consistent with HGT of NRPS/PKS genes between FIESC and other *Fusarium* lineages (Table 3, Additional file 5). In eight of these putative HGT events, FIESC was the recipient of a gene. Figure 7 shows an example of a tree suggestive of HGT of *NRPS22*. In the species tree (Fig. 1), FIESC and the Fujikuroi complex were relatively distantly related lineages, but in the *NRPS22* tree FIESC 23 and FIESC 28 were nested within the Fujikuroi complex in the *NRPS22* tree (Fig. 7a). This branch conflict between the species and *NRPS22* trees is consistent with HGT of *NRPS22* from the Fujikuroi complex to FIESC.

**Table 3:** Putative horizontal gene transfer (HGT) events of NRPS and PKS genes between FIESC and other lineages of *Fusarium*. Grey highlighting indicates that results from all analyses were consistent with HGT

Gene	HGT Donor	HGT Recipient	Identification^a^		Additional Evidence for HGT^b^		
			Manual Tree Comparison	NOTUNG	Bootstrap	SH-AU	*d*_*S*_
*HGT Events with FIESC Recipient*							
*NRPS4*	Tricinctum complex	FIESC (Incarnatum clade)	+	+	100	+	+
*NRPS11*	Sambucinum complex (or close relative)	FIESC	–	+	–	–	–
*NRPS14*	Sambucinum complex (or close relative)	FIESC	–	+	100^c^	+	–
*NRPS16*	*F. longipes* (or close relative)	FIESC	+	+	77	–	±
*NRPS22*	Fujikuroi complex (African clade)	FIESC (Incarnatum clade)	+	+	100	+	+
*PKS10*	Tricinctum complex	*F. scirpi* (or recent ancestor)	+	+	100	+	+
*PKS10*	Sambucinum complex	FIESC (Incarnatum clade)	+	+	100	+	–
*PKS22*	*F. torreyae* relative	FIESC (Equiseti clade)	–	+	100	+	–
*PKS23*	Fujikuroi complex	FIESC (Incarnatum clade)	+	+	100^d^	+	+
*PKS48*	Tricinctum complex (*F. avenaceum* relative)	FIESC (Incarnatum clade)	+	+	100	+	–
*PKS62*^e^	Fujikuroi complex	FIESC	NA	NA	–	NA	+
*PKS69*^e^	Fujikuroi complex	FIESC	+^f^	NA	90	+	+
*HGT Events with FIESC Donor*							
*NRPS19*	FIESC	Sambucinum complex	+	+	74	+	±
*NRPS34*	FIESC (Equiseti clade)	*F. longipes*	–	+	–	–	±
*PKS22*	FIESC (Equiseti clade)	Tricinctum complex	+	+	100	+	–
*PKS42*	FIESC (Equiseti clade)	Ancestor of Sambucinum and Chlamydosporum complexes	–	+	–	–	±
*PKS43*	FIESC (Incarnatum clade)	Sambucinum complex (*F. sporotrichioides* ancestor)	–	+	–	–	±
*PKS43*	FIESC (Equiseti clade)	*F. avenaceum* (or recent ancestor)	–	+	–	–	±
*PKS43*	FIESC5 (or close relative)	*F. aywerte* (or recent ancestor)	–	+	–	–	±

^a^Putative HGT events were identified by manual comparison of individual NRPS and PKS gene trees to the species tree (Fig. 1) and by using the gene tree reconciliation program NOTUNG [76]. + indicates the method identified the putative HGT event, and – indicates the method did not identify the HGT event. For the Manual comparison column, ± indicates a branch conflict was identified, but that we considered an alternative hypothesis (i.e., an hypothesis that did not involved HGT to FIESC) was also plausible. NA indicates not applicable

^b^Three analyses were done to further assess evidence for HGT: bootstrap analysis, SH- AU tests, and estimates of number of synonymous substitutions per synonymous site (*d*_*S*_). In the Bootstrap column, numerical values correspond to the bootstrap values for the branch in the NRPS/PKS gene tree that conflicted with the species tree; and – indicates that the bootstrap value for the conflicting branch was < 70%, or that in our estimation the branch indicative of HGT in NOTUNG analysis did not conflict with the species tree. In the SH-AU column, + indicates that the constrained tree was significantly worse than the unconstrained tree; and – indicates that the constrained tree was not significantly worse than the unconstrained tree. In the *d*_*S*_ column, + indicates *d*_*S*_ ratio < 1; indicates *d*_*S*_ ratio > 1; and ± indicates comparisons for which *d*_*S*_ ratios < 1 may not be evidence of HGT, because over 50% of comparisons for the gene yielded *d*_*S*_ ratios < 1. NA indicates not applicable

^c^The bootstrap value of 100 was for a FIESC and Sambucinum complex branch that excluded *F. aywerte* (i.e., Chlamydosporum complex). From our manual comparison of the *NRPS14* tree with the species tree, we considered that the topology of the *NRPS14* tree could have resulted if the relationship of FIESC and Sambucinum complex sequences were concordant with the species tree, but the relationship of *F. aywerte* sequences to Sambucinum complex sequences was not concordant with the species tree

^d^This bootstrap value is for a clade that includes members of FIESC and the Fujikuroi and Nisikadoi complexes (See Additional file 5). The bootstrap value for the clade consisting of only FIESC and the Fujikuroi complex was < 70%, and therefore not significant. This in turn suggests that the donor of HGT of *PKS23* may have been an ancestor or other relative of the Fujikuroi and Nisikadoi complexes

^e^Some non-FIESC homologs used in the *PKS62* and *PKS69* analyses were not species included in the species tree inferred the current study. *Fusarium agapanthi* and *F. dlaminii* are members of the Fujikuroi complex [80], and *Fusarium* sp. NRRL 25184 (25184) is a member of the *F. newnesense* species complex, a lineage that is closely related to the Fujikuroi, Nisikadoi and Oxysporum complexes [81]

^f^This branch conflict was inferred by comparison of the *PKS69* tree to previously reported species trees showing the relationships of FIESC and *F. dlaminii* to one another and/or other lineages of *Fusarium* [3, 29]. The previous studies indicate that *F. dlaminii* and *F. fujikuroi* are both members of the Fujikuroi complex. In the *PKS69* tree, the *F. dlaminii* homolog is more closely related to FIESC homologs than to the *F. fujikuroi* homolog. Thus, the *PKS69* tree conflicts with the relationships of species

Reconciliation analysis using NOTUNG inferred 17 HGT events of NRPS/PKS genes between FIESC and other *Fusarium* lineages (Additional file 6). These NOTUNG-inferred events included all the HGT events inferred by the manual tree comparisons noted above, including the putative transfer of *NRPS22* from the Fujikuroi complex to FIESC (Fig. 7b). FIESC was the recipient in 10 of the NOTUNG-inferred HGT events. Thus, together NOTUNG reconciliation and manual comparison of species and gene trees revealed 10 putative HGT events in which NRPS/PKS genes were transferred to FIESC from other *Fusarium* lineages (Table 3).

### HGT – Constraint analysis

Constraint analysis has been used previously to assess support for putative HGT events [48, 49]. Therefore, we used it to further assess the 17 putative HGT events of NRPS/PKS genes noted in Table 3. For this analysis, we manually altered the topology of NRPS/PKS gene trees so that individual branches that conflicted with the species tree in the unconstrained trees conformed to the species tree in the constrained trees (Additional files 5 and 7). We then used SH-AU tests to determine whether the constrained trees were less well supported than the corresponding unconstrained trees [48, 49]. For example, we generated two constrained trees from the unconstrained *NRPS22* tree (Fig. 7a). In the first tree, taxa from the Fujikuroi, Oxysporum and Nisikadoi complexes, and *F. beomiforme* were constrained to a clade that excluded FIESC 23 and FIESC 28; and in the other tree, FIESC 23, FIESC 25, *F. aywerte*, and Sambucinum-complex taxa were constrained to a clade that excluded Fujikuroi-complex taxa. The results of the SH-AU tests indicated that constrained trees were significantly worse (*p* < 0.05) than the corresponding unconstrained trees for 11 of the 17 putative HGT events, including the putative HGT of *NRPS22* from the Fujikuroi complex to FIESC (Table 3). Thus, the SH-AU tests provided additional support for 11 putative HGT events between FIESC and other *Fusarium* lineages.

### HGT – Analysis of synonymous site divergence

Estimates of the number of synonymous changes per synonymous site (d_S_) in coding regions of HK genes tend to be positively correlated with divergence of *Fusarium* species [50, 51]. Given this, recent HGT of a gene should result in low d_S_ values for the transferred gene relative to HK genes in the donor and recipient species, because d_S_ values for the transferred gene should reflect divergence levels since the transfer, whereas d_S_ values of HK genes should reflect divergence levels since the speciation event(s) that led to the donor and recipient. Based on this rationale, we used PAML to generate d_S_ values for each NRPS/PKS gene and for concatenated sequences of 30 HK genes (Additional file 1) for all possible pairwise combinations of taxa included in the species tree (Fig. 1). We then compared the d_S_ values for the NRPS/PKS genes and HK genes. For most comparisons, d_S_ values for NRPS/PKS genes were significantly higher than d_S_ values for HK genes (Fig. 7c, Additional file 8). This difference in d_S_ values occurred for almost all comparisons involving NRPS/PKS genes that were continuously or nearly continuously distributed and that yielded phylogenetic trees that were largely concordant with the species tree (Additional files 5 and 8). Thus, NRPS/PKS genes whose distribution and phylogenetic tree were consistent with vertical inheritance tended to have larger d_S_ values than HK genes. This suggests that in *Fusarium* vertically inherited NRPS/PKS genes tend to diverge more rapidly than HK genes.

Because of the tendency for larger d_S_ values for NRPS/PKS genes than HK genes, the ratio of the NRPS/PKS d_S_ value to HK d_S_ value (d_S_ ratio) for a given pairwise comparison of taxa was most often 1.5–4, and even higher for some comparisons (Fig. 7d, Additional file 8). Examination of d_S_ values for all pairwise combinations of taxa that had homologs of NRPS/PKS genes involved in the 17 putative HGT events revealed d_S_ ratios of less than 1 for taxa involved in 13 of the events (Table 3). For example, for comparisons involving *NRPS22*, most d_S_ ratios were 2–5, but d_S_ ratios were less than 1 for comparisons of FIESC 23 or FIESC 28 with members of the Fujikuroi, Nisikadoi and Oxysporum complexes (Additional file 8). The d_S_ ratios for comparisons of FIESC 23 or FIESC 28 to *F. nygamai* (Fujikuroi complex) were particularly low (0.27 and 0.30). This suggested that *NRPS22* homologs in FIESC23 and FIESC28 began diverging from homologs in the Fujikuroi, Nisikadoi and Oxysporum complexes after FIESC began diverging from these other complexes. Together, results of d_S_ analysis, manual tree comparisons, NOTUNG analysis, and SH-AU tests are consistent with HGT of *NRPS22* from the Fujikuroi complex (a close relative of *F. nygamai*) to a recent ancestor of FIESC 23 and FIESC 25. Putative HGT events for *NRPS4*, *PKS10* and *PKS23* were also consistent with results from all the phylogenetic analyses used in this study, whereas other putative HGT events were consistent with results from only one or a subset of analyses (Table 3).

### Evidence for hybrid polyketide-nonribosomal peptide biosynthetic gene cluster

We detected the genes *PKS42* and *NRPS34* in all *Equiseti*-clade genomes, except for the genome of FIESC 5, ITEM 11348. In addition, we did not detect the genes in any of the *Incarnatum*-clade genomes. Further examination of the *Equiseti*-clade genomes indicated that *PKS42* and *NRPS34* were located near one another in a region that spanned 62,536–64,747 bp and that included seven other genes (Additional file 9 A). The seven genes were predicted to encode proteins with functions consistent with SM biosynthesis; i.e., ABC transporter, cytochrome P450 monooxygenase, dioxygenase, reductase, and C_2_H_2_ Zn finger transcription factor. We also found *PKS42*, *NRPS34* and the other seven genes arranged in the same order in the genome sequences of *F. avenaceum* strain LH27 [52] and *F. aywerte* (Additional file 9A). The genes were also present, albeit in a different arrangement, in the genome sequence of *Colletotrichum simmondsii* (NCBI Accession JFBX00000000.1). The predicted functions of *PKS42*, *NRPS34*, and the other seven genes combined with their proximity to one another in multiple taxa suggests that they constitute a SM biosynthetic gene cluster that confers the ability to synthesize a hybrid polyketide-nonribosomal peptide. Examination of the cluster flanking region among members of the *Equiseti* clade revealed that some of the flanking genes are shared by all members of the clade, suggesting that cluster homologs are in similar genomic contexts (Additional file 9 B). A similar arrangement of the flanking genes also exists in other members of FIESC that lack the cluster, including FIESC 5 ITEM 11348.

### Trichothecene cluster organizations

**Fig. 8:**
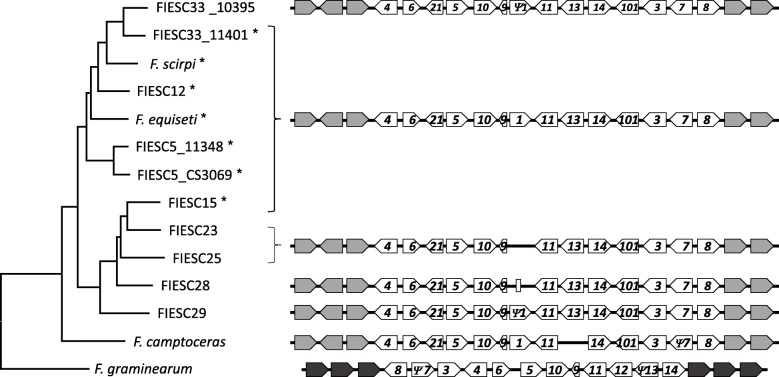
Left: FIESC species tree derived from ML tree in Fig. 1. Right: variation in trichothecene biosynthetic cluster homologs in FIESC. Arrows indicate genes and direction of transcription. White arrows labelled with numbers indicate known *TRI* genes. Gray arrows indicate genes flanking *TRI* cluster . *indicates that *TRI16* is present in the genome

The trichothecene biosynthetic gene (*TRI*) cluster was detected in all FIESC genomes examined in the current study. In seven of the FIESC genome sequences, *TRI* cluster orthologs were identical to the cluster ortholog previously reported in FIESC 12 (NRRL 13405) [27] with respect to gene content, gene order, direction of transcription, and genomic context (Fig. 8). Within the *Equiseti* clade all genomes examined have an intact and apparently functional *TRI1* gene except for FIESC 33 (ITEM 10395). The *TRI1* coding region in 10,395 has a single nucleotide deletion near the 5′ end of the coding region that introduces a frameshift and subsequently internal stop codons.

These stop codons should prevent translation of a full length Tri1 protein. Within the *Incarnatum* clade, FIESC 15 strain has an intact and apparently functional *TRI1*, while *TRI1* is absent in the FIESC 23 and FIESC 25 strains and it is pseudogenized in FIESC 28 and FIESC 29. In FIESC 28 there is only a 127 base fragment of *TRI1*, corresponding to bases 6–132 of the *TRI1* coding region, while FIESC 29 has a 122 base deletion near the 5′ end of the *TRI1* coding region.

In all fusaria that have been examined, *TRI16* is not located in the *TRI* cluster, and it is pseudogenized or absent in some species [27, 53, 54]. Analysis of FIESC genomes in the current study indicated that *TRI16* was present and in the same genomic context in FIESC 15 and in all members of the *Equiseti* clade examined, but that it was absent in *F. camptoceras* and all other members of the *Incarnatum* clade.

## Discussion

The results of this study revealed that collectively members of FIESC have the potential to produce diverse SMs. This was evident by the presence of 22 PKS and 18 NRPS genes in the FIESC genome sequences analyzed. Homologs from approximately 50% of the PKS and NRPS ortholog groups were present in all FIESC genomes examined (Fig. 2), which suggests that about half of the potential polyketide and non-ribosomal peptide-derived metabolites produced by FIESC species could be produced by most or even all members of FIESC. Some PKS and NRPS genes, as well as the associated gene clusters, were distributed discontinuously in a few species of the *Equiseti* or *Incarnatum* clade, or a few species in both clades. The fusarin biosynthetic gene cluster was among those with discontinuous distributions. As far as we are aware, fusarin production has never been reported in members of FIESC. Therefore, finding the fusarin cluster in three FIESC genomes attests to the value of whole genome sequence analysis to determine the genetic potential of fungi to produce metabolites.

Gene deletion and pseudogenization, here identified by alignment against orthologous SM reference sequences and altering the gene coding sequence, have likely contributed to the discontinuous distribution of some SM genes and gene clusters. For example, the distribution of the intact and degenerated *ZEA* cluster homologs in FIESC is consistent with the presence of the intact cluster in the common ancestor of FIESC followed by pseudogenization and deletion events in some FIESC species that resulted in the absence of the cluster, or presence of only a degenerated cluster, in multiple members of FIESC.

Our analysis showed the presence of a putative cluster, including the *PKS42* and *NRPS34* genes and seven other genes, distributed in all *Equiseti* clade species, except for the genome of FIESC 5 ITEM 11348, as well as in the genome of *F. avenaceum* (LH27), *F. aywerte*, and *C. simmondsii* (Additional file 9). The presence of the *PKS42*-*NRPS34* cluster in FIESC 5 (CS3069), and its absence in FIESC 5 ITEM 11348, indicates two possible evolutionary scenarios for the cluster in FIESC 5: 1) the cluster was present in the common ancestor of CS3069 and ITEM 11348, and was subsequently lost during divergence of ITEM 11348; or 2) the genomic region that includes the *PKS42*-*NRPS34* cluster exists as two alternative alleles, one allele with the cluster and another without it. There is evidence for the existence of similar scenarios with the fumonisin and ochratoxin biosynthetic gene clusters in species of *Aspergillus* [55, 56]. However, unlike the situation in *Aspergillus*, our efforts to find evidence for or against the two scenarios for the *PKS42*-*NRPS34* cluster in FIESC 5 yielded equivocal results. For example, we searched for remnants of *PKS42*-*NRPS34* cluster genes in the ITEM 11348 genome sequence as evidence for recent deletion of the cluster, but without any success. Analysis of the *PKS42*-*NRPS34* cluster region in the five haplotypes of FIESC 5 already described [14] could provide further insight into the evolutionary history of the cluster.

In this study, we used antiSMASH to estimate the percentage of genes in a genome that are involved in SM biosynthesis (Table 2). While such antiSMASH-based estimates are likely imperfect, overestimation or underestimation of SM genes are likely to be systematic due to criteria that antiSMASH uses to identify clusters. As a result, overestimates and underestimates are likely similar for genome sequence assemblies with similar properties. Thus, we attributed the low percentage of SM genes in the FIESC 5 CS3069 genome sequence to the high number of contigs in the genome sequence assembly for this strain compared to other FIESC members examined, including FIESC 5 ITEM 11348 (Table 2). The CS3069 genome sequence was assembled into 5111 contigs, that is 3.4–12.1 times as many contigs as the other FIESC strains examined in this study. If we exclude CS3069, estimates of SM genes percentage per genome is similar to other FIESC members, ranging from 3.3–4.3%. This similarity contrasts the finding that only about 50% of the observed PKS and NRPS genes were shared by all the genomes examined. Thus, the estimates of homologs of some clusters were present in all the FIESC strains examined, others clusters were present in subsets of species, sometimes only in members of either the *Equiseti* or *Incarnatum* clade, while other clusters were unique to one strain, such as *NRPS15* and *NRPS17*. The antiSMASH prediction of SM clusters is mainly based on PKS, NRPS and terpene synthase (TS) genes, therefore it was not surprising that the predicted gene clusters exhibited the same distribution patterns as PKS and NRPS genes.

Additionally, FIESC appears to have few unique SM gene clusters. The large majority of clusters that occur in FIESC genomes have been already reported in other fusaria. What is unique about FIESC, and most likely other *Fusarium* species complexes, is the combinations of clusters that are present within each lineage. For example, all members of the FSAMSC that have been examined have the aurofusarin, fusarubin and trichohecene clusters, but not the bikaverin cluster; all members of FFSC that have been examined have the bikaverin and fusarubin clusters but not the aurofusarin or trichothecene cluster; and all members of FIESC examined in the current study have the fusarubin and trichothecene clusters but not the aurofusarin or bikaverin cluster [8, 9, 30, 34, 57]. The trichothecene cluster was the only biosynthetic gene cluster for a major *Fusarium* mycotoxin (i.e., enniatins/beauvericin, fumonisins, trichothecenes and zearalenone) that occurred in all members of FIESC. In contrast, the enniatin/beauvericin and zearalenone clusters exhibited discontinuous distributions among the genomes (Fig. 2), as well as the fumonisin cluster was absent from all the genomes.

The evidence that all members of FIESC have trichothecene biosynthetic cluster is significant, given previous reports of variation in trichothecene production among members of FIESC [22, 24, 58] and because trichothecenes are among the mycotoxins of greatest concern to agricultural production and food/feed safety. Despite of the presence of the trichothecene cluster in all FIESC strains examined, a variation in gene content of the *TRI-*cluster homologs was observed. For example, *TRI1* was absent or pseudogenized in some members of the *Incarnatum* clade, while *TRI13* was absent and *TRI7* was pseudogenized in *F. camptoceras*. Such differences in gene content determine which trichothecene analogs are produced.

During trichothecene biosynthesis, the *TRI13*- and *TRI7*-encoded enzymes catalyze hydroxylation and *O*-acetylation of carbon atom 4 (C4)*,* respectively [59]. Although trichothecene production has not been reported in *F. camptoceras*, the absence of *TRI13* and pseudogenization of *TRI7* suggests that this species would produce trichothecene analogs that lack hydroxyl or acetyl substituent at C4. Within the *F. sambucinum* species complex (FSAMSC), there is a lineage of closely related species in which *TRI13* and *TRI7* are pseudogenized. As a result, these fusaria produce only trichothecene analogs that lack modifications at C4, including deoxynivalenol (DON), 3-acetyl DON (3ADON) and 15-acetyl DON (15ADON) [60, 61]. For example, in most strains of *F. graminearum*, *TRI7* and *TRI13* are present as pseudogenes and as a result these strains produce either 3ADON or 15ADON. However, most species within FSAMSC have functional *TRI13* and *TRI7* orthologs. The results of the current study indicate that a similar situation occurs also in FIESC. That is, with the exception of *F. camptoceras*, FIESC members have functional copies of *TRI7* and *TRI13* and, therefore, the genetic potential to produce trichothecenes with C4 modifications. Because most members of FIESC and FSAMSC have functional copies of *TRI7* and *TRI13*, we propose that the loss/pseudogenization of *TRI7* and *TRI13* in *F. camptoceras* occurred independently of the pseudogenization in FSAMSC. If this hypothesis is correct, it would constitute convergent evolution of the trichothecene biosynthetic pathway in FSAMSC and FIESC. Furthermore, the loss/pseudogenization of *TRI7* and *TRI13* in two lineages of *Fusarium* suggests that a lack of selection for production of trichothecenes with a C4 modification has occurred twice during the evolutionary history of trichothecene-producing fusaria.

In FIESC, as well as in all examined fusaria, *TRI16* is not located within the *TRI* core cluster, and it is pseudogenized or absent in some species [27, 53, 54]. The *TRI16*-encoded acyltransferase catalyzes esterification of a five-carbon metabolite (3-methylbutanoate) to a hydroxyl at C8, resulting in the formation of T-2 toxin and structurally related trichothecenes [52, 53]. Despite the presence of *TRI16* in some members of FIESC, multiple survey studies showed that members of FIESC do not produce T-2 toxin or other trichothecenes with a 3-methylbutanoate ester at C8 [22, 24, 58]. This raises the possibility that *TRI16* homologs in FIESC species are not expressed, not functional due to changes in its amino acid sequence, or that they have taken on a function in a different metabolic pathway. There is evidence that, during the evolutionary divergence of FIESC from other trichothecenes-producing lineages of *Fusarium*, *TRI1* was translocated into the *TRI* cluster from elsewhere in the genome [27]. The absence of *TRI1* in *TRI* cluster orthologs of some members of FIESC suggests that following this translocation, *TRI1* was lost in some FIESC lineages (Fig. 8).

In the last decade, genome sequence analyses have revealed the existence of frequent variation in the content of secondary metabolite biosynthetic gene clusters among filamentous fungi. Multiple studies suggest that vertical inheritance, gene loss, and HGT are major contributors to this variation [50, 62, 63]. However, the frequency with which each process has contributed to the variation remains unclear. To assess how often vertical inheritance, gene loss, and HGT have contributed to the presence and absence of clusters among FIESC members, we did a series of phylogenetic analyses. In this assessment, we focused on NRPS and PKS (NRPS/PKS) genes, because they are present in a large proportion of SM clusters, and they can contain substantial phylogenetic signal as a result of their large size. To begin the assessment, we used BLAST analysis with NCBI’s GenBank database and a local database to examine the occurrence of homologs of each FIESC NRPS/PKS gene in the fusaria included in the species tree (Fig. 1). This analysis revealed that some of the NRPS/PKS genes were present in all or almost all fusaria examined, others were discontinuously distributed across a wide range of species, and still others were more narrowly distributed, occurring exclusively or almost exclusively in FIESC (Fig. 2).

We presumed that vertical inheritance was the process that most frequently introduced NRPS/PKS genes into and subsequently distributed them within FIESC. This assumption was supported by continuous or wide distribution of *NRPS1-NRPS4*, *NRPS6*, *NRPS10*-*NRPS14*, *NRPS16*, *NRPS19*, *PKS3*, *PKS5*, *PKS7*, and *PKS8* in FIESC and fusaria most closely related to FIESC (i.e., the Sambucinum complex and *F. aywerte*) (Fig. 2, Additional file 5). The results of our study suggest that gene loss and HGT have contributed to the diversity of SM clusters in FIESC. In particular, gene loss has contributed to distribution of 21 clusters. This is likely an overestimation of the true number of losses, because in the absence of polytomy in the species tree, which was the case for the species tree used in this study, NOTUNG does not take into account that topological differences between a gene tree and the species tree can result from sorting of ancestral alleles. Also, NOTUNG suggested that multiple distribution patters were affected by gene duplication, although the hypothesis is not consistent with other analyses.

d_S_ values for *NRPS16*, *NRPS19*, *NRPS34*, *PKS42*, *PKS43* and *PKS69* reveal a likely limitation in the use of dS values for assessing putative HGT events, because almost all d_S_ ratios for comparisons involving these genes were less than 1. The cause of these low d_S_ ratios is not clear. One possibility is rampant HGT of the genes. However, in ML trees inferred from these genes, most internal branches were short, and most terminal branches were long (Additional file 5). Furthermore, most branches in these trees had poor bootstrap support (< 70). The cause(s) of these branching characteristics is unclear. Assessment of homoplasy using consistency and retention indices [64] suggested that the level of homoplasy in *NRPS16*, *NRPS19*, *NRPS34*, *PKS42*, *PKS43*, and *PKS69* sequences was within the range for sequences of other NRPS/PKS genes. Thus the cause(s) of the low d_S_ ratios for *NRPS16*, *NRPS19*, *NRPS34*, *PKS42*, *PKS43*, and *PKS69* is not clear.

Low d_S_ ratios also resulted from some pairwise comparisons that involved two strains of the same species (e.g., *F. oxysporum* strains Fol4287 and FOSC3a) or closely related species (e.g., *F. langsethiae* and *F. sporotrichioides*; species within the *Incarnatum* or *Equiseti* clades of FIESC). However, in some other comparisons involving strains of the same species or closely related species, d_S_ ratios were high. d_S_ values for both HK and NRPS/PKS genes in comparisons within species or between closely related species tended to be low (i.e., less than 0.1), and as a result small differences in d_S_ values for HK and NRPS genes could lead to very low or very high d_S_ ratios.

Overall, all evolutionary approaches hereby considered, showed that HGT has contributed to distribution of four clusters, such as the known fusarin and enniatin/beauvericin biosynthetic gene clusters and the unknown clusters with *PKS23* and *NRPS4* genes, respectively. These data provide evidence that a complex interplay of evolutionary processes contributes to variation in secondary metabolite cluster content in fungi and, therefore, to variation in their ability to produce the corresponding metabolites.

## Conclusion

The results of this study suggest that: 1) collectively, the FIESC strains examined in this study have the genetic potential to produce 22 structurally distinct PKSs and 18 NRPSs; 2) vertical inheritance has contributed to the distribution of almost all FIESC clusters; 3) HGT has contributed to distribution of four clusters and likely to other nine additional clusters, although the results from all analyses were not consistent.

Environmental factors that affect the maintenance of SM clusters in a recipient of HGT are poorly understood, but they could include soil conditions, competitors, hosts, and/or climate. However, which ones among these factors affected maintenance of SM clusters horizontally transferred to members of FIESC remains to be determined.

In conclusion, all those data can led to understanding which members of FIESC can produce which mycotoxins, concretely impacting growers response to fungal surveys in their fields, and supporting regulators to develop accurate assessments of the risks that members of FIESC pose to the food and feed supply.

## Methods

### Fungal strains

The FIESC strains examined in this study are listed in Table 1. Nine strains were obtained from the Agri-Food Toxigenic Fungi Culture Collection (ITEM; http://server.ispa.cnr.it/ITEM/Collection) at the Institute of Science of Food production (ISPA; Bari Italy); two strains were obtained from the U.S. Department of Agriculture (USDA), Agricultural Research Service (ARS) Culture Collection (NRRL; Peoria IL, USA, https://nrrl.ncaur.usda.gov/), and one strain was obtained from the Fusarium Research Center (FRC, Pennsylvania State University, State College PA, USA). Recently, Villani et al. [22] characterized a phylogenetically distinct group of FIESC isolates and designated them FIESC 31. However, prior to this publication, the designation FIESC 31 had already been applied to a different phylogenetic species [26]. In the current study, therefore, we redesignated FIESC 31 sensu Villani et al. [22] as FIESC 33, and we use the designation FIESC 31 as described by Short et al. [26]. We compared these genomes to the publicly available genome sequence of FIESC 5 strain CS3069 (National Center for Biotechnology Information (NCBI) accession number: QGEC00000000) [28]. In addition to the strains listed in Table 1, partial genomic sequences for other *Fusarium* species were obtained from GenBank for inferring species phylogeny, as well as PKS and NRPS analyses.

### Genome sequencing and assembly

Genome sequences for FIESC strains were generated using an Illumina MiSeq sequencer. The exception to this was the sequence for *F. scirpi* NRRL 66328, which was generated using a Life Technologies Ion Torrent PGM™ sequencer. Both sequencers were located at the Mycotoxin Prevention and Applied Mycology Research Unit at USDA ARS, Peoria IL. High-quality genomic DNA was prepared from each species grown in GYP (2% glucose, 1% peptone and 0.3% yeast extract) liquid cultures using Zymo DNA Clean & ConcentratorTM-5 kit as described by the manufacture (Zymo Research, Orange, California). One ng of DNA was used to generate paired-end DNA libraries for the MiSeq using the Nextera XT DNA Library Preparation Kit (Nextera XT DNA Library Preparation Experienced User Card 15,031,943 D). DNA (1 μg) from *F. scirpi* was used to generate the DNA library for the Ion Torrent using the New England Biolabs NEBnext Fast DNA Library prep set. Sequence reads were processed and assembled with CLC Genomics Workbench version 8.0 (CLC bio-Qiagen, Aarhus, Denmark) using default parameters, except that the minimum contig length was set to 500 bp. The resulting unannotated genome sequences were deposited at DDBJ/ENA/GenBank under the accessions shown in Table 1. Gene predictions for each genome were done with the program Augustus [65] using *F. graminearum* as the training species. When we identified predicted gene models that appeared to have errors (e.g., incorrect intron splicing), we subjected the corresponding genomic sequence to FGENESH [66] analysis and/or manual annotation by aligning the corresponding genomic DNA to predicted genes from other fusaria using the program MEGA 7.0 [67]

### Species phylogeny

A species phylogeny was inferred using coding region sequences of 30 HK genes (Additional file 1) from the 13 members of FIESC and 24 other *Fusarium* species that spanned the phylogenetic breadth of the genus and for which genome sequence data were publicly available (Additional file 1). FIESC HK gene sequences were mined by BLASTn analysis of individual genome sequence databases in CLC Genomic Workbench, while sequences from other fusaria were downloaded from GenBank. Most of the HK genes have been used previously to assess phylogenetic relationships of *Fusarium* species [3, 15, 29, 35] and are involved in metabolic processes essential for growth and development. However, some of the genes (e.g., *CAR1*) are not essential, but occur widely as single-copy genes in *Fusarium*. All DNA sequences of coding regions were inspected, and when necessary were manually annotated to correct large gaps resulting from incorrect prediction of introns and translational start and stop sites.

Housekeeping gene sequences were aligned using Muscle as implemented in MEGA7 [67], and the resulting alignments were used to infer ML trees for each gene using IQ-Tree (version 1.6.7) with 1000 bootstrap replicates [68]. The species tree was inferred from the 30 HK gene ML trees using the MRE method as implemented in RAxML (v.8.2.10) [69] Branch support for the MRE consensus tree was obtained by the internode certainty method as implemented in RAxML [70, 71]. For comparison purposes, a species tree was also inferred using the ML method implemented in IQ-Tree (version 1.6.7) from the concatenated coding region sequences of the 30 housekeeping genes. Branch support for the ML tree was determined by bootstrap analysis with 1000 replicates [68]. Trees with support values were displayed using Dendroscope software V3.5.9 [72] and MEGA7 [67].

### Identification of putative secondary metabolite gene clusters

To investigate the genetic potential of members of FIESC to produce mycotoxin and other SMs, we subjected the FIESC genome sequences to BLAST and antiSMASH [73, 74] analyses. We then assessed the results from both analyses to draw conclusions about the presence and absence of SM gene clusters. In the BLAST analysis, we employed both BLASTn and BLASTx methods in CLC Genomics Workbench. BLAST query sequences consisted of representative genes from previously described PKS and NRPS ortholog groups/clades [29, 30]. We also queried the databases with sequences of 166 genes previously reported to be required for biosynthesis of 27 families of *Fusarium* SMs (Additional file 3). Proteins encoded by these latter genes included terpene synthases, oxidoreductases, acyltrasferases, transporters, and transcription factors in addition to PKSs and NRPSs. The retrieved sequences were then aligned with sequences of genes or gene clusters publically available for other annotated *Fusarium* species, known to be involved or considered potentially involved in mycotoxins and related SMs production. The prediction of novel putative secondary metabolite genes was done by analysis of 13 FIESC genomes using Augustus [65] and FGENESH [66] selecting *Fusarium* species as references. The antiSMASH analysis was also performed to obtain information about partition involved in SM biosynthesis in each genome. We used this number and the total number of genes per genome predicted by Augustus program to estimate the percentage of SM biosynthetic genes per FIESC genome.

Conservation of synteny of some clusters between FIESC genomes and other fungal species was examined by BlastP analysis in GenBank and confirmed by the alignment of potential homologous clusters and flanking regions using Sequencher (version 5.2.4; Gene Codes Corp.)

### Phylogenetic analyses of NRPS and PKS genes

We used full-length, predicted amino acid sequences to assess diversity and phylogenetic relationships of FIESC PKSs. The PKS analysis included sequences of 135 PKS enzymes identified in the 12 FIESC genome sequences generated during the course of this study, 11 PKSs from CS3069, and 216 PKSs from other fusaria previously identified [29] or identified as top hits (e-value cutoff 1e^− 05^) in BLAST searches at NCBI. Sequences were aligned using Mafft [75] and an ML tree was generated using IQ-TREE [68] with the fatty acid synthase (FAS) from *Gallus gallus* serving as the outgroup. To gain further insight into diversity of NRPSs in FIESC, we assessed the phylogenetic relationships of A domains of FIESC NRPSs using the same phylogenomic approach that Bushley and Turgeon [46] used to assess NRPS diversity in a range of fungal genera. In our analysis, we used BLAST to identify NRPS homologs that were collectively encoded by the 13 FIESC genomes included in this study. We then retrieved and aligned using Mafft all the adenylation domain (A domains) from these and other *Fusarium* NRPSs [30], and subjected the alignment to ML analysis, using IQ-TREE with the ultrafast bootstrap with 10,000 bootstrap replicates [76].

### Evolutionary forces acting upon distribution of SM gene clusters within FIESC

We did a series of phylogenetic analyses to assess evolutionary processes that have likely contributed to the introduction of SM gene clusters into FIESC and subsequent distribution of the clusters among FIESC members. The phylogenetic analyses consisted of: 1) assessment of the occurrence of the genes in FIESC and other *Fusarium* lineages; 2) manual comparison of single NRSP/PKS gene trees to the species tree; 3) constraint analyses using the Shimodaira-Hasegawa and Approximately Unbiased (SH-AU) tests to assess statistical support for branches indicative of HGT [48, 49]; 4) reconciliation analysis using NOTUNG v2.9 with default cost [77, 78]; and 5) genetic divergence analysis using estimates of synonymous substitutions per synonymous site (dS), using the ML method in CodeML program as implemented in PAML [79] (with the options seqtype = 1, runmode = − 2, and CodonFreq = 0 in the codeml.ctl files).

## Additional files

Additional file 1: List of 30 HK genes and strains used to infer species trees in this study. (DOCX 16 kb)

Additional file 2: *Fusarium* species tree inferred by maximum likelihood analysis of concatenated sequences of 30 housekeeping genes (see Additional file 1). Each gene sequence was aligned separately using MUSCLE as implemented in MEGA7. The resulting alignments were then concatenated using SequenceMatrix, and then subjected to maximum likelihood analysis as implemented in IQ-Tree (version 1.6.7). (PPTX 90 kb)

Additional file 3: Results of BLASTn analysis to assess presence and absence of genes or gene clusters for which the secondary metabolic products are known. (XLSX 25 kb)

Additional file 4: Trees inferred by maximum likelihood analysis of alignment of predicted amino acid sequences of the four major groups of PKS genes retrieved from FIESC genome sequences examined in this study. Supplementary Figs. **A**. – **D**. correspond to the major groups of PKS genes previously described in *Fusarium* and other fungi [29]. The major groups are the non-reducing PKSs (NR-PKS) (**A**.) and the three subgroups of reducing PKSs: R-PKS I (**B**.), R-PKS II (**C**.) and R-PKS III (**D**.). For each major group, phylogenetically distinct clades (or homolog groups) are labeled with clade numbers (C3, C5, C8, etc.) that were previously described in analysis of other fusaria (Brown and Proctor 2016). Homologs from other fusaria were included in current study and are designated using the same abbreviations used by Brown and Proctor [29]. Numbers near branches are bootstrap values based on 1000 pseudoreplicates. Bootstrap values below 70% are not shown. (PPTX 154 kb)

Additional file 5: Trees inferred by ML analysis of individual NRPS and PKS genes retrieved from FIESC genome sequences examined in this study. All the homologous genes retrieved from NCBI and were included in the analysis. Numbers near branches are bootstrap values based on 1000 pseudoreplicates. Bootstrap values below 70% are not shown. (PPTX 671 kb)

Additional file 6: Reconciled NRPS and PKS trees with duplication, losses and HGT events as obtained from NOTUNG. Duplications are indicated with red D, transfers are indicated by yellow T’s, and losses in grey. (PPTX 303 kb)

Additional file 7: Results of Shimodaira-Hasegawa (SH) and Approximately Unbiassed (AU) tests used to assess branches that suggest horizontal gene transfer of NRPS and PKS genes between FIESC and other lineages of *Fusarium*. (DOCX 21 kb)

Additional file 8: Results of the d_S_ analyses. (XLSX 1391 kb)

Additional file 9: **A**. Analysis of homologs of the putative PKS42-NRPS34 cluster. On the left, tree inferred by maximum likelihood analysis of concatenated sequences of the nine genes in the PKS43-NRPS34 cluster. Numbers near branches are bootstrap values based on 1000 pseudoreplicates. Right, gene organization in PKS42-NRPS34 cluster homologs. The vertical dashed lines between genes in the F. aywerte homolog indicate that the genes are one different contigs. The genes on either side of a vertical lines are at the ends of contigs and, therefore, could be adjacent to one another in the genome. **B**. Analysis of genes flanking the putative *PKS42*-*NRPS34* cluster and their homologs in selected members of FIESC that lack the cluster. The tree to the left was inferred by maximum likelihood analysis of flanking gene *F2*. Numbers near branches are bootstrap values based on 1000 pseudoreplicates. Bootstrap values below 70% are not shown. Genes and their direction of transcription are represented by arrows. Gray arrows represent genes in the *PKS42*-*NRPS34* cluster, white arrows represent cluster flanking genes, and the yellow arrow represents a gene that was unique to the region in *F. camptoceras*. (PPTX 154 kb)

## Abbreviations

A domain: Adenylation domain
HGT: Horizontal Gene Transfer
HK: Housekeeping
ML: Maximum Likelihood
MRE: Majority Rule Extended tree
NRPS: non-ribosomal peptide synthetase
PKS: polyketide synthase
