# Supplementary material for: Variation in secondary metabolite production potential in the Fusarium incarnatum-equiseti species complex revealed by comparative analysis of 13 genomes
Source: BMC Genomics. 2019 Apr 24;20:314. doi: 10.1186/s12864-019-5567-7 (PMC6480918; doi:10.1186/s12864-019-5567-7)
Supplement: Supplementary file 1 — List of 30 HK genes and strains used to infer species trees in this study. (DOCX 16 kb) [file 12864_2019_5567_MOESM1_ESM.docx]

**List of 30 HK genes**

| **Gene** | ***F. graminearum* Gene Model** | **Predicted Protein Product** |
| --- | --- | --- |
| *CAL1* | FGSG_01891 | Calmodulin |
| *CAR1* | FGSG_03066 | Carotenoid Biosynthetic Gene |
| *CPR1* | FGSG_09786 | Cytochrome P450 Reductase |
| *DPA1* | FGSG_05421 | DNA Polymerase Alpha Subunit |
| *DPE1* | FGSG_12863 | DNA Polymerase Epsilon Subunit |
| *ERG1* | FGSG_06215 | Ergosterol Monooxygenase/Oxidase |
| *FAS1* | FGSG_05321 | Fatty Acid Synthase Alpha Subunit |
| *FAS2* | FGSG_05322 | Fatty Acid Synthase Beta Subunit |
| *FLB1* | FGSG_03597 | Regulatory Gene |
| *FPS1* | FGSG_06784 | Farnesyl Pyrophosphate Synthase |
| *GPD1* | FGSG_06257 | Glyceraldehyde Phosphate Dehydrogenase |
| *HGR1* | FGSG_09197 | Hydroxy Methyl Glutaryl Co-A Reductase |
| *HIS3* | FGSG_04290 | Histone H3 |
| *LAC1-SPH1* | FGSG_05525 | Longevity Assurance Factor/Sphinganine N Acyl Transferase Subunit 1 |
| *LAC1-SPH2* | FGSG_03851 | Longevity Assurance Factor/Sphinganine N Acyl Transferase Subunit 2 |
| *LAE1* | FGSG_00657 | Global Regulatory Gene/Chromatin Methyl Transferase |
| *LCB1* | FGSG_07945 | Sphinganine Palmitoyl Transferase Subunit 1 |
| *LCB2* | FGSG_04102 | Sphinganine Palmitoyl Transferase Subunit 2 |
| *MCM7* | FGSG_07105 | DNA Replication Licensing Factor |
| *PGK1* | FGSG_03992 | Phosphoglycerate Kinase |
| *PPT1* | FGSG_08779 | 4'-Phosphopantetheinyl Transferase |
| *RPB1* | FGSG_00916 | RNA Polymerase Largest Subunit |
| *RPB2* | FGSG_02659 | RNA Polymerase 2nd Largest Subunit |
| *TEF1* | FGSG_08811 | Translation Elongation Factor 1-alpha |
| *TOP1* | FGSG_06874 | Topoisomerase |
| *TPS1* | FGSG_06051 | Trehalose Phosphate Synthase |
| *TSR1* | FGSG_04403 | Ribosomal biogenesis protein |
| *TUB1* | FGSG_00639 | Tubulin alpha subunit |
| *TUB2* | FGSG_09530 | Tubulin beta subunit |
| *URA7* | FGSG_07897 | Ammonium Ligase (required for uracil biosynthesis) |

**List of strains used in the study**

| **Nº** | ***Fusarium* species** | **strain** |
| --- | --- | --- |
| 1 | *F. acuminatum* | CS5907 |
| 2 | *F. avenaceum* | Fa05001 |
| 3 | *F. aywerte* | NRRL 25410 |
| 4 | *F. beomiforme* | NRRL 25174 |
| 5 | *F. camptoceras* | NRRL 13381 |
| 6 | *F. circinatum* | NRRL 25331 |
| 7 | *F. culmorum* | UK99 |
| 8 | *F. fujikuroi* | IMI 58289 |
| 9 | *F. gaditjirrii* | NRRL 45417 |
| 10 | *F. graminearum* | PH-1 |
| 11 | *F. langsethiae* | Fl201059 |
| 12 | *F. longipes* | NRRL 20695 |
| 13 | *F. miscanthi* | NRRL 26231 |
| 14 | *F. nygamai* | NRRL 66327 |
| 15 | *F. oxysporum* | FOSC 3a (FOSC3a) |
| 16 | *F. oxysporum* | NRRL 34936 (Fol4287) |
| 17 | *F. poae* | 2516 |
| 18 | *F. proliferatum* | NRRL 62905 |
| 19 | *F. pseudograminearum* | CS3096 |
| 20 | *F. scirpi* | NRRL 66328 |
| 21 | *F. solani* | 77-13-4 |
| 22 | *F. sporotrichioides* | NRRL 3299 |
| 23 | *F. temperatum* | CMWF389 |
| 24 | *F. torreyae* | NRRL 54149 |
| 25 | *F. udum* | NRRL 25194 |
| 26 | *F. venenatum* | A3/5 |
| 27 | *F. verticillioides* | 7600 |
| 28 | FIESC5 | ITEM 11348 |
| 29 | FIESC5 | CS3069 |
| 30 | FIESC12 | ITEM 11294 |
| 31 | FIESC14 (*F. equiseti*) | ITEM 11363 |
| 32 | FIESC15 | NRRL 31160 |
| 33 | FIESC23 | ITEM 7155 |
| 34 | FIESC25 | ITEM 6748 |
| 35 | FIESC28 | ITEM 1616 |
| 36 | FIESC29 | ITEM 10392 |
| 37 | FIESC33 | ITEM 10395 |
| 38 | FIESC33 | ITEM 11401 |
