# Supplementary material for: Variation in secondary metabolite production potential in the Fusarium incarnatum-equiseti species complex revealed by comparative analysis of 13 genomes
Source: BMC Genomics. 2019 Apr 24;20:314. doi: 10.1186/s12864-019-5567-7 (PMC6480918; doi:10.1186/s12864-019-5567-7)
Supplement: Supplementary file 2 — Fusarium species tree inferred by maximum likelihood analysis of concatenated sequences of 30 housekeeping genes (see Additional file 1). Each gene sequence was aligned separately using MUSCLE as implemented in MEGA7. The resulting alignments were then concatenated using SequenceMatrix, and then subjected to maximum likelihood analysis as implemented in IQ-Tree (version 1.6.7). (PPTX 90 kb) [file 12864_2019_5567_MOESM2_ESM.pptx]

## Slide 1
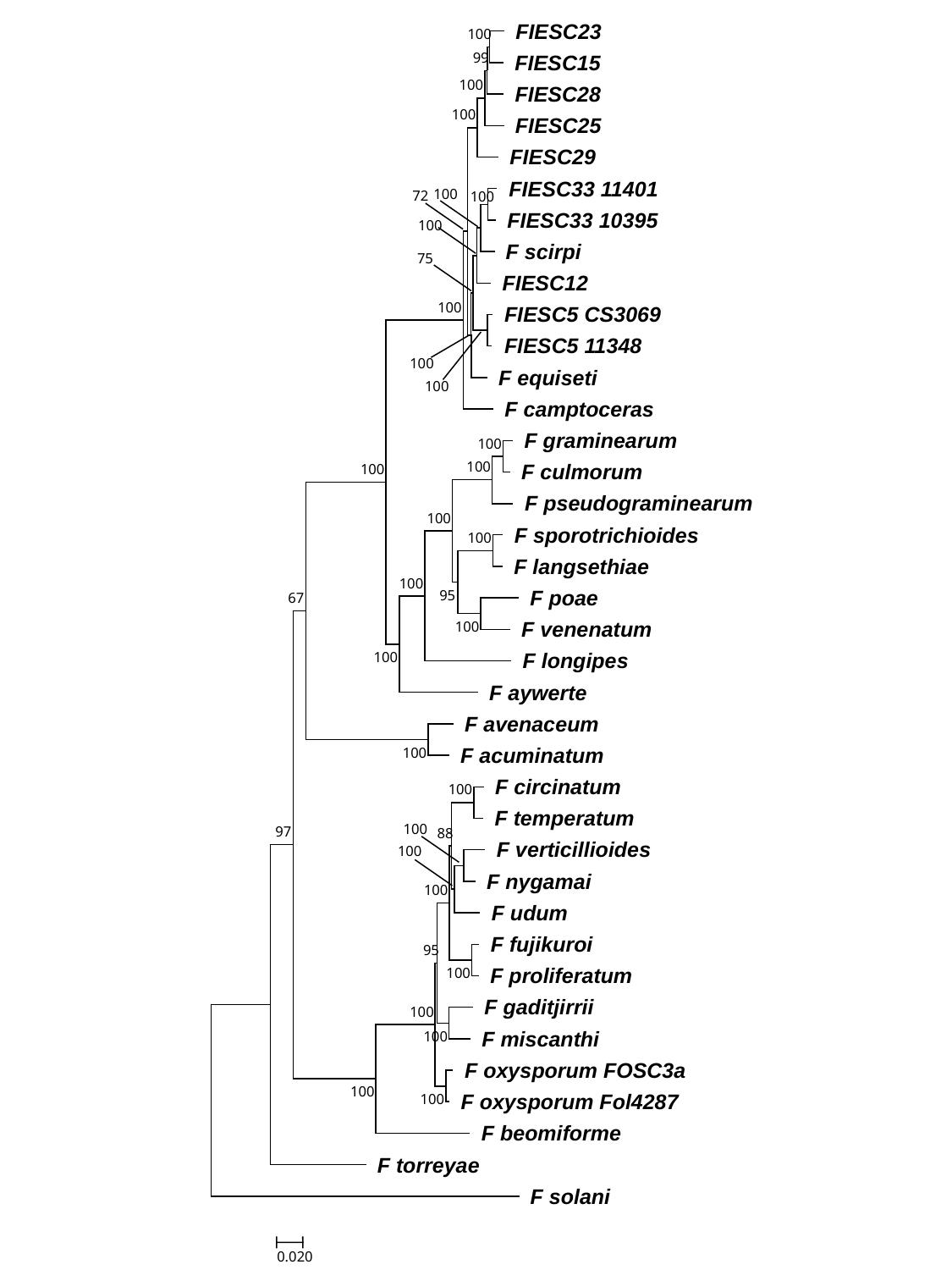

FIESC23
100
99
 FIESC15
100
 FIESC28
100
 FIESC25
 FIESC29
 FIESC33 11401
100
72
100
 FIESC33 10395
100
 F scirpi
75
 FIESC12
100
 FIESC5 CS3069
 FIESC5 11348
100
 F equiseti
100
 F camptoceras
 F graminearum
100
100
 F culmorum
100
 F pseudograminearum
100
 F sporotrichioides
100
 F langsethiae
100
 F poae
95
67
 F venenatum
100
 F longipes
100
 F aywerte
 F avenaceum
 F acuminatum
100
 F circinatum
100
 F temperatum
100
97
88
 F verticillioides
100
 F nygamai
100
 F udum
 F fujikuroi
95
 F proliferatum
100
 F gaditjirrii
100
 F miscanthi
100
 F oxysporum FOSC3a
100
 F oxysporum Fol4287
100
 F beomiforme
 F torreyae
 F solani
0.020
