# Supplementary material for: Variation in secondary metabolite production potential in the Fusarium incarnatum-equiseti species complex revealed by comparative analysis of 13 genomes
Source: BMC Genomics. 2019 Apr 24;20:314. doi: 10.1186/s12864-019-5567-7 (PMC6480918; doi:10.1186/s12864-019-5567-7)
Supplement: Supplementary file 4 — Trees inferred by maximum likelihood analysis of alignment of predicted amino acid sequences of the four major groups of PKS genes retrieved from FIESC genome sequences examined in this study. Supplementary Figs. A. – D. correspond to the major groups of PKS genes previously described in Fusarium and other fungi [29]. The major groups are the non-reducing PKSs (NR-PKS) (A.) and the three subgroups of reducing PKSs: R-PKS I (B.), R-PKS II (C.) and R-PKS III (D.). For each major group, phylogenetically distinct clades (or homolog groups) are labeled with clade numbers (C3, C5, C8, etc.) that were previously described in analysis of other fusaria (Brown and Proctor 2016). Homologs from other fusaria were included in current study and are designated using the same abbreviations used by Brown and Proctor [29]. Numbers near branches are bootstrap values based on 1000 pseudoreplicates. Bootstrap values below 70% are not shown. (PPTX 154 kb) [file 12864_2019_5567_MOESM4_ESM.pptx]

## Slide 1
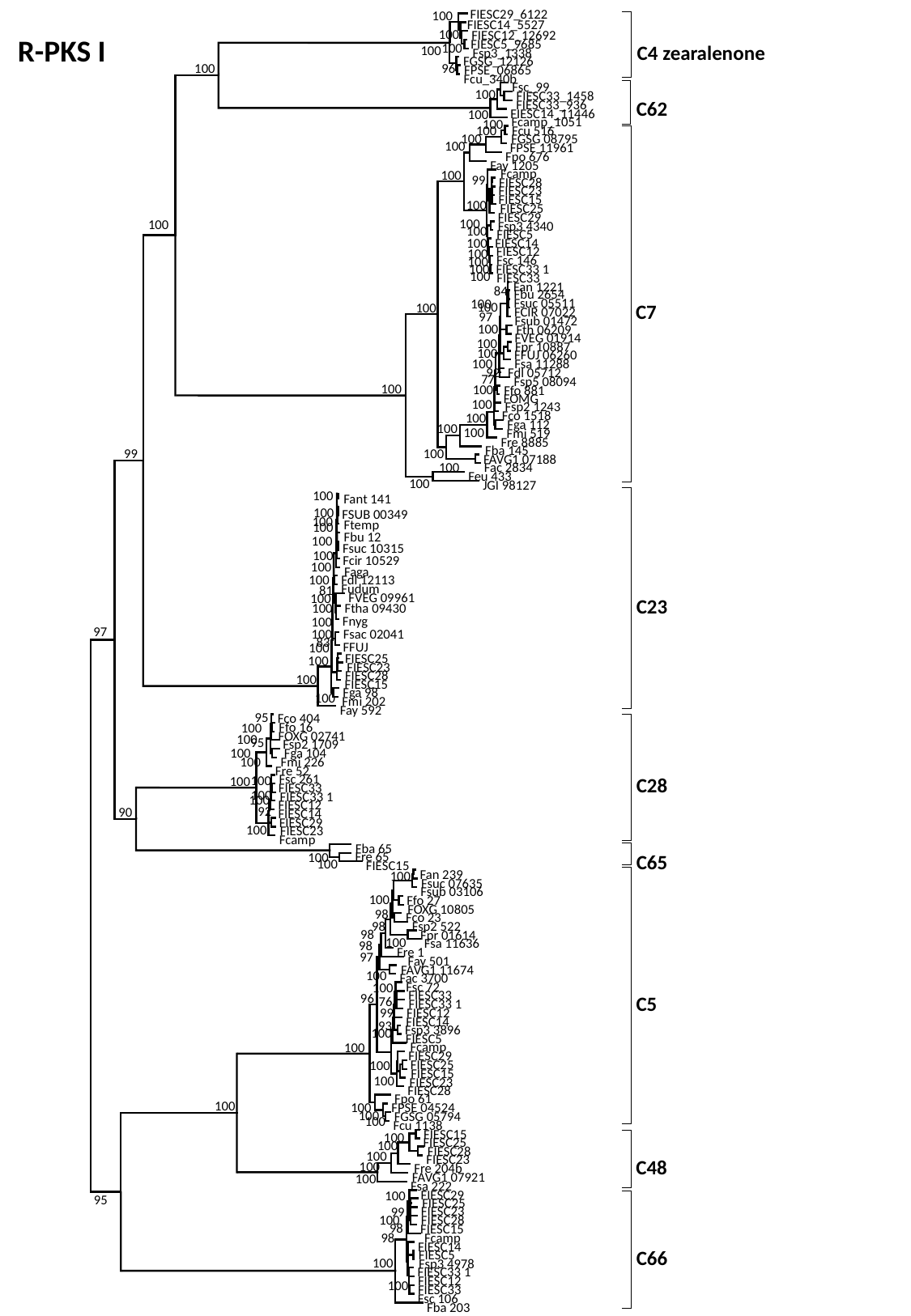

FIESC29_6122
100
FIESC14_5527
100
R-PKS I
 FIESC12_12692
 FIESC5_9685
100
C4 zearalenone
100
 Fsp3_1338
 FGSG_12126
96
100
 FPSE_06865
 Fcu_340b
 Fsc_99
100
 FIESC33_1458
C62
 FIESC33_936
 FIESC14_11446
100
 Fcamp_1051
100
 Fcu 516
100
100
 FGSG 08795
100
 FPSE 11961
 Fpo 676
 Fay 1205
 Fcamp
100
99
 FIESC28
 FIESC23
 FIESC15
100
 FIESC25
 FIESC29
100
100
 Fsp3 4340
100
 FIESC5
 FIESC14
100
 FIESC12
100
 Fsc 146
100
 FIESC33 1
100
100
 FIESC33
 Fan 1221
84
 Fbu 2654
 Fsuc 05511
100
100
C7
100
 FCIR 07022
97
 Fsub 01472
100
 Fth 06209
 FVEG 01914
100
 Fpr 10887
100
 FFUJ 06260
100
 Fsa 11288
92
 Fdl 05712
77
 Fsp5 08094
100
100
 Ffo 881
 FOMG
100
 Fsp2 1243
 Fco 1518
100
 Fga 112
100
100
 Fmi 519
 Fre 8885
 Fba 145
100
99
 FAVG1 07188
100
 Fac 2834
 Feu 433
100
 JGI 98127
100
 Fant 141
100
 FSUB 00349
100
 Ftemp
100
 Fbu 12
100
 Fsuc 10315
100
 Fcir 10529
100
 Faga
100
 Fdl 12113
 Fudum
81
 FVEG 09961
100
C23
100
 Ftha 09430
 Fnyg
100
97
 Fsac 02041
100
83
 FFUJ
100
 FIESC25
100
 FIESC23
 FIESC28
100
 FIESC15
 Fga 98
100
 Fmi 202
 Fay 592
95
 Fco 404
 Ffo 16
100
 FOXG 02741
100
95
 Fsp2 1709
100
 Fga 104
100
 Fmi 226
 Fre 52
 Fsc 261
100
C28
100
 FIESC33
100
 FIESC33 1
100
 FIESC12
92
90
 FIESC14
 FIESC29
100
 FIESC23
 Fcamp
 Fba 65
 Fre 65
100
C65
100
 FIESC15
 Fan 239
100
 Fsuc 07635
 Fsub 03106
100
 Ffo 27
 FOXG 10805
98
 Fco 23
 Fsp2 522
98
98
 Fpr 01614
100
 Fsa 11636
98
 Fre 1
97
 Fay 501
 FAVG1 11674
100
 Fac 3700
 Fsc 72
100
 FIESC33
96
C5
76
 FIESC33 1
99
 FIESC12
 FIESC14
93
 Fsp3 3896
100
 FIESC5
 Fcamp
100
 FIESC29
 FIESC25
100
 FIESC15
100
 FIESC23
 FIESC28
 Fpo 61
100
100
 FPSE 04524
100
 FGSG 05794
100
 Fcu 1138
 FIESC15
100
 FIESC25
100
 FIESC28
100
 FIESC23
C48
100
 Fre 204b
 FAVG1 07921
100
 Fsa 222
 FIESC29
100
95
 FIESC25
 FIESC23
99
100
 FIESC28
98
 FIESC15
 Fcamp
98
 FIESC14
C66
 FIESC5
100
 Fsp3 4978
 FIESC33 1
 FIESC12
100
 FIESC33
 Fsc 106
 Fba 203

## Slide 2
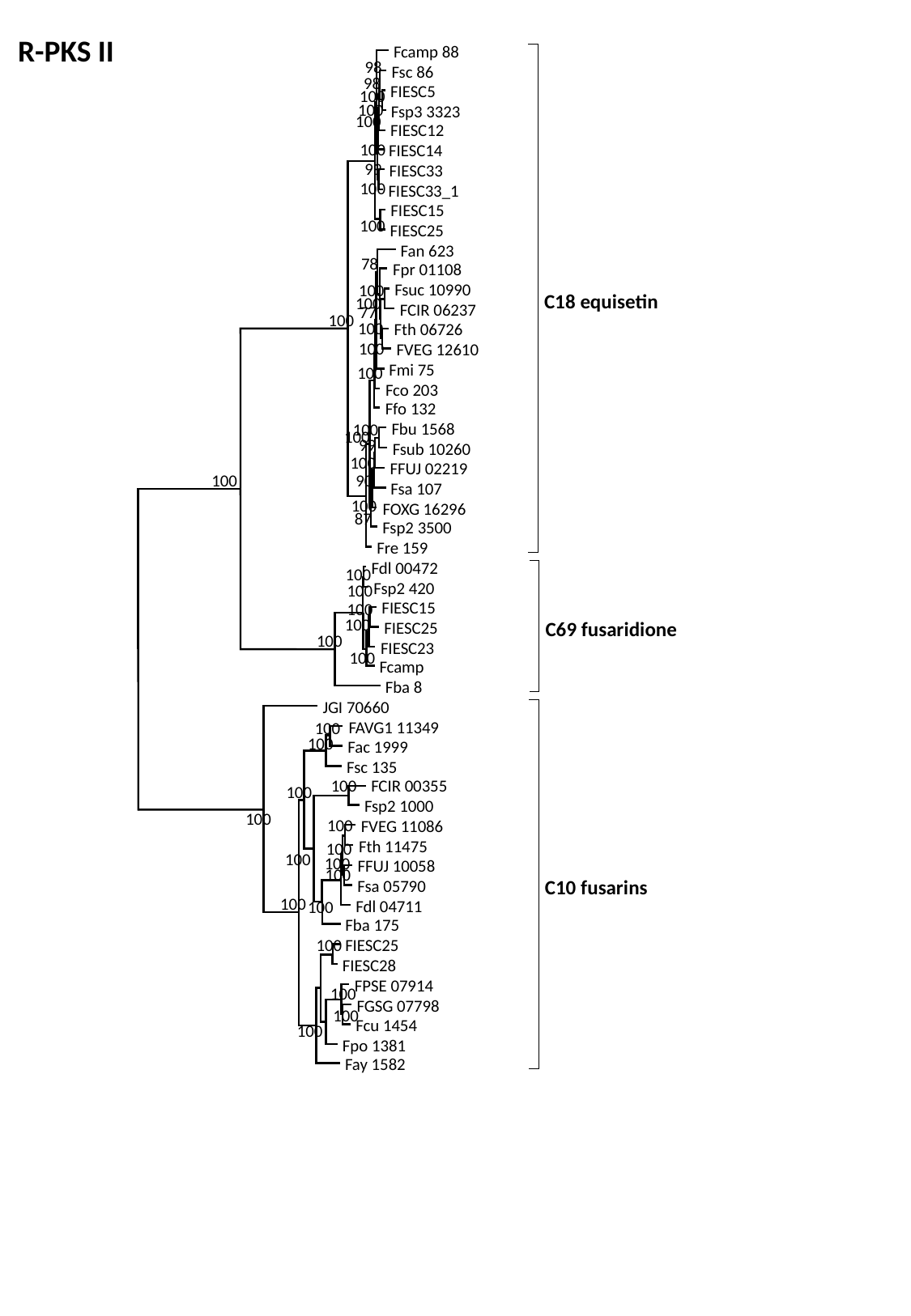

R-PKS II
 Fcamp 88
98
 Fsc 86
98
 FIESC5
100
100
 Fsp3 3323
100
 FIESC12
100
 FIESC14
99
 FIESC33
100
 FIESC33_1
 FIESC15
100
 FIESC25
 Fan 623
78
 Fpr 01108
 Fsuc 10990
100
C18 equisetin
100
 FCIR 06237
77
100
100
 Fth 06726
100
 FVEG 12610
 Fmi 75
100
 Fco 203
 Ffo 132
 Fbu 1568
100
100
99
 Fsub 10260
100
 FFUJ 02219
100
90
 Fsa 107
100
 FOXG 16296
87
 Fsp2 3500
 Fre 159
 Fdl 00472
100
 Fsp2 420
100
 FIESC15
100
100
C69 fusaridione
 FIESC25
100
 FIESC23
100
 Fcamp
 Fba 8
 JGI 70660
 FAVG1 11349
100
100
 Fac 1999
 Fsc 135
100
 FCIR 00355
100
 Fsp2 1000
100
100
 FVEG 11086
 Fth 11475
100
100
100
 FFUJ 10058
100
C10 fusarins
 Fsa 05790
100
 Fdl 04711
100
 Fba 175
 FIESC25
100
 FIESC28
 FPSE 07914
100
 FGSG 07798
100
 Fcu 1454
100
 Fpo 1381
 Fay 1582

## Slide 3
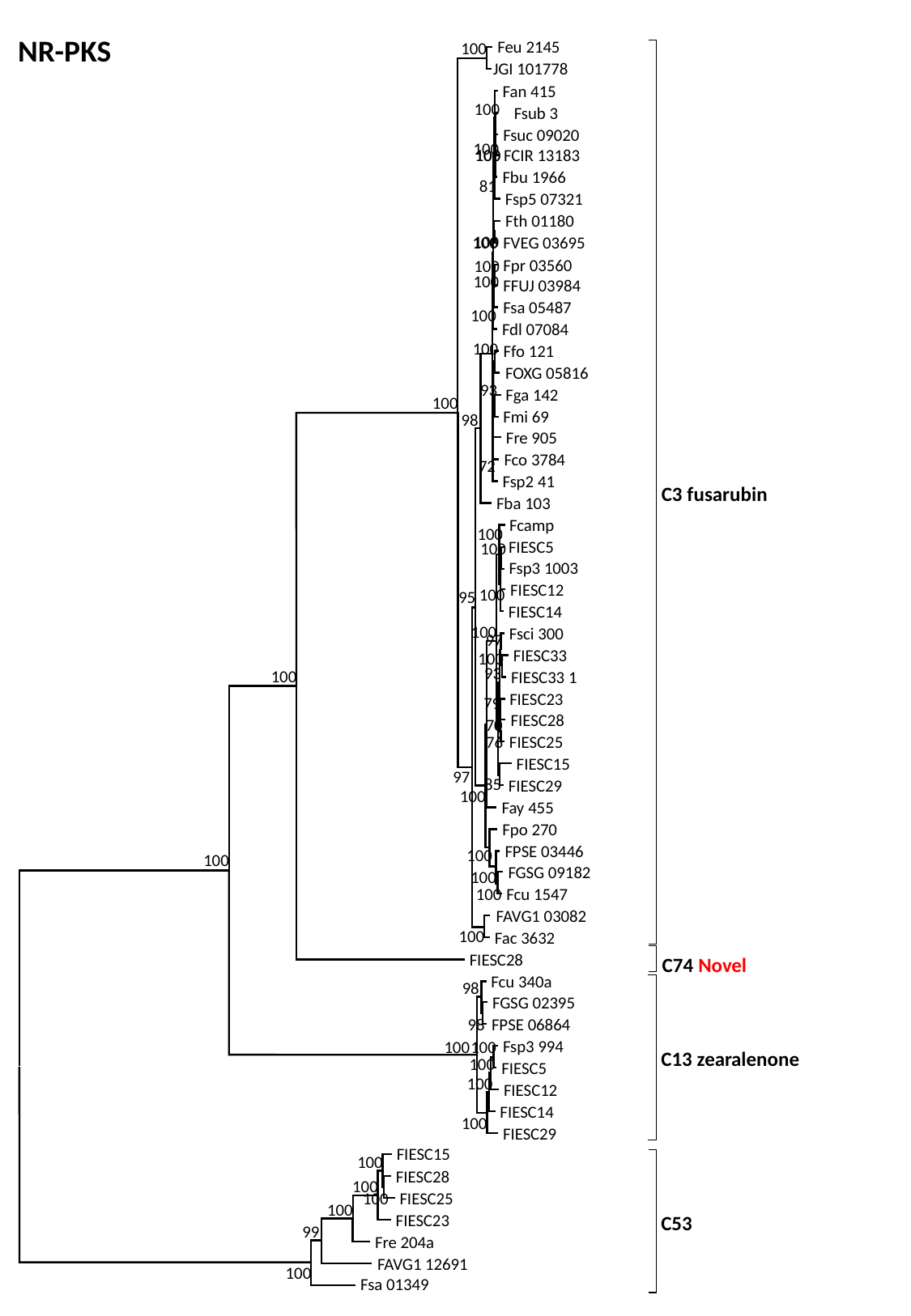

NR-PKS
 Feu 2145
100
100
100
100
100
81
100
100
100
100
100
100
93
98
72
100
100
100
JGI 101778
 Fan 415
 Fsub 3
 Fsuc 09020
 FCIR 13183
 Fbu 1966
 Fsp5 07321
 Fth 01180
 FVEG 03695
 Fpr 03560
 FFUJ 03984
 Fsa 05487
 Fdl 07084
 Ffo 121
 FOXG 05816
 Fga 142
100
95
100
97
100
93
100
79
70
76
97
85
100
100
100
100
100
100
98
98
100
100
100
100
100
100
100
100
100
99
100
 Fmi 69
 Fre 905
 Fco 3784
 Fsp2 41
C3 fusarubin
 Fba 103
 Fcamp
 FIESC5
 Fsp3 1003
 FIESC12
 FIESC14
 Fsci 300
 FIESC33
 FIESC33 1
 FIESC23
 FIESC28
 FIESC25
 FIESC15
 FIESC29
 Fay 455
 Fpo 270
 FPSE 03446
 FGSG 09182
 Fcu 1547
 FAVG1 03082
 Fac 3632
 FIESC28
C74 Novel
 Fcu 340a
 FGSG 02395
 FPSE 06864
 Fsp3 994
C13 zearalenone
 FIESC5
 FIESC12
 FIESC14
 FIESC29
 FIESC15
 FIESC28
 FIESC25
 FIESC23
C53
 Fre 204a
 FAVG1 12691
 Fsa 01349

## Slide 4
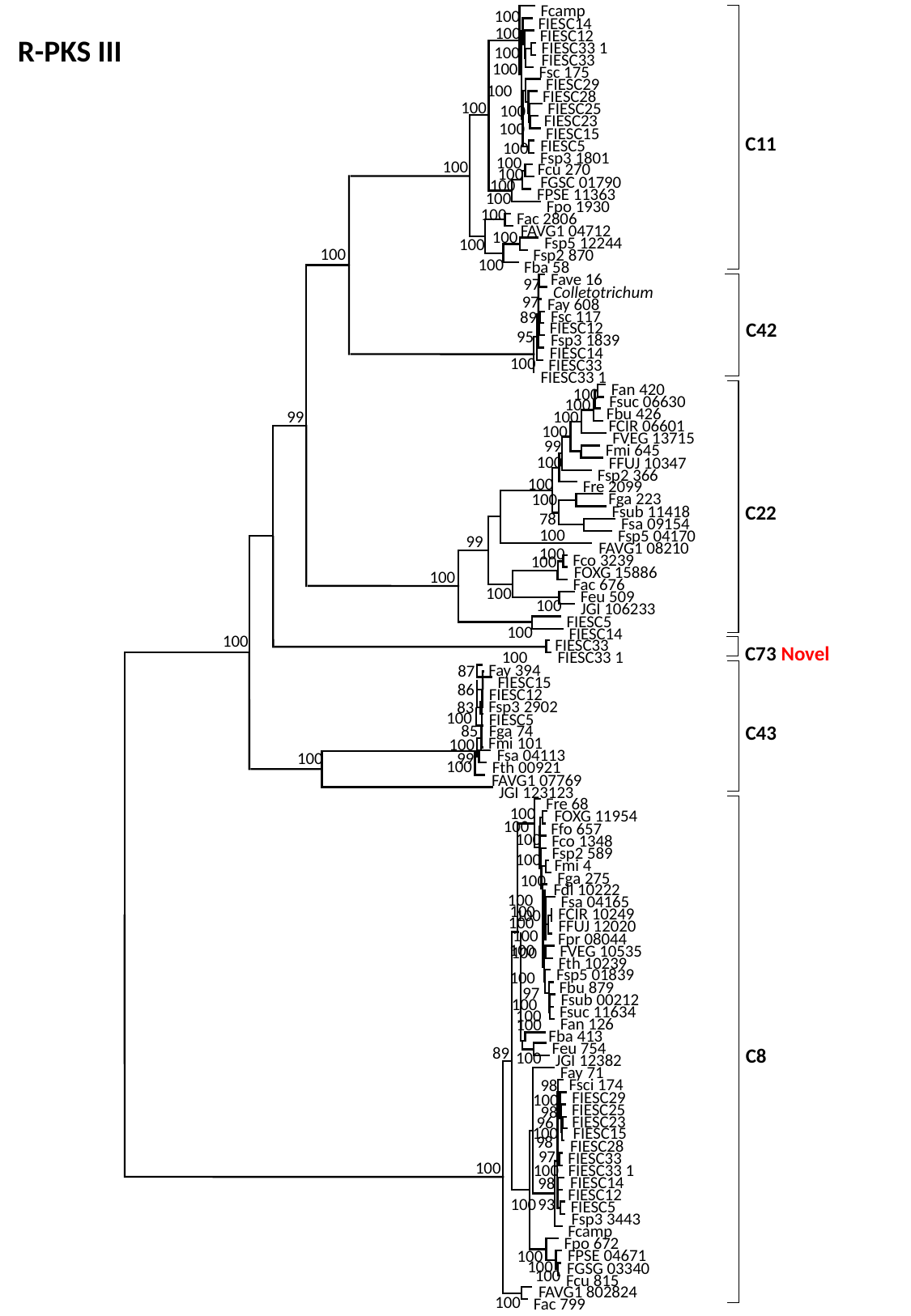

Fcamp
100
 FIESC14
100
 FIESC12
R-PKS III
 FIESC33 1
100
 FIESC33
100
 Fsc 175
 FIESC29
100
 FIESC28
100
 FIESC25
100
 FIESC23
100
 FIESC15
C11
 FIESC5
100
 Fsp3 1801
100
100
 Fcu 270
100
 FGSC 01790
100
 FPSE 11363
100
 Fpo 1930
100
 Fac 2806
 FAVG1 04712
100
 Fsp5 12244
100
100
 Fsp2 870
100
 Fba 58
 Fave 16
97
 Colletotrichum
97
 Fay 608
 Fsc 117
89
 FIESC12
C42
95
 Fsp3 1839
 FIESC14
100
 FIESC33
 FIESC33 1
 Fan 420
100
 Fsuc 06630
100
 Fbu 426
100
99
 FCIR 06601
100
 FVEG 13715
99
 Fmi 645
100
 FFUJ 10347
 Fsp2 366
100
 Fre 2099
 Fga 223
100
C22
 Fsub 11418
78
 Fsa 09154
100
 Fsp5 04170
99
 FAVG1 08210
100
 Fco 3239
100
 FOXG 15886
100
 Fac 676
100
 Feu 509
100
 JGI 106233
 FIESC5
100
 FIESC14
100
 FIESC33
C73 Novel
100
 FIESC33 1
 Fay 394
87
 FIESC15
86
 FIESC12
 Fsp3 2902
83
100
 FIESC5
C43
 Fga 74
85
 Fmi 101
100
 Fsa 04113
100
99
100
 Fth 00921
 FAVG1 07769
 JGI 123123
 Fre 68
100
 FOXG 11954
100
 Ffo 657
100
 Fco 1348
 Fsp2 589
100
 Fmi 4
 Fga 275
100
 Fdl 10222
100
 Fsa 04165
100
 FCIR 10249
100
100
 FFUJ 12020
100
 Fpr 08044
100
 FVEG 10535
100
 Fth 10239
 Fsp5 01839
100
 Fbu 879
97
 Fsub 00212
100
 Fsuc 11634
100
 Fan 126
100
 Fba 413
 Feu 754
89
C8
100
 JGI 12382
 Fay 71
 Fsci 174
98
 FIESC29
100
 FIESC25
98
 FIESC23
96
100
 FIESC15
98
 FIESC28
97
 FIESC33
100
100
 FIESC33 1
 FIESC14
98
 FIESC12
100
93
 FIESC5
 Fsp3 3443
 Fcamp
 Fpo 672
 FPSE 04671
100
100
 FGSG 03340
100
 Fcu 815
 FAVG1 802824
100
 Fac 799
