# Supplementary material for: Variation in secondary metabolite production potential in the Fusarium incarnatum-equiseti species complex revealed by comparative analysis of 13 genomes
Source: BMC Genomics. 2019 Apr 24;20:314. doi: 10.1186/s12864-019-5567-7 (PMC6480918; doi:10.1186/s12864-019-5567-7)
Supplement: Supplementary file 5 — Trees inferred by ML analysis of individual NRPS and PKS genes retrieved from FIESC genome sequences examined in this study. All the homologous genes retrieved from NCBI and were included in the analysis. Numbers near branches are bootstrap values based on 1000 pseudoreplicates. Bootstrap values below 70% are not shown. (PPTX 671 kb) [file 12864_2019_5567_MOESM5_ESM.pptx]

## Slide 1
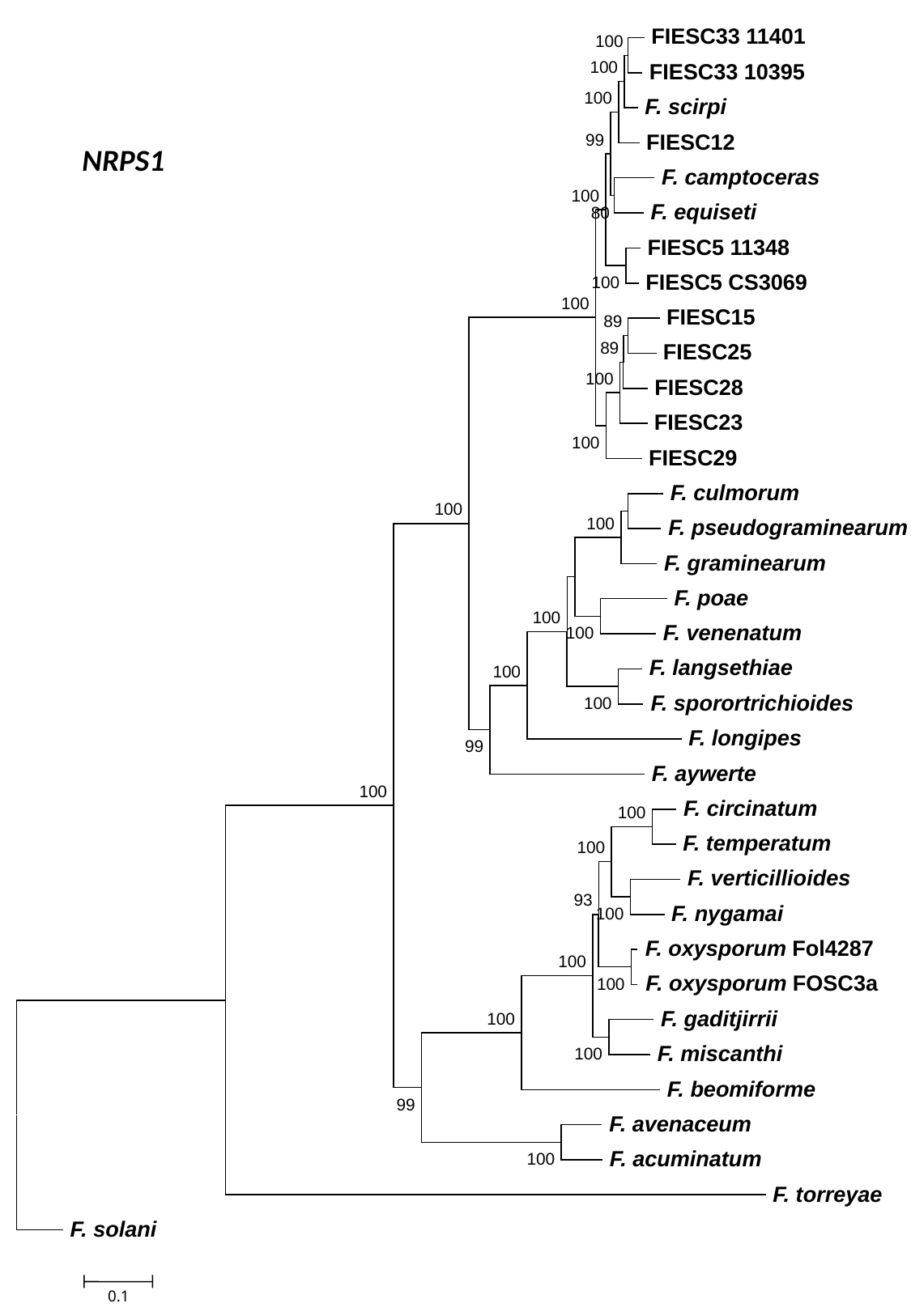

FIESC33 11401
100
100
 FIESC33 10395
100
 F. scirpi
 FIESC12
99
 F. camptoceras
100
 F. equiseti
80
 FIESC5 11348
 FIESC5 CS3069
100
100
 FIESC15
89
89
 FIESC25
100
 FIESC28
 FIESC23
100
 FIESC29
 F. culmorum
100
100
 F. pseudograminearum
 F. graminearum
 F. poae
100
 F. venenatum
100
 F. langsethiae
100
 F. sporortrichioides
100
 F. longipes
99
 F. aywerte
100
 F. circinatum
100
 F. temperatum
100
 F. verticillioides
93
 F. nygamai
100
 F. oxysporum Fol4287
100
 F. oxysporum FOSC3a
100
 F. gaditjirrii
100
 F. miscanthi
100
 F. beomiforme
99
 F. avenaceum
 F. acuminatum
100
 F. torreyae
 F. solani
0.1
NRPS1

## Slide 2
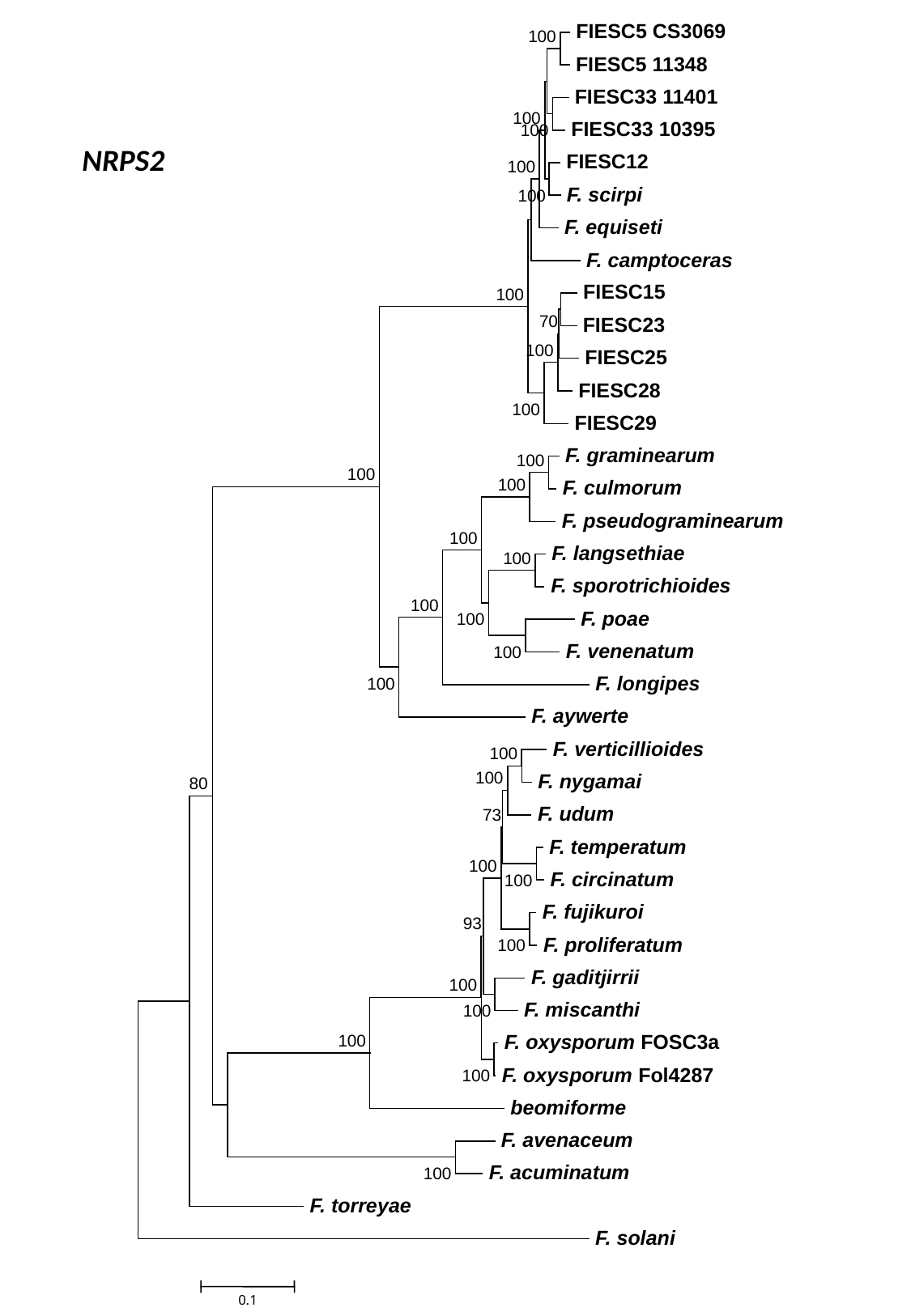

FIESC5 CS3069
100
 FIESC5 11348
 FIESC33 11401
100
 FIESC33 10395
100
 FIESC12
100
 F. scirpi
100
 F. equiseti
 F. camptoceras
 FIESC15
100
70
 FIESC23
100
 FIESC25
 FIESC28
100
 FIESC29
 F. graminearum
100
100
100
 F. culmorum
 F. pseudograminearum
100
 F. langsethiae
100
 F. sporotrichioides
100
 F. poae
100
 F. venenatum
100
 F. longipes
100
 F. aywerte
 F. verticillioides
100
100
 F. nygamai
80
 F. udum
73
 F. temperatum
100
 F. circinatum
100
 F. fujikuroi
93
 F. proliferatum
100
 F. gaditjirrii
100
 F. miscanthi
100
 F. oxysporum FOSC3a
100
 F. oxysporum Fol4287
100
 beomiforme
 F. avenaceum
 F. acuminatum
100
 F. torreyae
 F. solani
0.1
NRPS2

## Slide 3
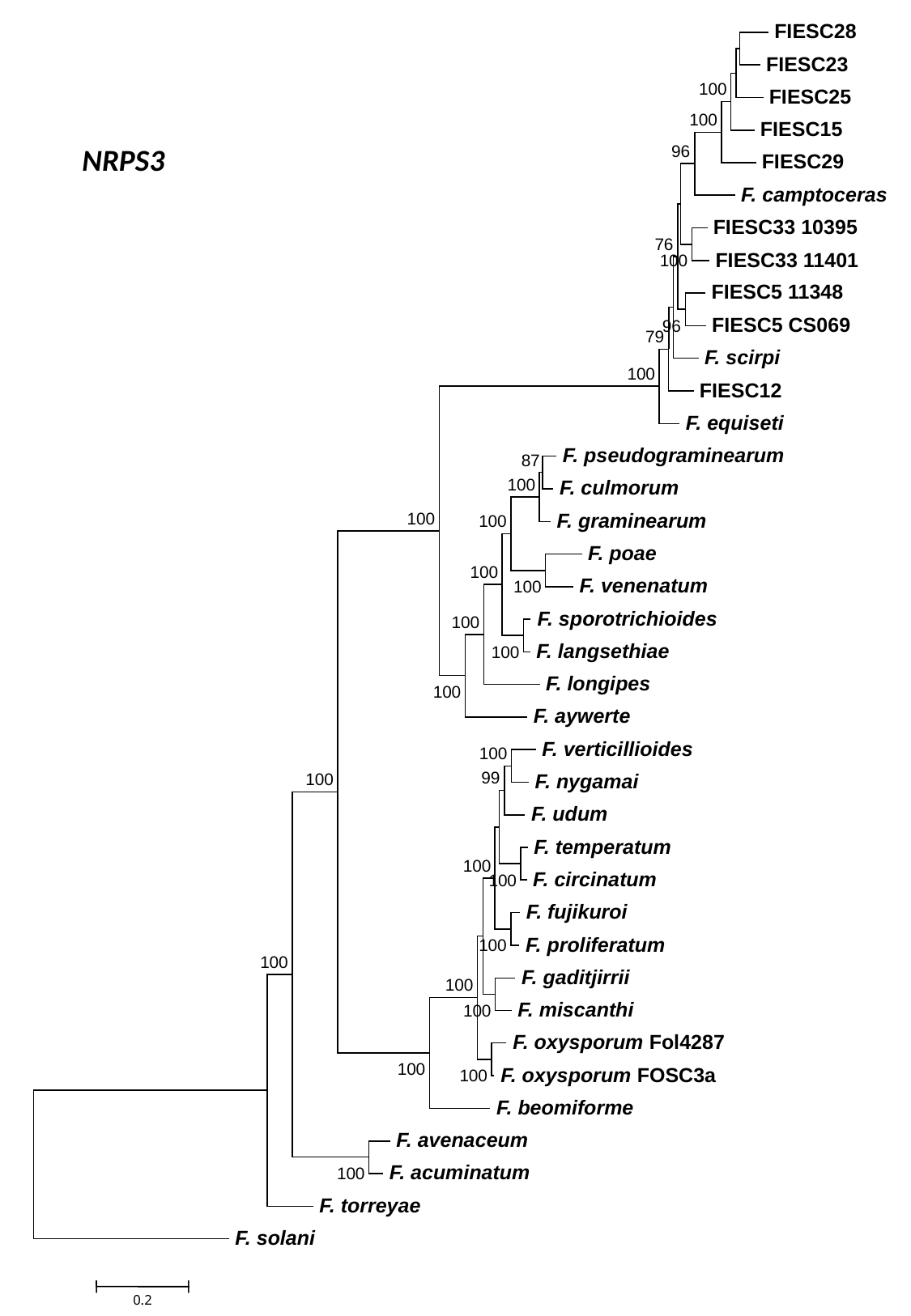

FIESC28
 FIESC23
100
 FIESC25
100
 FIESC15
96
 FIESC29
 F. camptoceras
 FIESC33 10395
76
 FIESC33 11401
100
 FIESC5 11348
 FIESC5 CS069
96
79
 F. scirpi
100
 FIESC12
 F. equiseti
 F. pseudograminearum
87
100
 F. culmorum
 F. graminearum
100
100
 F. poae
100
 F. venenatum
100
 F. sporotrichioides
100
 F. langsethiae
100
 F. longipes
100
 F. aywerte
 F. verticillioides
100
99
 F. nygamai
100
 F. udum
 F. temperatum
100
 F. circinatum
100
 F. fujikuroi
 F. proliferatum
100
100
 F. gaditjirrii
100
 F. miscanthi
100
 F. oxysporum Fol4287
100
 F. oxysporum FOSC3a
100
 F. beomiforme
 F. avenaceum
 F. acuminatum
100
 F. torreyae
 F. solani
0.2
NRPS3

## Slide 4
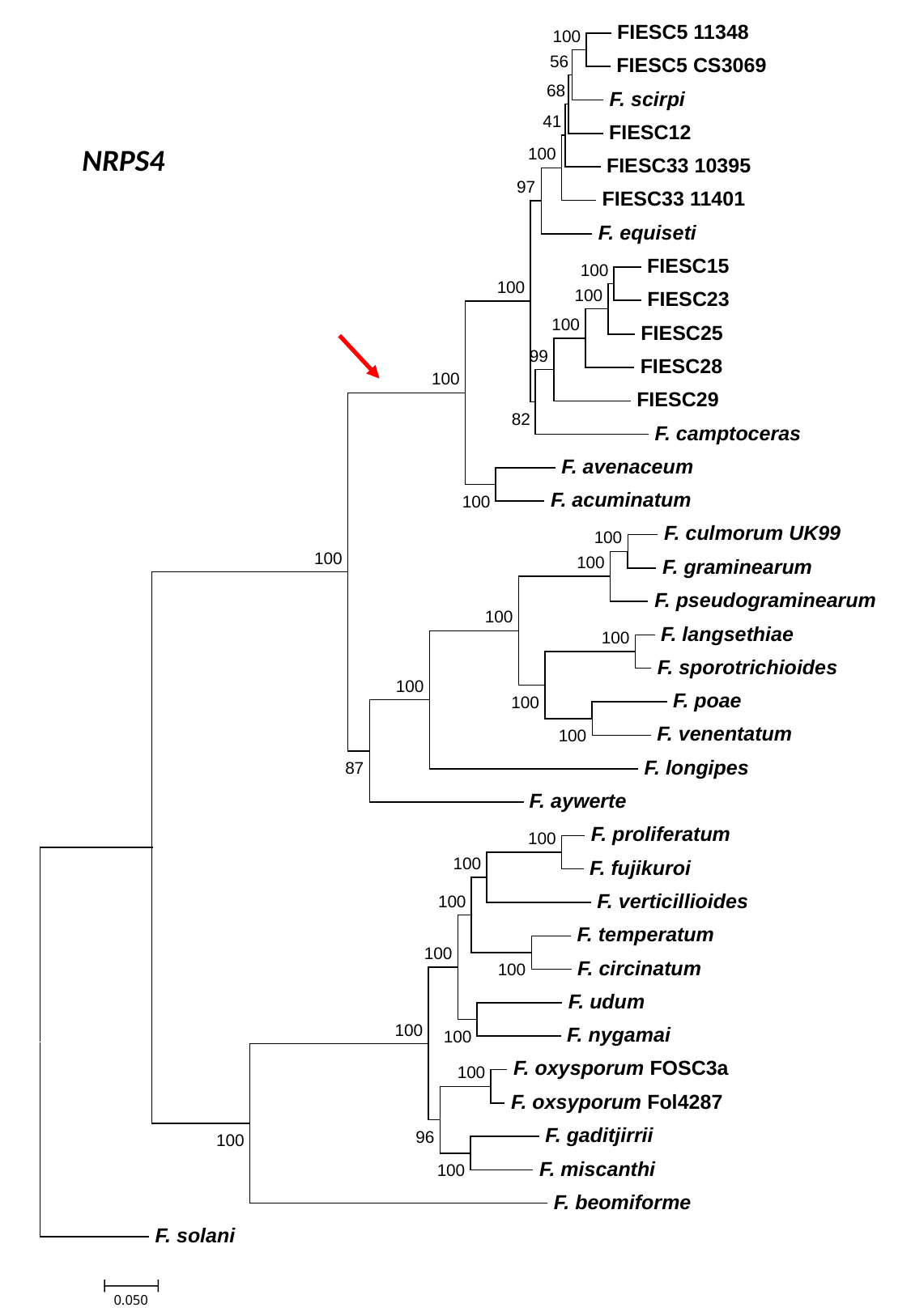

FIESC5 11348
100
56
 FIESC5 CS3069
68
 F. scirpi
41
 FIESC12
100
 FIESC33 10395
97
 FIESC33 11401
 F. equiseti
 FIESC15
100
100
100
 FIESC23
100
 FIESC25
99
 FIESC28
100
 FIESC29
82
 F. camptoceras
 F. avenaceum
 F. acuminatum
100
 F. culmorum UK99
100
100
100
 F. graminearum
 F. pseudograminearum
100
 F. langsethiae
100
 F. sporotrichioides
100
 F. poae
100
 F. venentatum
100
 F. longipes
87
 F. aywerte
 F. proliferatum
100
100
 F. fujikuroi
 F. verticillioides
100
 F. temperatum
100
 F. circinatum
100
 F. udum
100
 F. nygamai
100
 F. oxysporum FOSC3a
100
 F. oxsyporum Fol4287
 F. gaditjirrii
96
100
 F. miscanthi
100
 F. beomiforme
 F. solani
0.050
NRPS4

## Slide 5
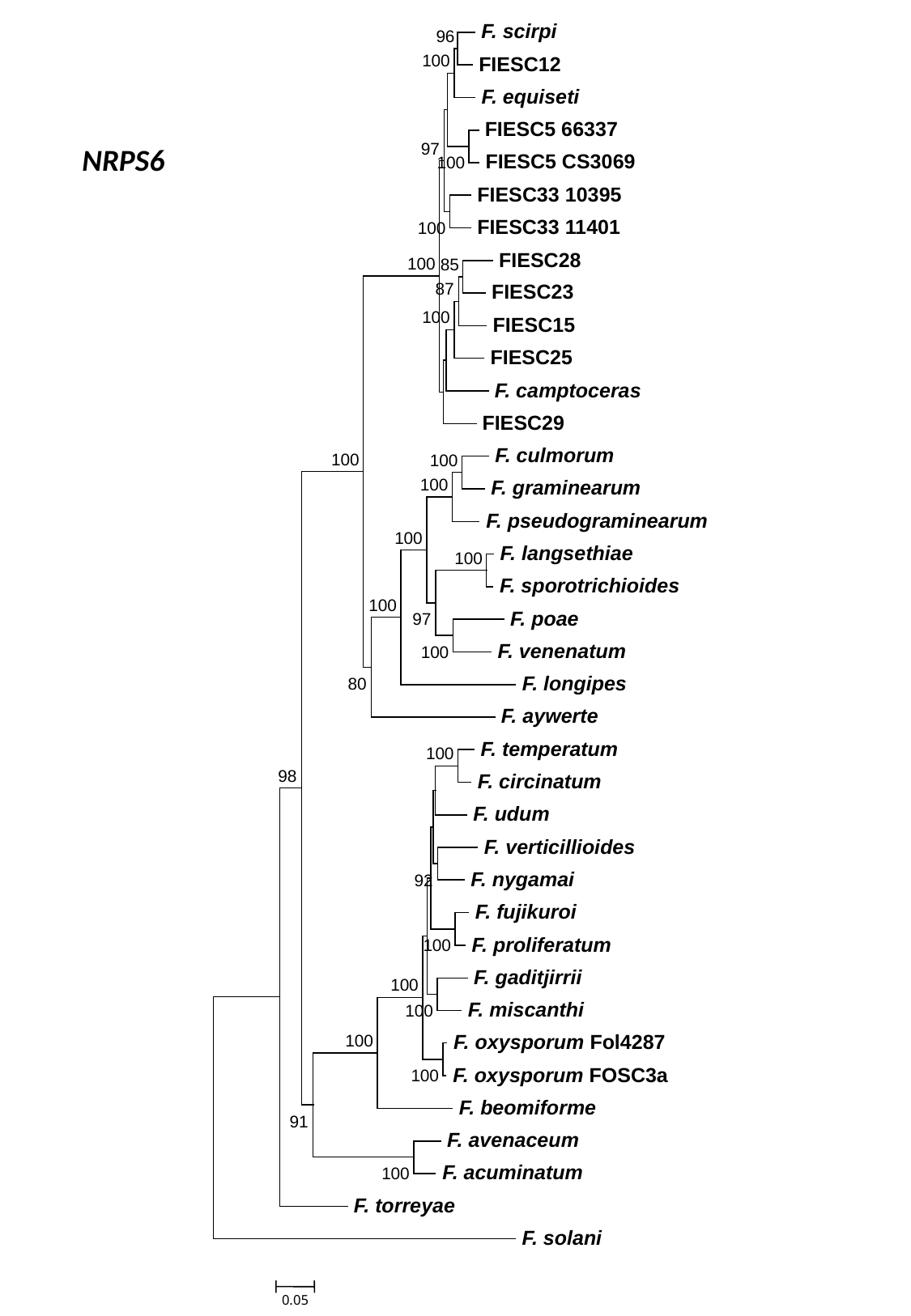

F. scirpi
96
100
 FIESC12
 F. equiseti
 FIESC5 66337
97
 FIESC5 CS3069
100
 FIESC33 10395
 FIESC33 11401
100
 FIESC28
100
85
87
 FIESC23
100
 FIESC15
 FIESC25
 F. camptoceras
 FIESC29
 F. culmorum
100
100
100
 F. graminearum
 F. pseudograminearum
100
 F. langsethiae
100
 F. sporotrichioides
100
 F. poae
97
 F. venenatum
100
 F. longipes
80
 F. aywerte
 F. temperatum
100
98
 F. circinatum
 F. udum
 F. verticillioides
 F. nygamai
92
 F. fujikuroi
 F. proliferatum
100
 F. gaditjirrii
100
 F. miscanthi
100
 F. oxysporum Fol4287
100
 F. oxysporum FOSC3a
100
 F. beomiforme
91
 F. avenaceum
 F. acuminatum
100
 F. torreyae
 F. solani
0.05
NRPS6

## Slide 6
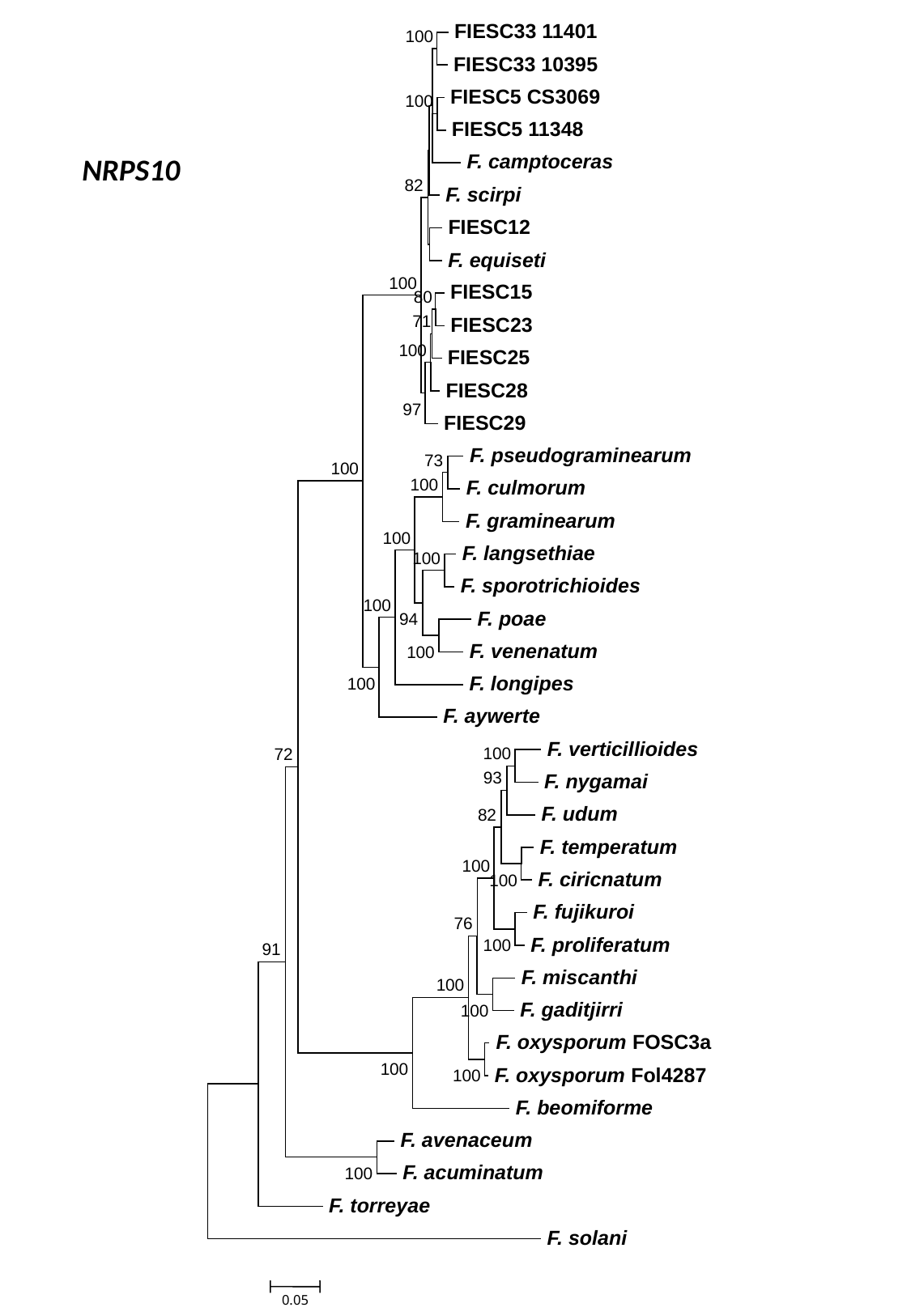

FIESC33 11401
100
 FIESC33 10395
 FIESC5 CS3069
100
 FIESC5 11348
 F. camptoceras
82
 F. scirpi
 FIESC12
 F. equiseti
100
 FIESC15
80
71
 FIESC23
100
 FIESC25
 FIESC28
97
 FIESC29
 F. pseudograminearum
73
100
100
 F. culmorum
 F. graminearum
100
 F. langsethiae
100
 F. sporotrichioides
100
 F. poae
94
 F. venenatum
100
 F. longipes
100
 F. aywerte
 F. verticillioides
100
72
93
 F. nygamai
 F. udum
82
 F. temperatum
100
 F. ciricnatum
100
 F. fujikuroi
76
 F. proliferatum
100
91
 F. miscanthi
100
 F. gaditjirri
100
 F. oxysporum FOSC3a
100
 F. oxysporum Fol4287
100
 F. beomiforme
 F. avenaceum
 F. acuminatum
100
 F. torreyae
 F. solani
0.05
NRPS10

## Slide 7
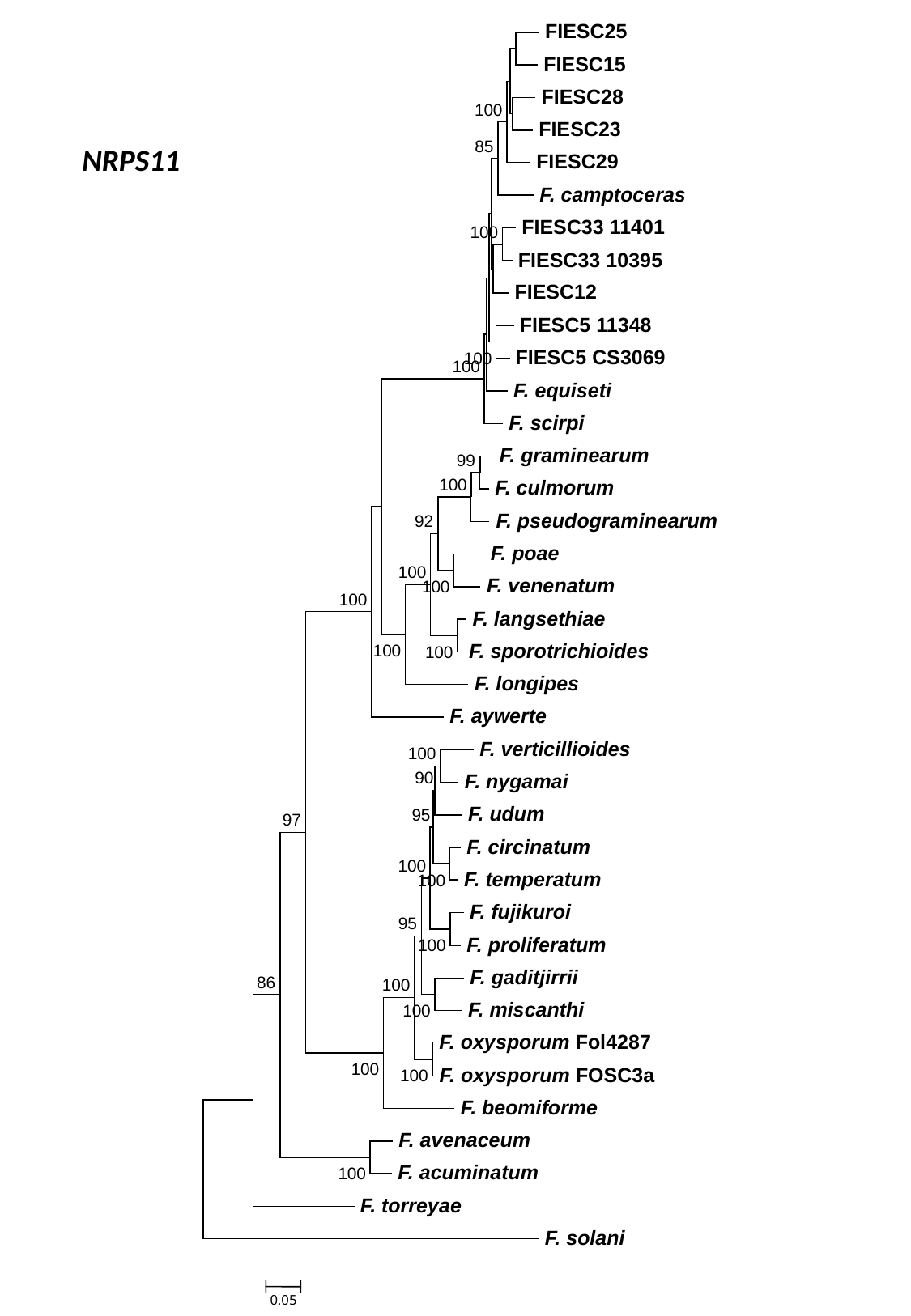

FIESC25
 FIESC15
 FIESC28
100
 FIESC23
85
 FIESC29
 F. camptoceras
 FIESC33 11401
100
 FIESC33 10395
 FIESC12
 FIESC5 11348
 FIESC5 CS3069
100
100
 F. equiseti
 F. scirpi
 F. graminearum
99
100
 F. culmorum
 F. pseudograminearum
92
 F. poae
100
 F. venenatum
100
100
 F. langsethiae
 F. sporotrichioides
100
100
 F. longipes
 F. aywerte
 F. verticillioides
100
90
 F. nygamai
 F. udum
95
97
 F. circinatum
100
 F. temperatum
100
 F. fujikuroi
95
 F. proliferatum
100
 F. gaditjirrii
86
100
 F. miscanthi
100
 F. oxysporum Fol4287
100
 F. oxysporum FOSC3a
100
 F. beomiforme
 F. avenaceum
 F. acuminatum
100
 F. torreyae
 F. solani
0.05
NRPS11

## Slide 8
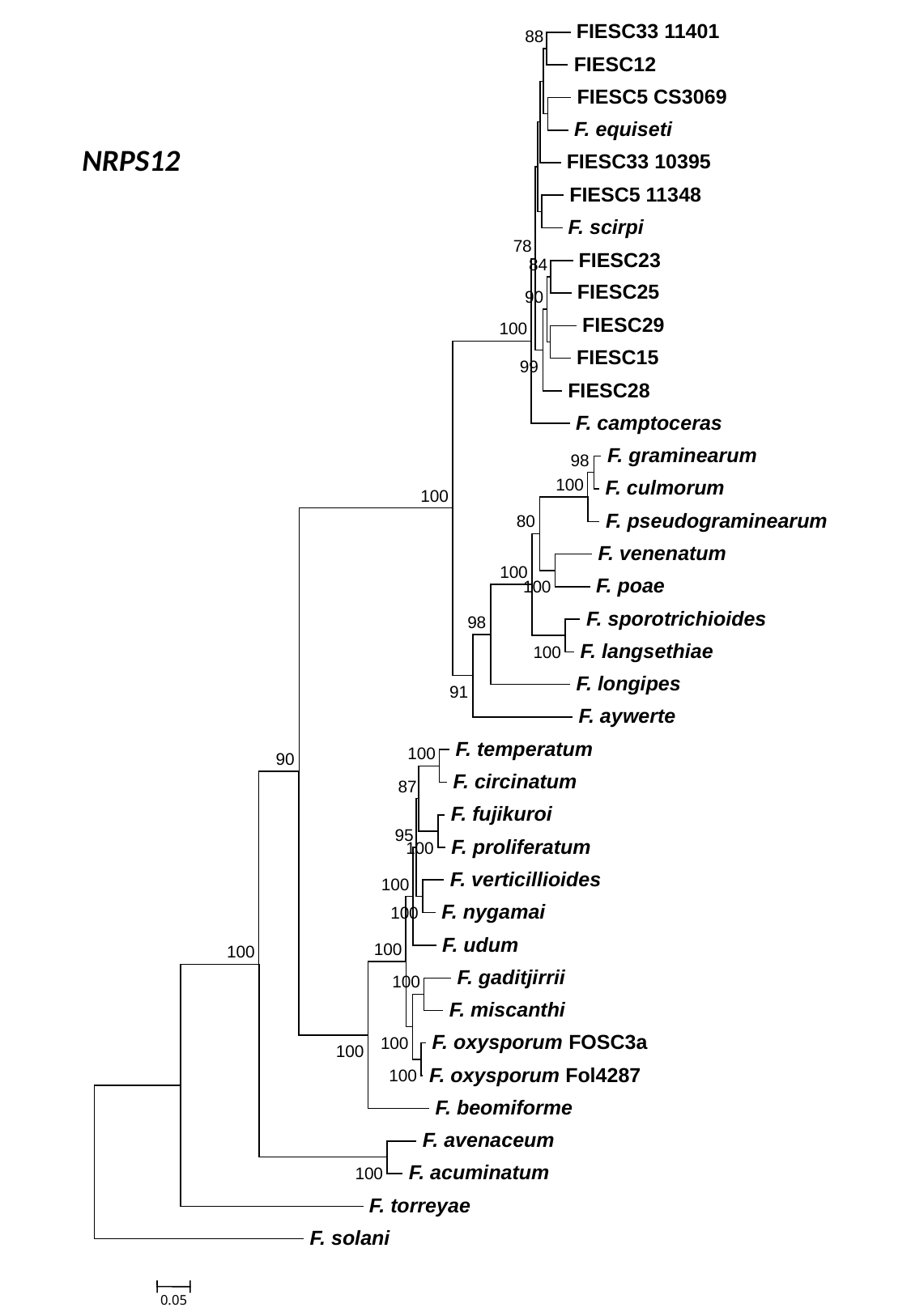

FIESC33 11401
88
 FIESC12
 FIESC5 CS3069
 F. equiseti
 FIESC33 10395
 FIESC5 11348
 F. scirpi
78
 FIESC23
84
 FIESC25
90
 FIESC29
100
 FIESC15
99
 FIESC28
 F. camptoceras
 F. graminearum
98
100
 F. culmorum
100
 F. pseudograminearum
80
 F. venenatum
100
 F. poae
100
 F. sporotrichioides
98
 F. langsethiae
100
 F. longipes
91
 F. aywerte
 F. temperatum
100
90
 F. circinatum
87
 F. fujikuroi
95
 F. proliferatum
100
 F. verticillioides
100
 F. nygamai
100
 F. udum
100
100
 F. gaditjirrii
100
 F. miscanthi
 F. oxysporum FOSC3a
100
100
 F. oxysporum Fol4287
100
 F. beomiforme
 F. avenaceum
 F. acuminatum
100
 F. torreyae
 F. solani
0.05
NRPS12

## Slide 9
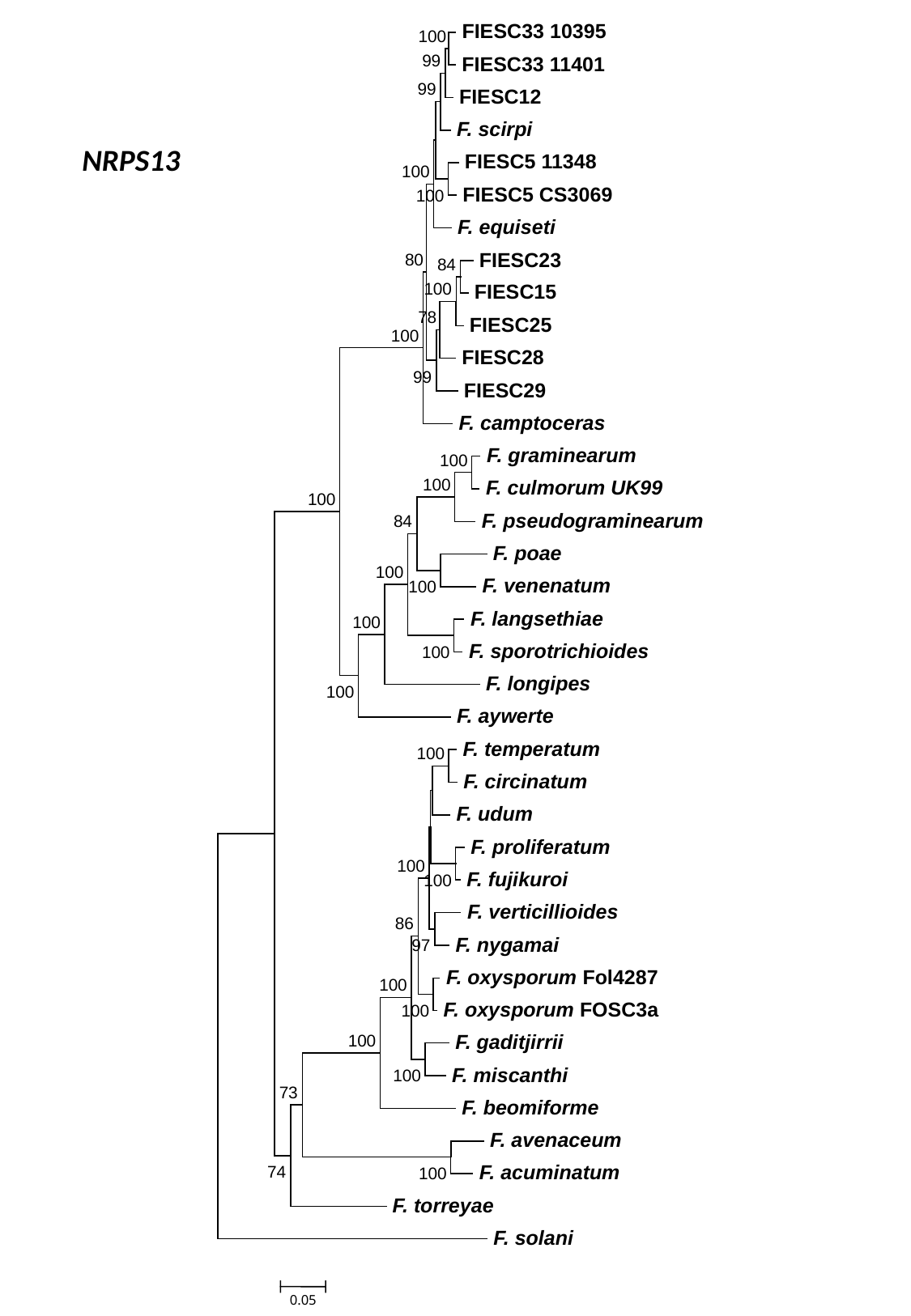

FIESC33 10395
100
99
 FIESC33 11401
99
 FIESC12
 F. scirpi
 FIESC5 11348
100
 FIESC5 CS3069
100
 F. equiseti
 FIESC23
80
84
100
 FIESC15
78
 FIESC25
100
 FIESC28
99
 FIESC29
 F. camptoceras
 F. graminearum
100
100
 F. culmorum UK99
100
 F. pseudograminearum
84
 F. poae
100
 F. venenatum
100
 F. langsethiae
100
 F. sporotrichioides
100
 F. longipes
100
 F. aywerte
 F. temperatum
100
 F. circinatum
 F. udum
 F. proliferatum
100
 F. fujikuroi
100
 F. verticillioides
86
 F. nygamai
97
 F. oxysporum Fol4287
100
 F. oxysporum FOSC3a
100
 F. gaditjirrii
100
 F. miscanthi
100
73
 F. beomiforme
 F. avenaceum
 F. acuminatum
74
100
 F. torreyae
 F. solani
0.05
NRPS13

## Slide 10
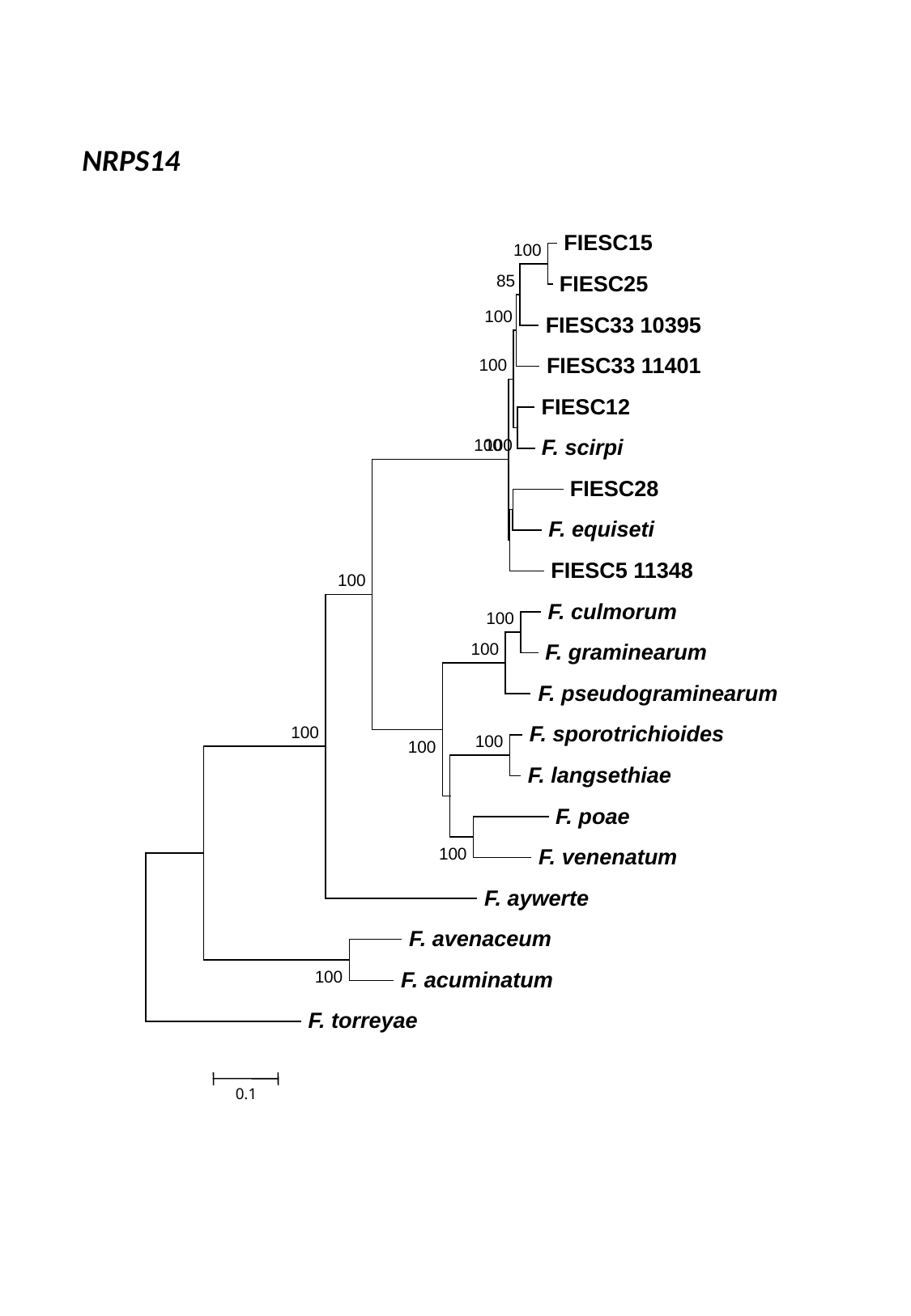

NRPS14
 FIESC15
100
 FIESC25
85
100
 FIESC33 10395
 FIESC33 11401
100
 FIESC12
 F. scirpi
100
100
 FIESC28
 F. equiseti
 FIESC5 11348
100
 F. culmorum
100
 F. graminearum
100
 F. pseudograminearum
 F. sporotrichioides
100
100
100
 F. langsethiae
 F. poae
 F. venenatum
100
 F. aywerte
 F. avenaceum
 F. acuminatum
100
 F. torreyae
0.1

## Slide 11
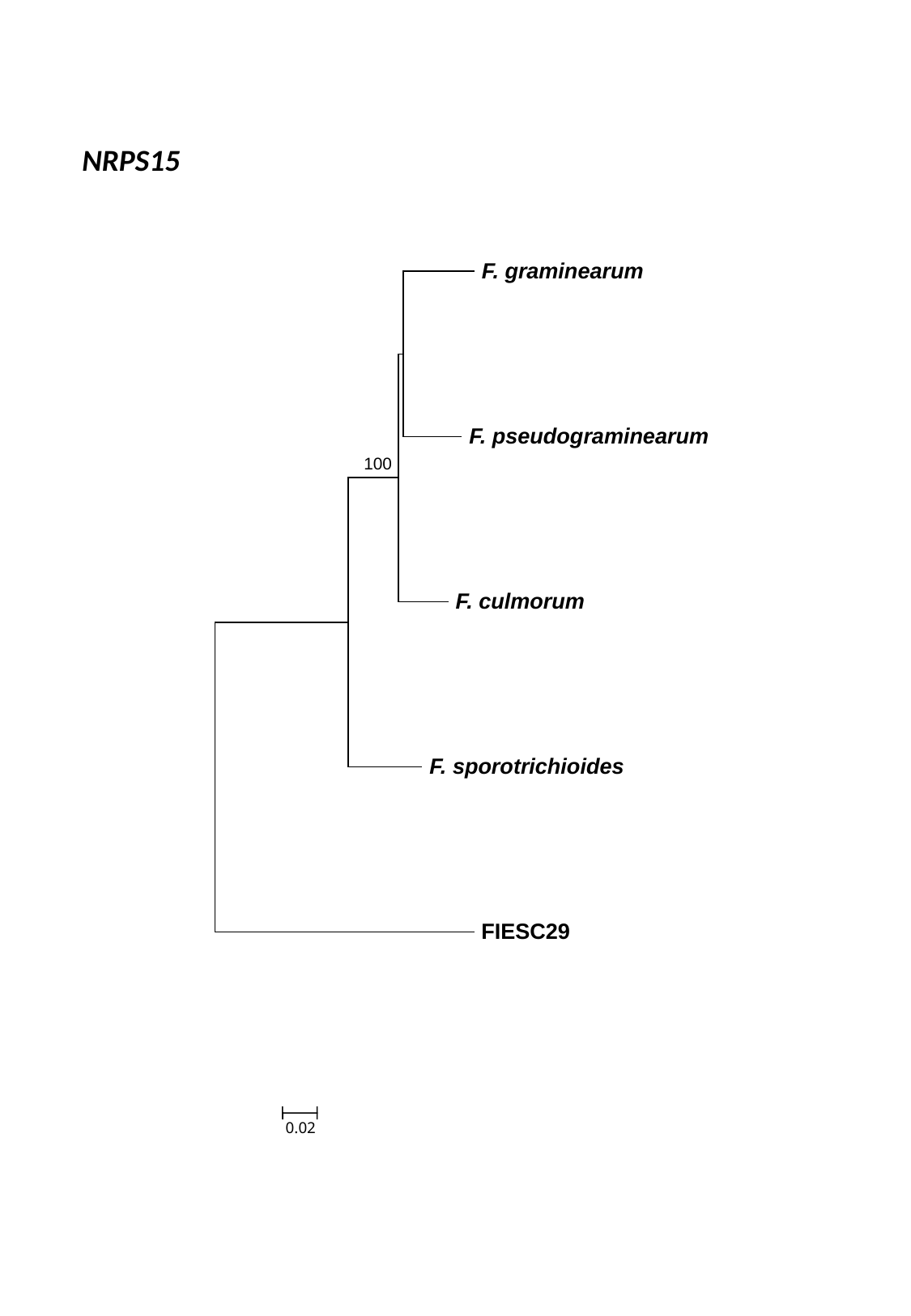

NRPS15
 F. graminearum
 F. pseudograminearum
100
 F. culmorum
 F. sporotrichioides
 FIESC29
0.02

## Slide 12
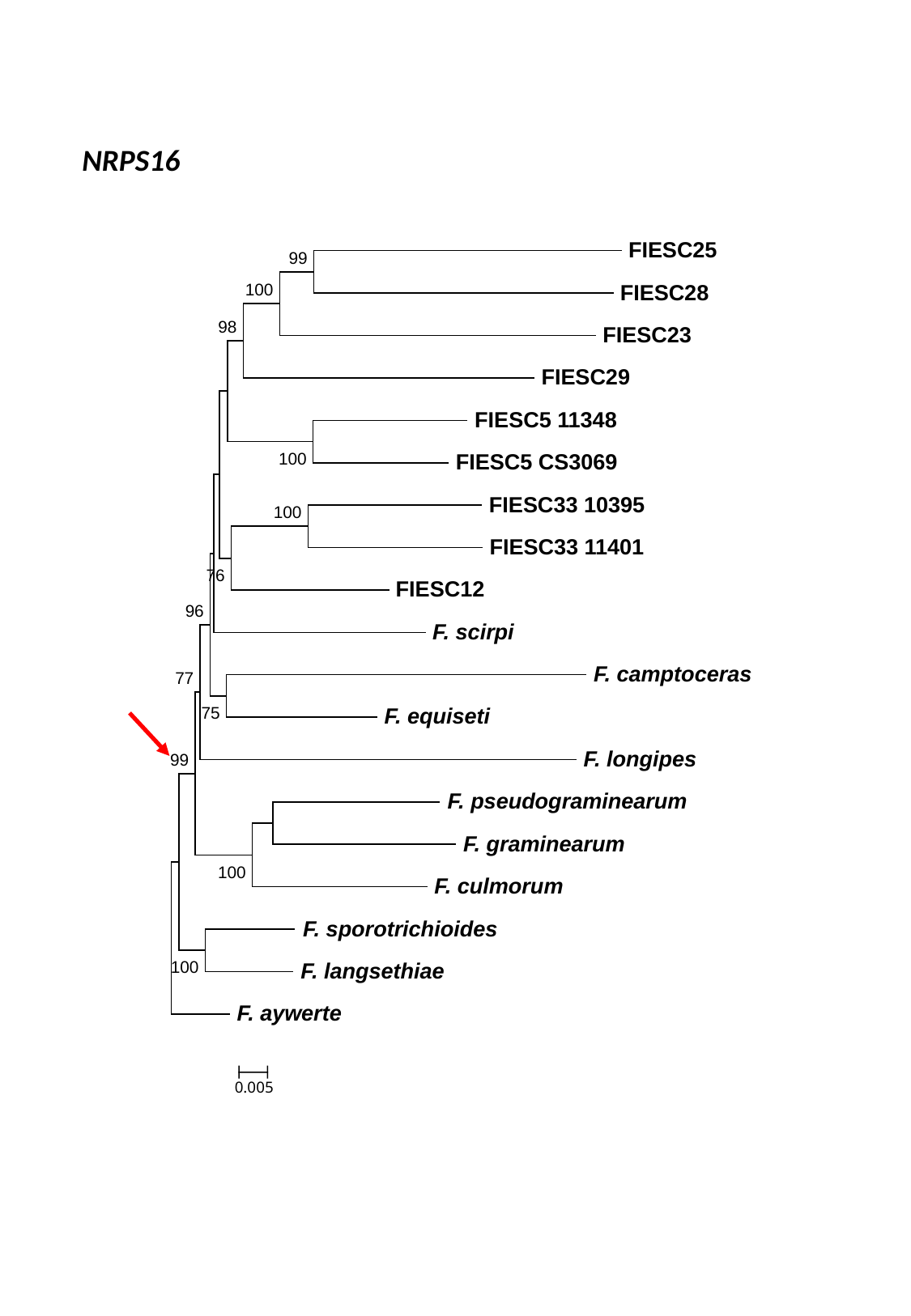

NRPS16
 FIESC25
99
 FIESC28
100
98
 FIESC23
 FIESC29
 FIESC5 11348
100
 FIESC5 CS3069
 FIESC33 10395
100
 FIESC33 11401
76
 FIESC12
96
 F. scirpi
 F. camptoceras
77
75
 F. equiseti
 F. longipes
99
 F. pseudograminearum
 F. graminearum
100
 F. culmorum
 F. sporotrichioides
100
 F. langsethiae
 F. aywerte
0.005

## Slide 13
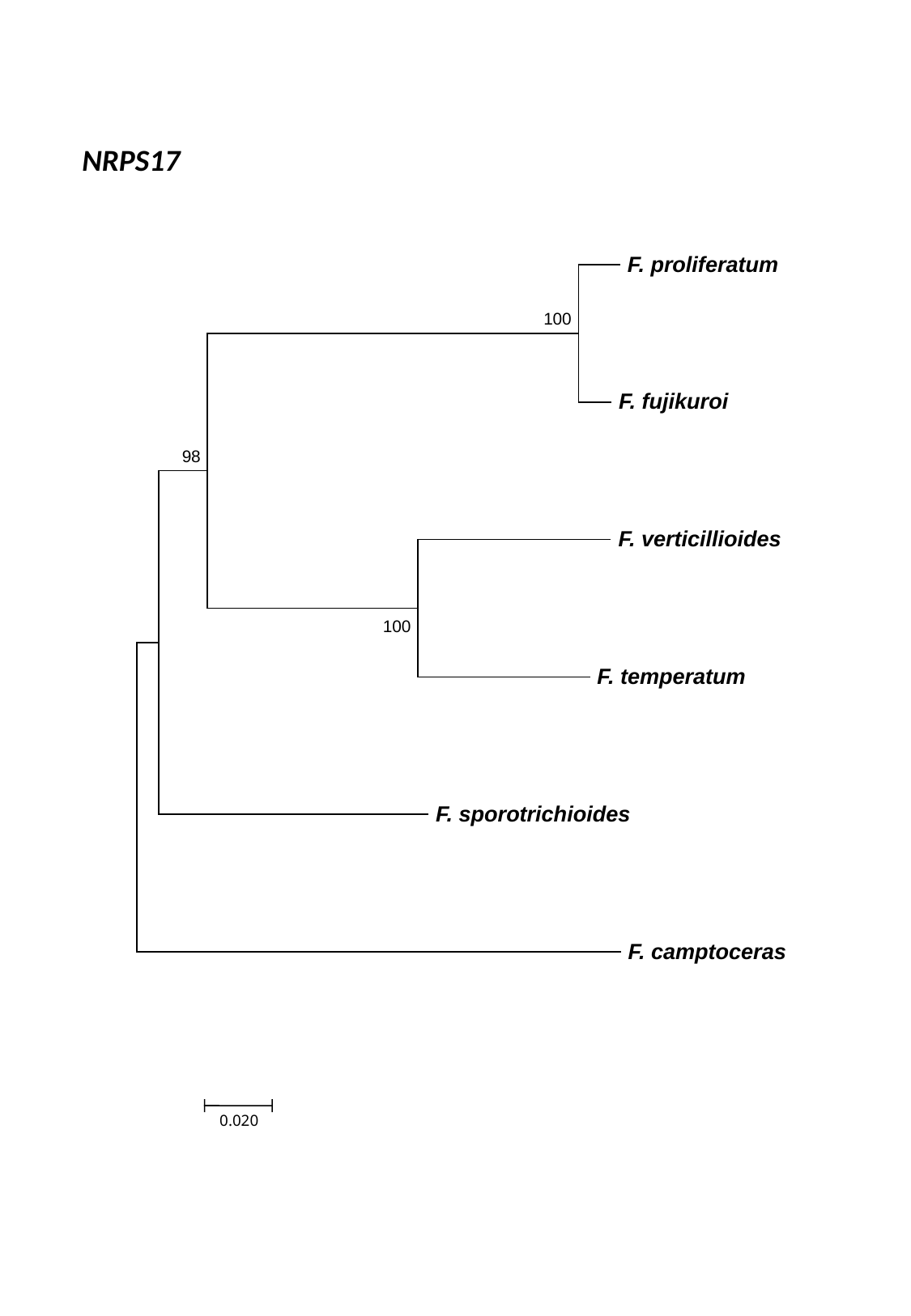

NRPS17
 F. proliferatum
100
 F. fujikuroi
98
 F. verticillioides
100
 F. temperatum
 F. sporotrichioides
 F. camptoceras
0.020

## Slide 14
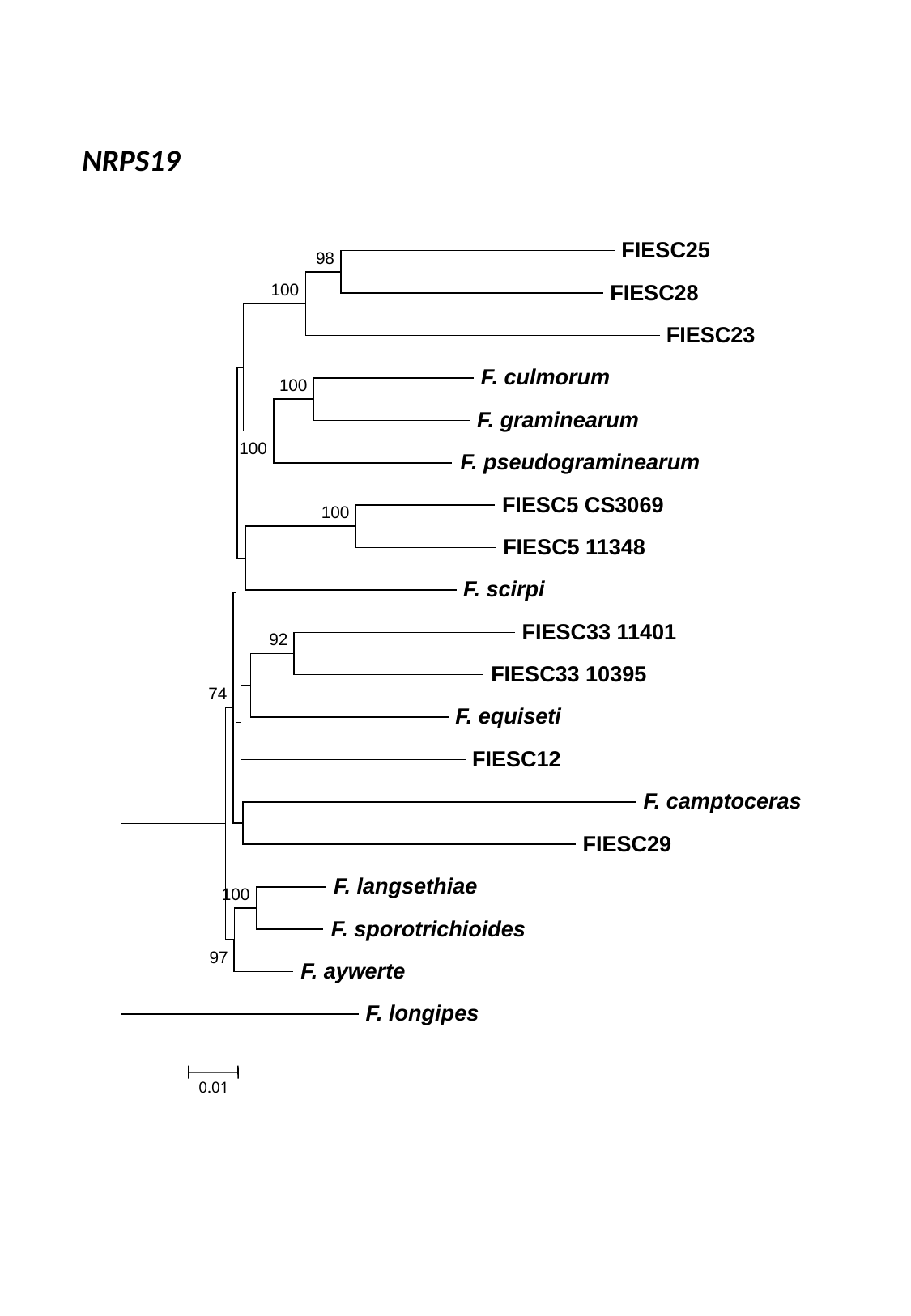

NRPS19
 FIESC25
98
 FIESC28
100
 FIESC23
 F. culmorum
100
 F. graminearum
100
 F. pseudograminearum
 FIESC5 CS3069
100
 FIESC5 11348
 F. scirpi
 FIESC33 11401
92
 FIESC33 10395
74
 F. equiseti
 FIESC12
 F. camptoceras
 FIESC29
 F. langsethiae
100
 F. sporotrichioides
97
 F. aywerte
 F. longipes
0.01

## Slide 15
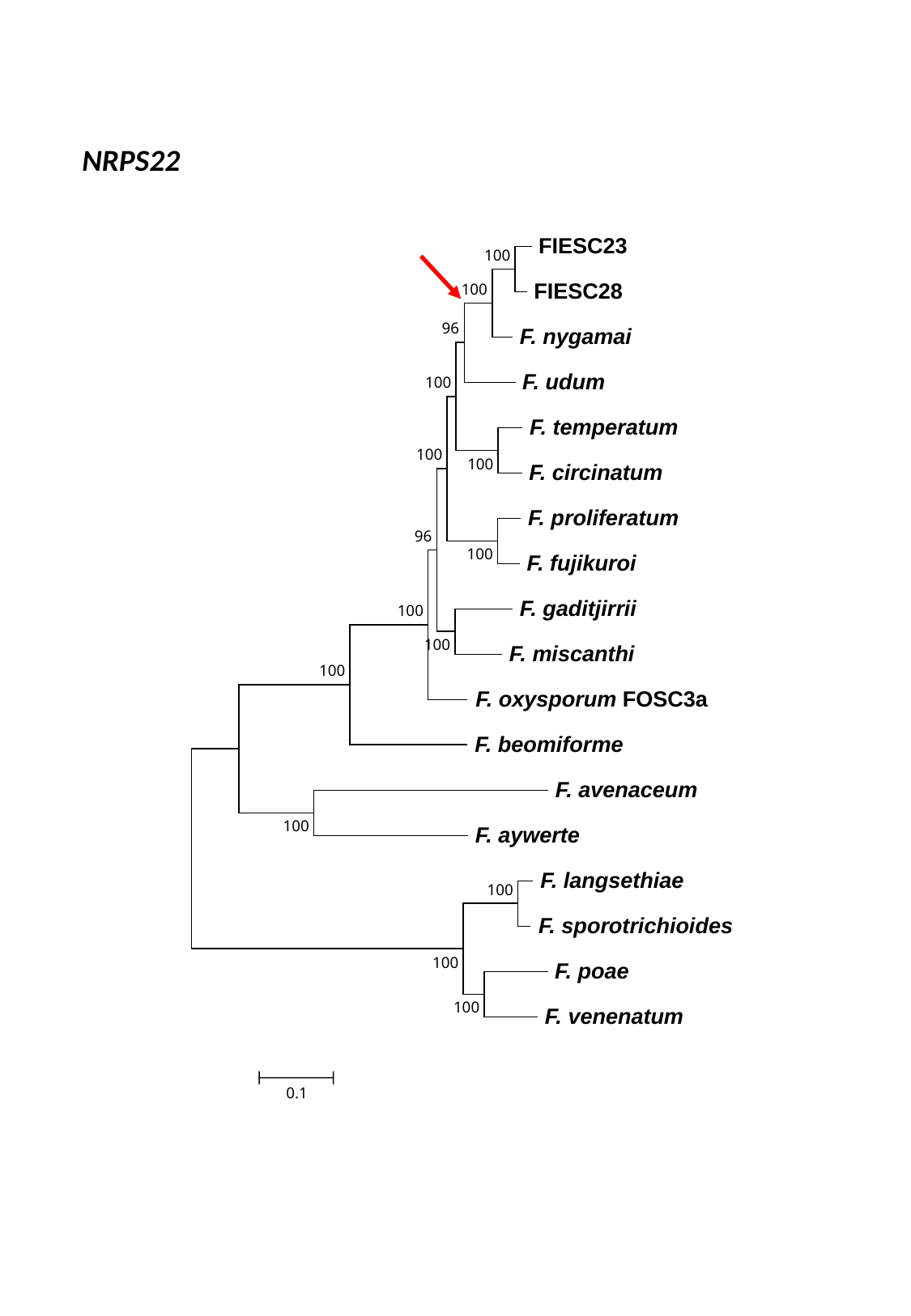

NRPS22
 FIESC23
100
 FIESC28
100
96
 F. nygamai
 F. udum
100
 F. temperatum
100
100
 F. circinatum
 F. proliferatum
96
100
 F. fujikuroi
 F. gaditjirrii
100
100
 F. miscanthi
100
 F. oxysporum FOSC3a
 F. beomiforme
 F. avenaceum
100
 F. aywerte
 F. langsethiae
100
 F. sporotrichioides
100
 F. poae
100
 F. venenatum
0.1

## Slide 16
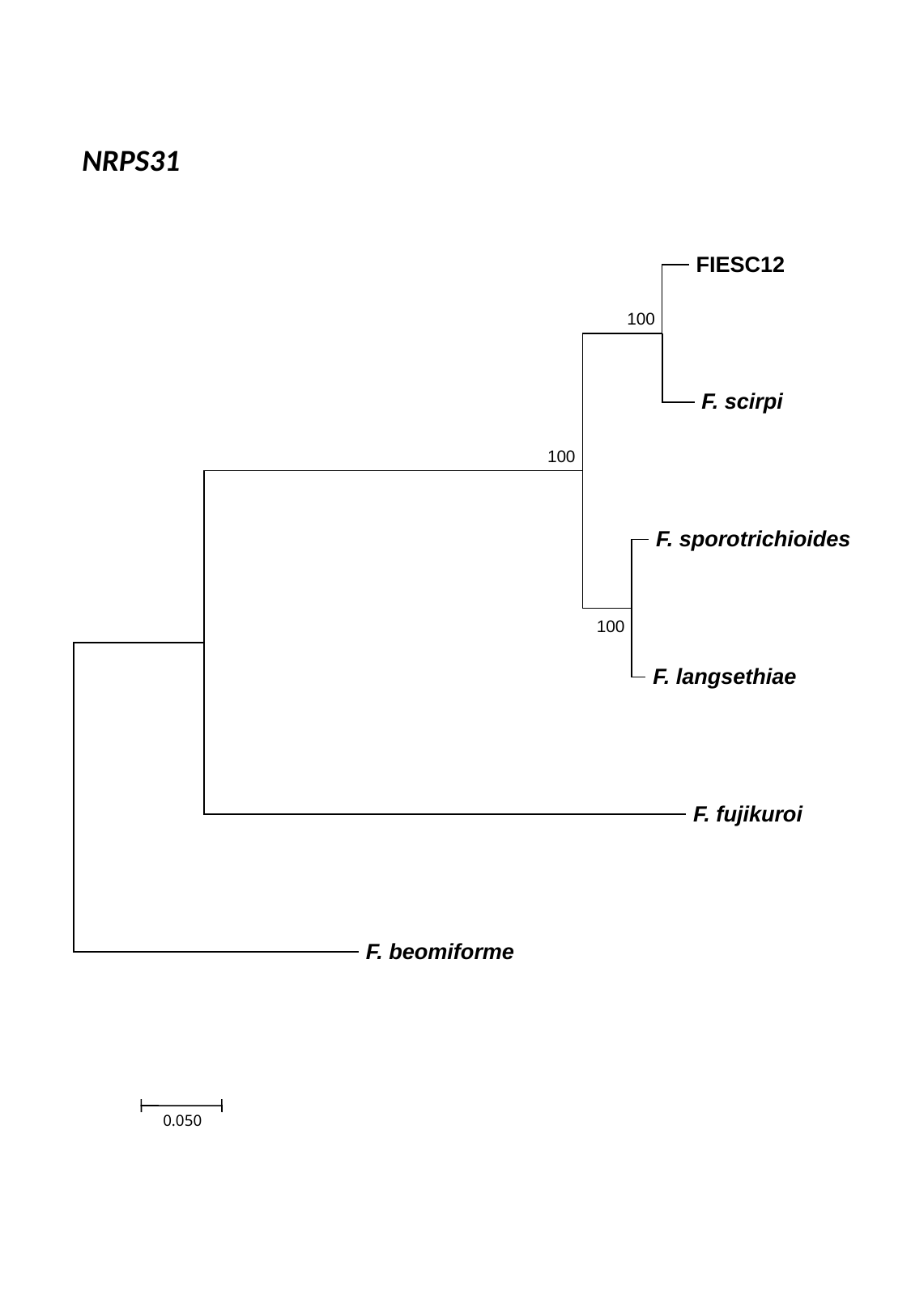

NRPS31
 FIESC12
100
 F. scirpi
100
 F. sporotrichioides
100
 F. langsethiae
 F. fujikuroi
 F. beomiforme
0.050

## Slide 17
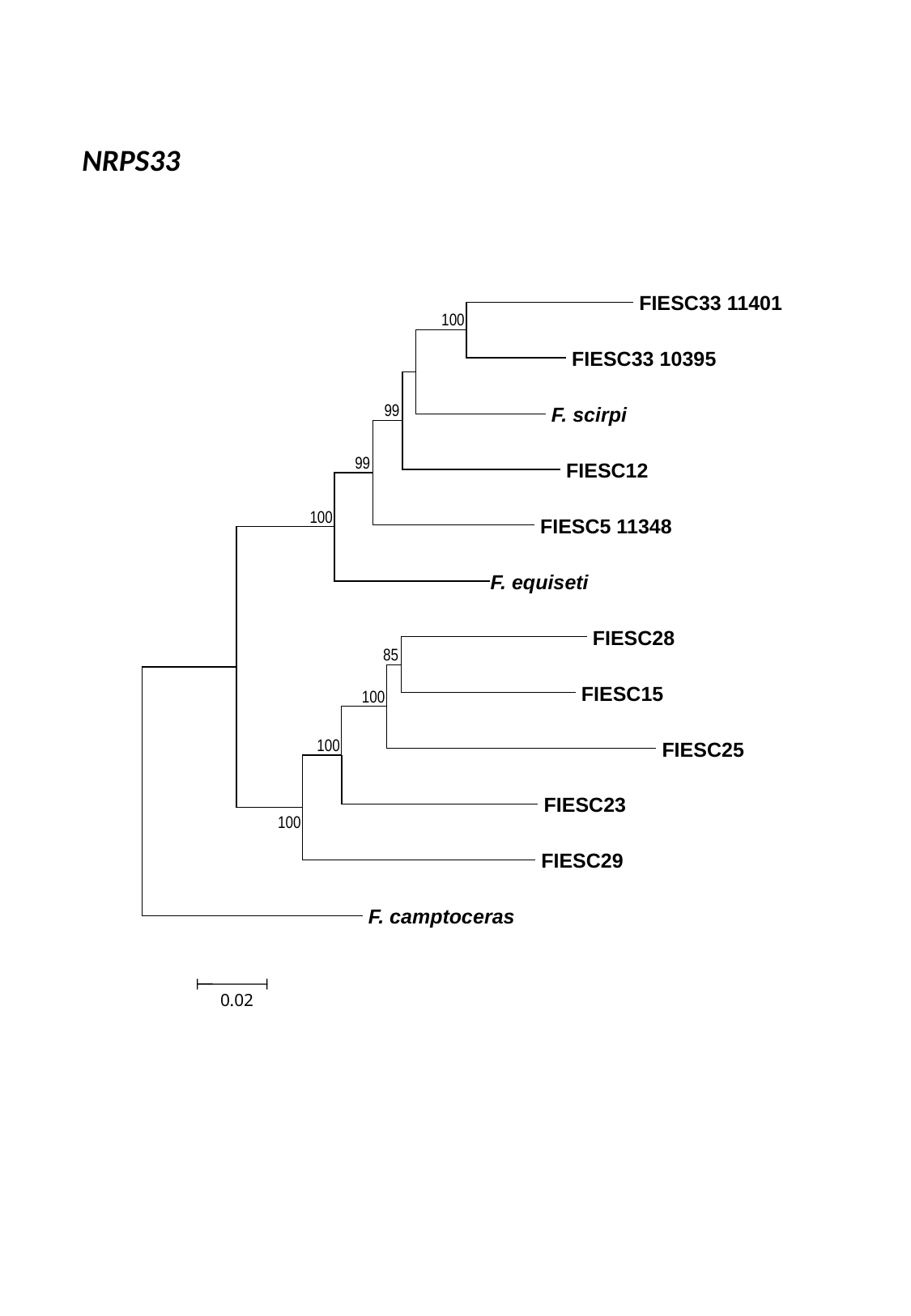

NRPS33
 FIESC33 11401
100
 FIESC33 10395
99
 F. scirpi
99
 FIESC12
100
 FIESC5 11348
F. equiseti
 FIESC28
85
 FIESC15
100
100
 FIESC25
 FIESC23
100
 FIESC29
 F. camptoceras
0.02

## Slide 18
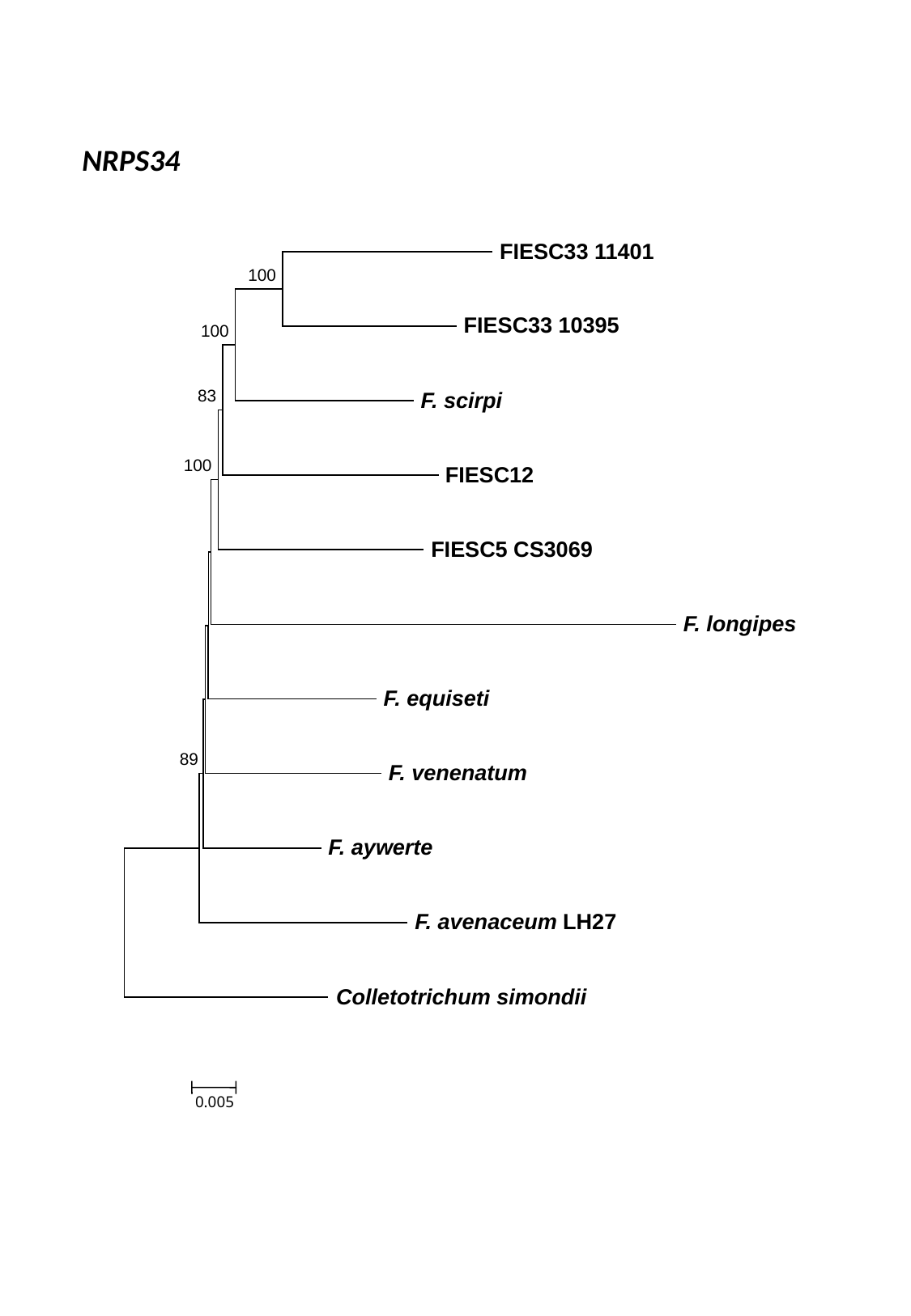

NRPS34
 FIESC33 11401
100
 FIESC33 10395
100
83
 F. scirpi
100
 FIESC12
 FIESC5 CS3069
 F. longipes
 F. equiseti
89
 F. venenatum
 F. aywerte
 F. avenaceum LH27
 Colletotrichum simondii
0.005

## Slide 19
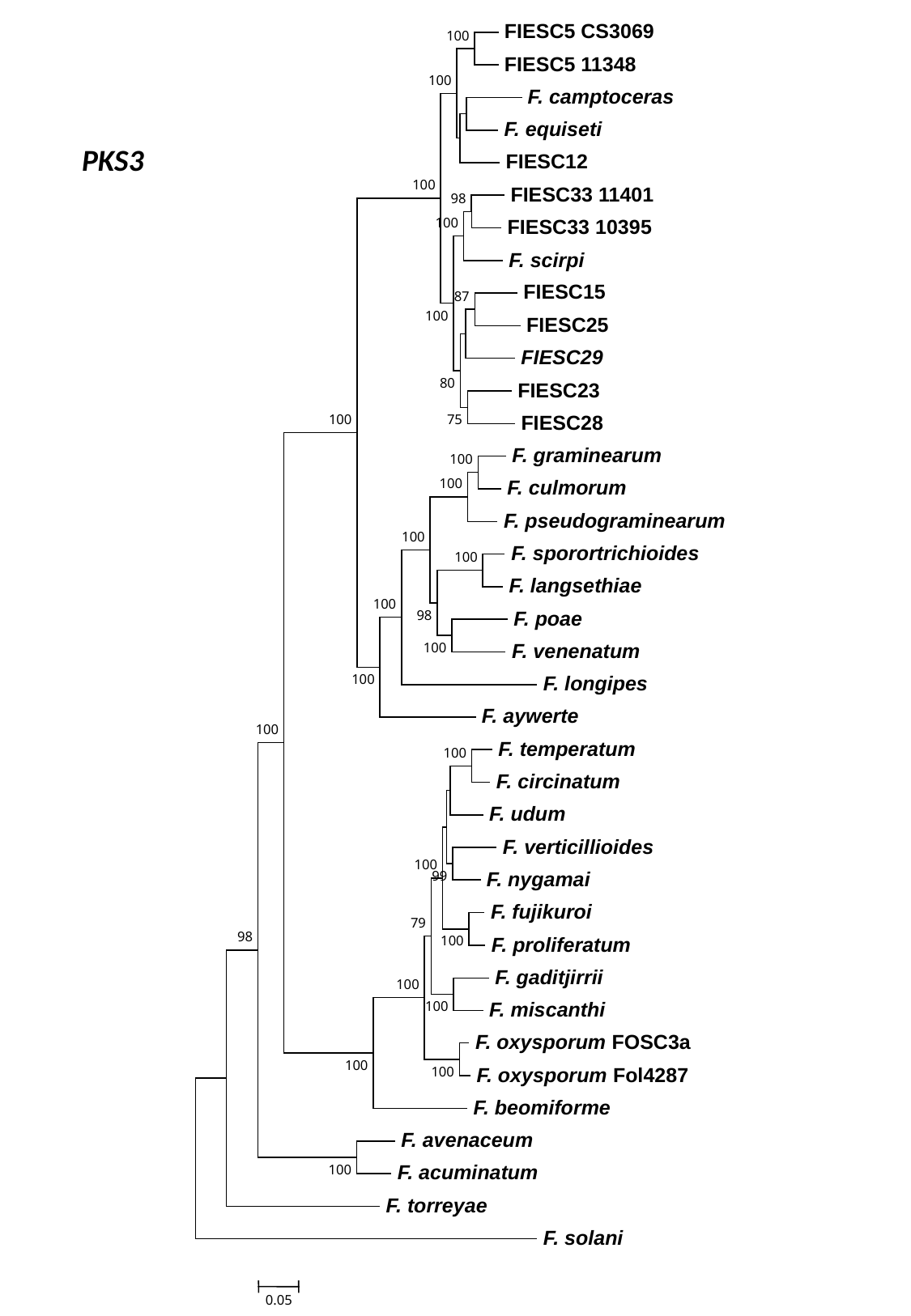

FIESC5 CS3069
100
 FIESC5 11348
100
 F. camptoceras
 F. equiseti
 FIESC12
100
 FIESC33 11401
98
 FIESC33 10395
100
 F. scirpi
 FIESC15
87
100
 FIESC25
 FIESC29
80
 FIESC23
 FIESC28
100
75
 F. graminearum
100
 F. culmorum
100
 F. pseudograminearum
100
 F. sporortrichioides
100
 F. langsethiae
100
 F. poae
98
 F. venenatum
100
 F. longipes
100
 F. aywerte
100
 F. temperatum
100
 F. circinatum
 F. udum
 F. verticillioides
100
 F. nygamai
99
 F. fujikuroi
79
98
 F. proliferatum
100
 F. gaditjirrii
100
 F. miscanthi
100
 F. oxysporum FOSC3a
100
 F. oxysporum Fol4287
100
 F. beomiforme
 F. avenaceum
 F. acuminatum
100
 F. torreyae
 F. solani
0.05
PKS3

## Slide 20
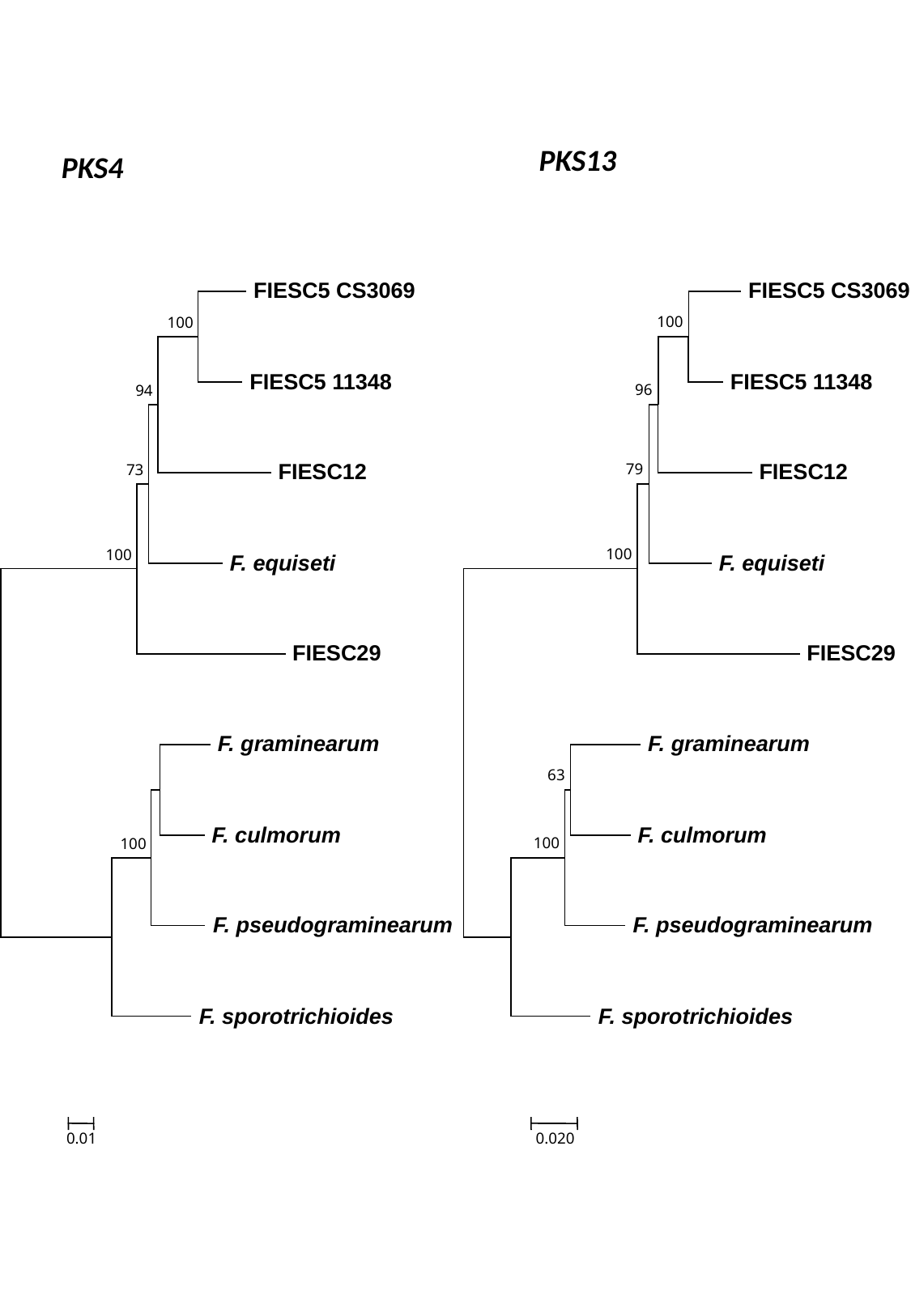

PKS13
PKS4
 FIESC5 CS3069
100
 FIESC5 11348
94
 FIESC12
73
100
 F. equiseti
 FIESC29
 F. graminearum
 F. culmorum
100
 F. pseudograminearum
 F. sporotrichioides
0.01
 FIESC5 CS3069
100
 FIESC5 11348
96
 FIESC12
79
100
 F. equiseti
 FIESC29
 F. graminearum
63
 F. culmorum
100
 F. pseudograminearum
 F. sporotrichioides
0.020

## Slide 21
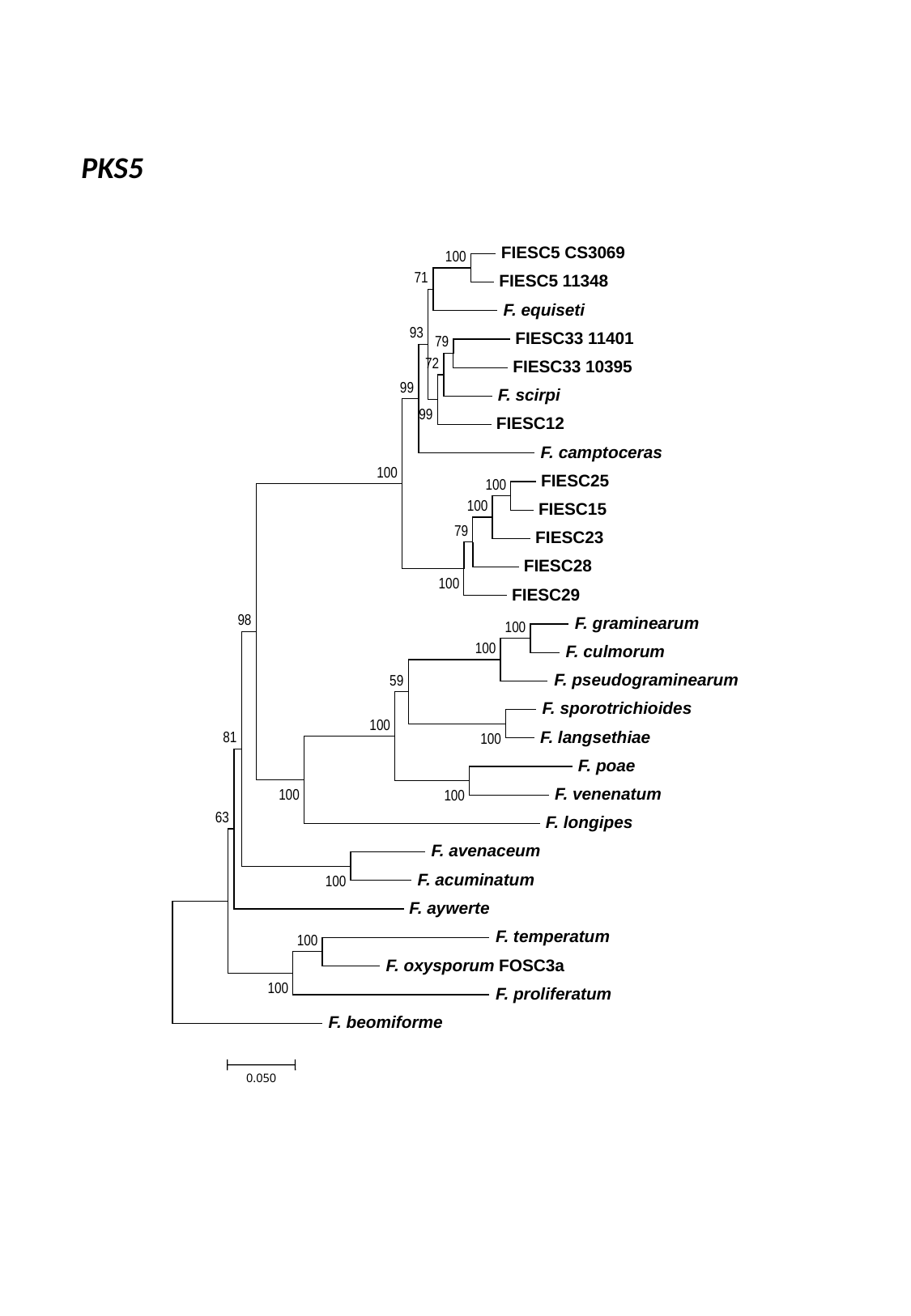

PKS5
 FIESC5 CS3069
100
71
 FIESC5 11348
 F. equiseti
93
 FIESC33 11401
79
72
 FIESC33 10395
99
 F. scirpi
99
 FIESC12
 F. camptoceras
100
 FIESC25
100
100
 FIESC15
79
 FIESC23
 FIESC28
100
 FIESC29
98
 F. graminearum
100
100
 F. culmorum
 F. pseudograminearum
59
 F. sporotrichioides
100
 F. langsethiae
81
100
 F. poae
 F. venenatum
100
100
63
 F. longipes
 F. avenaceum
 F. acuminatum
100
 F. aywerte
 F. temperatum
100
 F. oxysporum FOSC3a
100
 F. proliferatum
 F. beomiforme
0.050

## Slide 22
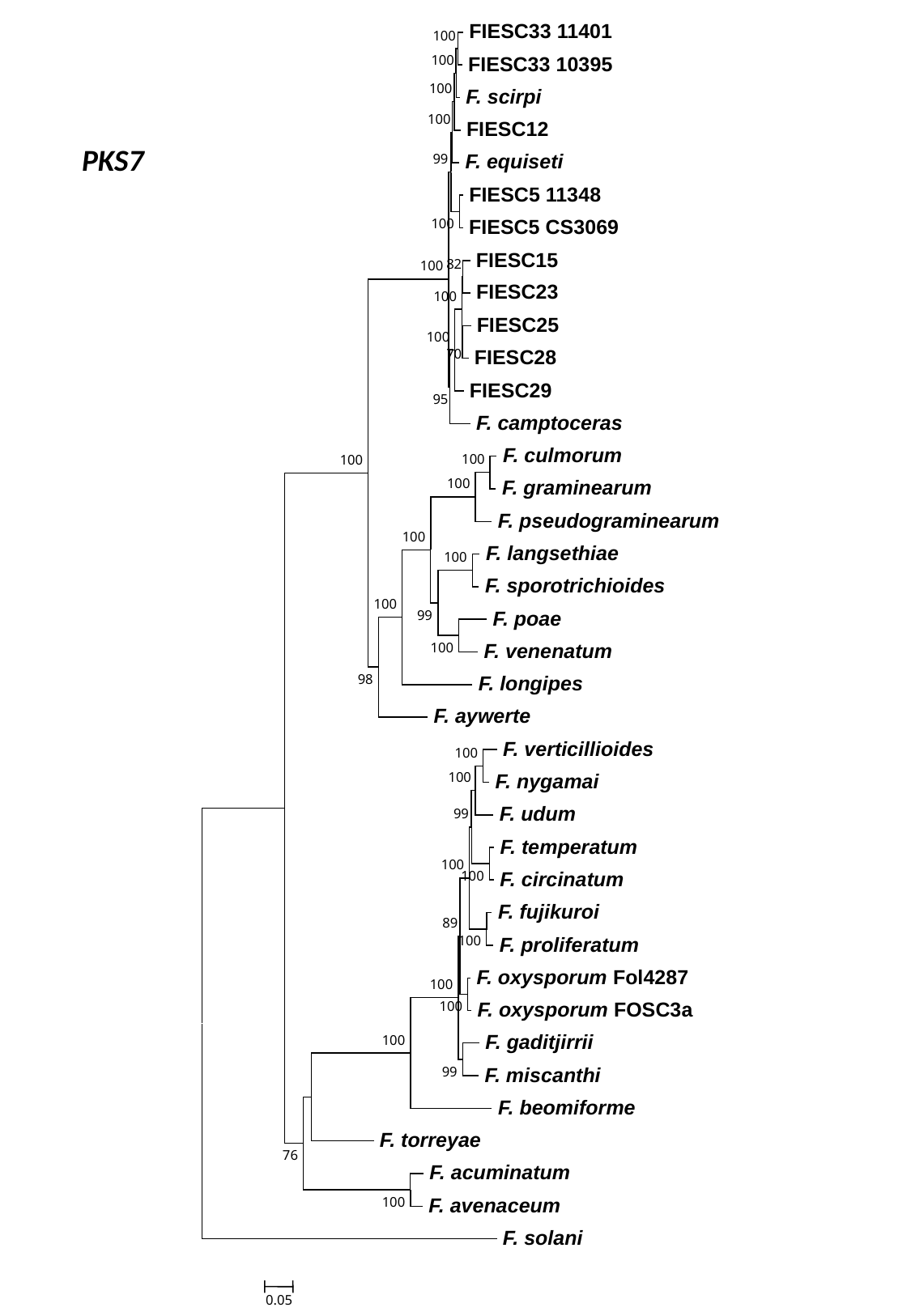

FIESC33 11401
100
 FIESC33 10395
100
100
 F. scirpi
100
 FIESC12
 F. equiseti
99
 FIESC5 11348
 FIESC5 CS3069
100
 FIESC15
82
100
 FIESC23
100
 FIESC25
100
 FIESC28
70
 FIESC29
95
 F. camptoceras
 F. culmorum
100
100
 F. graminearum
100
 F. pseudograminearum
100
 F. langsethiae
100
 F. sporotrichioides
100
 F. poae
99
 F. venenatum
100
 F. longipes
98
 F. aywerte
 F. verticillioides
100
 F. nygamai
100
 F. udum
99
 F. temperatum
100
 F. circinatum
100
 F. fujikuroi
89
 F. proliferatum
100
 F. oxysporum Fol4287
100
 F. oxysporum FOSC3a
100
 F. gaditjirrii
100
 F. miscanthi
99
 F. beomiforme
 F. torreyae
76
 F. acuminatum
 F. avenaceum
100
 F. solani
0.05
PKS7

## Slide 23
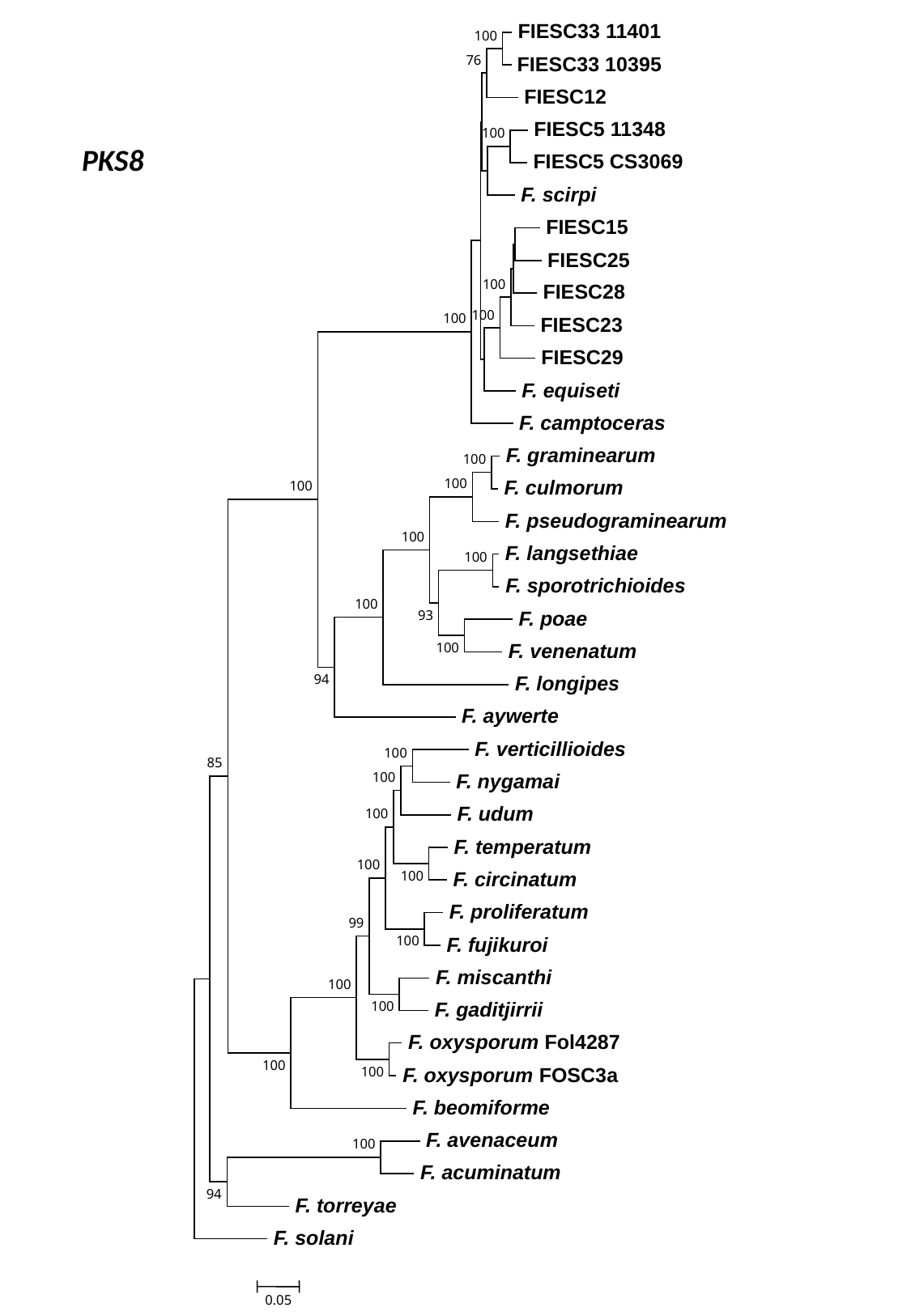

FIESC33 11401
100
 FIESC33 10395
76
 FIESC12
 FIESC5 11348
100
 FIESC5 CS3069
 F. scirpi
 FIESC15
 FIESC25
100
 FIESC28
100
100
 FIESC23
 FIESC29
 F. equiseti
 F. camptoceras
 F. graminearum
100
 F. culmorum
100
100
 F. pseudograminearum
100
 F. langsethiae
100
 F. sporotrichioides
100
 F. poae
93
 F. venenatum
100
 F. longipes
94
 F. aywerte
 F. verticillioides
100
85
 F. nygamai
100
 F. udum
100
 F. temperatum
100
 F. circinatum
100
 F. proliferatum
99
 F. fujikuroi
100
 F. miscanthi
100
 F. gaditjirrii
100
 F. oxysporum Fol4287
100
 F. oxysporum FOSC3a
100
 F. beomiforme
 F. avenaceum
100
 F. acuminatum
94
 F. torreyae
 F. solani
0.05
PKS8

## Slide 24
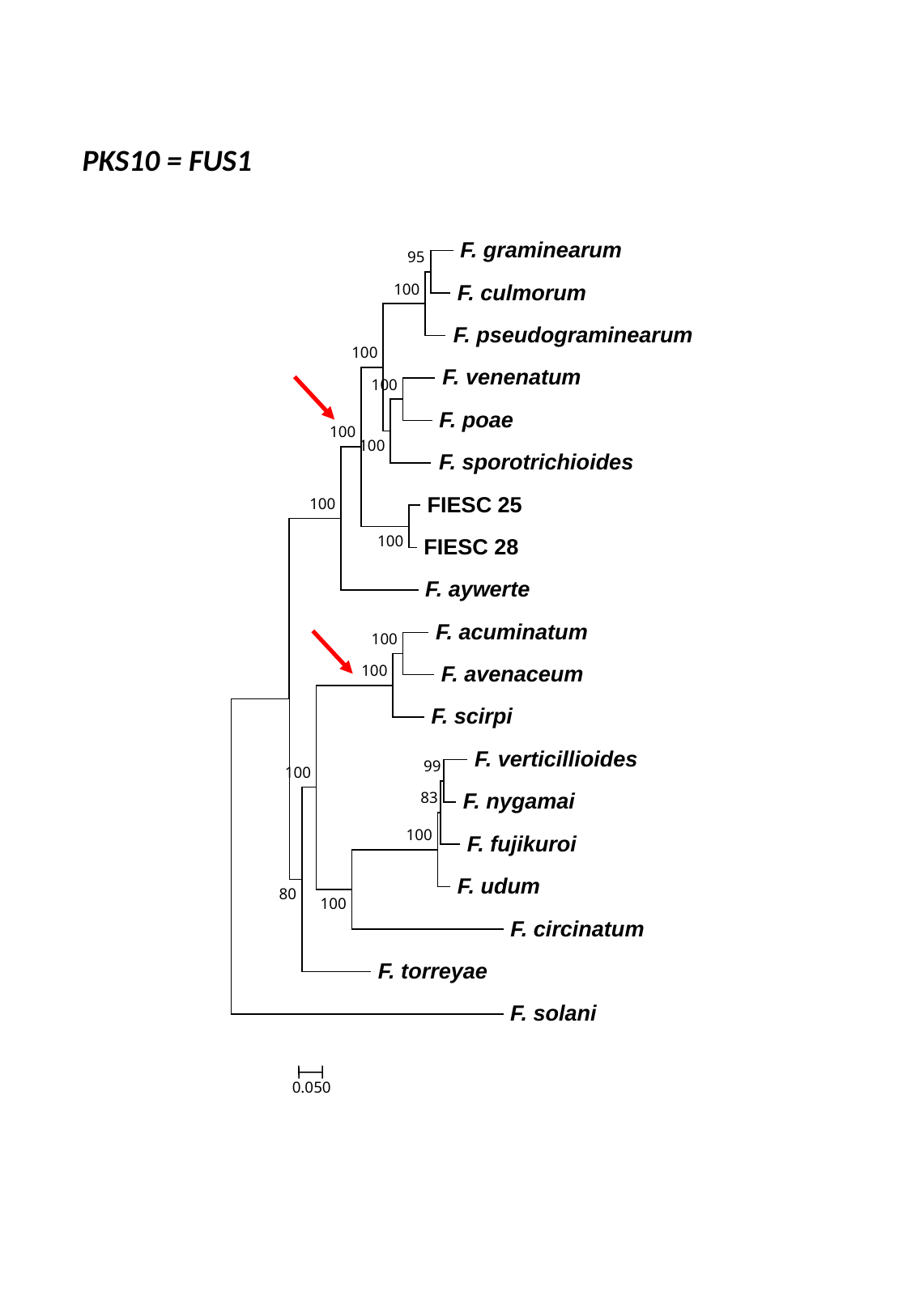

PKS10 = FUS1
 F. graminearum
95
 F. culmorum
100
 F. pseudograminearum
100
 F. venenatum
100
 F. poae
100
100
 F. sporotrichioides
 FIESC 25
100
100
 FIESC 28
 F. aywerte
 F. acuminatum
100
 F. avenaceum
100
 F. scirpi
 F. verticillioides
99
100
 F. nygamai
83
100
 F. fujikuroi
 F. udum
80
100
 F. circinatum
 F. torreyae
 F. solani
0.050

## Slide 25
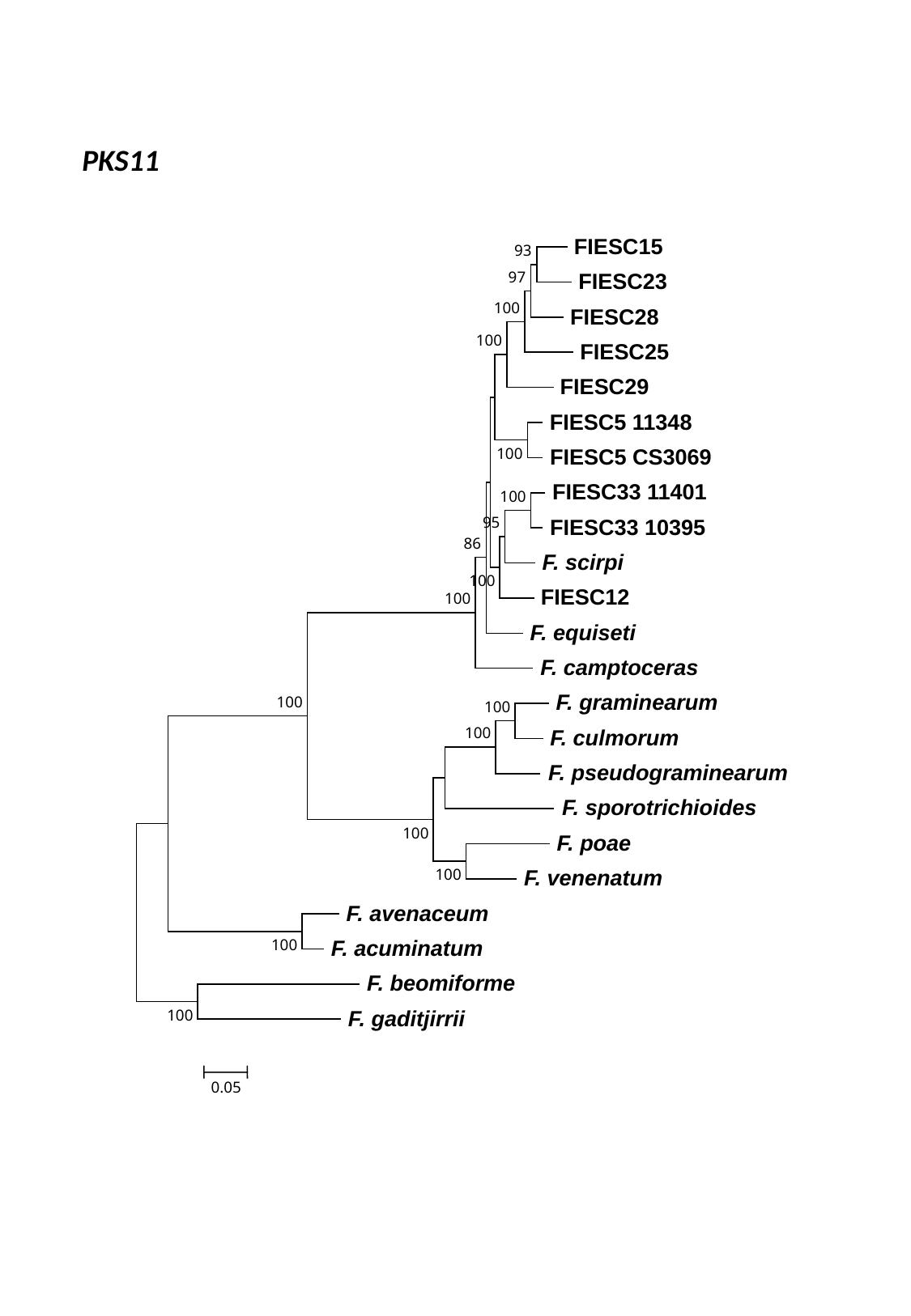

PKS11
 FIESC15
93
97
 FIESC23
100
 FIESC28
100
 FIESC25
 FIESC29
 FIESC5 11348
 FIESC5 CS3069
100
 FIESC33 11401
100
95
 FIESC33 10395
86
 F. scirpi
100
 FIESC12
100
 F. equiseti
 F. camptoceras
 F. graminearum
100
100
100
 F. culmorum
 F. pseudograminearum
 F. sporotrichioides
100
 F. poae
 F. venenatum
100
 F. avenaceum
 F. acuminatum
100
 F. beomiforme
 F. gaditjirrii
100
0.05

## Slide 26
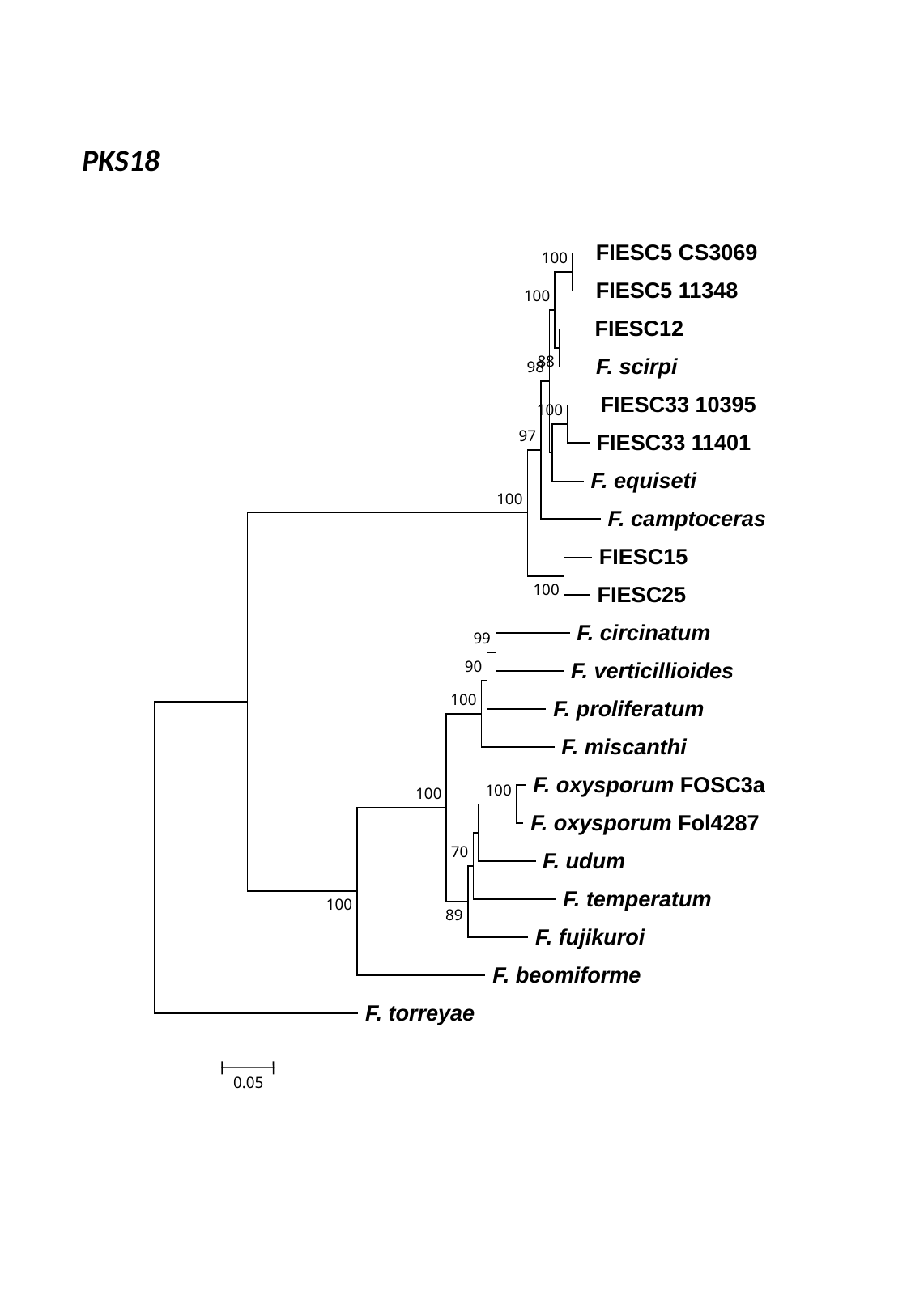

PKS18
 FIESC5 CS3069
100
 FIESC5 11348
100
 FIESC12
88
 F. scirpi
98
 FIESC33 10395
100
97
 FIESC33 11401
 F. equiseti
100
 F. camptoceras
 FIESC15
100
 FIESC25
 F. circinatum
99
 F. verticillioides
90
100
 F. proliferatum
 F. miscanthi
 F. oxysporum FOSC3a
100
100
 F. oxysporum Fol4287
70
 F. udum
 F. temperatum
100
89
 F. fujikuroi
 F. beomiforme
 F. torreyae
0.05

## Slide 27
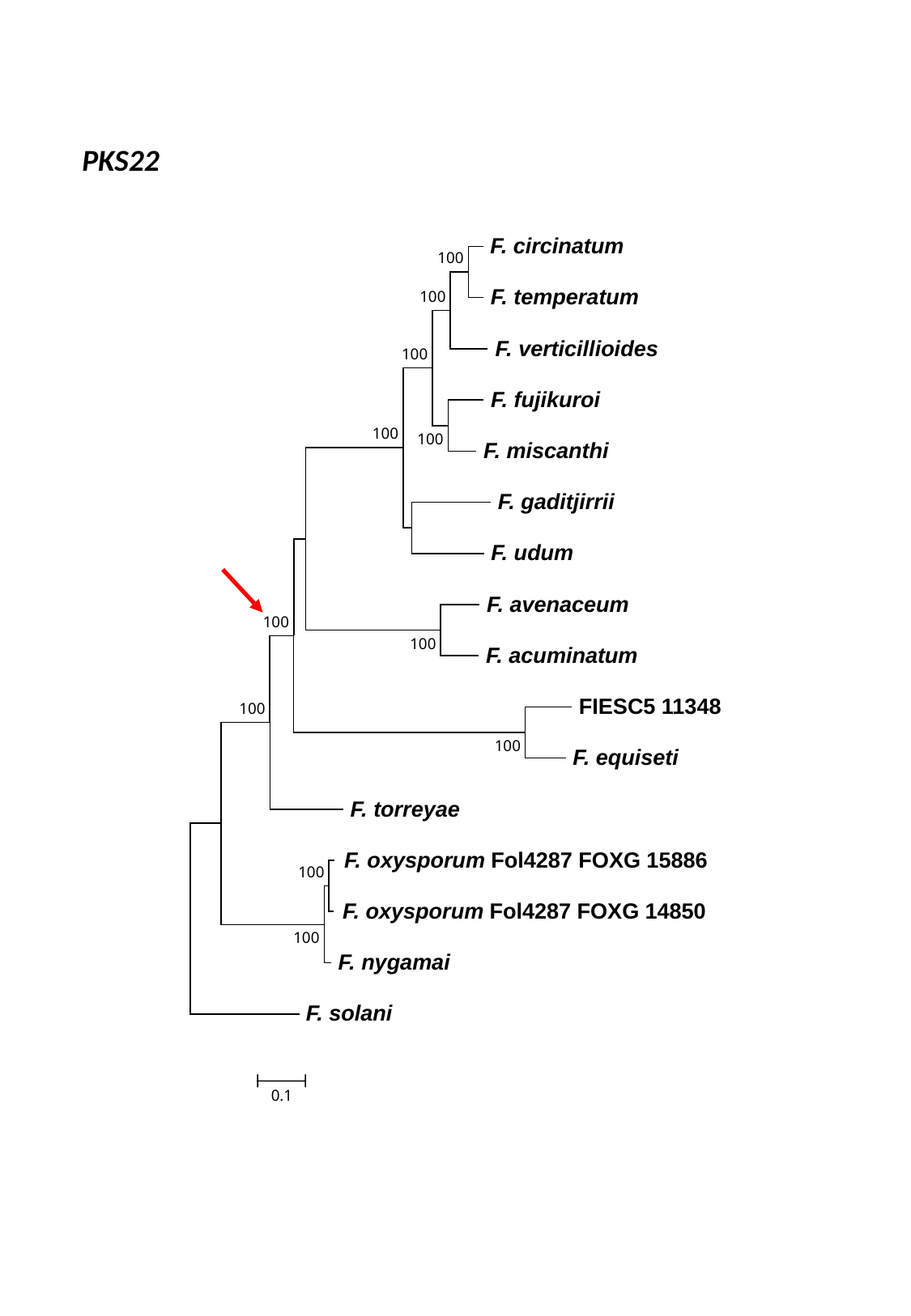

PKS22
 F. circinatum
100
 F. temperatum
100
 F. verticillioides
100
 F. fujikuroi
100
100
 F. miscanthi
 F. gaditjirrii
 F. udum
 F. avenaceum
100
100
 F. acuminatum
 FIESC5 11348
100
100
 F. equiseti
 F. torreyae
 F. oxysporum Fol4287 FOXG 15886
100
 F. oxysporum Fol4287 FOXG 14850
100
 F. nygamai
 F. solani
0.1

## Slide 28
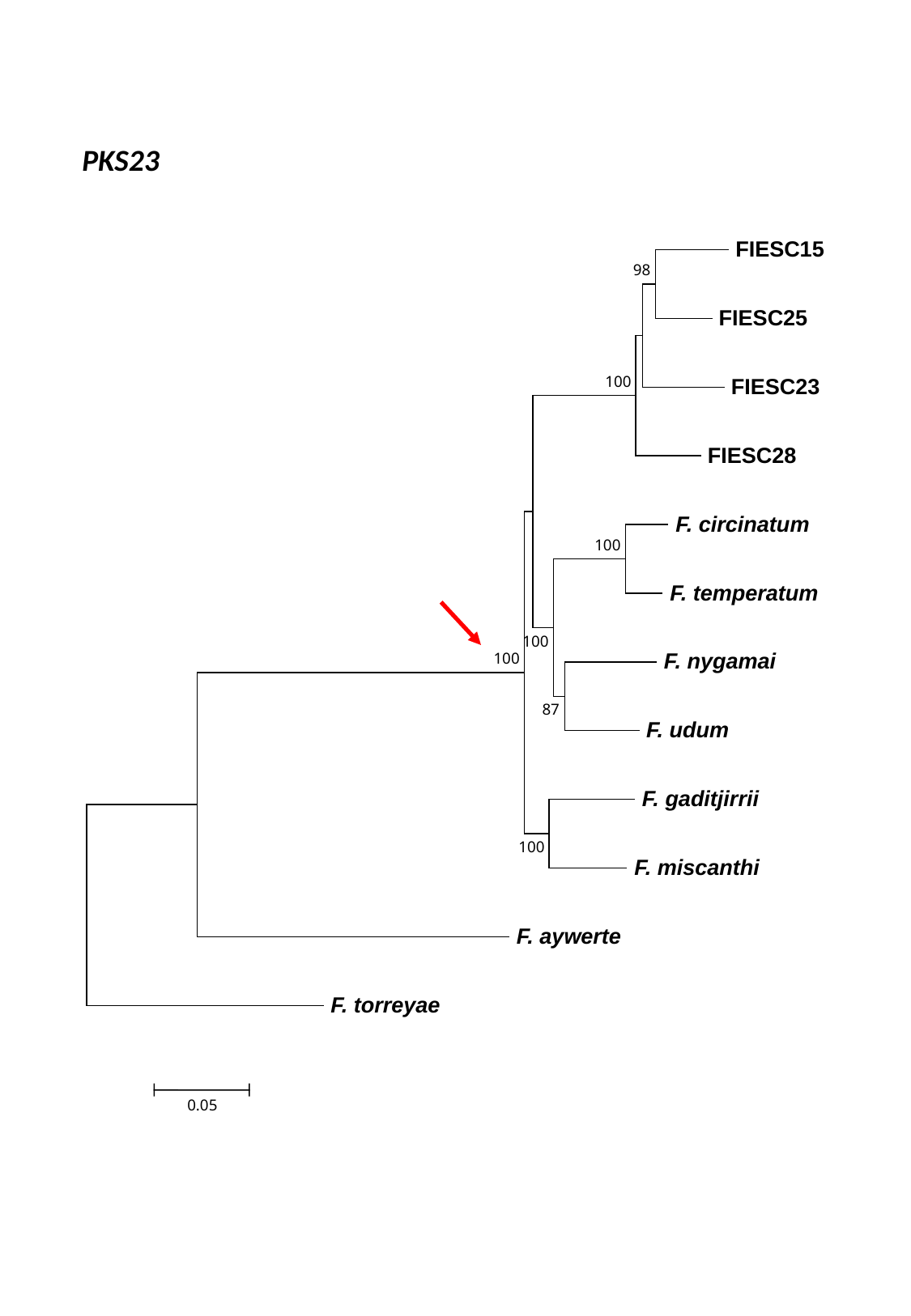

PKS23
 FIESC15
98
 FIESC25
100
 FIESC23
 FIESC28
 F. circinatum
100
 F. temperatum
100
 F. nygamai
100
87
 F. udum
 F. gaditjirrii
100
 F. miscanthi
 F. aywerte
 F. torreyae
0.05

## Slide 29
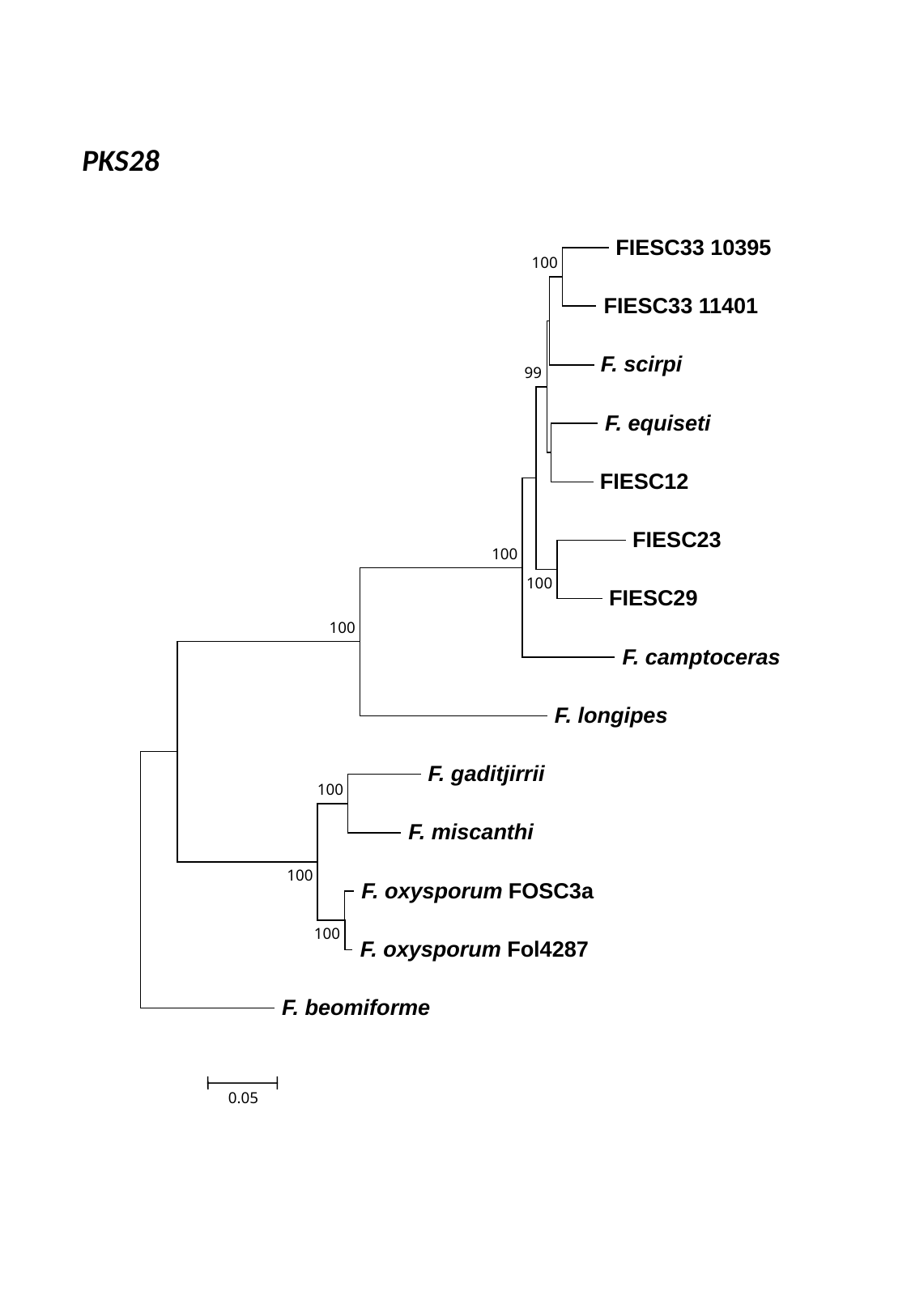

PKS28
 FIESC33 10395
100
 FIESC33 11401
 F. scirpi
99
 F. equiseti
 FIESC12
 FIESC23
100
100
 FIESC29
100
 F. camptoceras
 F. longipes
 F. gaditjirrii
100
 F. miscanthi
100
 F. oxysporum FOSC3a
100
 F. oxysporum Fol4287
 F. beomiforme
0.05

## Slide 30
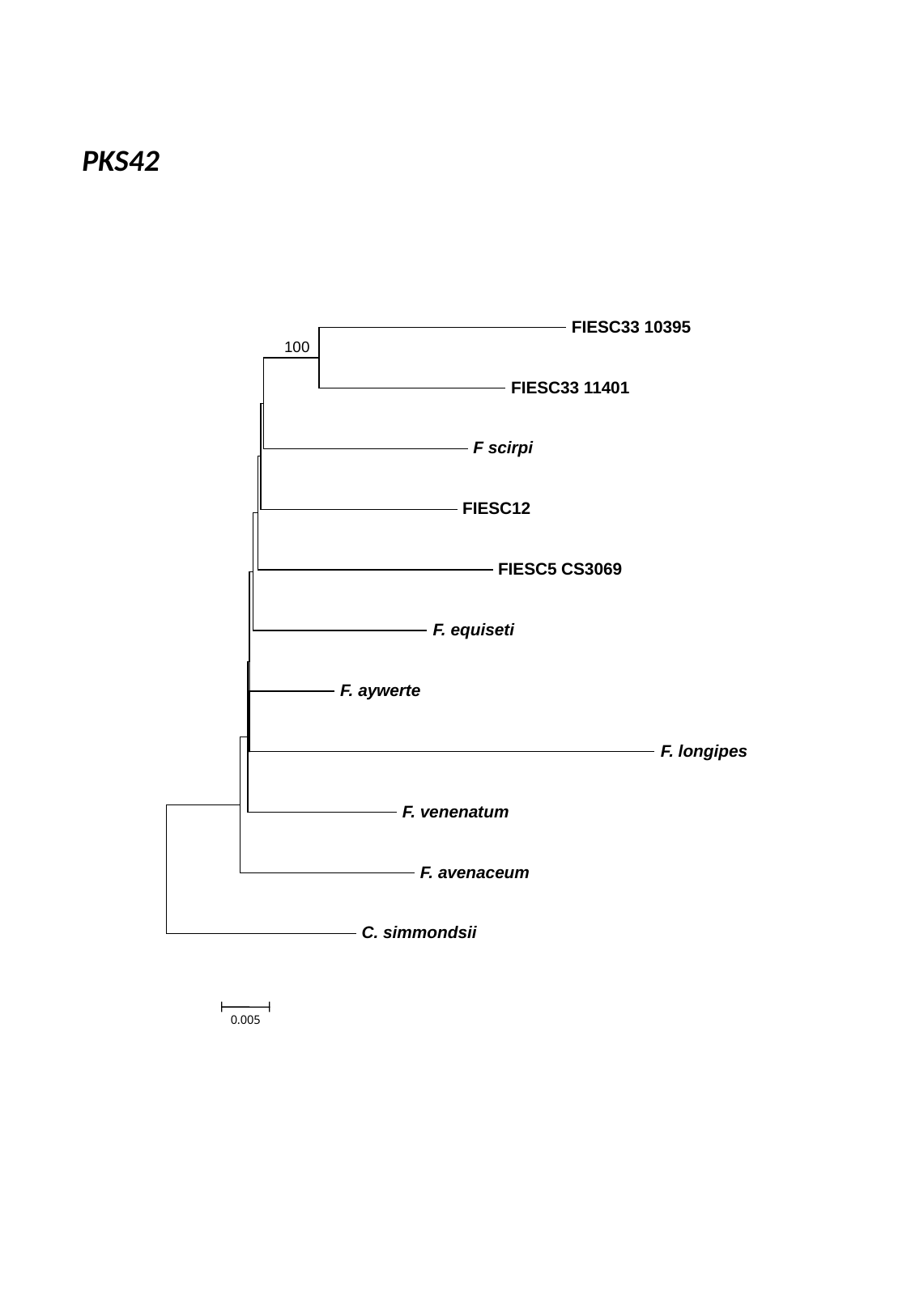

PKS42
 FIESC33 10395
100
 FIESC33 11401
 F scirpi
 FIESC12
 FIESC5 CS3069
 F. equiseti
 F. aywerte
 F. longipes
 F. venenatum
 F. avenaceum
 C. simmondsii
0.005

## Slide 31
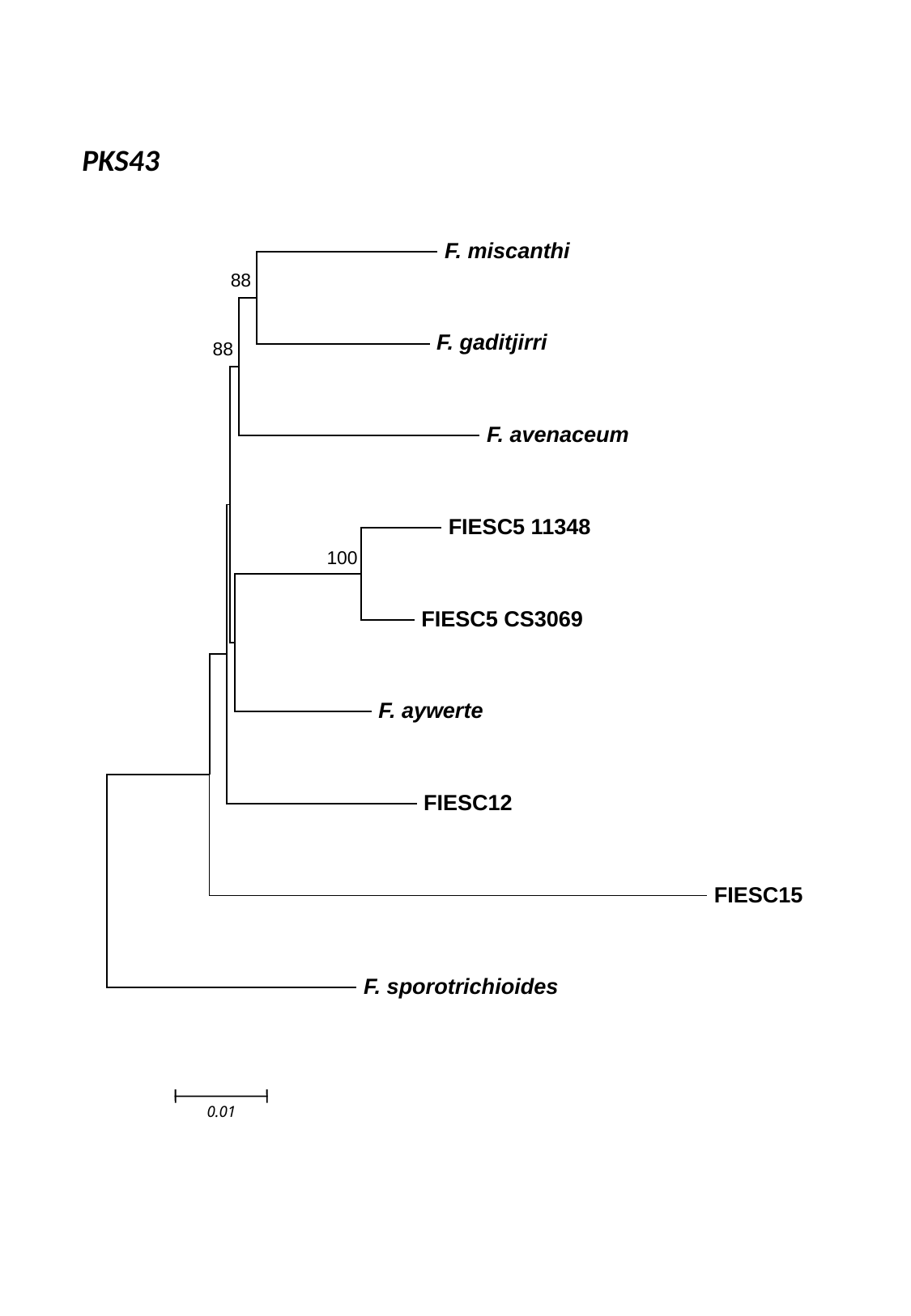

PKS43
 F. miscanthi
88
 F. gaditjirri
88
 F. avenaceum
 FIESC5 11348
100
 FIESC5 CS3069
 F. aywerte
 FIESC12
 FIESC15
 F. sporotrichioides
0.01

## Slide 32
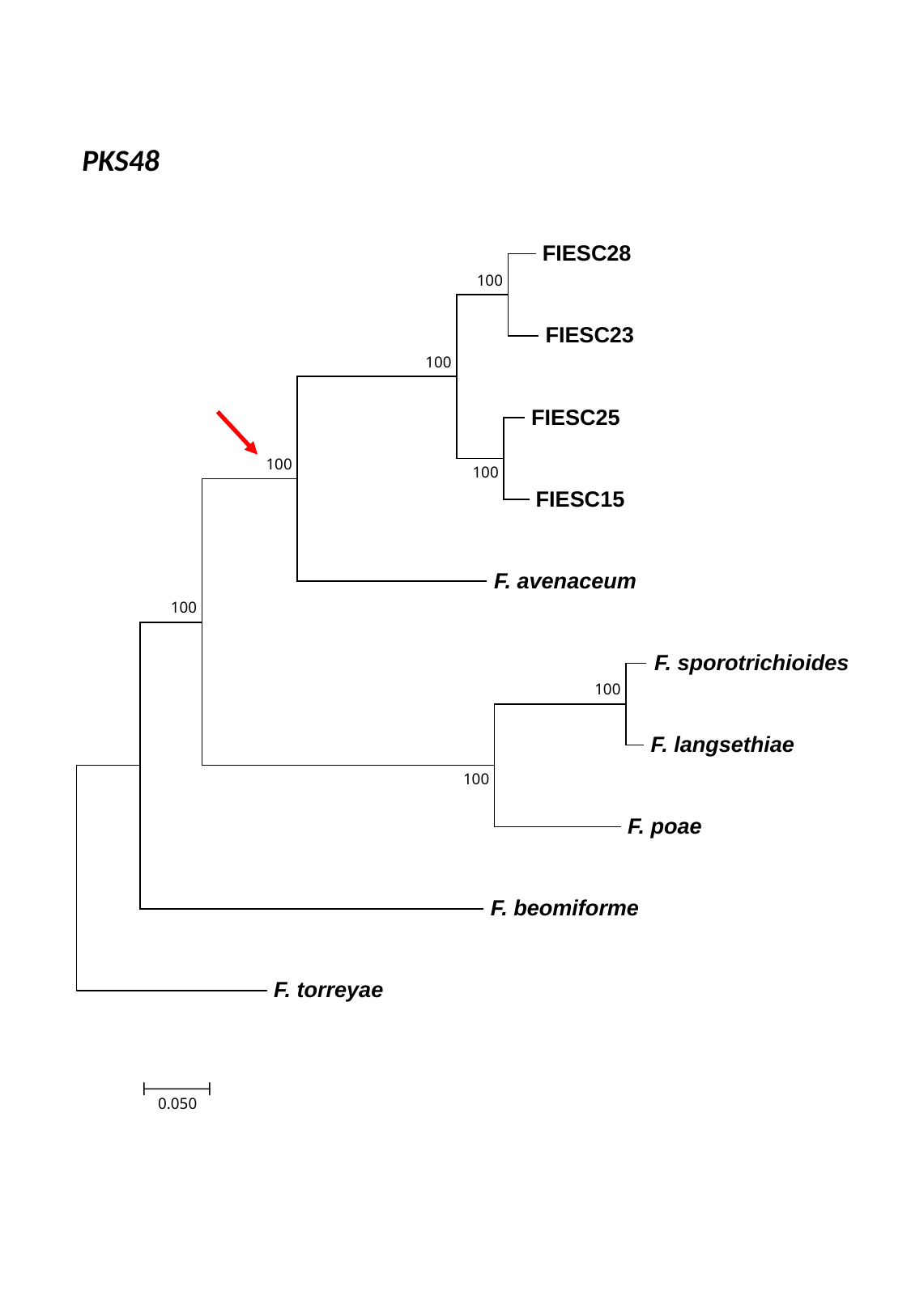

PKS48
 FIESC28
100
 FIESC23
100
 FIESC25
100
100
 FIESC15
 F. avenaceum
100
 F. sporotrichioides
100
 F. langsethiae
100
 F. poae
 F. beomiforme
 F. torreyae
0.050

## Slide 33
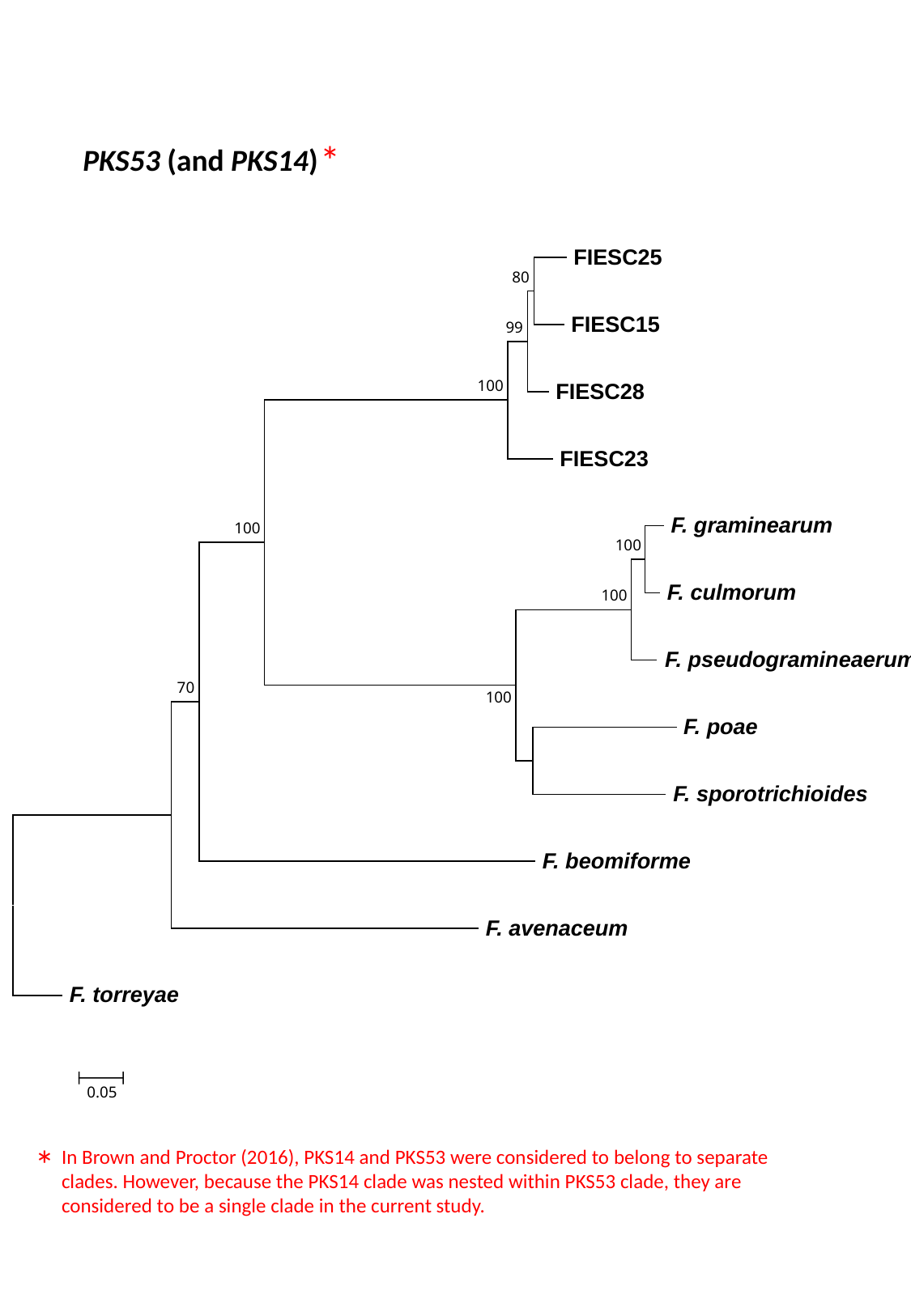

*
PKS53 (and PKS14)
 FIESC25
80
 FIESC15
99
100
 FIESC28
 FIESC23
 F. graminearum
100
100
 F. culmorum
100
 F. pseudogramineaerum
70
100
 F. poae
 F. sporotrichioides
 F. beomiforme
 F. avenaceum
 F. torreyae
0.05
*
In Brown and Proctor (2016), PKS14 and PKS53 were considered to belong to separate clades. However, because the PKS14 clade was nested within PKS53 clade, they are considered to be a single clade in the current study.

## Slide 34
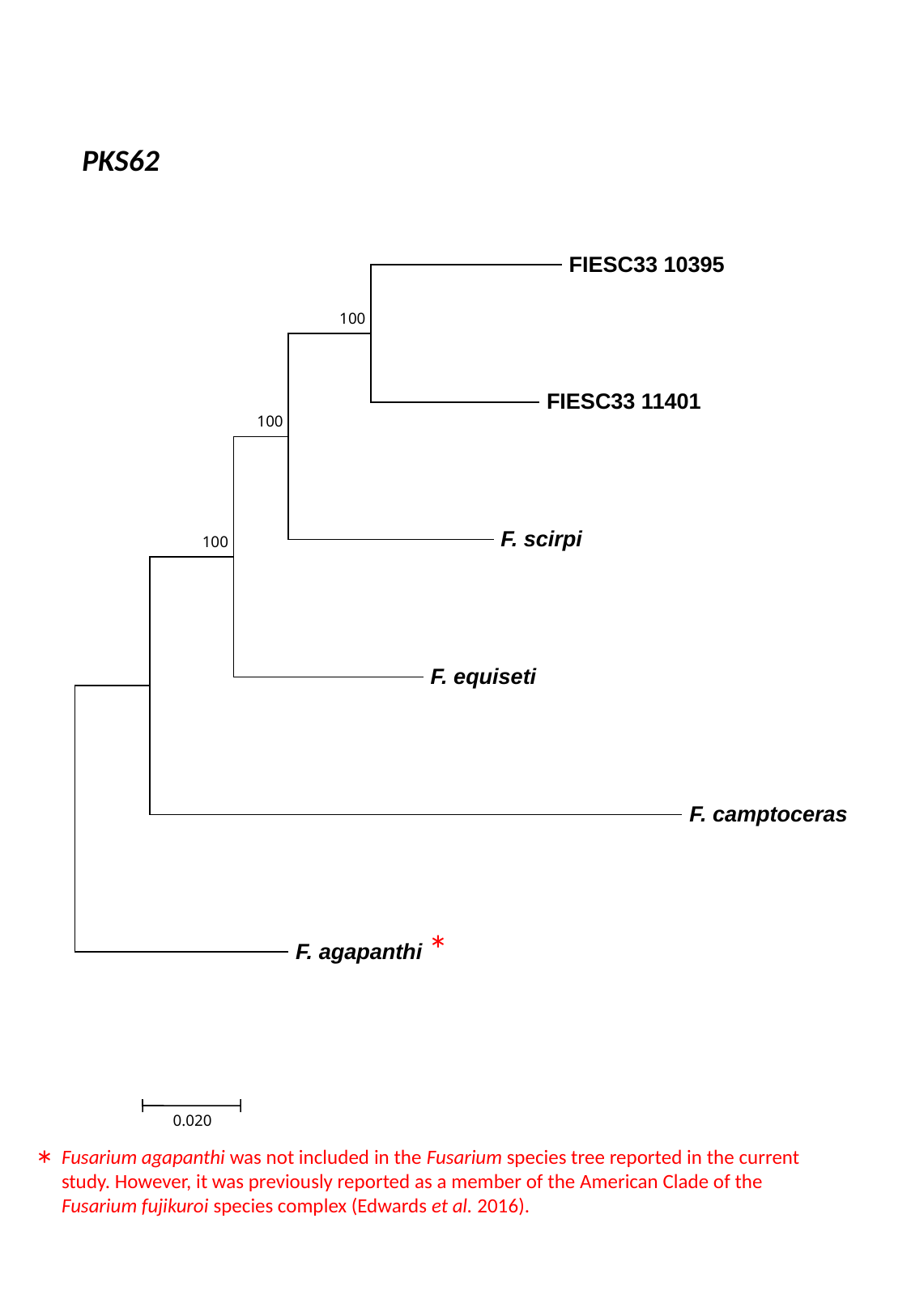

PKS62
 FIESC33 10395
100
 FIESC33 11401
100
 F. scirpi
100
 F. equiseti
 F. camptoceras
 F. agapanthi
0.020
*
*
Fusarium agapanthi was not included in the Fusarium species tree reported in the current study. However, it was previously reported as a member of the American Clade of the Fusarium fujikuroi species complex (Edwards et al. 2016).

## Slide 35
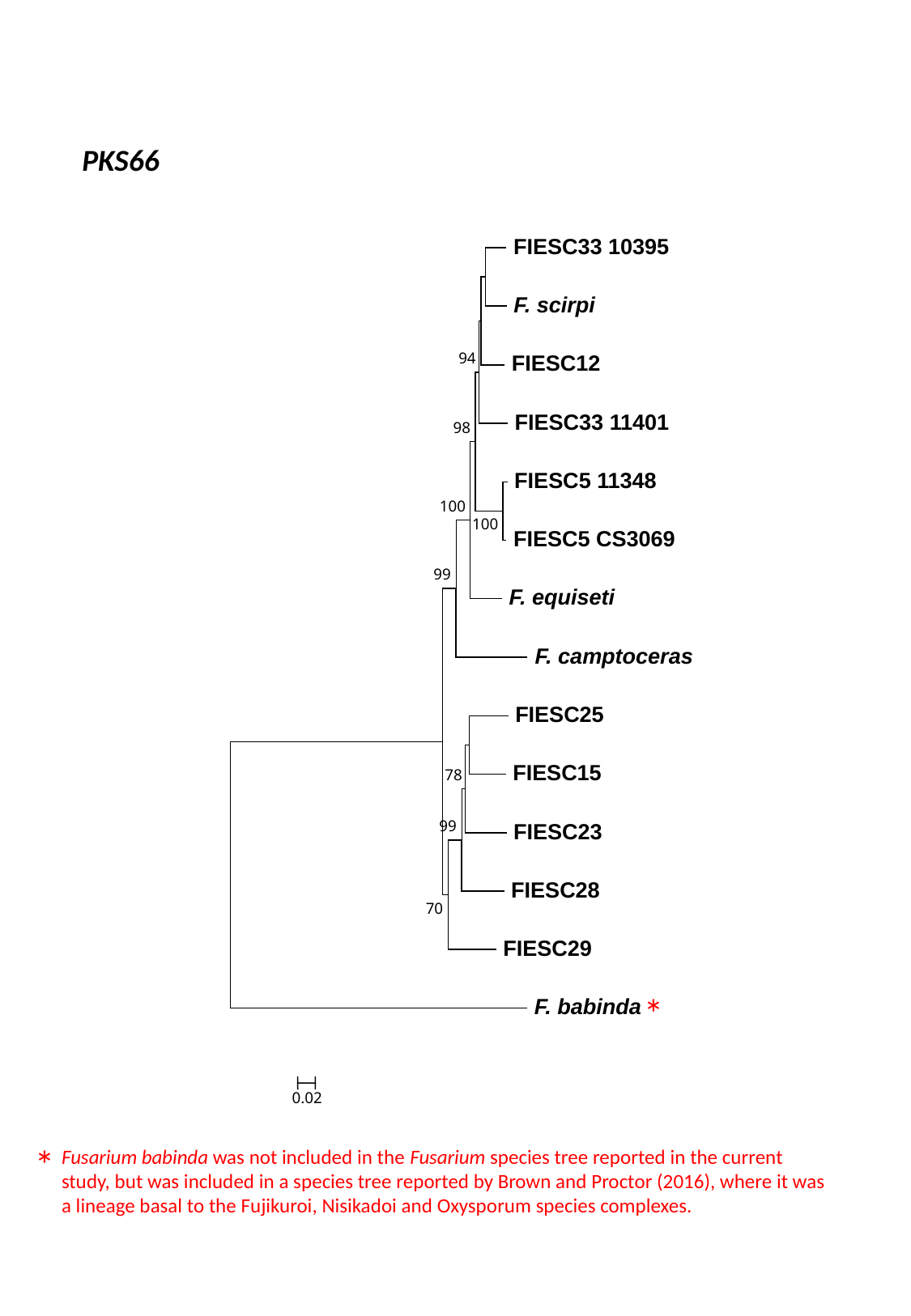

PKS66
 FIESC33 10395
 F. scirpi
94
 FIESC12
 FIESC33 11401
98
 FIESC5 11348
100
100
 FIESC5 CS3069
99
 F. equiseti
 F. camptoceras
 FIESC25
 FIESC15
78
99
 FIESC23
 FIESC28
70
 FIESC29
 F. babinda
0.02
*
*
Fusarium babinda was not included in the Fusarium species tree reported in the current study, but was included in a species tree reported by Brown and Proctor (2016), where it was a lineage basal to the Fujikuroi, Nisikadoi and Oxysporum species complexes.

## Slide 36
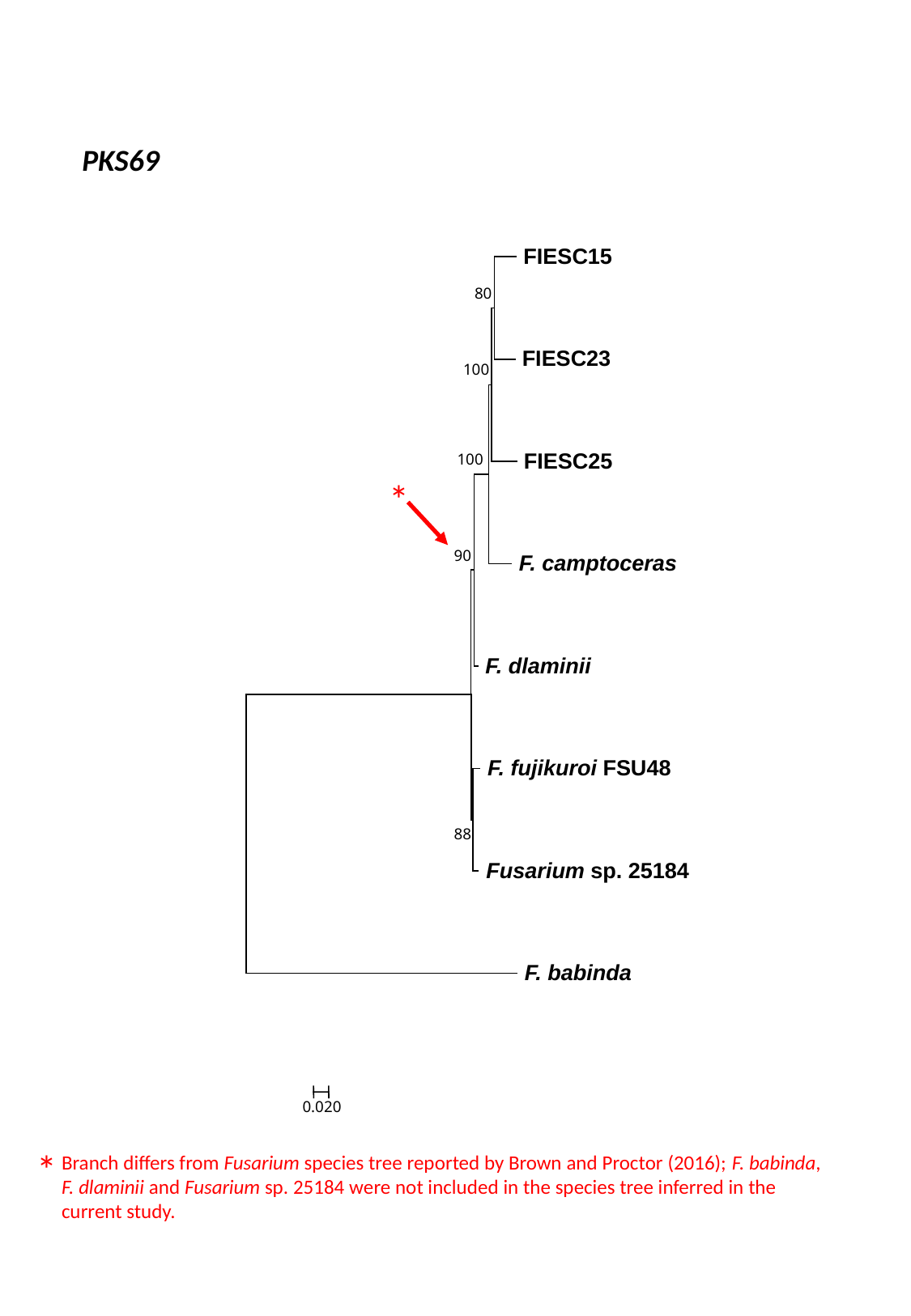

PKS69
 FIESC15
80
 FIESC23
100
 FIESC25
100
90
 F. camptoceras
 F. dlaminii
 F. fujikuroi FSU48
88
 Fusarium sp. 25184
 F. babinda
0.020
*
*
Branch differs from Fusarium species tree reported by Brown and Proctor (2016); F. babinda, F. dlaminii and Fusarium sp. 25184 were not included in the species tree inferred in the current study.

## Slide 37
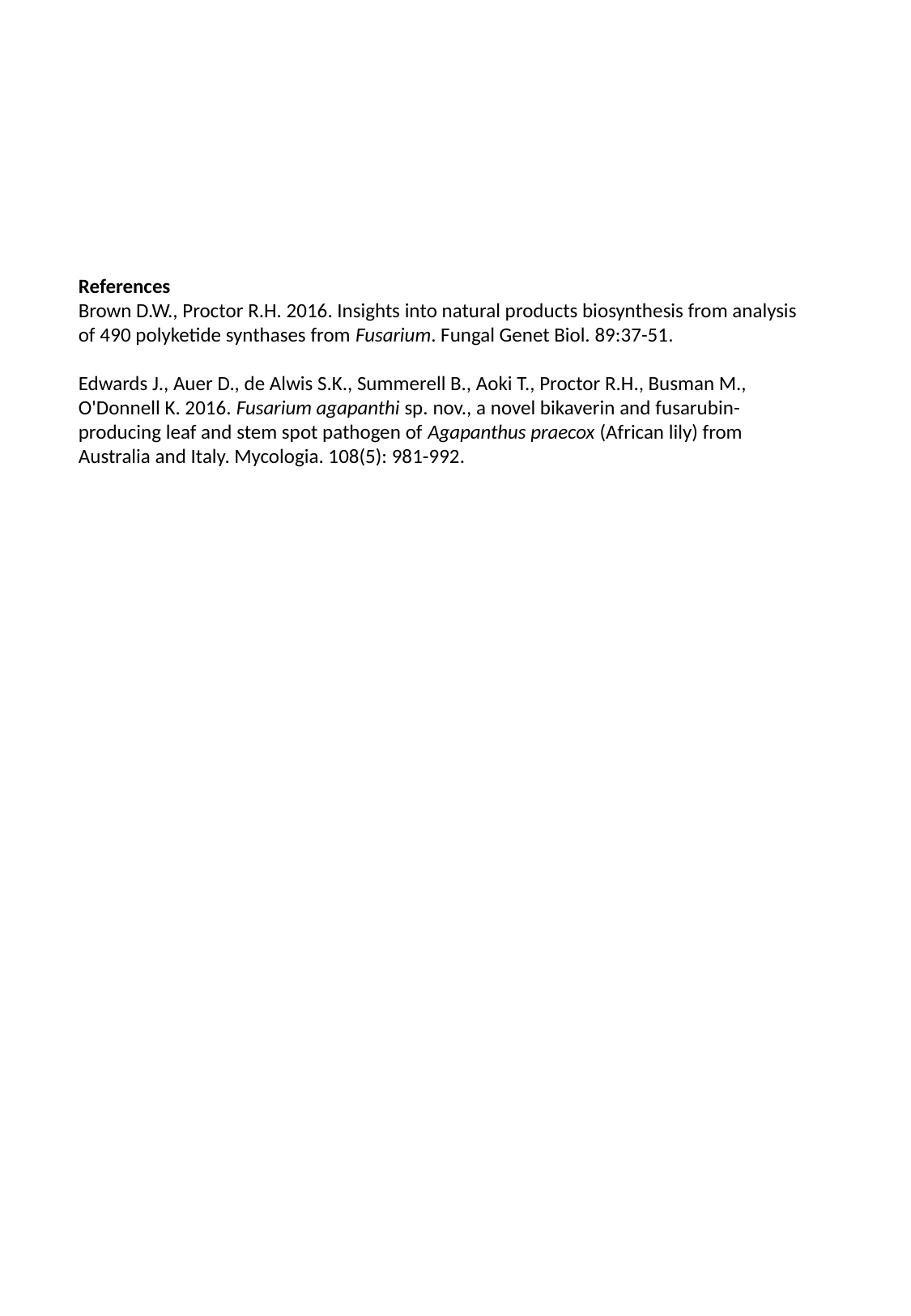

References
Brown D.W., Proctor R.H. 2016. Insights into natural products biosynthesis from analysis of 490 polyketide synthases from Fusarium. Fungal Genet Biol. 89:37-51.
Edwards J., Auer D., de Alwis S.K., Summerell B., Aoki T., Proctor R.H., Busman M., O'Donnell K. 2016. Fusarium agapanthi sp. nov., a novel bikaverin and fusarubin-producing leaf and stem spot pathogen of Agapanthus praecox (African lily) from Australia and Italy. Mycologia. 108(5): 981-992.
