# Supplementary material for: Variation in secondary metabolite production potential in the Fusarium incarnatum-equiseti species complex revealed by comparative analysis of 13 genomes
Source: BMC Genomics. 2019 Apr 24;20:314. doi: 10.1186/s12864-019-5567-7 (PMC6480918; doi:10.1186/s12864-019-5567-7)
Supplement: Supplementary file 6 — Reconciled NRPS and PKS trees with duplication, losses and HGT events as obtained from NOTUNG. Duplications are indicated with red D, transfers are indicated by yellow T’s, and losses in grey. (PPTX 303 kb) [file 12864_2019_5567_MOESM6_ESM.pptx]

## Slide 1
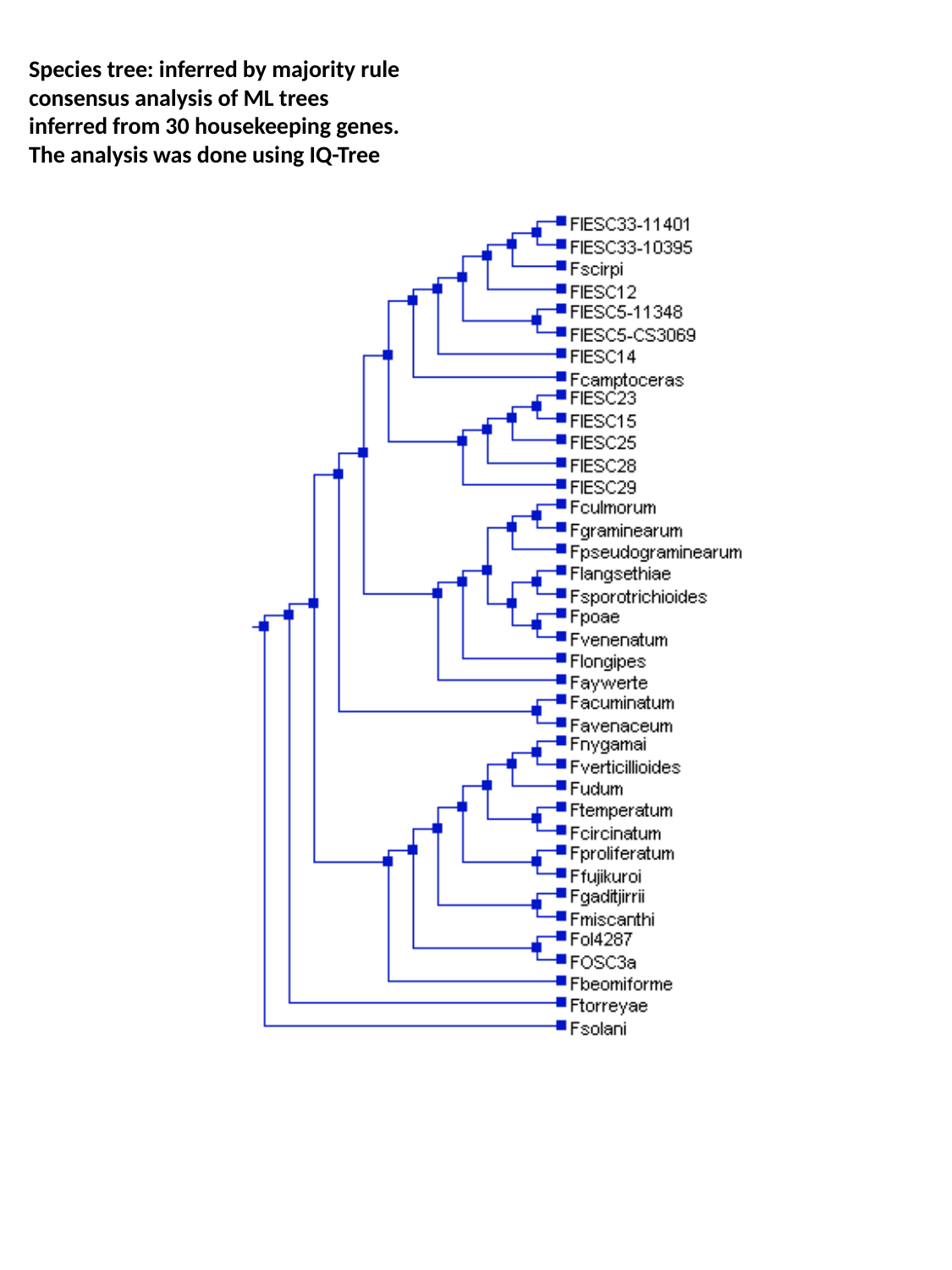

Species tree: inferred by majority rule consensus analysis of ML trees inferred from 30 housekeeping genes. The analysis was done using IQ-Tree

## Slide 2
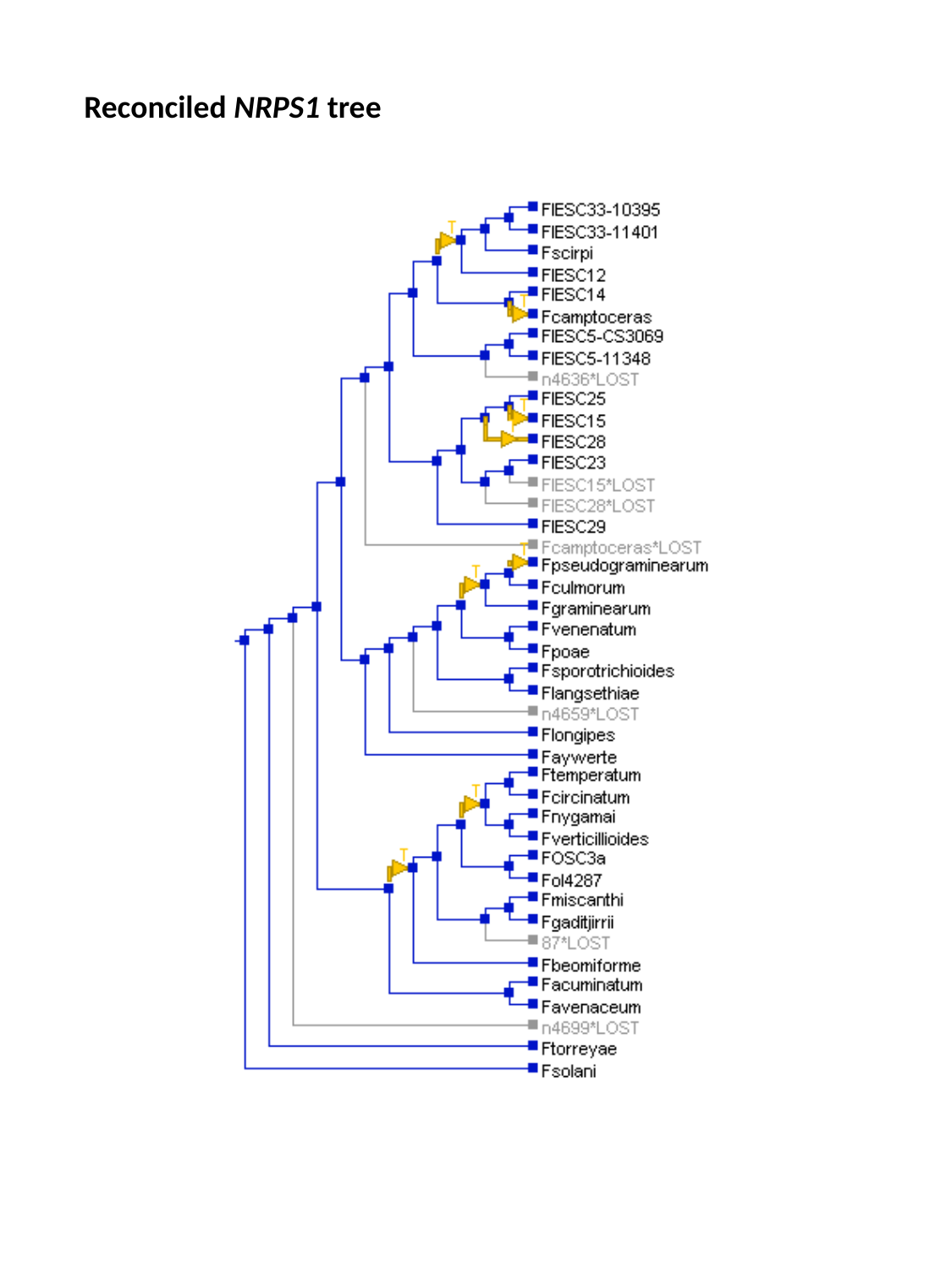

Reconciled NRPS1 tree

## Slide 3
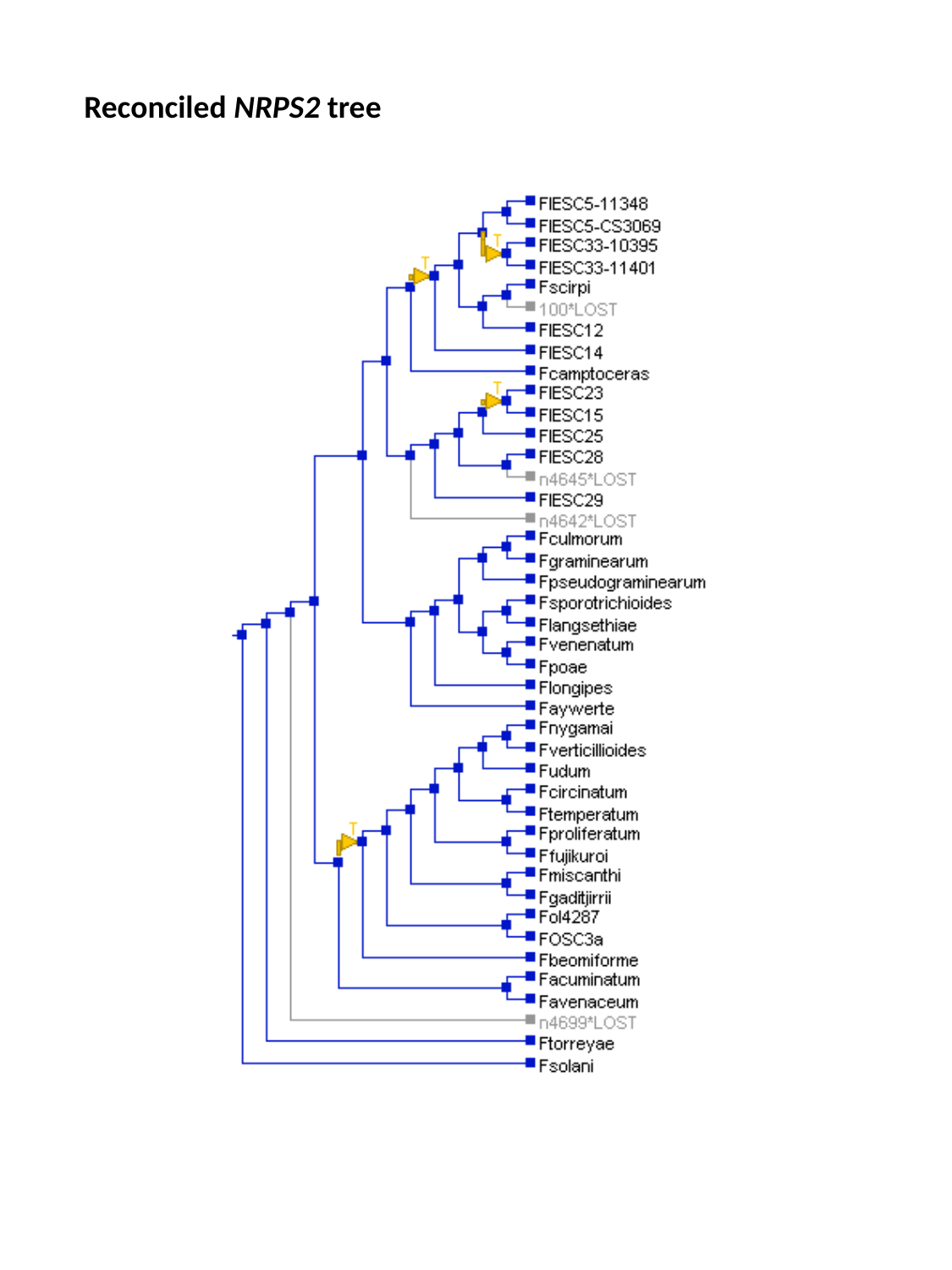

Reconciled NRPS2 tree

## Slide 4
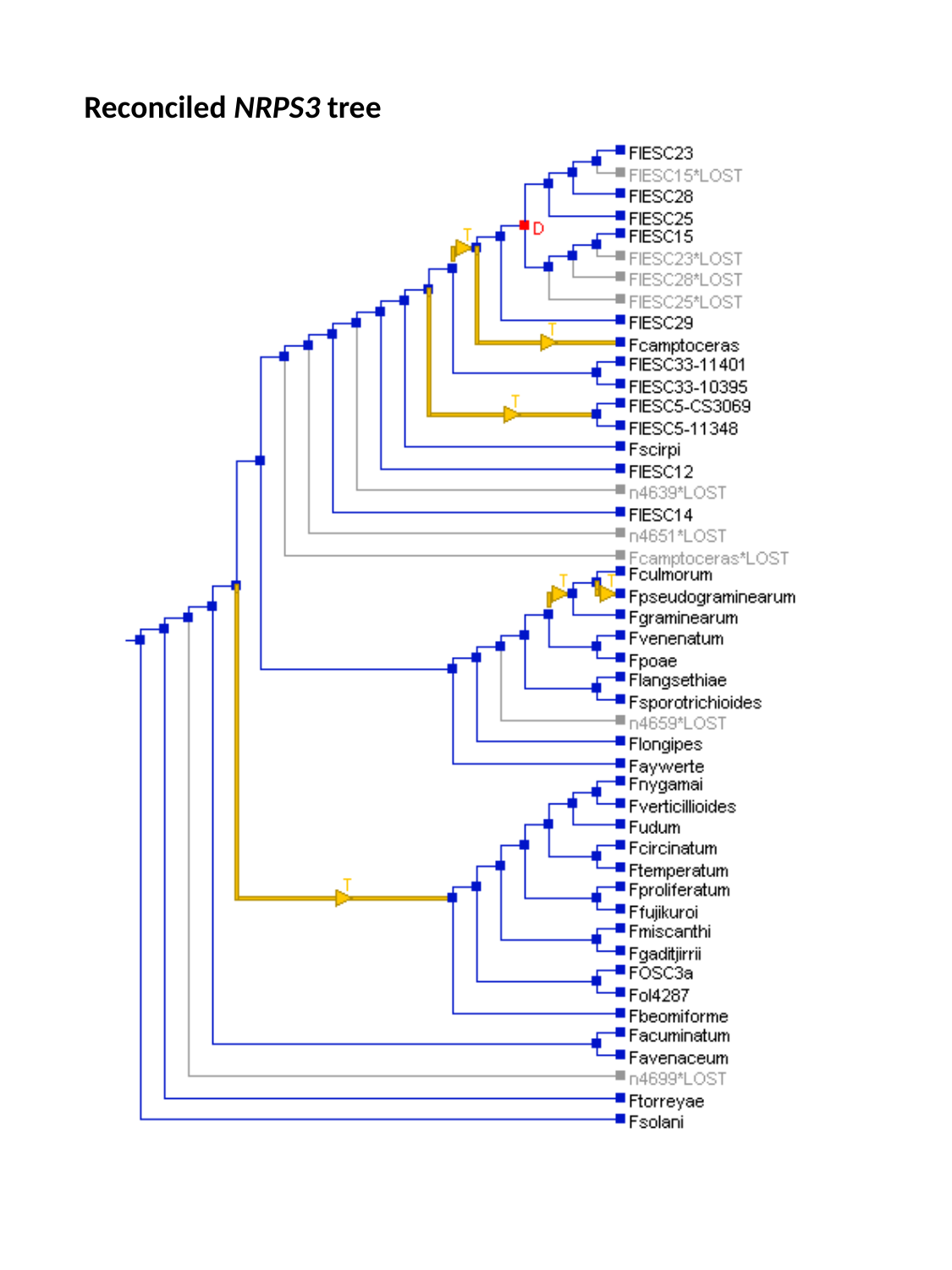

Reconciled NRPS3 tree

## Slide 5
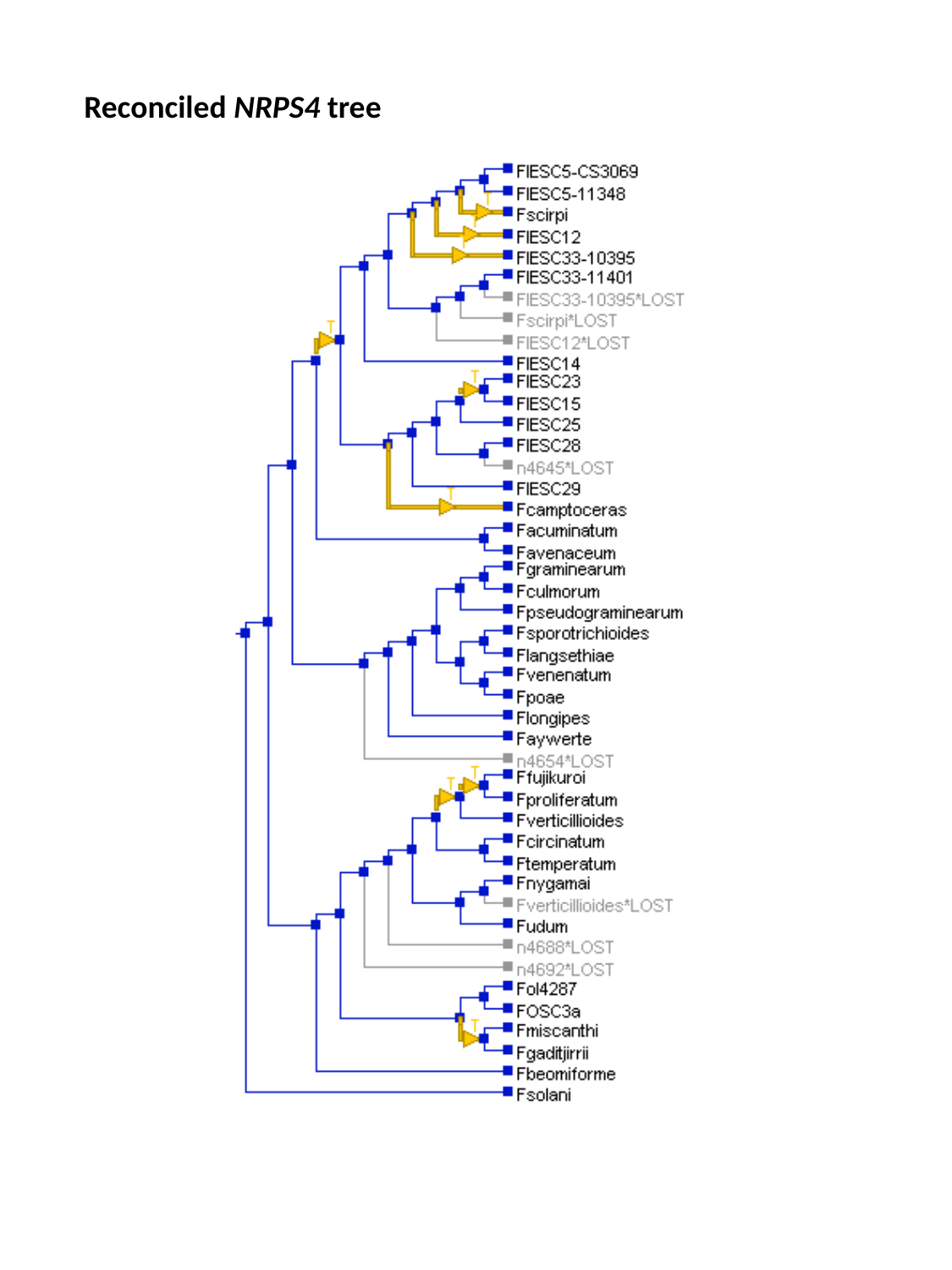

Reconciled NRPS4 tree

## Slide 6
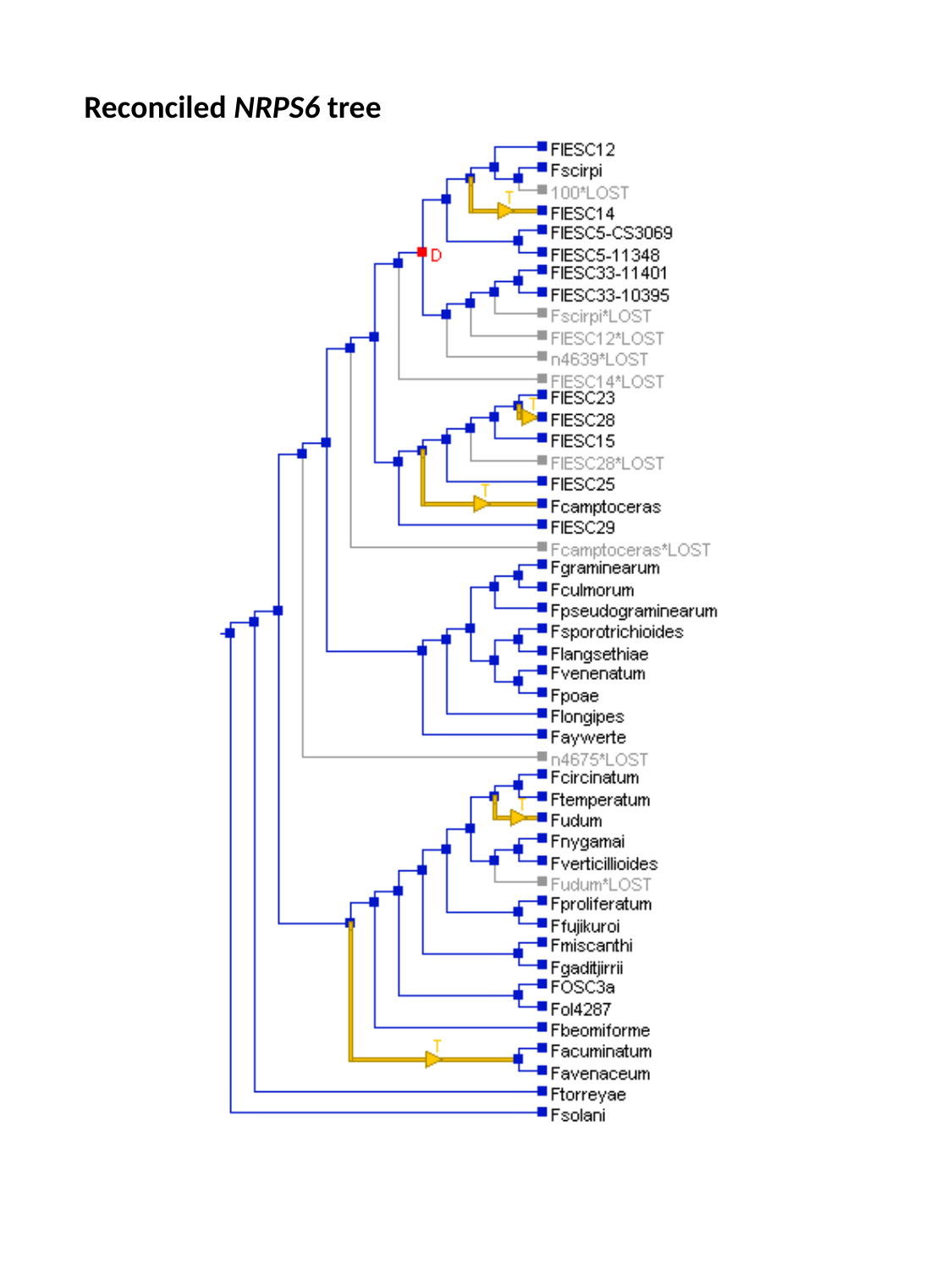

Reconciled NRPS6 tree

## Slide 7
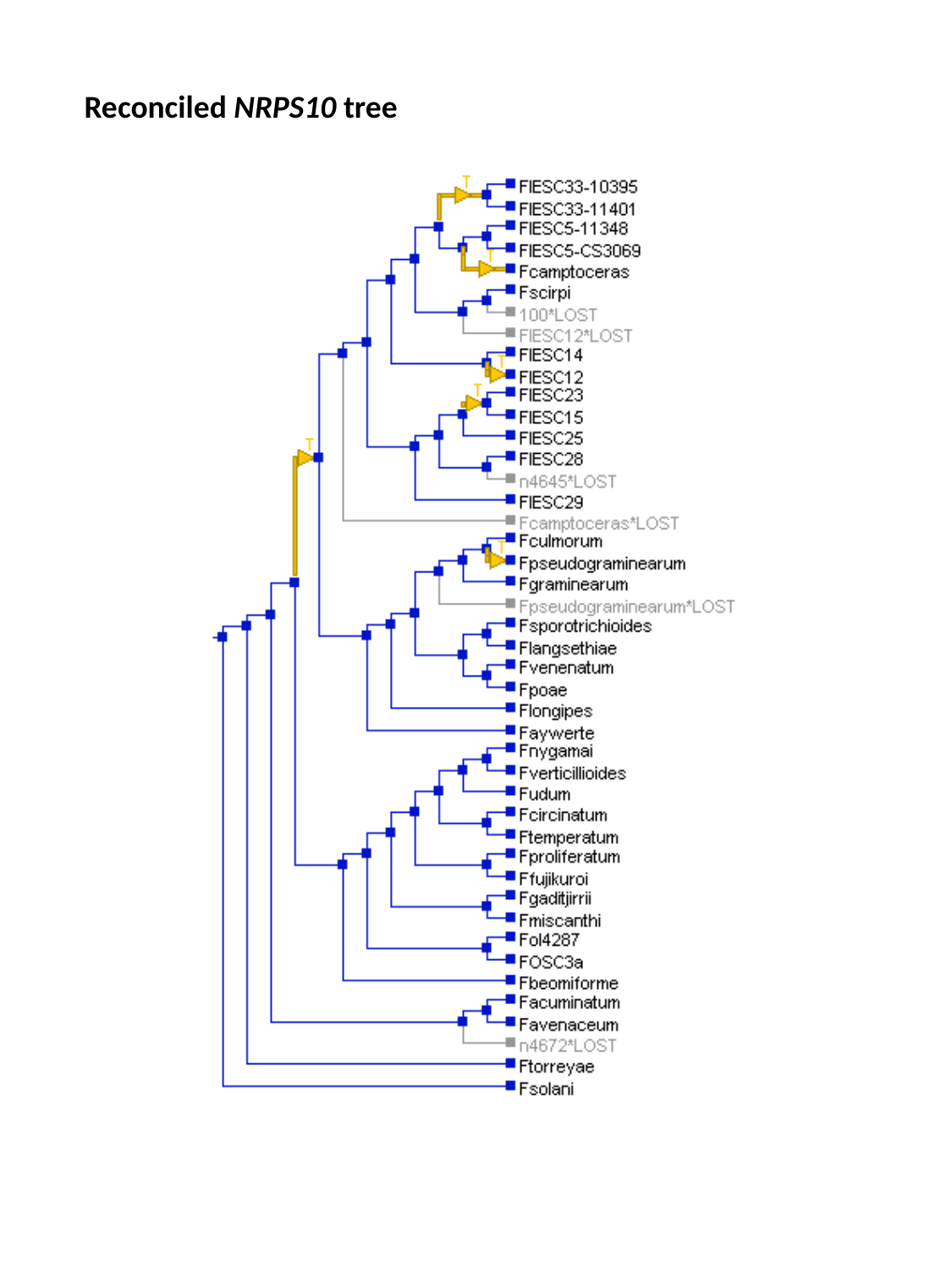

Reconciled NRPS10 tree

## Slide 8
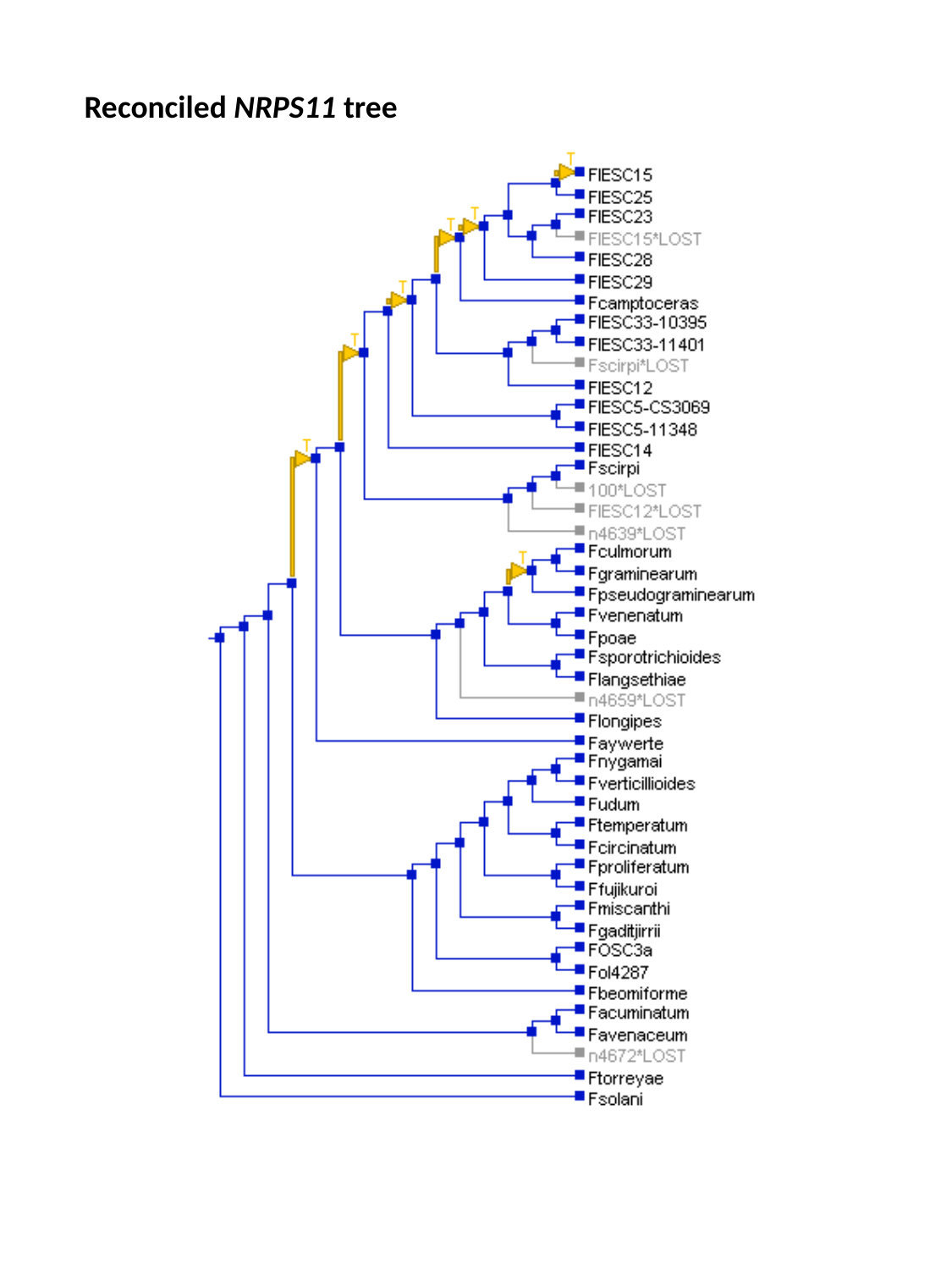

Reconciled NRPS11 tree

## Slide 9
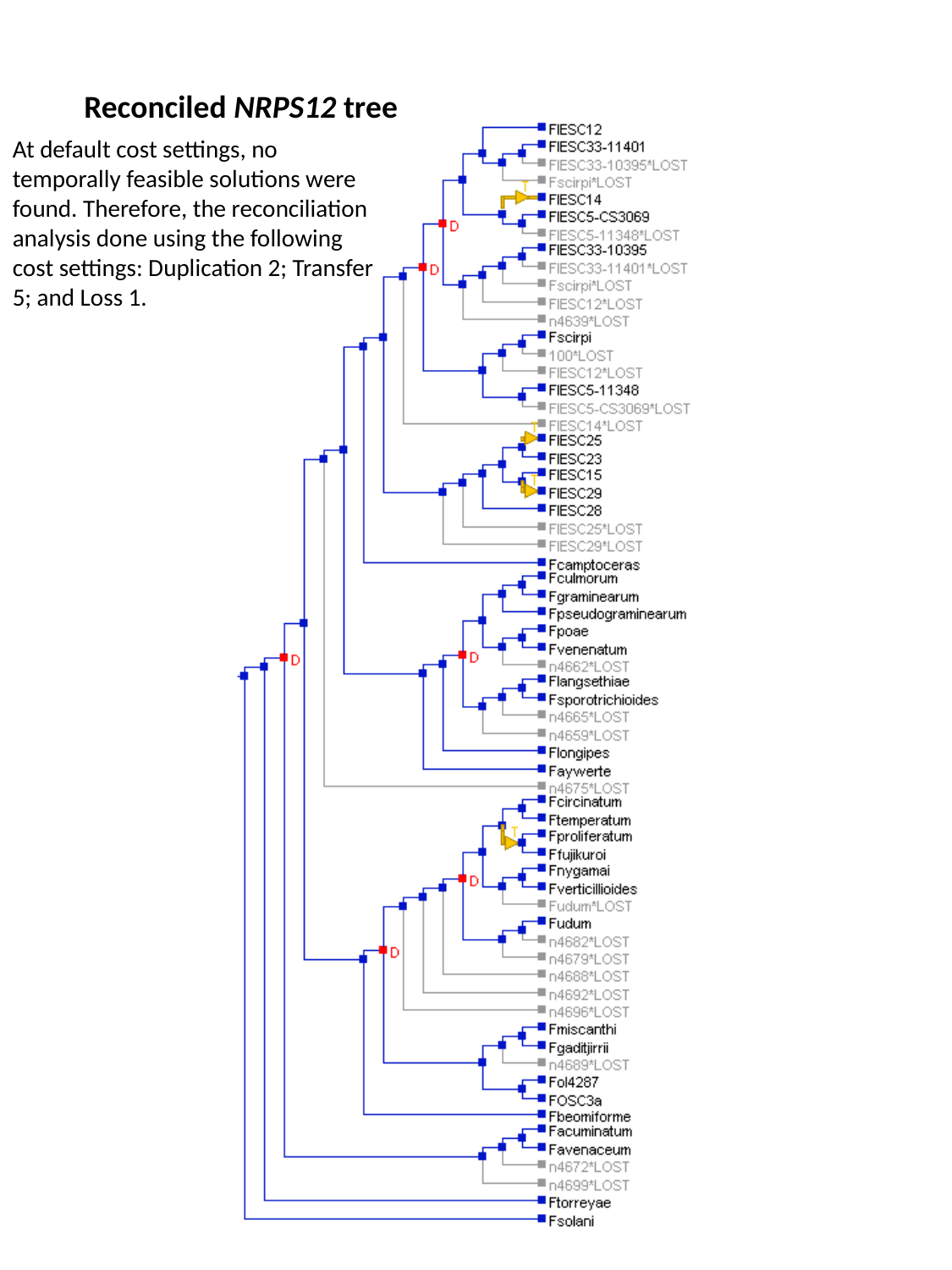

Reconciled NRPS12 tree
At default cost settings, no temporally feasible solutions were found. Therefore, the reconciliation analysis done using the following cost settings: Duplication 2; Transfer 5; and Loss 1.

## Slide 10
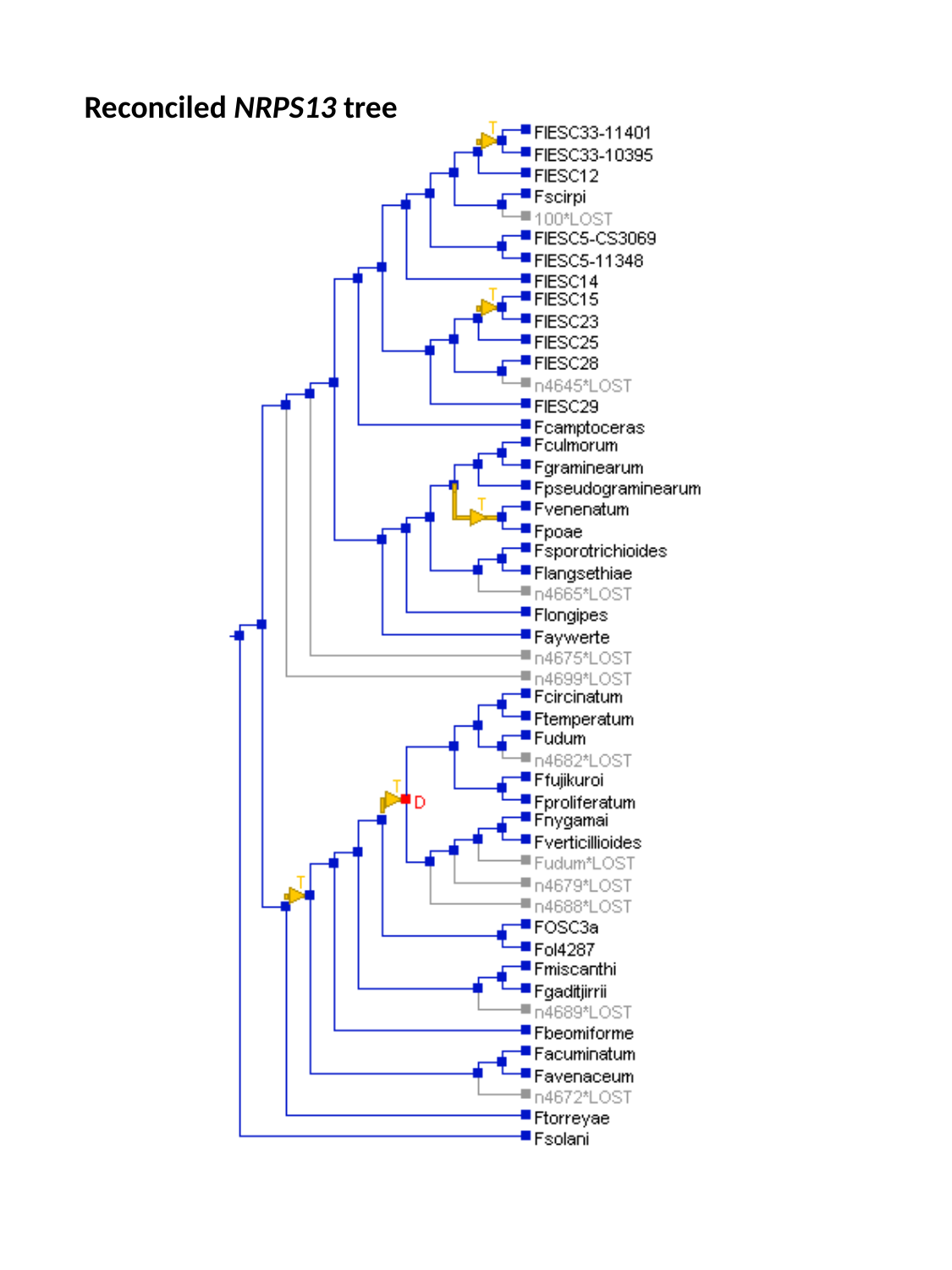

Reconciled NRPS13 tree

## Slide 11
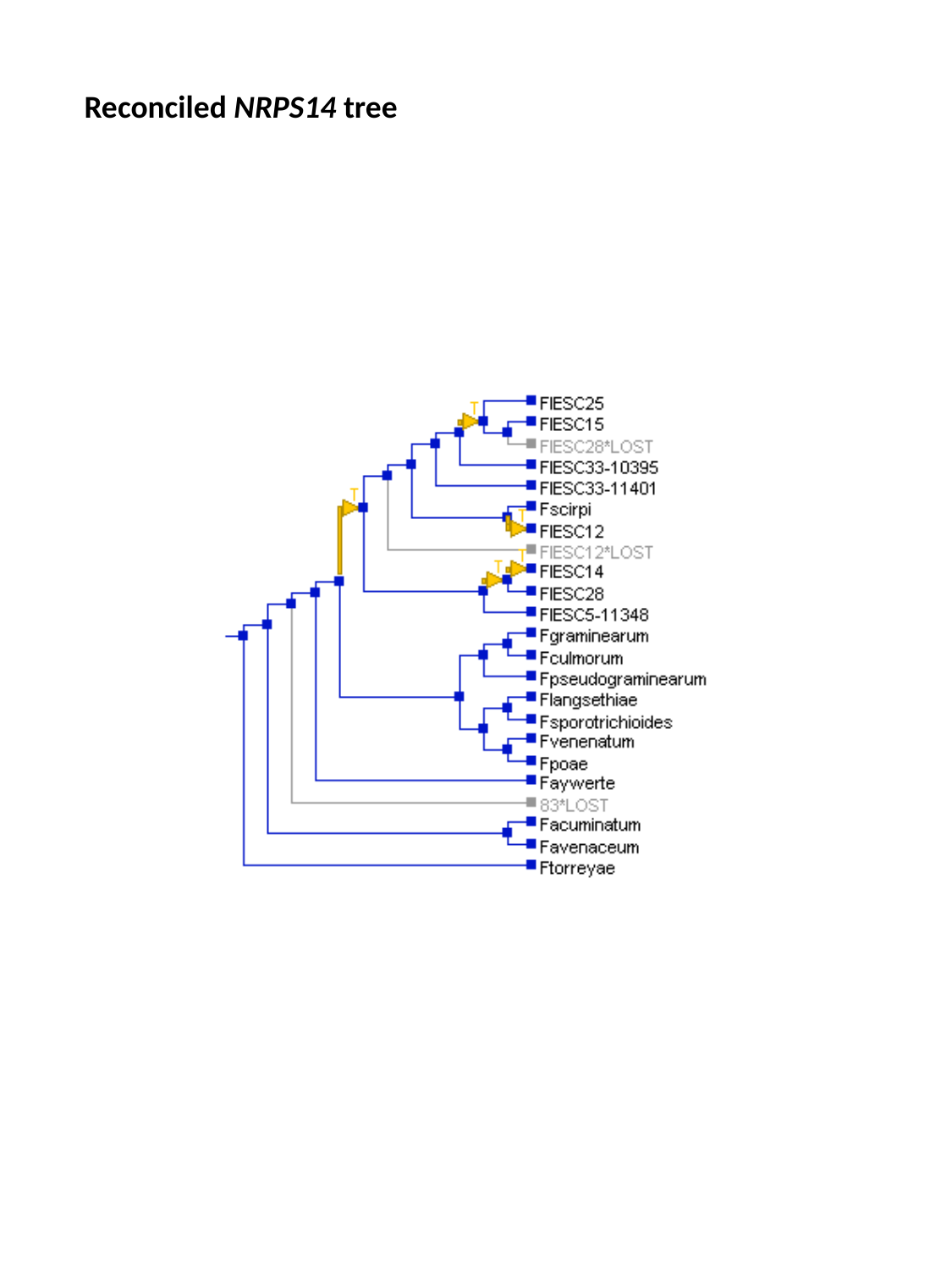

Reconciled NRPS14 tree

## Slide 12
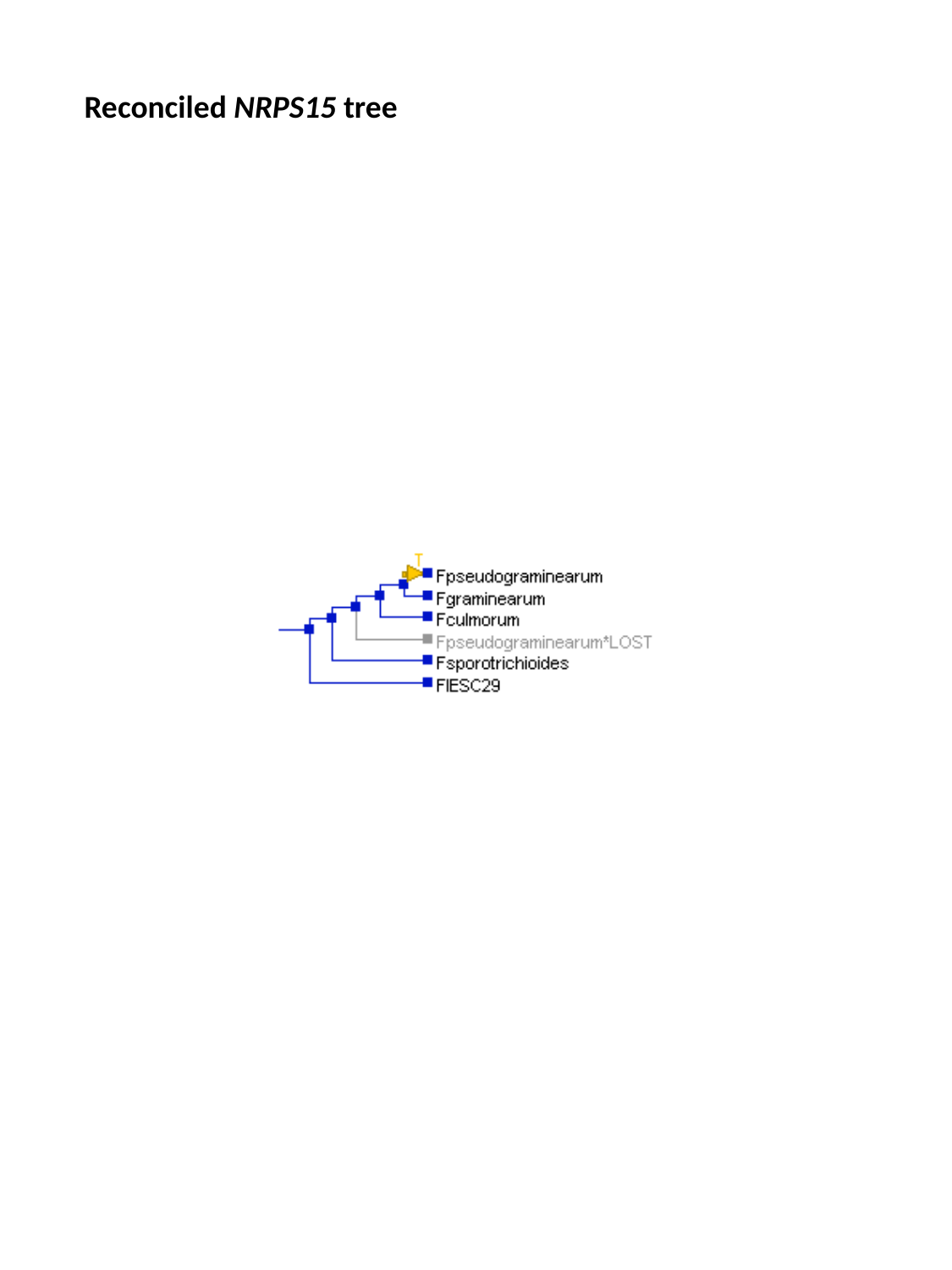

Reconciled NRPS15 tree

## Slide 13
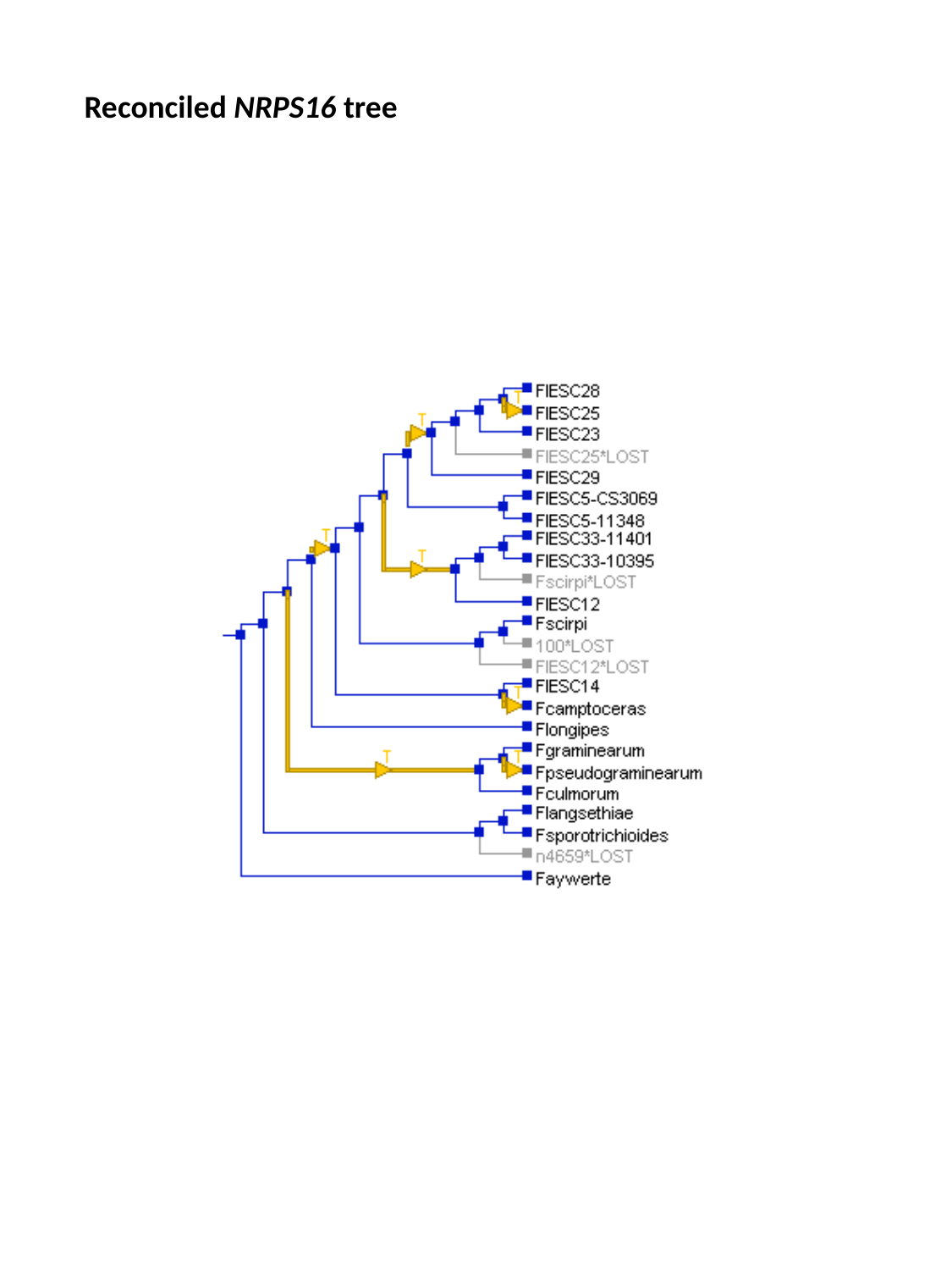

Reconciled NRPS16 tree

## Slide 14
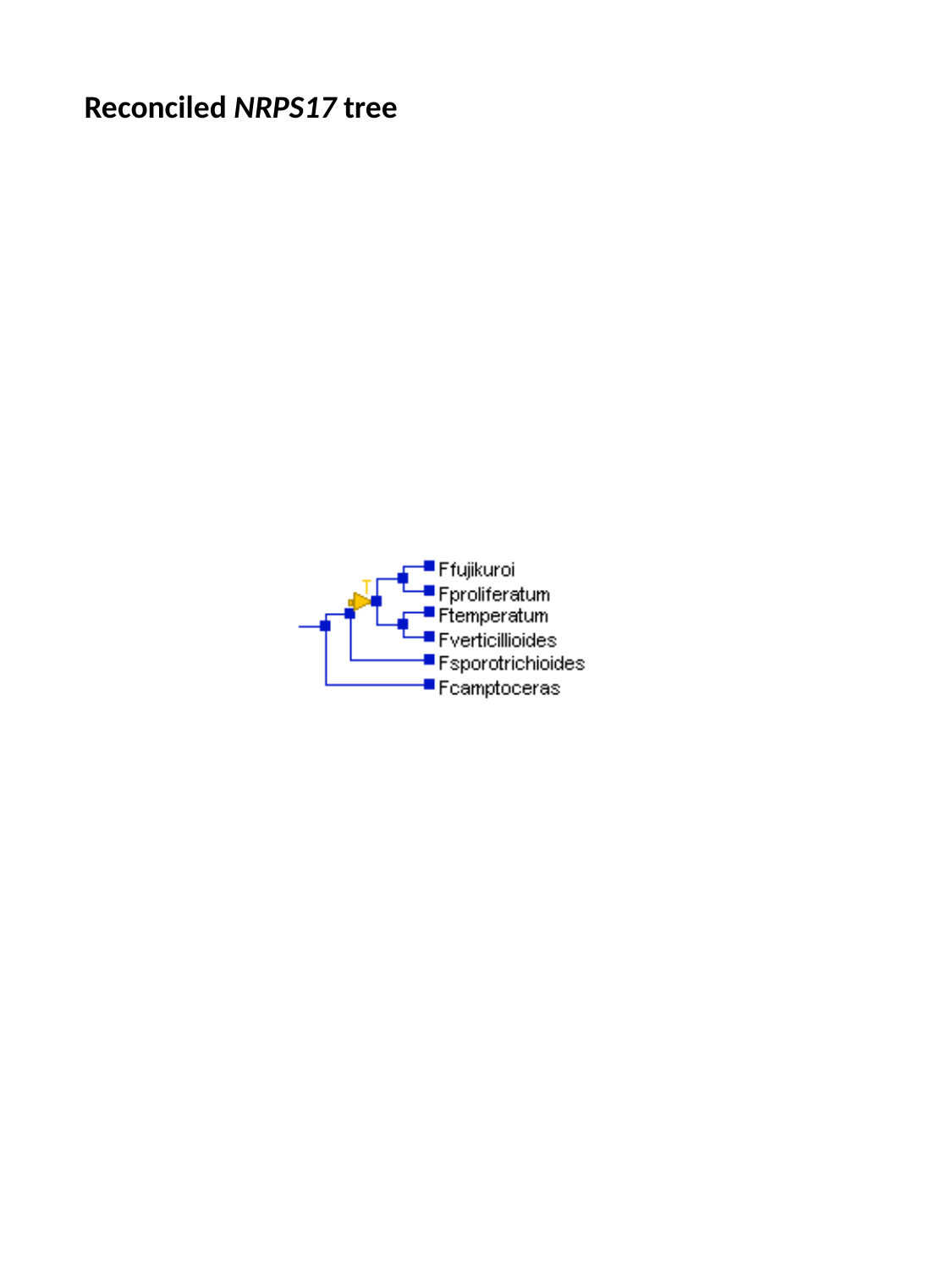

Reconciled NRPS17 tree

## Slide 15
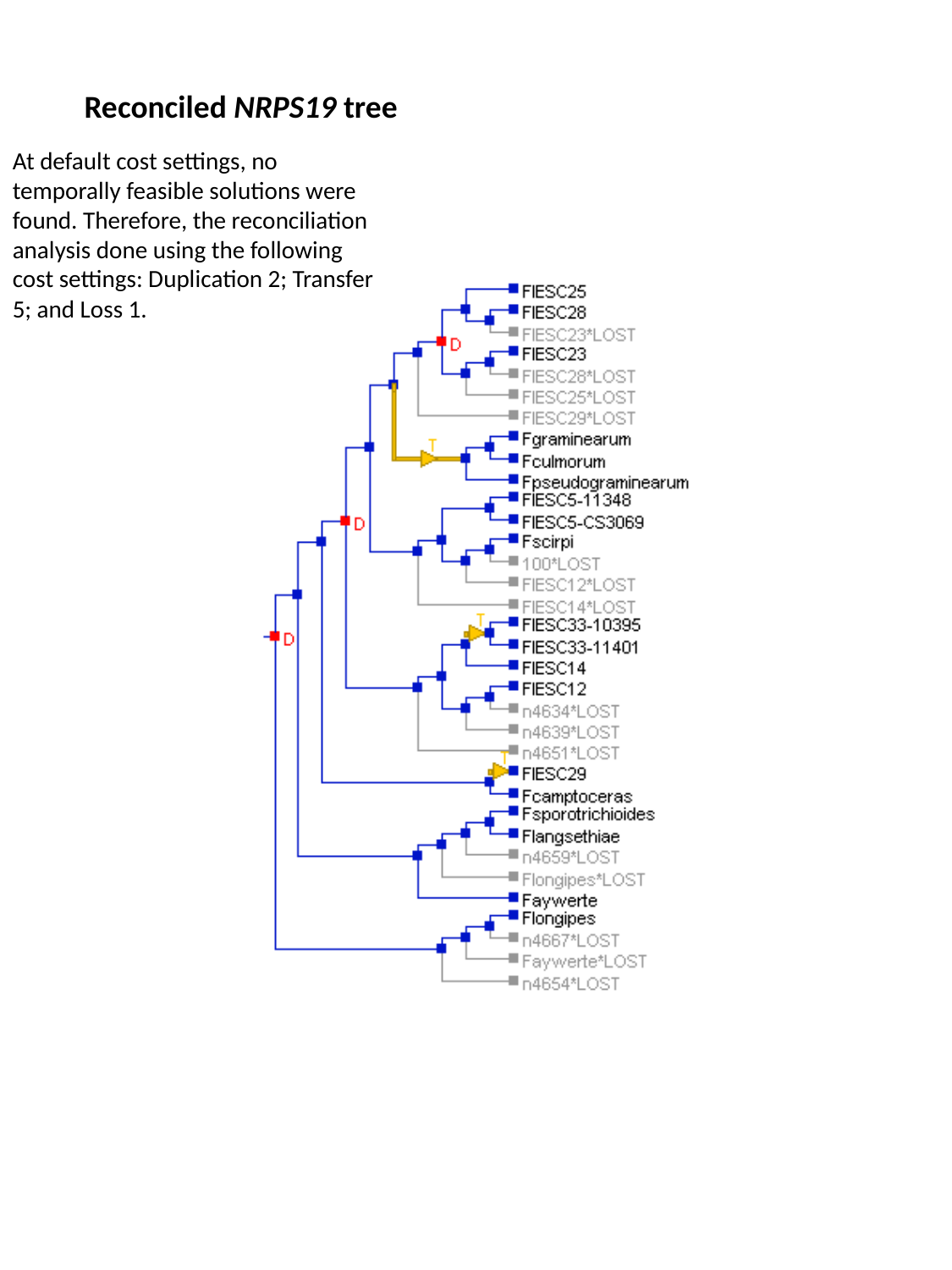

Reconciled NRPS19 tree
At default cost settings, no temporally feasible solutions were found. Therefore, the reconciliation analysis done using the following cost settings: Duplication 2; Transfer 5; and Loss 1.

## Slide 16
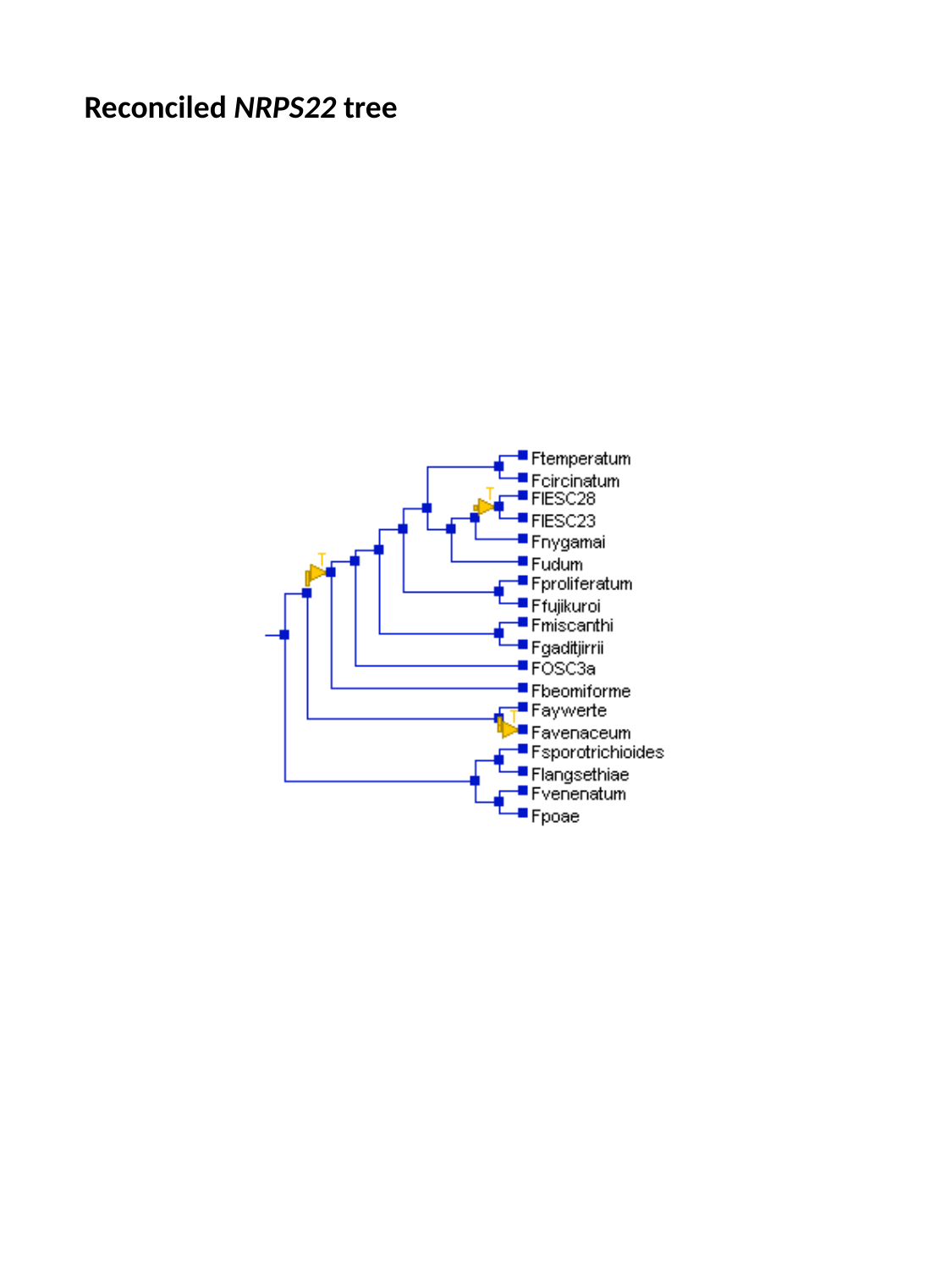

Reconciled NRPS22 tree

## Slide 17
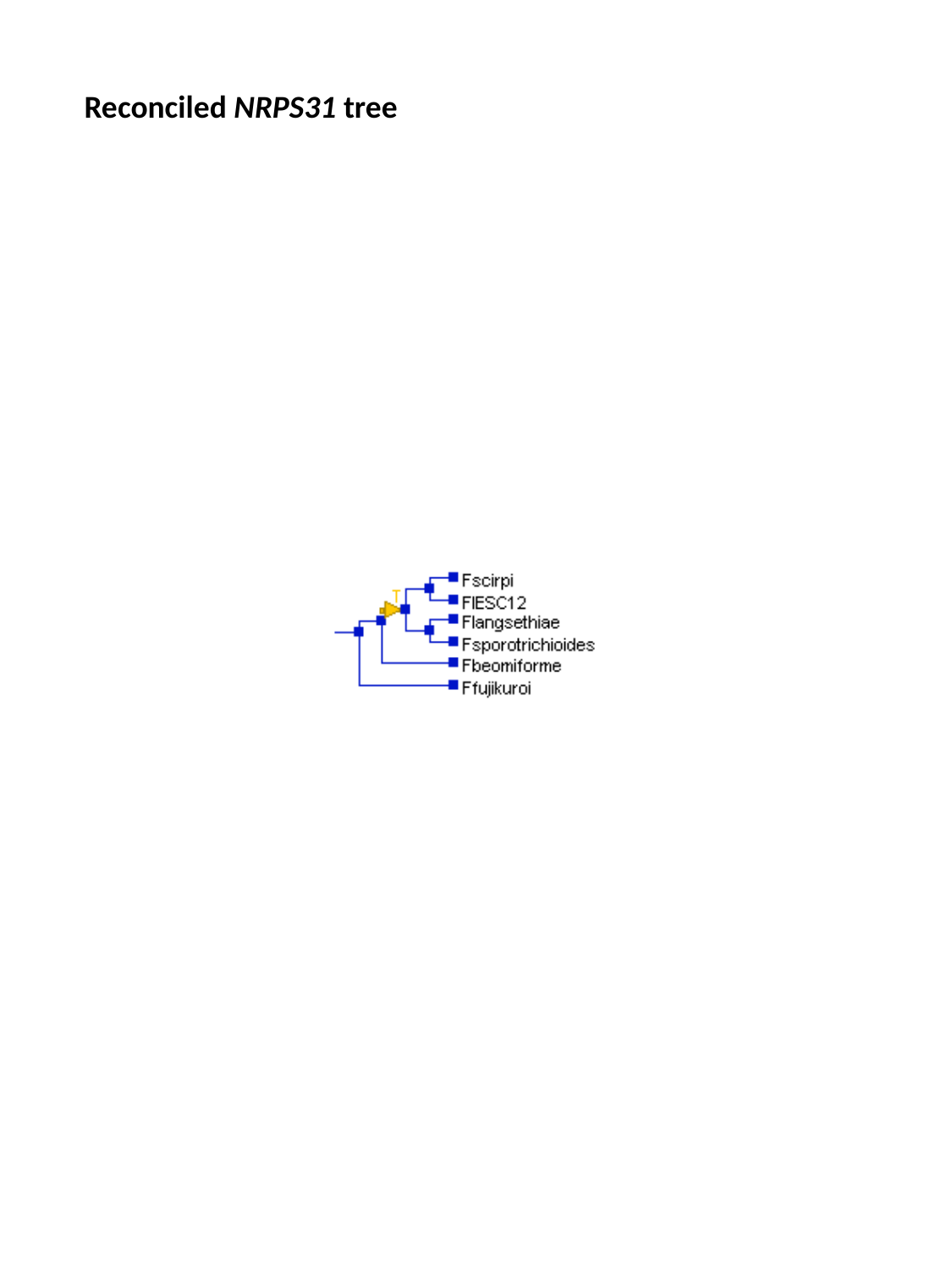

Reconciled NRPS31 tree

## Slide 18
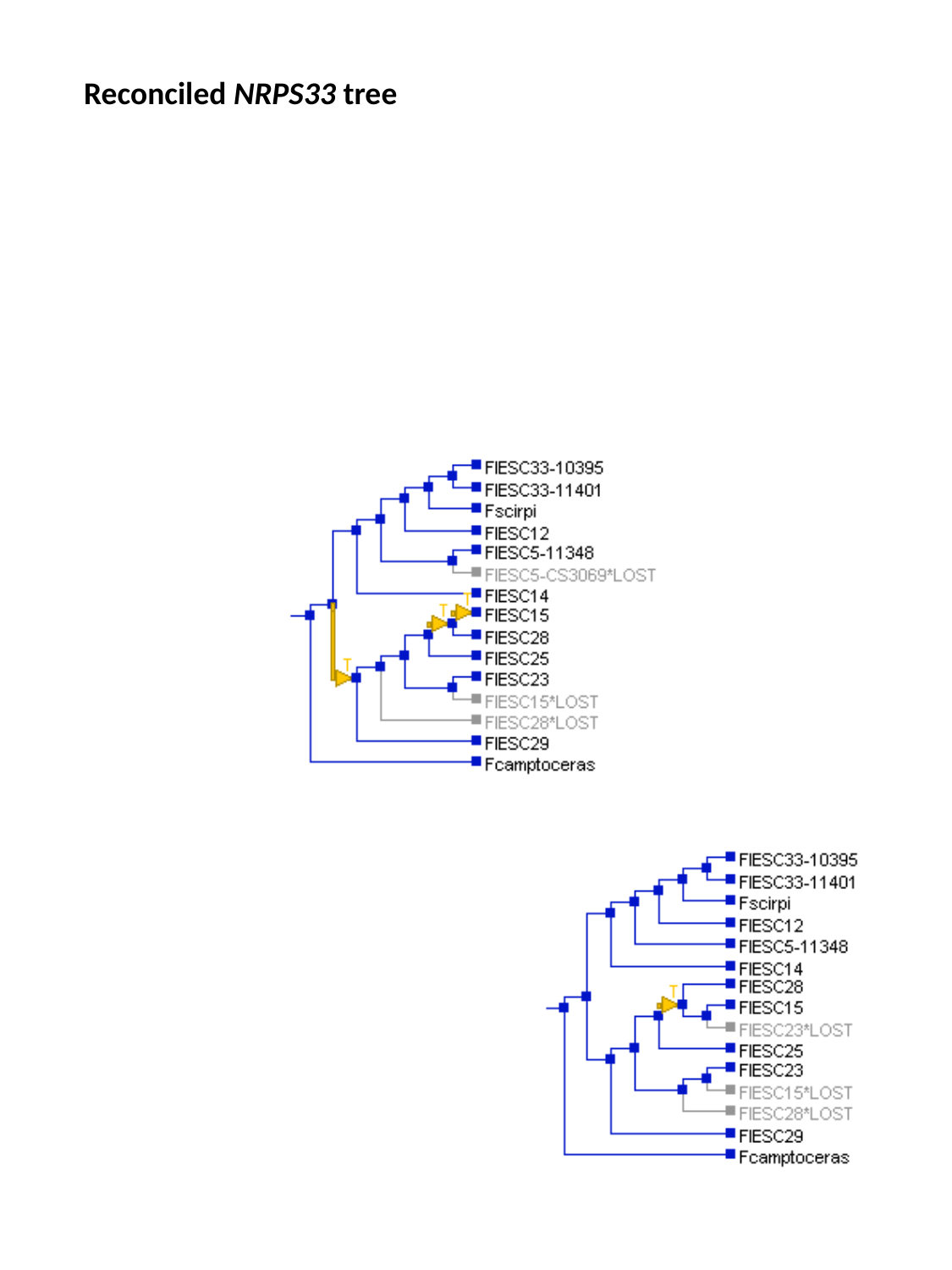

Reconciled NRPS33 tree

## Slide 19
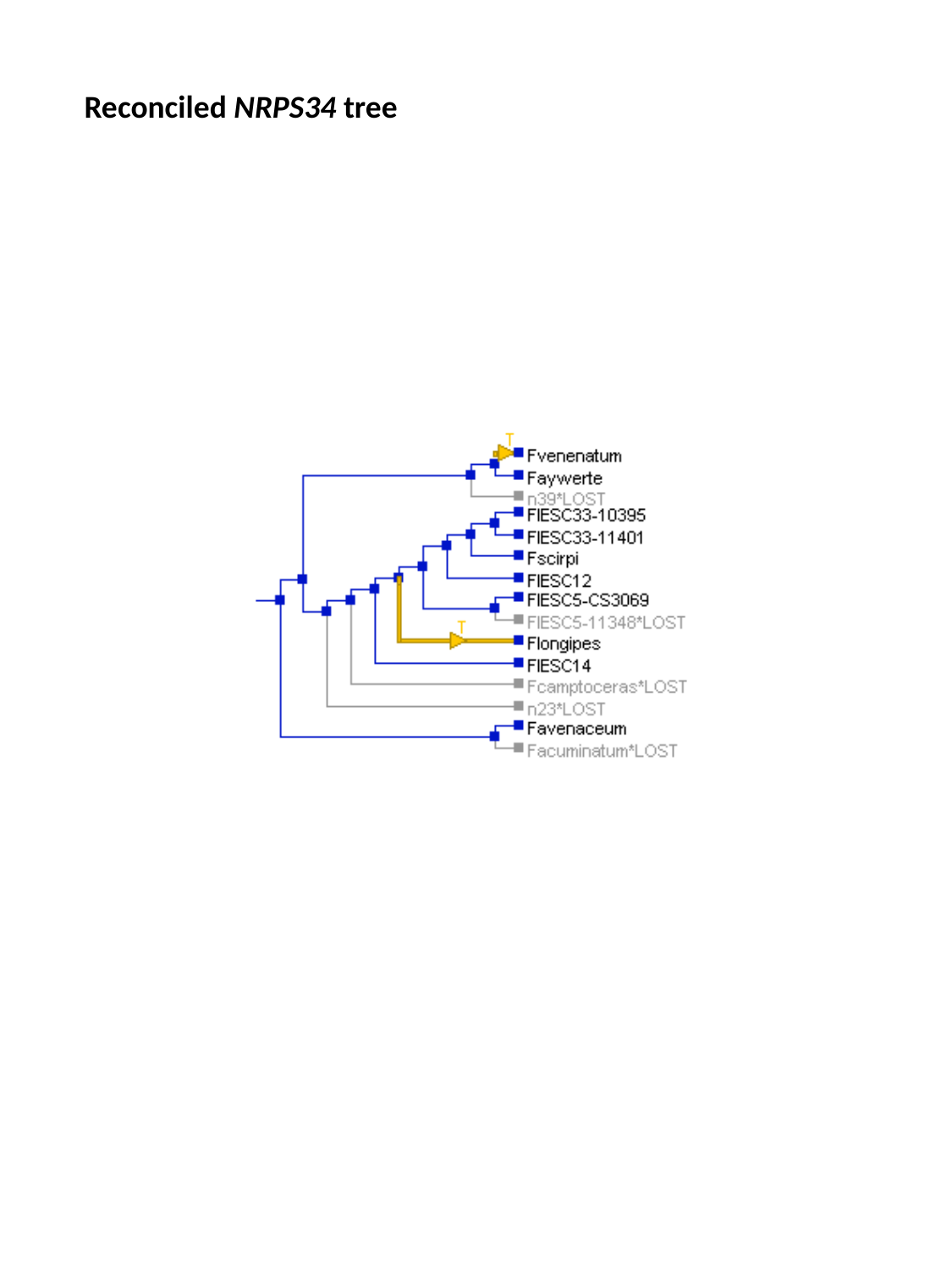

Reconciled NRPS34 tree

## Slide 20
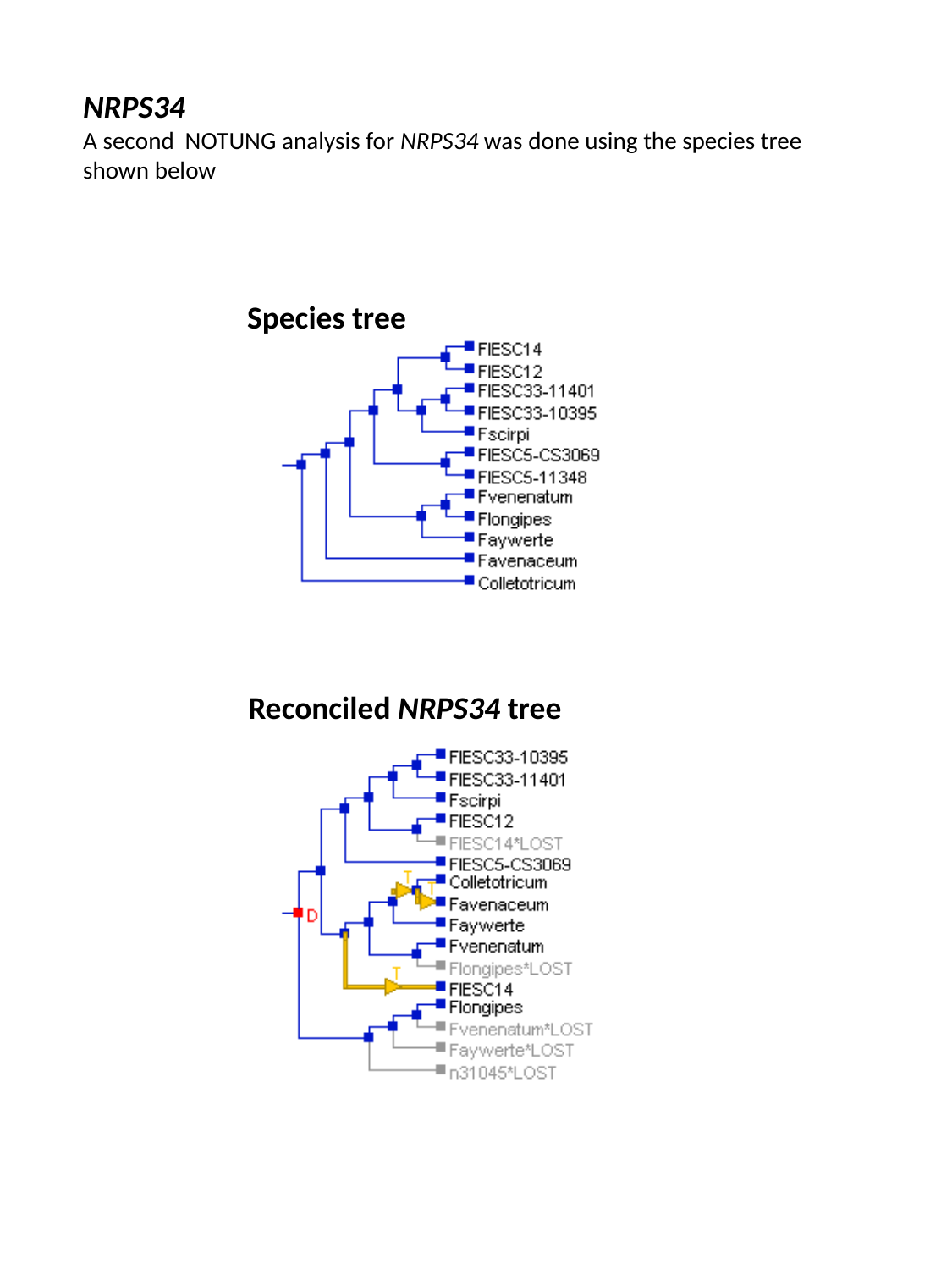

NRPS34
A second NOTUNG analysis for NRPS34 was done using the species tree shown below
Species tree
Reconciled NRPS34 tree

## Slide 21
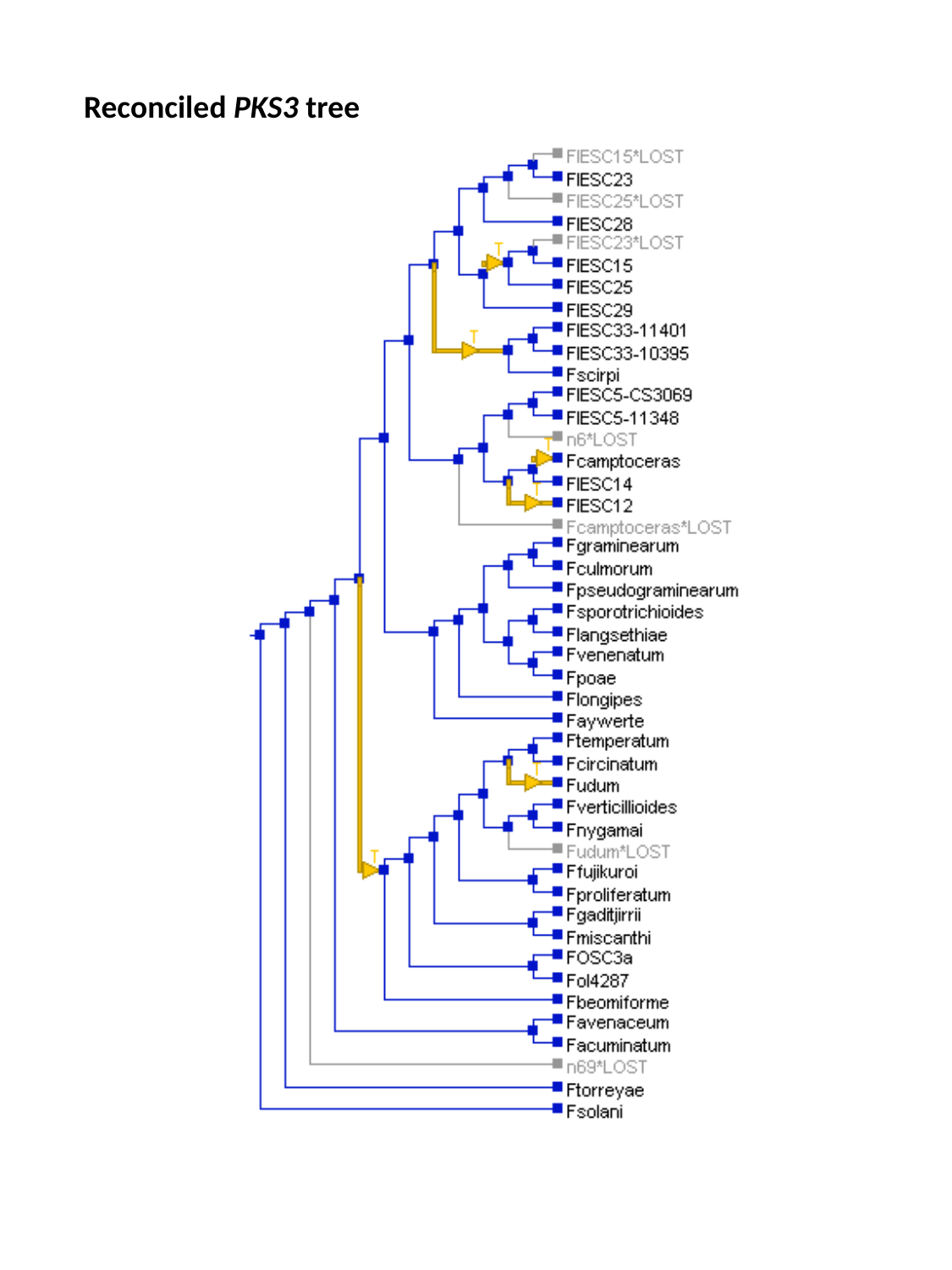

Reconciled PKS3 tree

## Slide 22
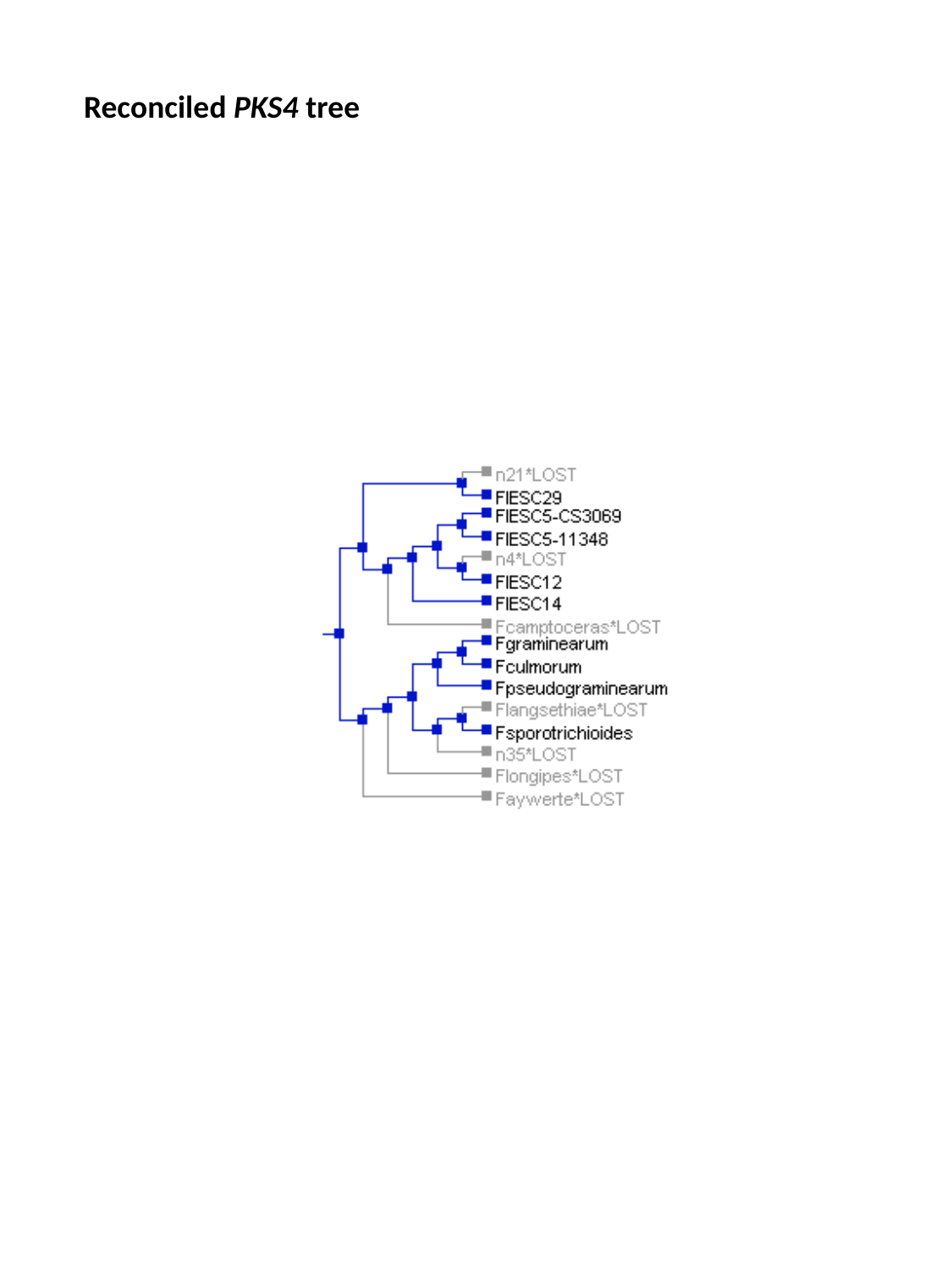

Reconciled PKS4 tree

## Slide 23
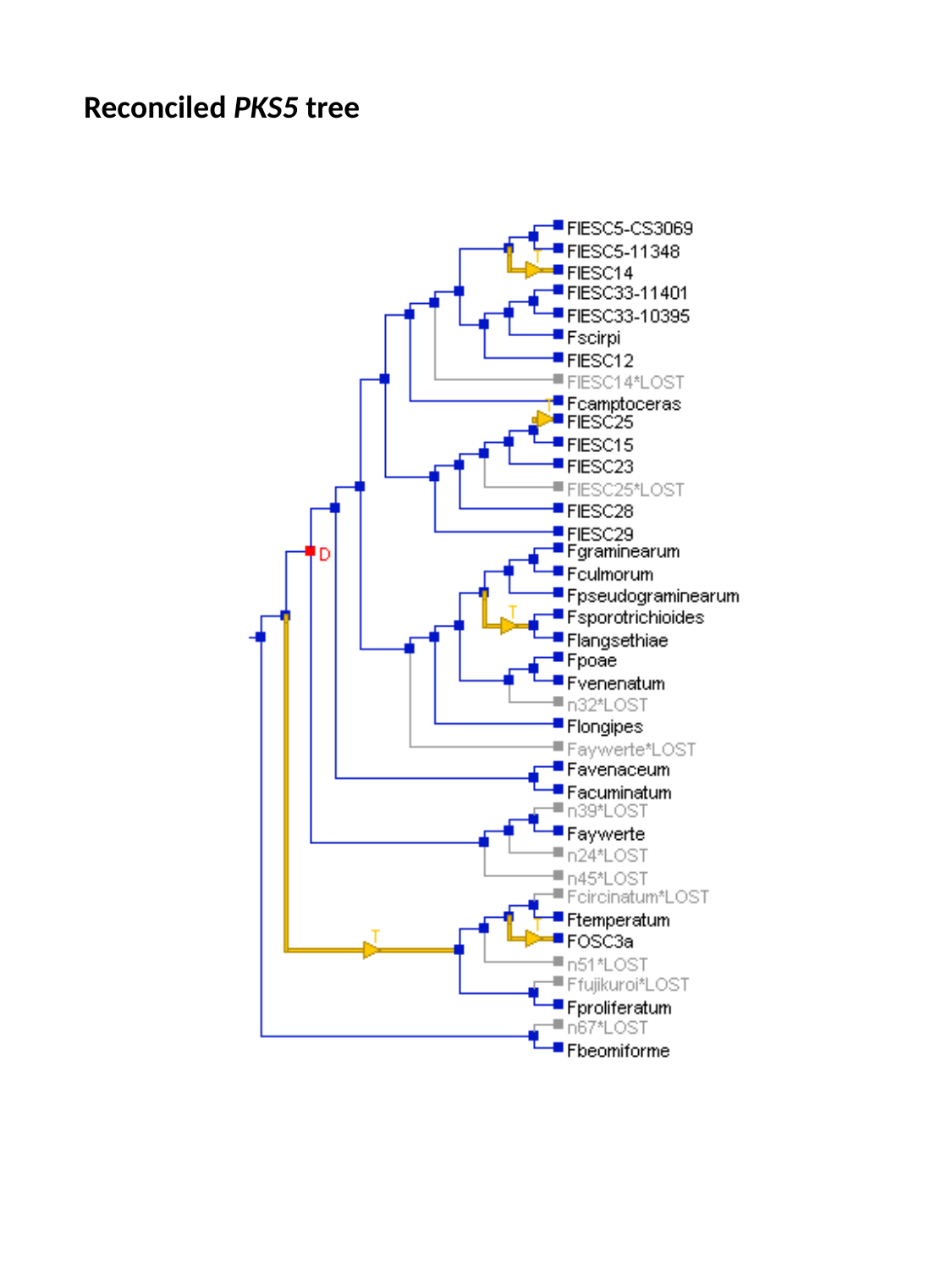

Reconciled PKS5 tree

## Slide 24
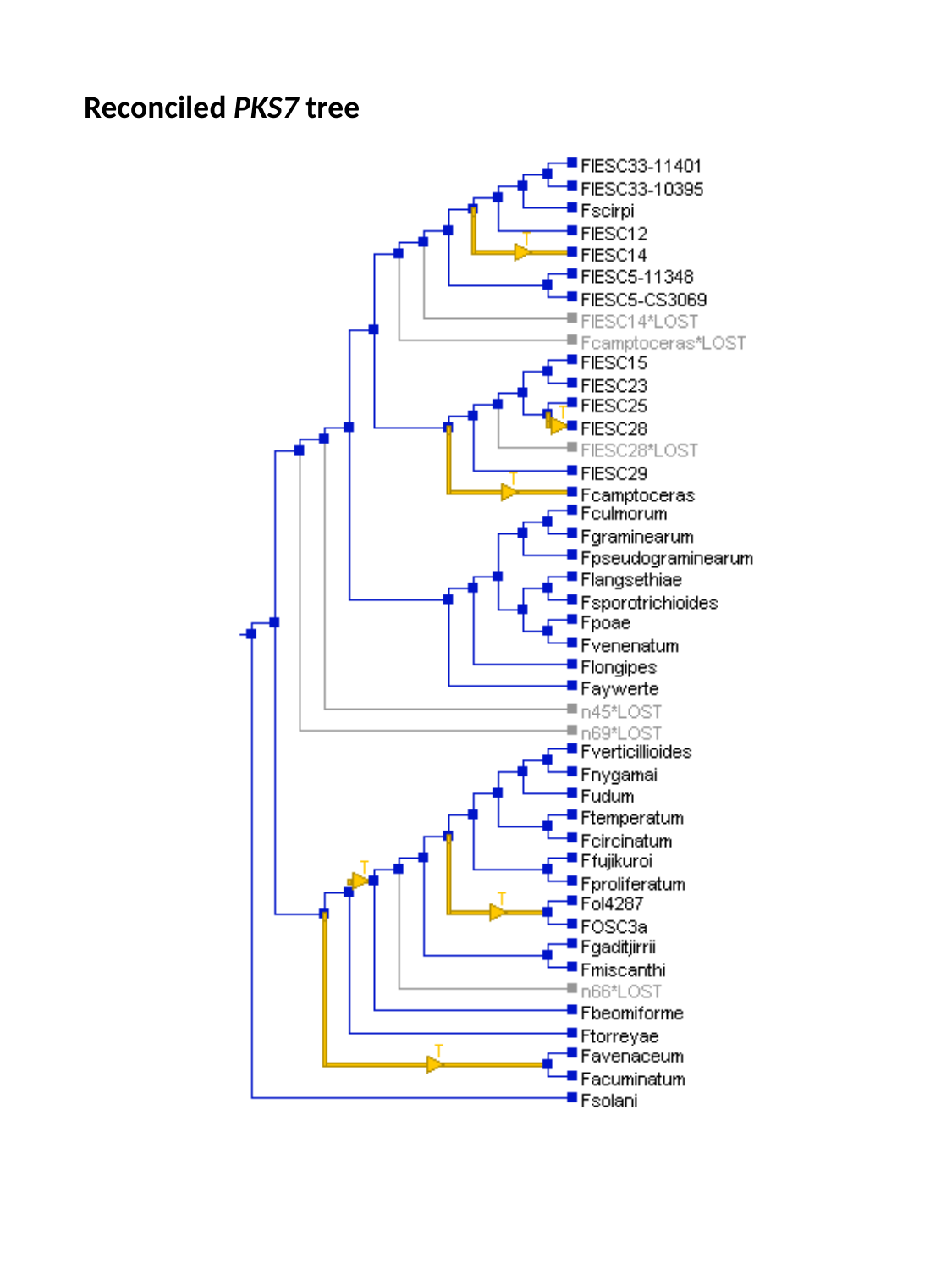

Reconciled PKS7 tree

## Slide 25
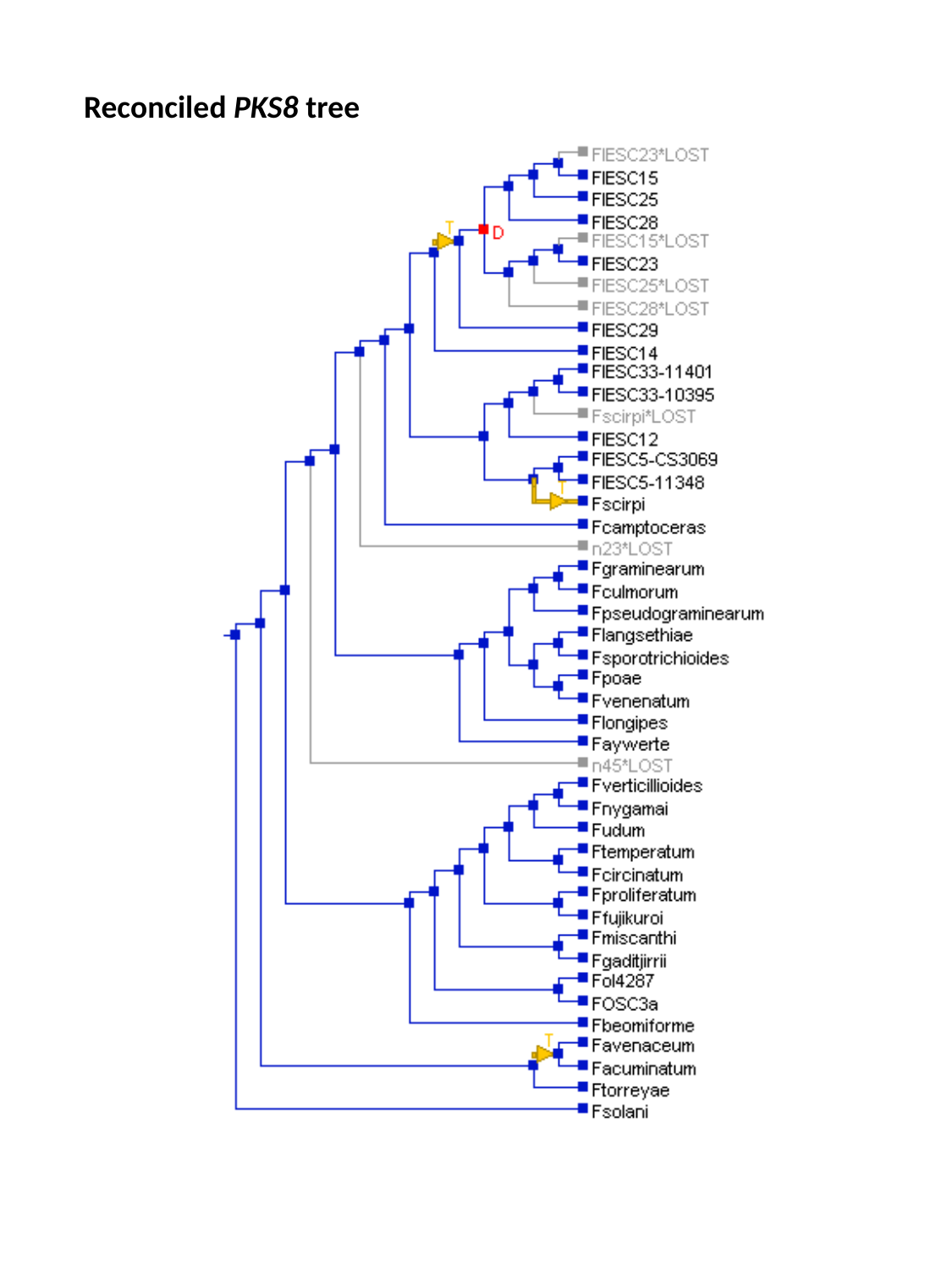

Reconciled PKS8 tree

## Slide 26
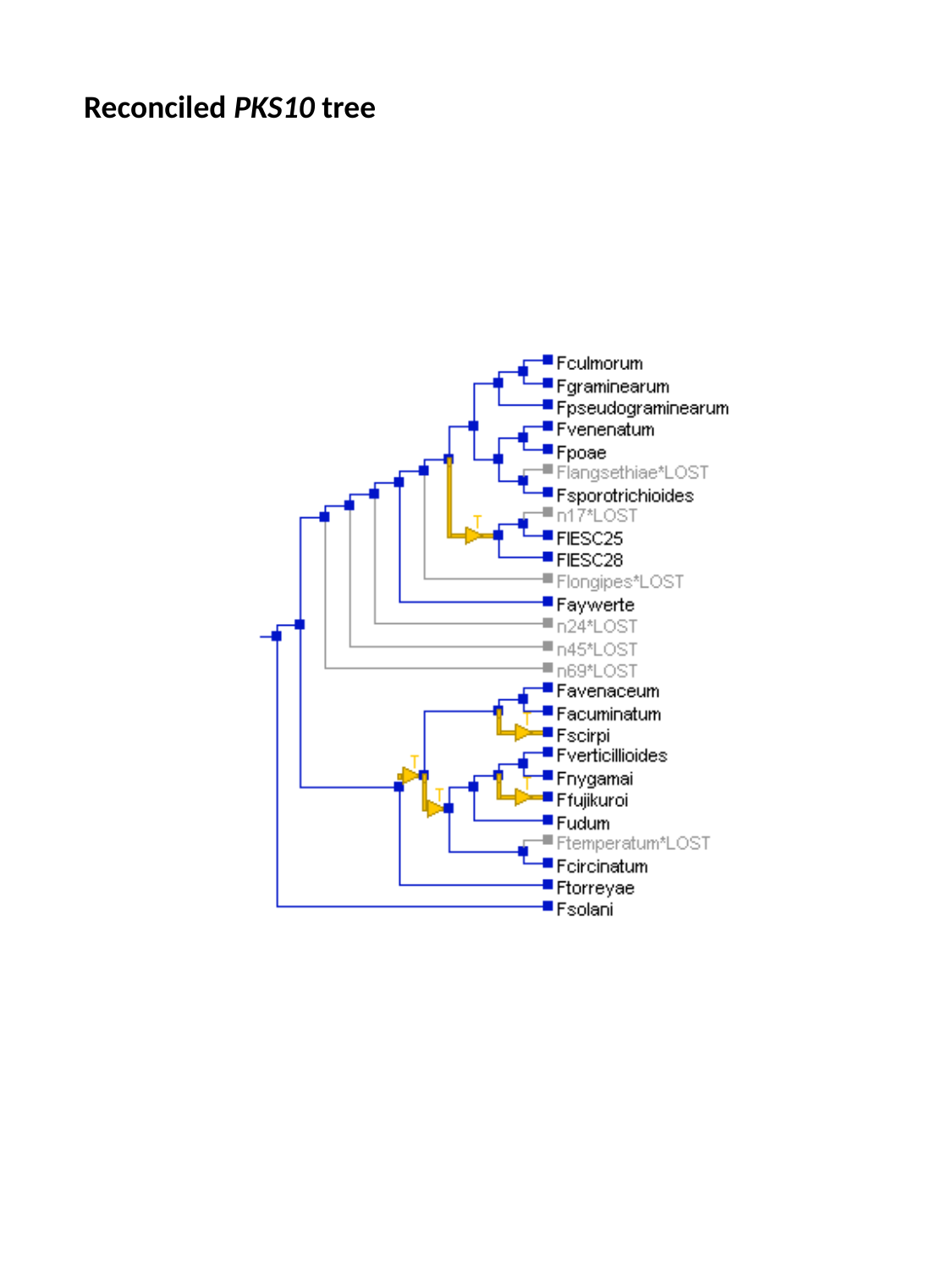

Reconciled PKS10 tree

## Slide 27
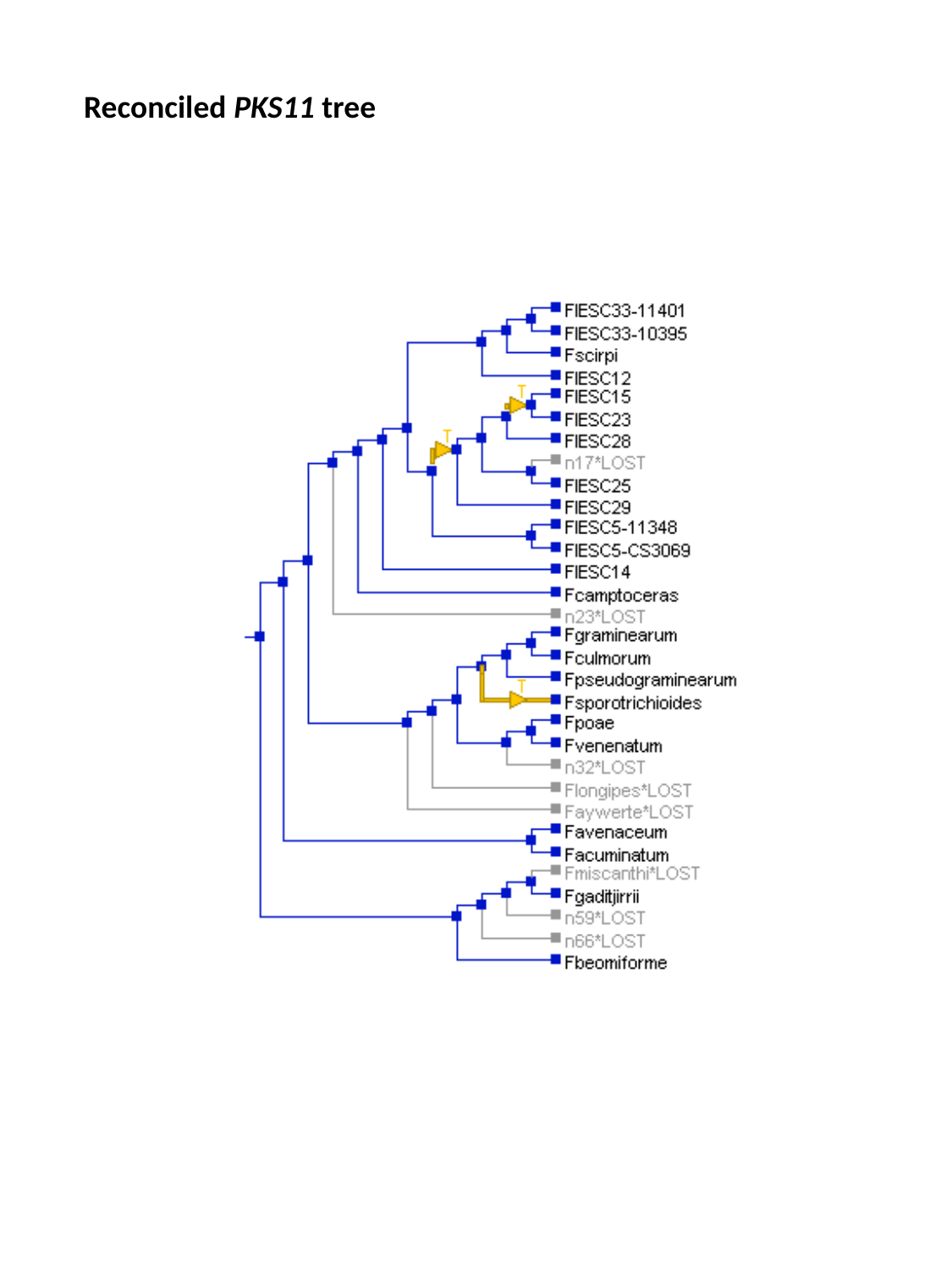

Reconciled PKS11 tree

## Slide 28
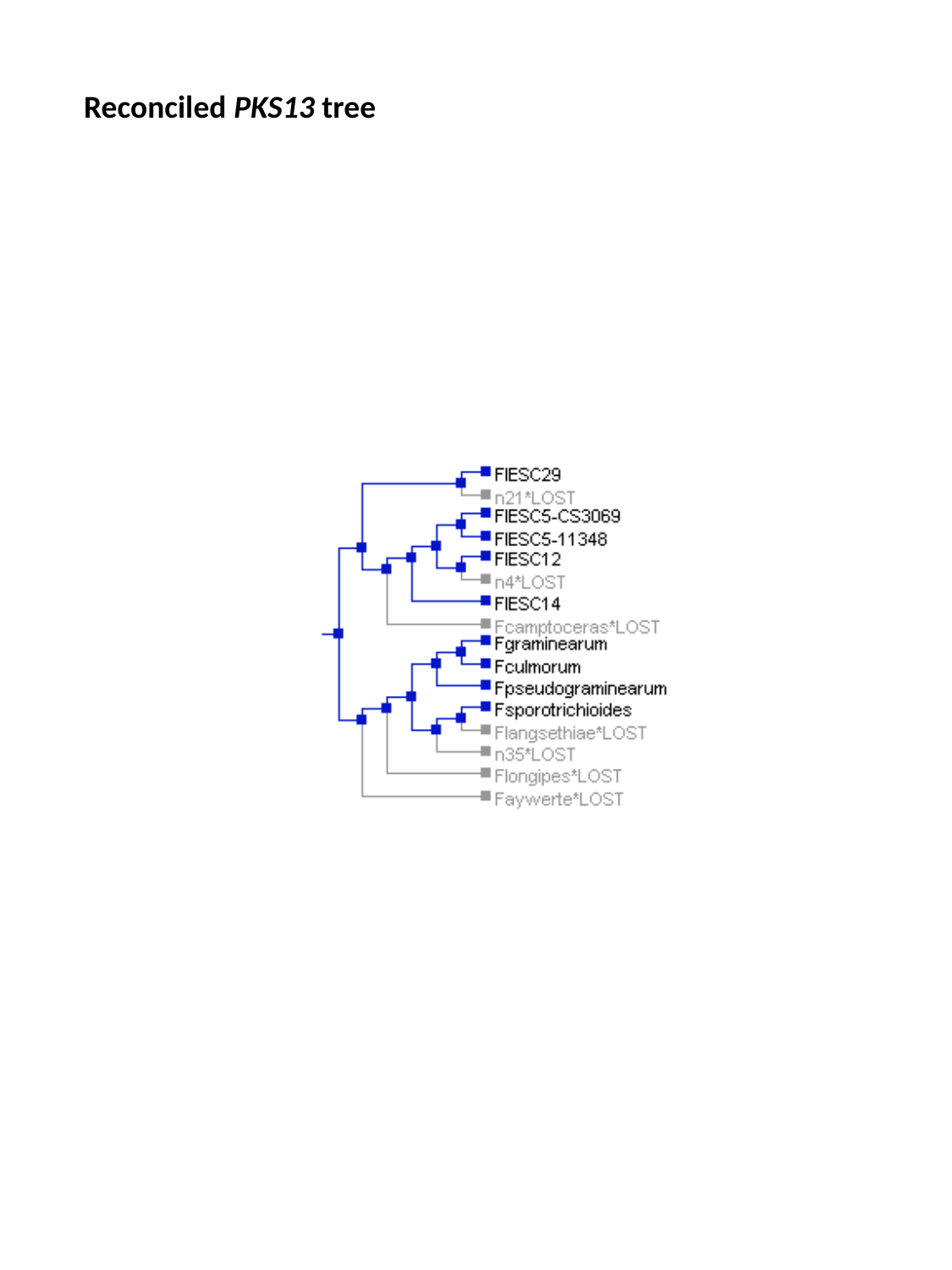

Reconciled PKS13 tree

## Slide 29
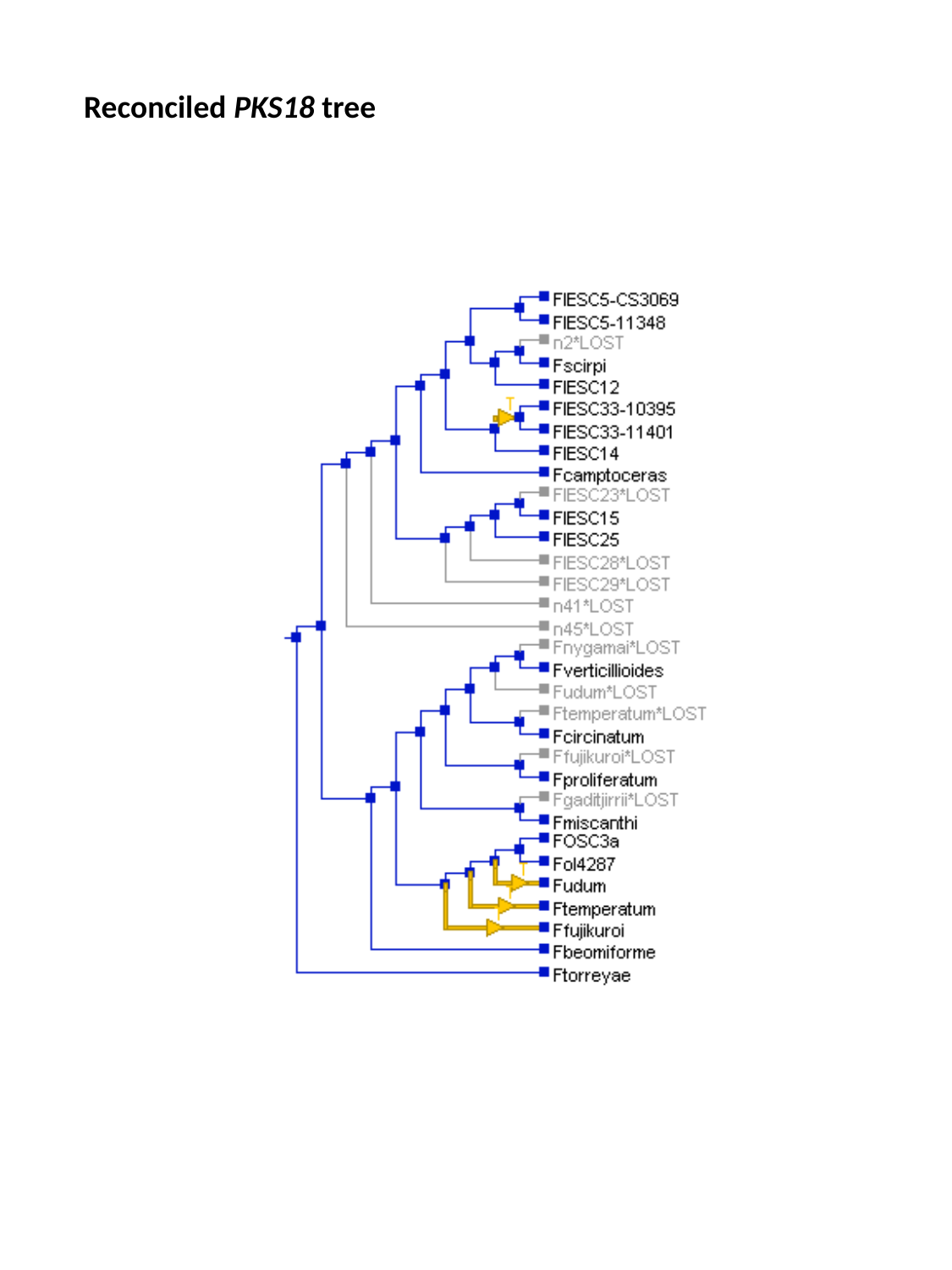

Reconciled PKS18 tree

## Slide 30
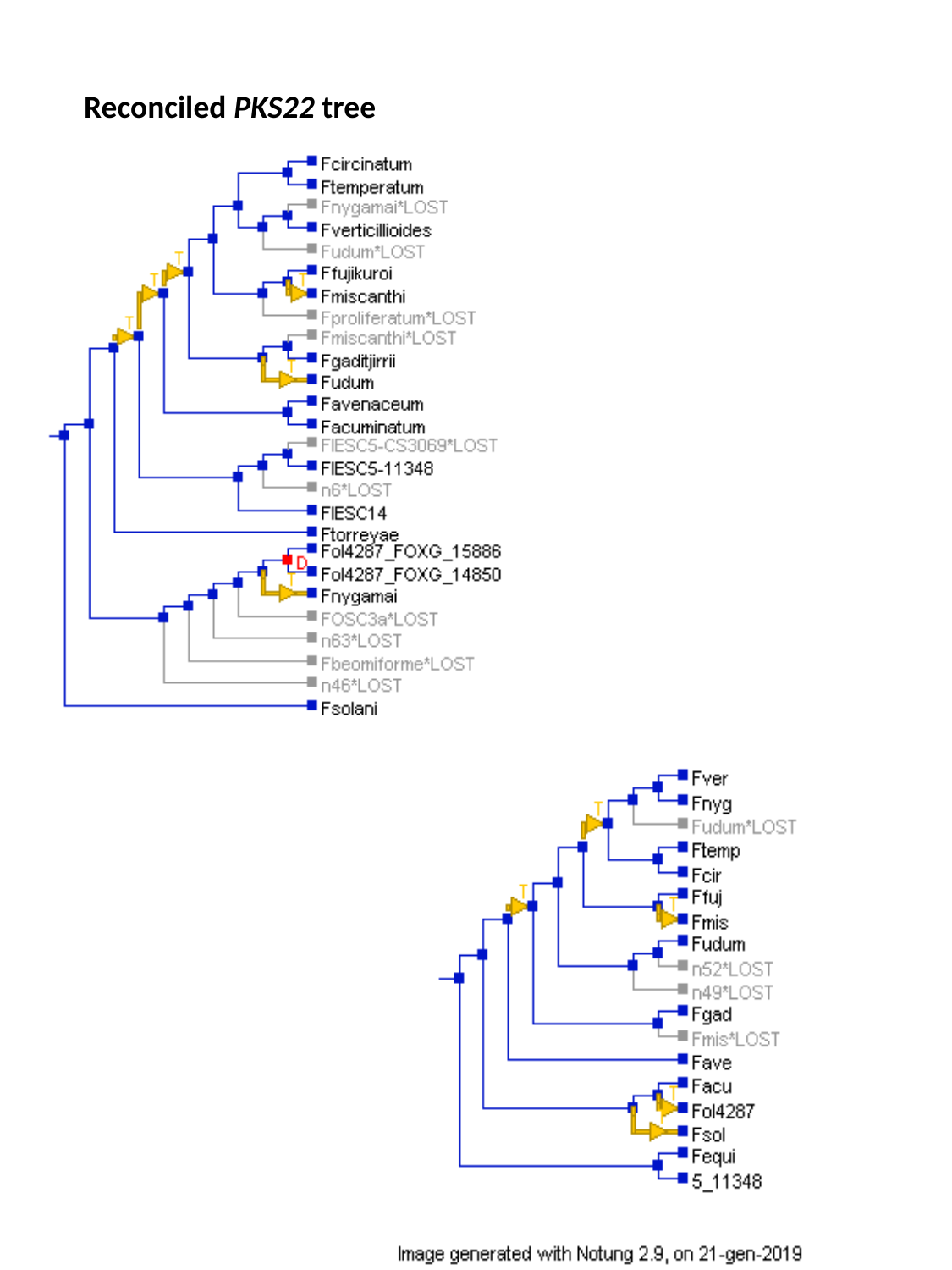

Reconciled PKS22 tree

## Slide 31
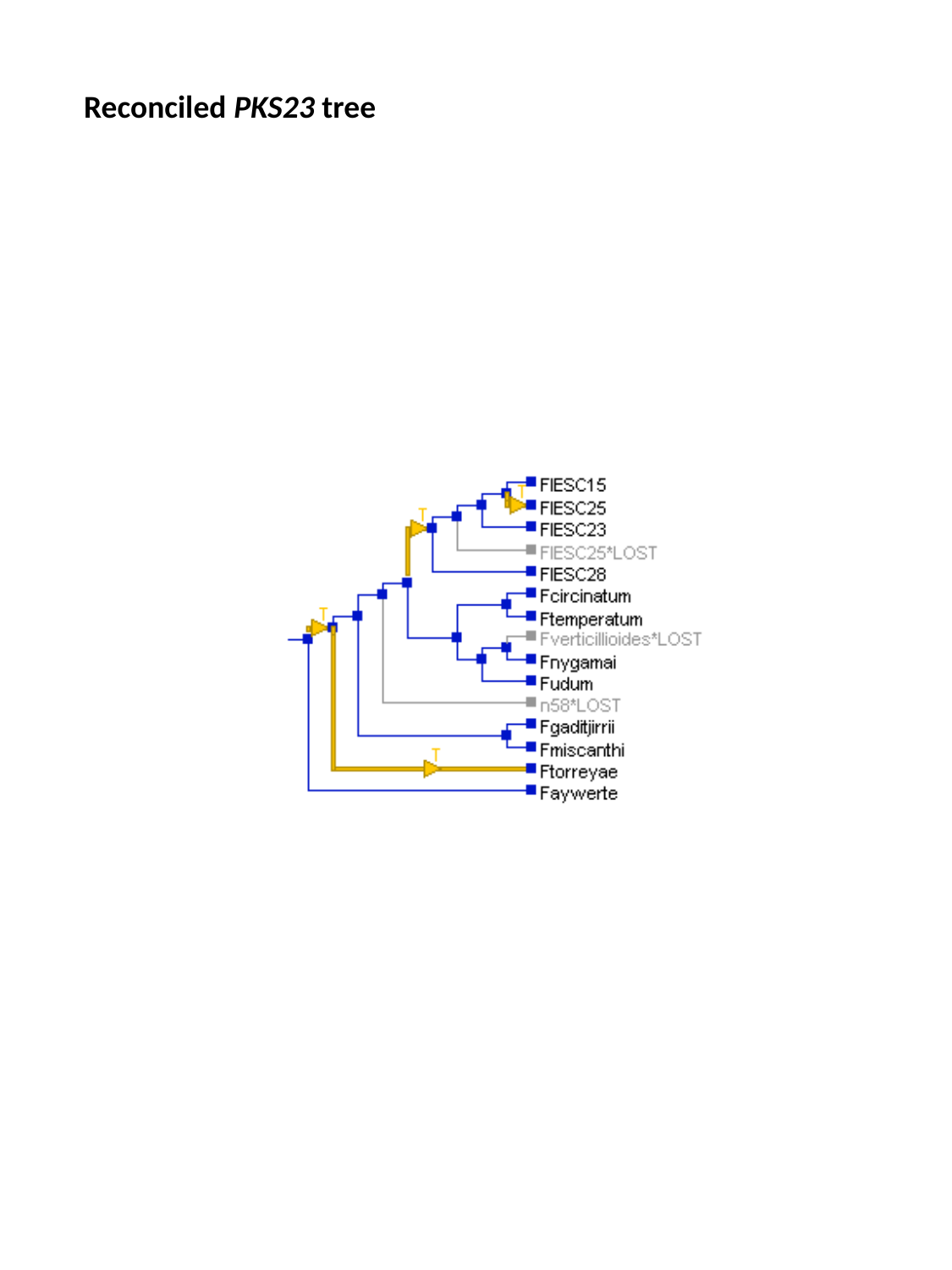

Reconciled PKS23 tree

## Slide 32
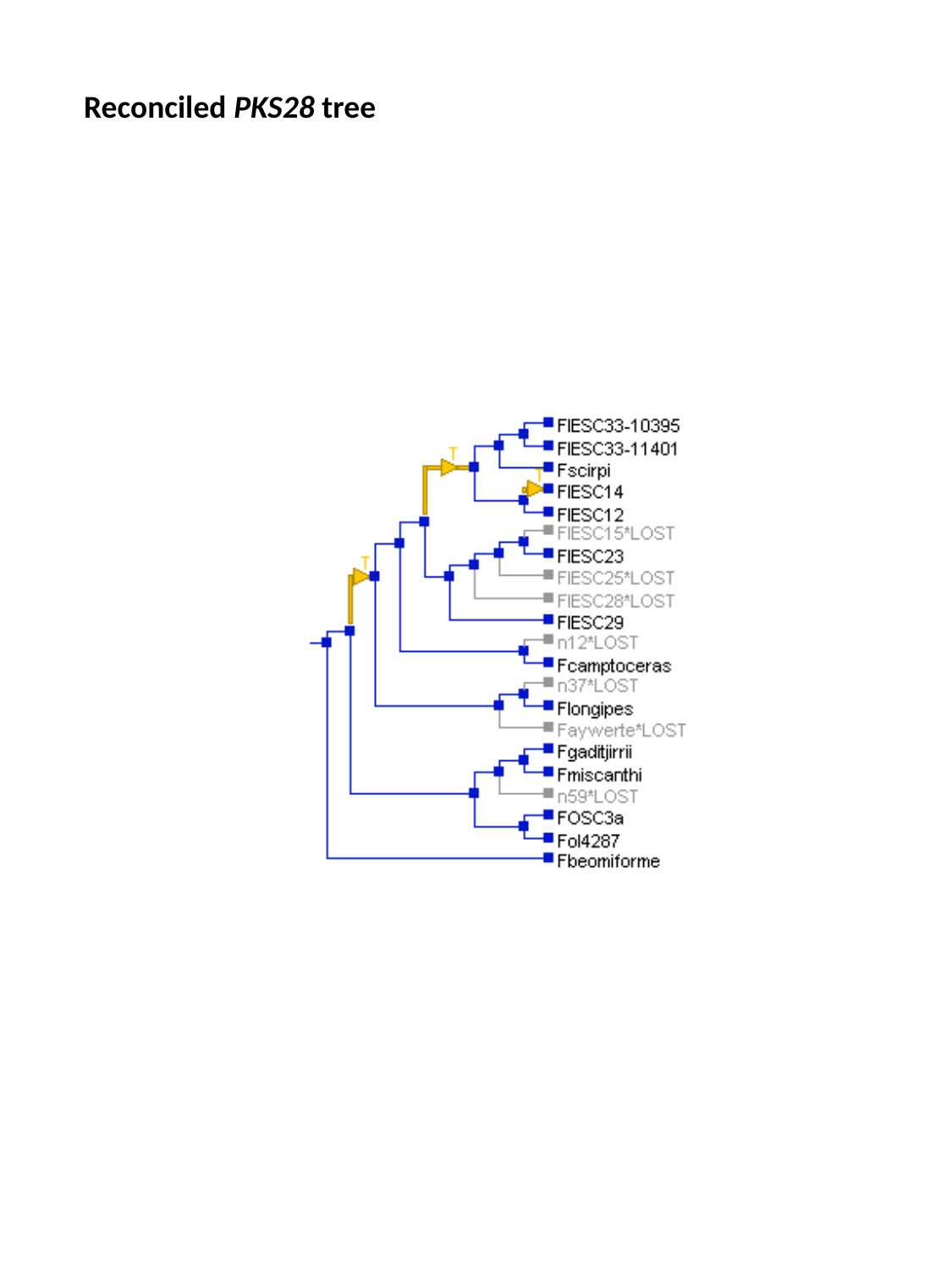

Reconciled PKS28 tree

## Slide 33
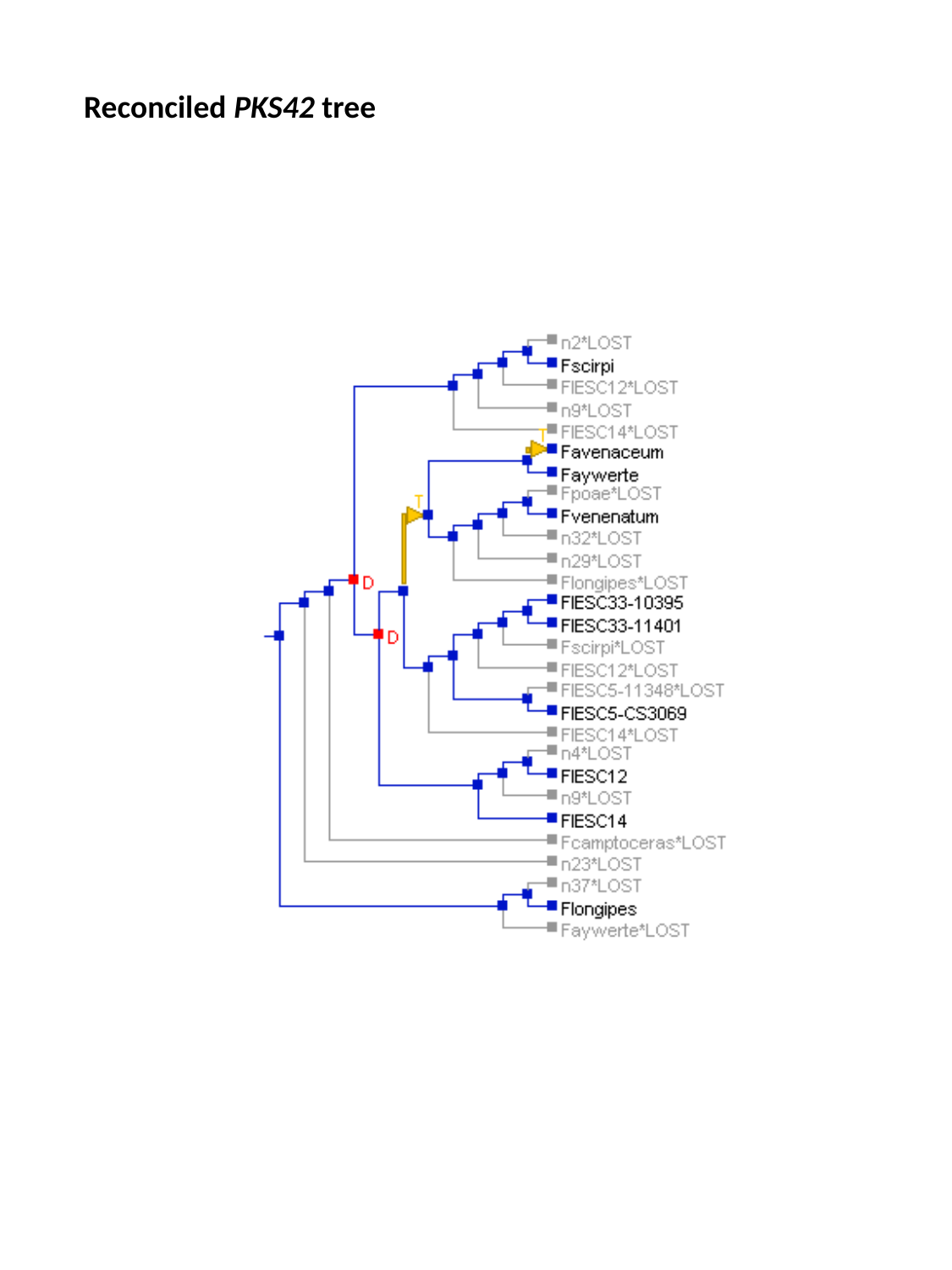

Reconciled PKS42 tree

## Slide 34
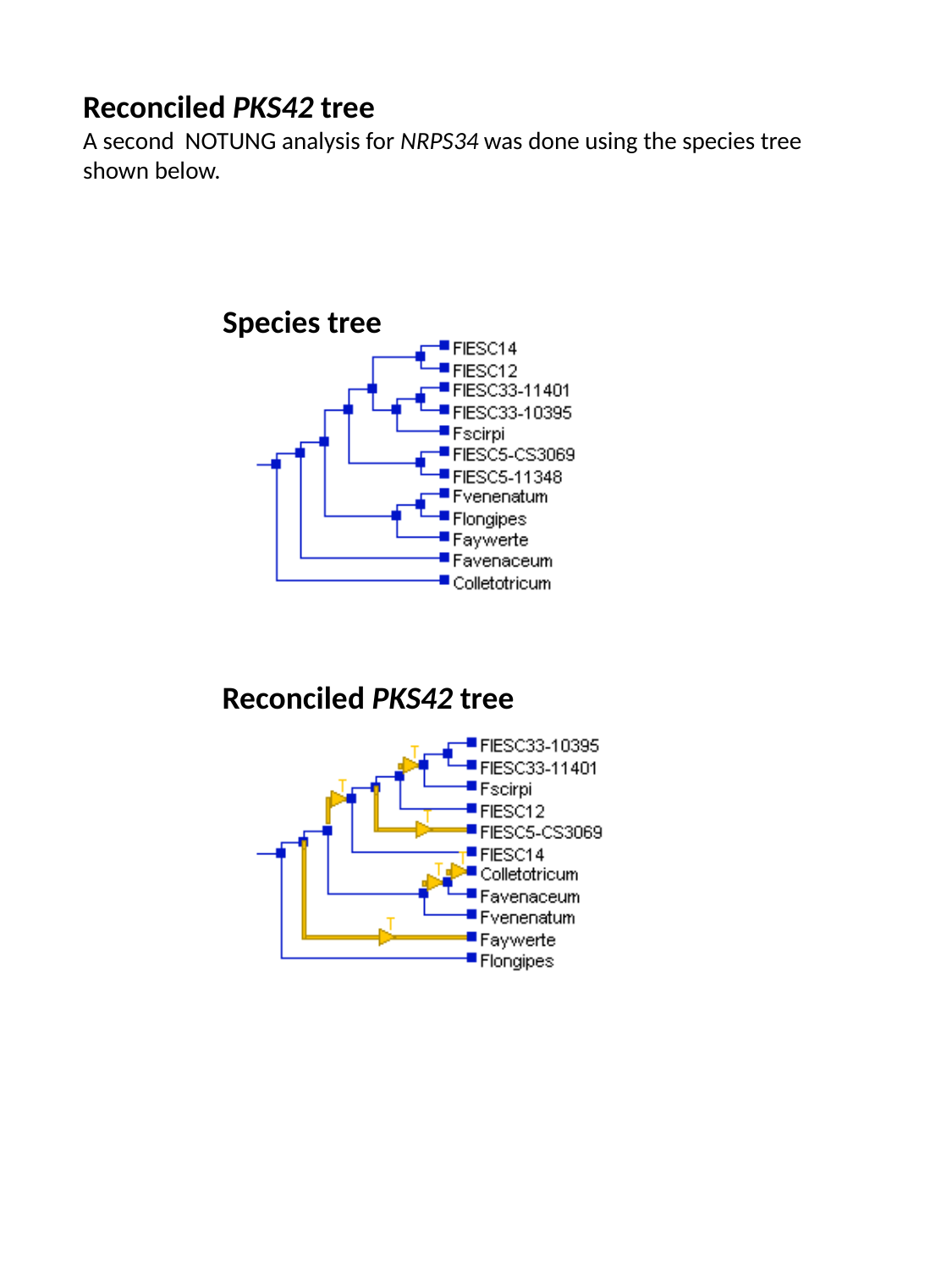

Reconciled PKS42 tree
A second NOTUNG analysis for NRPS34 was done using the species tree shown below.
Species tree
Reconciled PKS42 tree

## Slide 35
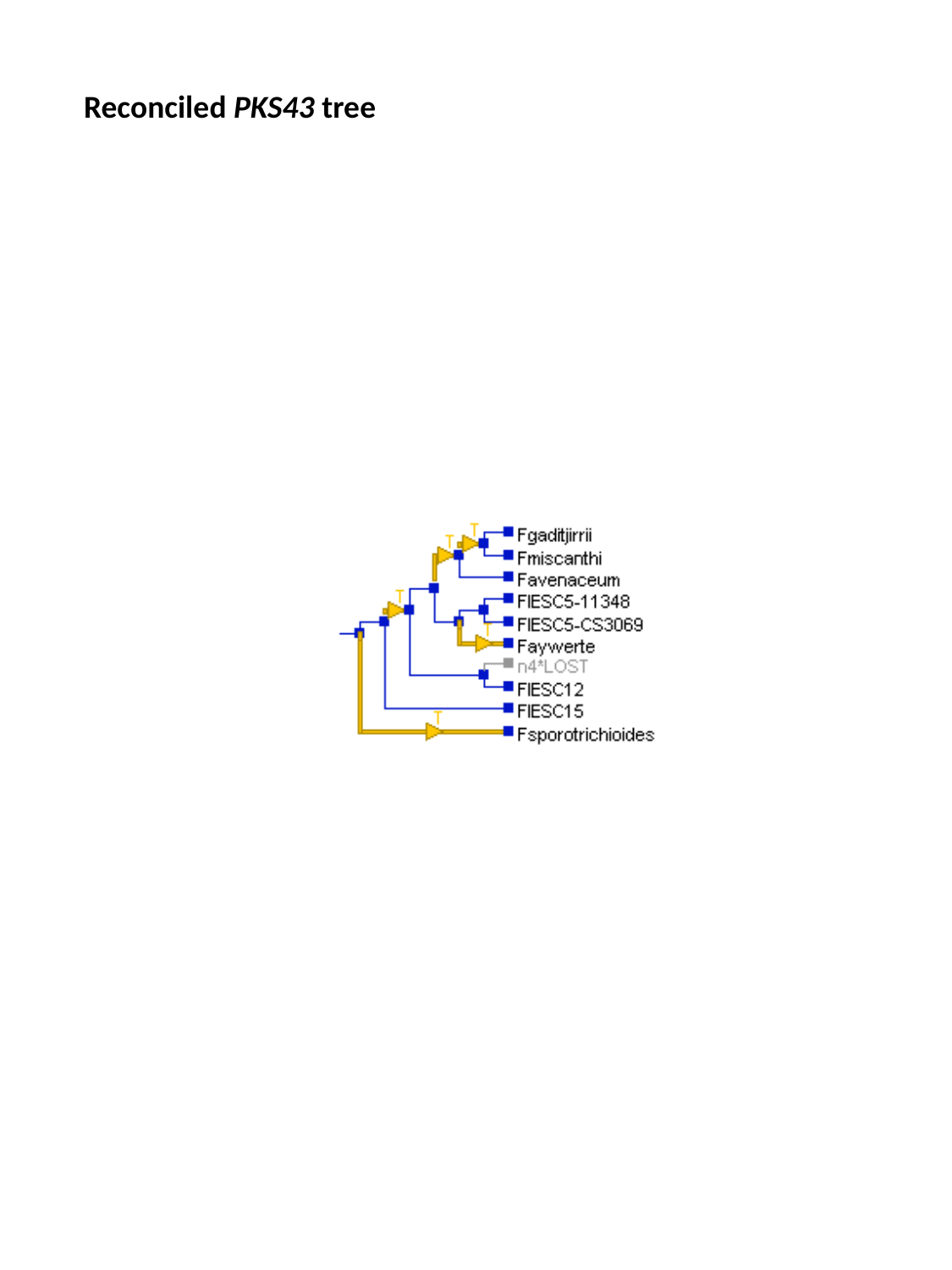

Reconciled PKS43 tree

## Slide 36
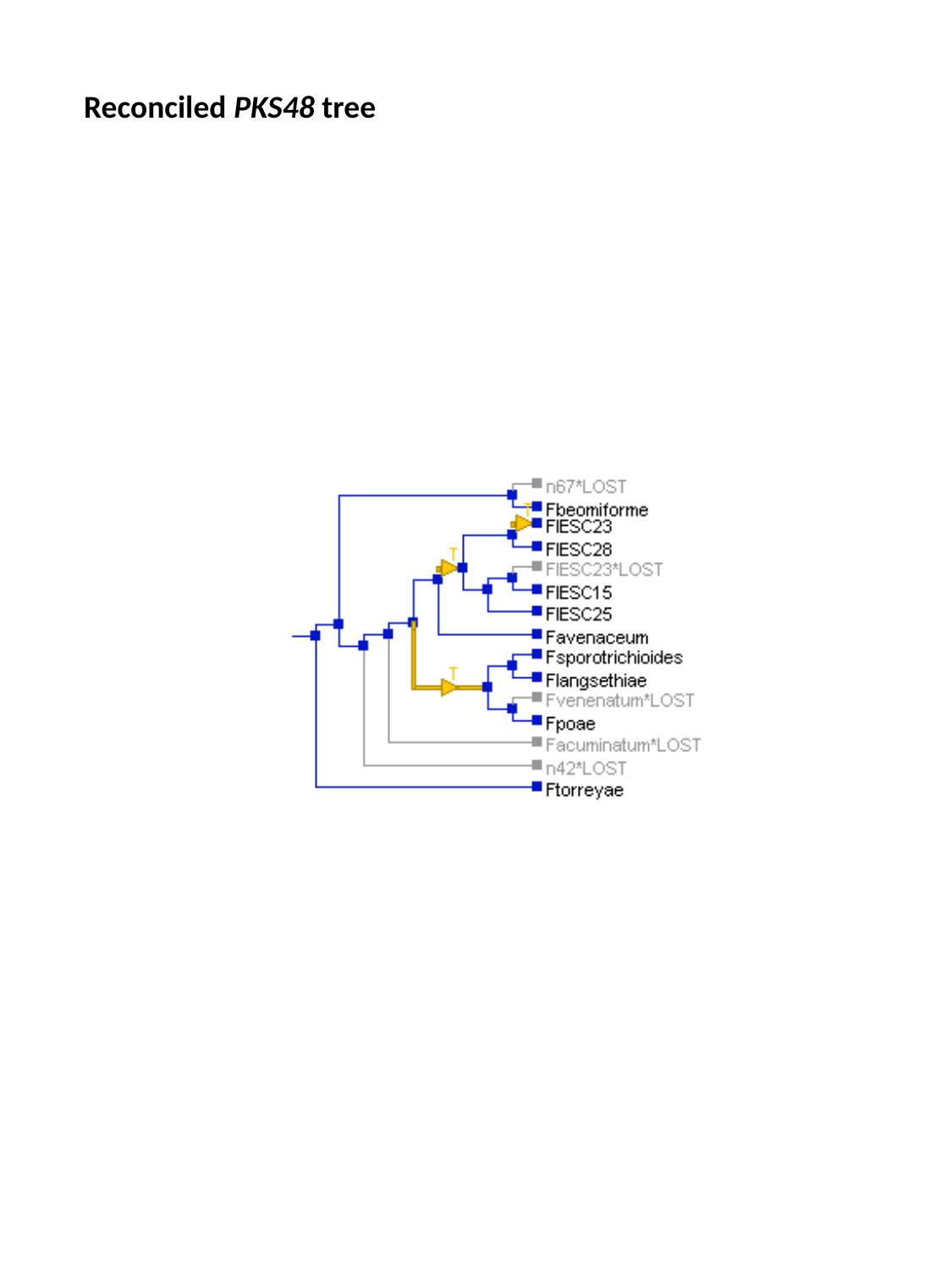

Reconciled PKS48 tree

## Slide 37
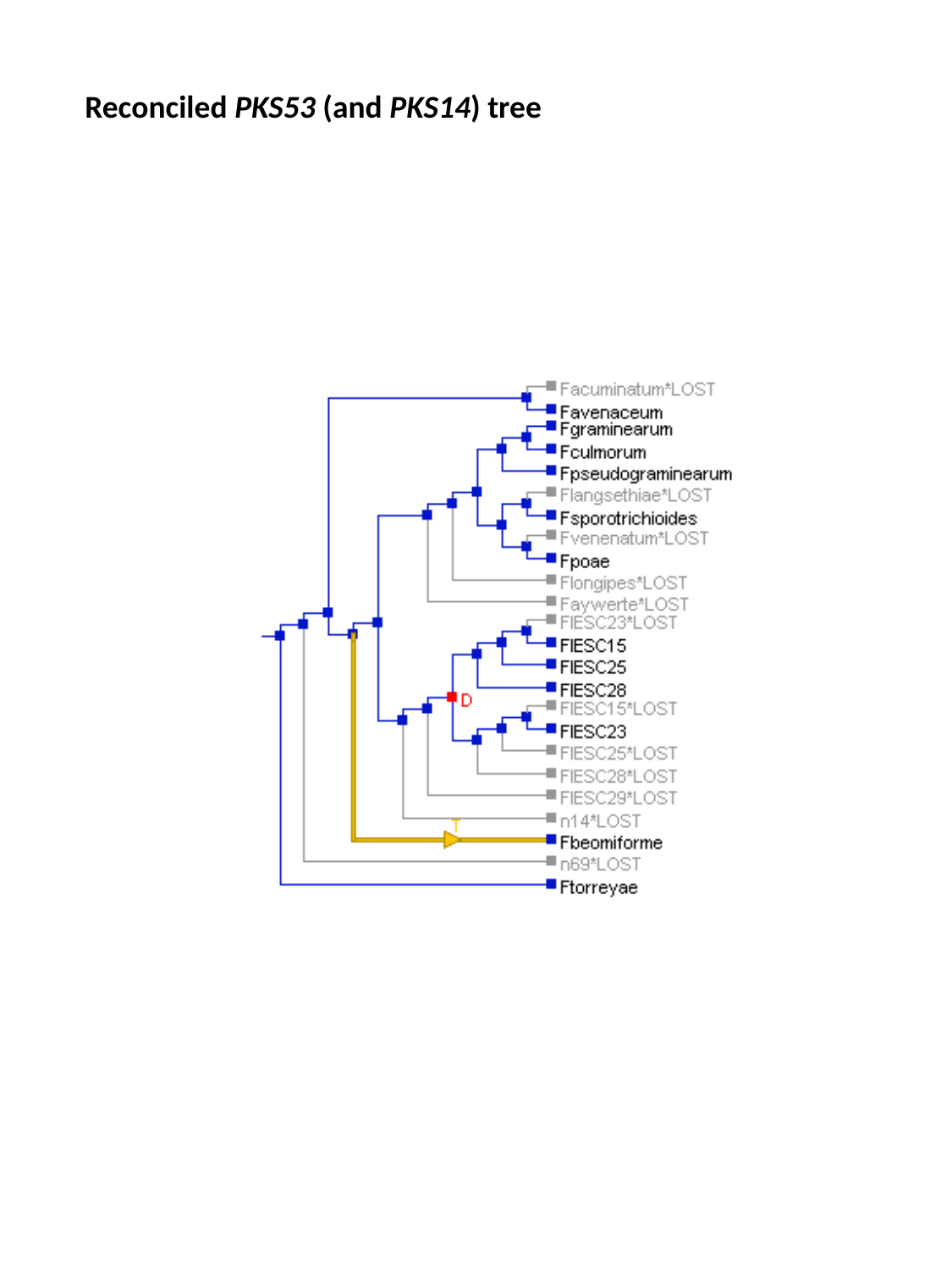

Reconciled PKS53 (and PKS14) tree

## Slide 38
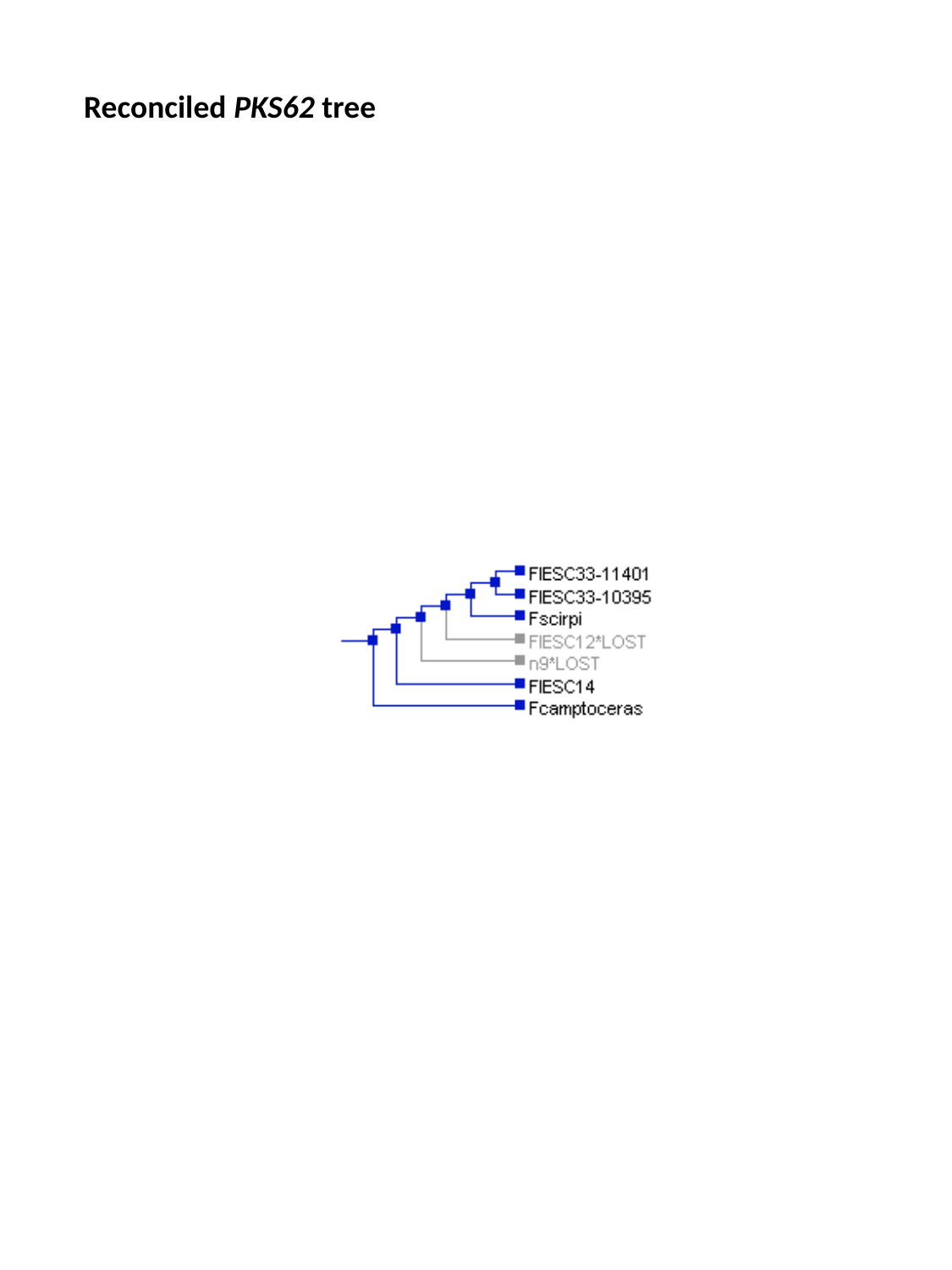

Reconciled PKS62 tree

## Slide 39
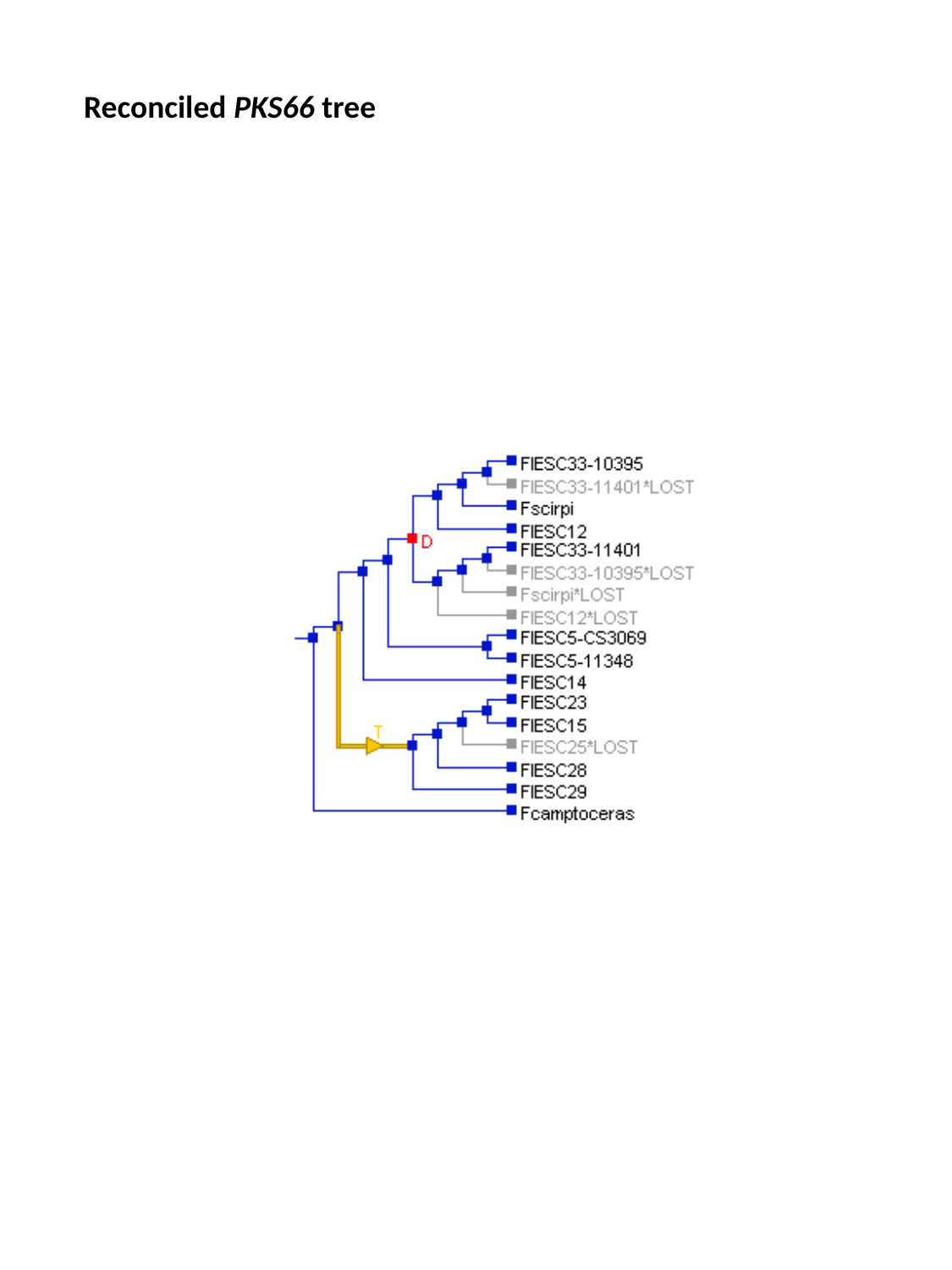

Reconciled PKS66 tree

## Slide 40
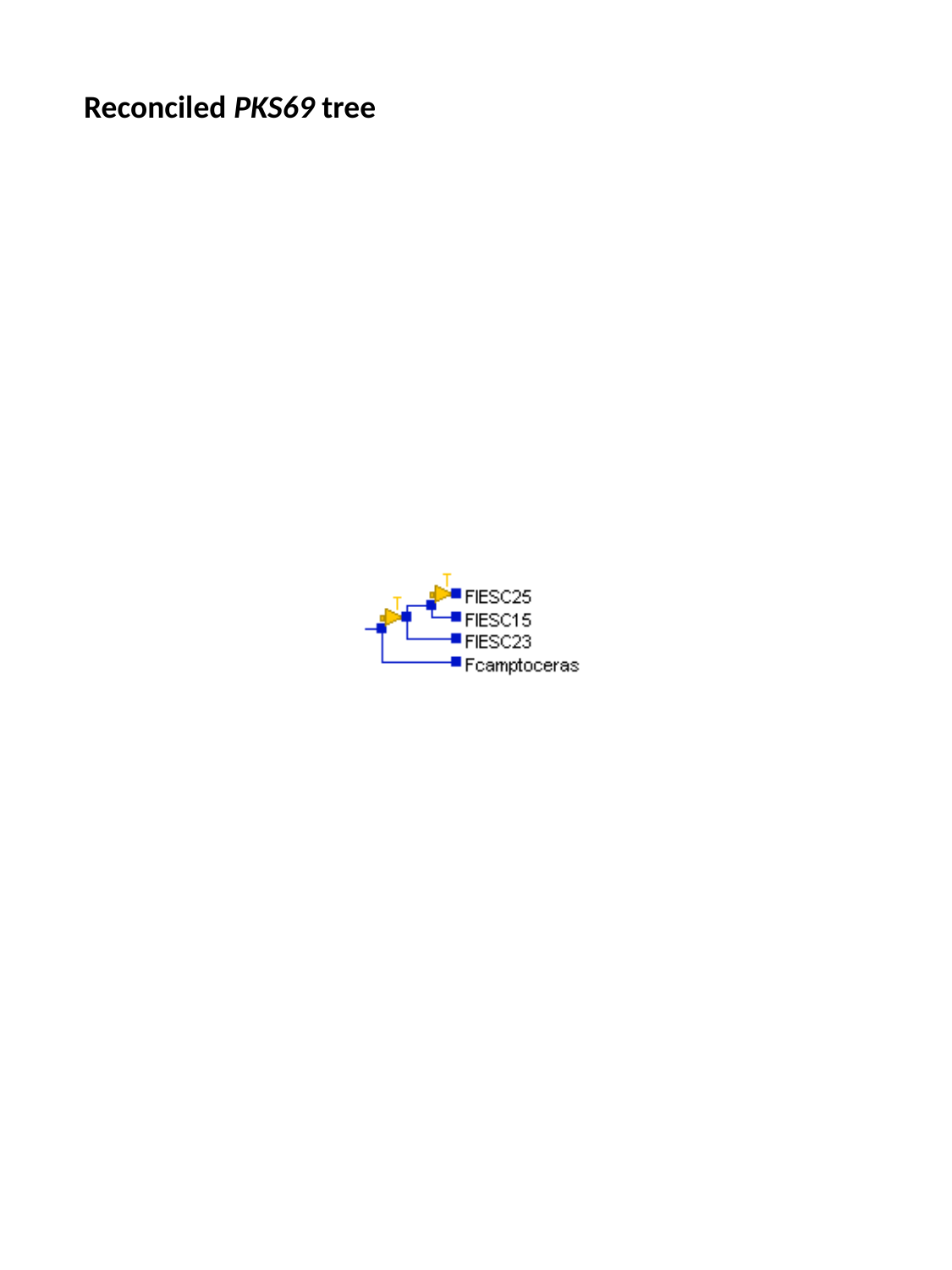

Reconciled PKS69 tree
