# Supplementary material for: Variation in secondary metabolite production potential in the Fusarium incarnatum-equiseti species complex revealed by comparative analysis of 13 genomes
Source: BMC Genomics. 2019 Apr 24;20:314. doi: 10.1186/s12864-019-5567-7 (PMC6480918; doi:10.1186/s12864-019-5567-7)
Supplement: Supplementary file 7 — Results of Shimodaira-Hasegawa (SH) and Approximately Unbiassed (AU) tests used to assess branches that suggest horizontal gene transfer of NRPS and PKS genes between FIESC and other lineages of Fusarium. (DOCX 21 kb) [file 12864_2019_5567_MOESM7_ESM.docx]

**Additional file 7**

**Tree^a^ log Likelihood p-SH^b^ p-AU^b^**

***NRPS4***

Unconstrained -320114.626 1.0000 1.0000

Constrained FIESC-Sambucinum-*F. aywerte* clade -321123.891 0.0000* 0.0000*

***NRPS11***

Unconstrained -30646.236 1.0000 0.5309

Constrained Sambucinum-*F. aywerte* clade -30646.767 0.4683 0.4691

***NRPS14***

Unconstrained -62701.085 1.0000 1.0000

Constrained Sambucinum-*F. aywerte* clade -6258.202 0.0000* 0.0000*

***NRPS16***

Unconstrained -24121.771 0.5033 0.5002

Constrained FIESC clade and Sambucinum-*F. aywerte* clade -24121.771 1.0000 0.4998

***NRPS19***

Unconstrained -25799.574 1.0000 0.5896

Constrained FIESC clade -25802.410 0.4030 0.4104

***NRPS22***

Unconstrained -78378.307 1.0000 1.0000

A. Constrained Fujikuroi-Nisikadoi-Oxysporum-*F. beomiforme* clade -78826.926 0.0000* 0.0000*

B. Constrained FIESC-Sambucinum-*F. aywerte* clade -79509.600 0.0000* 0.0018*

***NRPS34***

Unconstrained -107066.705 1.0000 0.5052

Constrained FIESC clade -107066.778 0.5011 0.4948

***PKS10-FUS1***

Unconstrained -108050.444 1.0000 0.9996

A. Constrained FIESC clade -110730.861 0.0000* 0.0000*

B. Constrained Sambucinum-*F. aywerte* clade -108132.339 0.0673 0.0000*

***PKS22***

Unconstrained -68381.569 1.0000 0.9999

A. Constrained Fujikuroi-Nisikadoi-Oxysporum clade -68571.439 0.0000* 0.0001*

B. Constrained all taxa to one clade except *F. torreyae* -68495.153 0.0000* 0.0000*

& F. solani

***PKS23***

Unconstrained -37152.239 1.0000 0.9977

Constrained FIESC-Sambucinum clade -37493.952 0.0000* 0.0023*

***PKS42***

Unconstrained -19619.141 1.0000 0.4919

Constrained FIESC clade -19619.141 0.5016 0.5081

***PKS43***

Unconstrained -19424.095 1.0000 0.9149

Constrained FIESC clade -19430.711 0.1053 0.0851

***PKS48***

Unconstrained -49177.266 1.0000 0.9997

Constrained FIESC-Sambucinum clade -49274.525 0.0000* 0.0003*

***PKS69***

Unconstrained -38365.3321 0.0000 1.0000

Constrained *F. dlaminii*-*F. fujikuroi* clade -38528.119 0.0000* 0.0000*

**^a^** The following are descriptions of how individual NRPS/PKS gene trees were constrained for the SH and AU tests. *NRPS4*: FIESC, *F. aywerte* and the Sambucinum complex were constrained to the same clade; all other taxa were unconstrained. *NRPS11* and *NRPS14*: *F. aywerte* and the Sambucinum complex were constrained to the same clade; all other taxa were unconstrained. *NRPS16*: FIESC taxa were constrained to one clade, and Sambucinum complex taxa were constrained to another clade. *NRPS19*, *NRPS34*, *PKS42*, *PKS43*: FIESC taxa were constrained to the same clade; all other taxa were unconstrained. *NRPS22*: in constrained tree A, *F. beomiforme* and the Fujikuroi, Nisikadoi, Oxysporum complexes were constrained to the same clade, while all other taxa were unconstrained; and in constrained tree B, *F. aywerte*, FIESC and the Sambucinum complex were constrained to the same clade, while all other taxa were unconstrained. *PKS10*: in constrained tree A, FIESC taxa were constrained to the same clade, while all other taxa were unconstrained; and in the constrained tree B, *F. aywerte* and the Sambucinum clade were constrained to the same clade, while all other taxa were unconstrained. *PKS22*: in constrained tree A, the Fujikuroi, Nisikadoi and Oxysporum complexes were constrained to the same clade, while all other taxa were unconstrained; and in constrained tree B, all taxa except *F. torreyae* and *F. solani* were constrained to the same clade. *PKS23* and *PKS48*: FIESC and the Sambucinum complex were constrained to the same clade; all other taxa were unconstrained. *PKS69*: *F. dlaminii* and *F. fujikuroi* were constrained to the same clade; all other taxa were unconstrained.

**^b^** Probability scores for SH and AU tests as implemented in IQ-Tree (version 1.5.5). An asterisk (*) indicates that the constrained tree is significantly worse than the unconstrained tree (p < 0.05).
