# Supplementary figures and images for: Variation in secondary metabolite production potential in the Fusarium incarnatum-equiseti species complex revealed by comparative analysis of 13 genomes
Source: BMC Genomics. 2019 Apr 24;20:314. doi: 10.1186/s12864-019-5567-7 (PMC6480918; doi:10.1186/s12864-019-5567-7)

## Slide 1
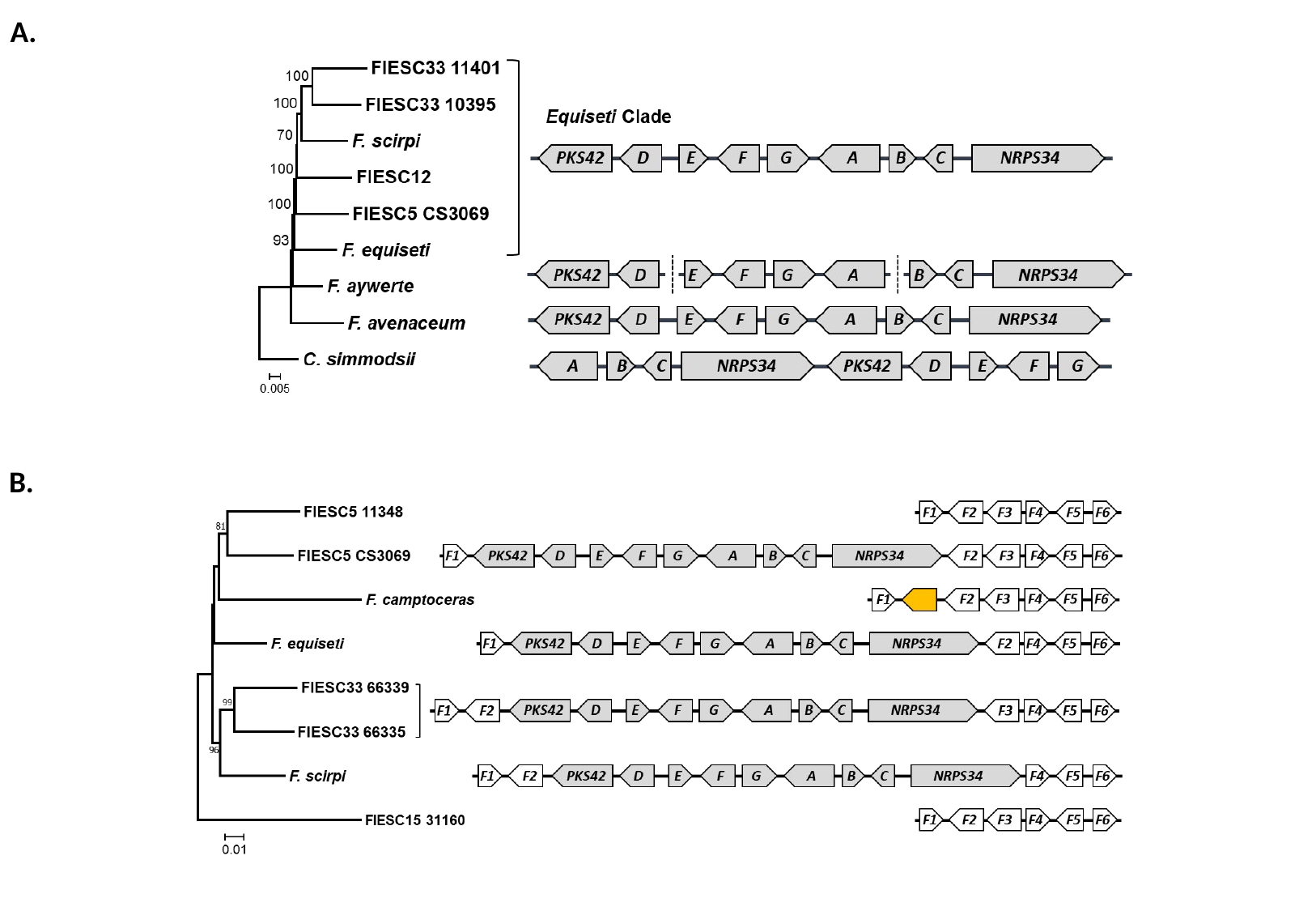

A.
B.

Supplement: Supplementary file 9 — A. Analysis of homologs of the putative PKS42-NRPS34 cluster. On the left, tree inferred by maximum likelihood analysis of concatenated sequences of the nine genes in the PKS43-NRPS34 cluster. Numbers near branches are bootstrap values based on 1000 pseudoreplicates. Right, gene organization in PKS42-NRPS34 cluster homologs. The vertical dashed lines between genes in the F. aywerte homolog indicate that the genes are one different contigs. The genes on either side of a vertical lines are at the ends of contigs and, therefore, could be adjacent to one another in the genome. B. Analysis of genes flanking the putative PKS42-NRPS34 cluster and their homologs in selected members of FIESC that lack the cluster. The tree to the left was inferred by maximum likelihood analysis of flanking gene F2. Numbers near branches are bootstrap values based on 1000 pseudoreplicates. Bootstrap values below 70% are not shown. Genes and their direction of transcription are represented by arrows. Gray arrows represent genes in the PKS42-NRPS34 cluster, white arrows represent cluster flanking genes, and the yellow arrow represents a gene that was unique to the region in F. camptoceras. (PPTX 154 kb) [file 12864_2019_5567_MOESM9_ESM.pptx]
